# The Assessment of the Readiness of Molecular Biomarker-Based Mobile Health Technologies for Healthcare Applications

**DOI:** 10.1038/srep17854

**Published:** 2015-12-08

**Authors:** Chu Qin, Lin Tao, Yik Hui Phang, Cheng Zhang, Shang Ying Chen, Peng Zhang, Ying Tan, Yu Yang Jiang, Yu Zong Chen

**Affiliations:** 1 Shenzhen Kivita Innovative Drug Discovery Institute, and the Ministry-Province Jointly Constructed Base for State Key Lab-Shenzhen Key Laboratory of Chemical Biology, the Graduate School at Shenzhen, Tsinghua University, Shenzhen, P. R. China; 2 Department of Pharmacy, and Center for Computational Science and Engineering, National University of Singapore, 117543 Singapore; 3 NUS Graduate School for Integrative Sciences and Engineering, National University of Singapore, 117456, Singapore; 4 Computation and Systems Biology, Singapore-MIT Alliance, National University of Singapore, Singapore

## Abstract

Mobile health technologies to detect physiological and simple-analyte biomarkers have been explored for the improvement and cost-reduction of healthcare services, some of which have been endorsed by the US FDA. Advancements in the investigations of non-invasive and minimally-invasive molecular biomarkers and biomarker candidates and the development of portable biomarker detection technologies have fuelled great interests in these new technologies for mhealth applications. But apart from the development of more portable biomarker detection technologies, key questions need to be answered and resolved regarding to the relevance, coverage, and performance of these technologies and the big data management issues arising from their wide spread applications. In this work, we analyzed the newly emerging portable biomarker detection technologies, the 664 non-invasive molecular biomarkers and the 592 potential minimally-invasive blood molecular biomarkers, focusing on their detection capability, affordability, relevance, and coverage. Our analysis suggests that a substantial percentage of these biomarkers together with the new technologies can be potentially used for a variety of disease conditions in mhealth applications. We further propose a new strategy for reducing the workload in the processing and analysis of the big data arising from widespread use of mhealth products, and discuss potential issues of implementing this strategy.

There have been intensifying efforts to explore mobile health (mhealth) technologies for delivering healthcare at reduced costs and for facilitating more precise and personalized medicine^1^,^2^,^3^ which have led to 73 apps endorsed (examples in Table 1, a complete list in Supplementary Table S1) and additional ones reviewed^1^ by the US Food and Drug Administration (FDA) for self-diagnosing acute diseases and monitoring chronic conditions^1^ based on such physiological biomarkers as body temperature and brainwave^4^,^5^, and such simple-analyte biomarkers as glucose and urine protein contents^4^,^5^.

Although these physiological and simple-analyte biomarkers cover many disease conditions, their coverage is substantially limited for such prevalent diseases as cancers, infectious, respiratory, digestive, endocrine and nervous system diseases, as indicated by the disease-coverage profiles of the 73 FDA endorsed, and 94 physiological and simple-analyte biomarker candidates described in the literatures (Fig. 1, Table 1 and 2, Supplementary Table S1 and S2). Apart from the development of more portable biomarker detection technologies, additional biomarkers are needed for fulfilling the tasks of mhealth technologies as efficient and effective means for providing wider coverage of healthcare and personalized treatments at reduced costs^1^,^2^,^3^.

Some genetic, proteomic and metabolomic molecular biomarkers have been clinically used and many more such molecular biomarker candidates (hitherto also tentatively named biomarkers) have been discovered for diagnosing and monitoring diseases, directing treatments and predicting patient responses^6^,^7^,^8^. Of immediate relevance to mhealth are the hundreds of literature-reported non-invasive and minimally-invasive diagnostic, prognostic and theragnotic molecular biomarkers from such non-invasive sources as urine, breath, saliva, tear, feces, sputum and oral mucosa samples (Examples in Table 3 and complete list in Supplementary Table S3) and from such minimally-invasive sources as finger-prick (the list of serum biomarkers potentially detectable from finger-prick is in Supplementary Table S4), which significantly expand the disease coverage as indicated by the disease-coverage profiles of the 664 (27 clinical trial) non-invasive and 592 serum (69 clinical trial or use) molecular biomarkers with respect to those of 73 FDA endorsed apps and 94 physiological and simple-analyte biomarkers (Fig. 1). Many biomarkers are detectable by the new biomarker-detection technologies that become increasingly portable, faster, user-friendly, inexpensive and accurate^9^,^10^,^11^, some of which have been explored for potential mhealth applications^9^,^12^,^13^,^14^,^15^.

From the investigations and opinions described in the literatures listed in Supplementary Table S3, there are good reasons to speculate the readiness of some of these technologies for mhealth applications. But before the acceptance and widespread utilization of these technologies, several key questions need to be answered or resolved. Apart from the development of more portable biomarker detection technologies, an important question is whether the new portable biomarker detection technologies are sufficiently sensitive, fast, convenient and inexpensive for biomarker detection in the typical mhealth settings (low sample volume and biomarker concentrations). Another question is whether the discovered and investigative molecular biomarkers extracted from the non-invasive and minimally invasive sources are relevant to mhealth applications in terms of the detection accuracies and the coverage of disease conditions and patient populations. The third is how to resolve the different readings generated from different mhealth devices and variations in individual operations. The fourth is how to manage the heavy workload in processing and analysing the big data arising from widespread use of mhealth devices.

Here, we address some of these questions by analysing (1) biomarker detection capability of the literature-reported new technologies with specific focus on their detection sensitivity, required sample volume, test time, and costs with respect to experimentally-determined biomarker levels in patients and the detection limits, and (2) the disease coverage, patient populations, and the diagnostic, prognostic, and theragnostic sensitivity and specificity of the literature-reported non-invasive and minimally-invasive finger-prick molecular biomarkers for mhealth applications with respect to the detection limits of the new detection technologies. We also discuss the feasibility and practical issues of adopting a new strategy for reducing the heavy workload of mhealth data processing by automated electronic pre-screening of the big biomarker screening data.

## Literature Search

The detailed information of 73 mhealth apps endorsed by the US FDA was obtained by manually checking the descriptions of the apps listed in FDA 510(k) medical device database^16^. The physiological and molecular biomarkers were obtained by the comprehensive literature search of the Pubmed database by using the combination of the keyword “biomarker” together with one of the keywords of “clinical”, “patient”, “disease”, “drug”, and specific disease names such as “cancer”, “inflammation” and “hypertension”. We also searched and evaluated biomarker review papers from reputable journals by using the combination of the keywords “biomarker” and “review”, with the cited original articles checked to collect detailed information about the discussed biomarker, such as the name, source, specific disease and function, specificity and sensitivity of the biomarker. The detailed information of these 254 evaluated review and research papers are listed in Supplementary Table S6. Additional sources such as the abstracts of the American society of clinical oncology were also systematically searched, with 658 biomarker conference abstracts in 1995–2013 extracted and evaluated by data mining and manual curation. Non-invasive biomarkers were selected if they were detected in non-invasive tissues such as urine, breath, saliva, tear, feces, sputum and oral mucosa samples. The information of disease conditions was searched from the websites of professional medical associations such as WHO^17^ and American Cancer Society^18^, and such additional sources as reputable books and review articles, using combinations of keywords such as the disease name and “prevalence” or “incidence”. These biomarkers were organized based on their international classification ICD-10 codes^19^ and were displayed with respect to these codes in a tree graph by using the automatic tree generator module in iTOL^20^.

The performance of the biomarkers in diagnosing, prognosing or theragnosing specific conditions has been statistically measured by sensitivity (the proportion of the condition-positive samples that are correctly identified as positive) and specificity (the proportion of the condition-negative samples that are correctly identified as negative)^21^. Wherever reported in the literature, these statistical performance measures were recorded. Apart from the collection of the biomarker detection technologies described in our searched biomarker literatures, additional literature search was conducted for searching biomarker detection technologies of potential mhealth applications by using the keyword “biomarker” in combination with one of the keywords “detection”, “detector”, “device”, “technology”, “technique” and “assay”. These detection technologies were analysed for selecting those with potential mhealth applications based on their detection performance, portability, detection time, cost and ease of use.

## New technologies for detecting non-invasive molecular biomarkers and their relevance to mhealth

The new biomarker-detection technologies combined with mobile phone or the equivalent imaging devices have been explored for detecting at least 23 molecular biomarkers including 11 non-invasive ones (Table 4). These new technologies can be categorized into four groups: (1) paper-based and mobile phone enabled, (2) paper-based, (3) mobile-phone enabled, and (4) the other point of care technologies. The first group of technologies combines innovative paper-based microfluidic analytical technologies with mobile phone enabled automated image processing tools, which are most relevant to mhealth applications because of the very low cost (~$2.60+ cost plus mobile phone), increasingly enhanced detection sensitivity (0.3–60 ng/mL, 0.13–21.3 μg/mL and 0.81–2000 ng/mL for small molecule, peptide and protein biomarkers respectively), low sample volumes (0.5–25 μL), short detection time (15–60 mins), and the convenient biomarker processing (mobile phone-based) capabilities. The recently developed paper-based microfluidic analytical technologies include paper-based enzyme-linked immunosorbent assays (P-ELISA)^9^,^22^, paper lateral flow immunoassays (P- LFIAs)^12^,^23^, and paper-based Au-nanoprobes^22^. These are integrated with or coupled to mobile phones equipped with the colorimetric algorithms^22^ and the applications for immediate data processing of the detection results without referring to peripheral equipment for read-out and analysis^9^.

The second group of technologies primarily employ innovative P-ELISA in combination with a scanner, printer or digital camera based image-processing facility to achieve a detection sensitivity as high as 33.7 fg/mL^24^ and 18 pM/mL^25^ for detecting peptide and protein biomarker respectively. The imaging processing component of these technologies may be potentially replaced by mobile phone-based ones for potential mhealth applications. The third group of technologies integrates mobile phone imaging processing tools with newly developed disposable microfluidic chip^26^, opto-acoustic immunoassay^27^, microfluidic capillary array equipped with optical signal amplifier^28^, microtiterplate based ELISA^29^ and other technologies. These technologies achieve detection sensitivity up to the level of 60–300 pg/mL for protein biomarkers^29^,^30^. Although their costs are more suitable for point of care (POC) rather than mhealth applications, the innovative design may be potentially implemented into paper-based platforms for more extensive mhealth applications. A new POC technology in the fourth group, the negative-pressure-driven microfluidic chip magnetic bead based ELISA, is capable of detecting a small molecule biomarker at sensitivity level of 0.3 ng/mL^31^,^32^. If implemented into paper-based and mobile phone-enabled platforms, this technology may potentially find wider applications for detecting small molecule biomarkers in mhealth.

Overall, 12 or 52.2% of the 23 tested molecular biomarkers are detectable by these new technologies at low concentrations (0.3–810 pg/mL and 4–50 ng/mL for 8 and 4 biomarkers respectively). Although the detectable concentrations of these 23 biomarkers are roughly 10-fold higher than those of the conventional technologies^24^, seven of them are nonetheless within the lower detection limit of the new technologies for non-invasive detection^24^,^27^. Of the eight biomarkers with available patient data, only two biomarkers in the corresponding non-invasive source are outside the detection limit of the new technologies. Moreover, 64.3% of these biomarkers are detectable at significantly lower sample volumes (0.5–12 μL) and shorter time (10–60 min) than the volumes (100–300 μL)^13^,^25^ and durations (up to 4h)^24^ of the conventional technologies. The costs of these detection devices are ~$300–$600 US dollars^33^. The per-test costs are in the range of 0.01–190. Therefore, the new technologies are fairly sensitive, efficient, and inexpensive for detecting a substantial percentage of the tested non-invasive biomarkers, and there is high likelihood that they can be applied for detecting other non-invasive biomarkers in mhealth applications.

## The non-invasive molecular biomarkers and their relevance to mhealth

Analysis of the 664 literature-reported non-invasive molecular biomarkers (examples in Table 5 and a complete list in Supplementary Table S5) showed that 546 and 183 biomarkers are for the diagnosis and prognosis of 85 and 45 disease conditions respectively, with 31 and 14 (or 36.5% and 31.1%) of the disease conditions covered by higher number (4–22) of biomarkers and 10 and 6 (or 11.8% and 13.3%) of the disease conditions by clinically-validated/evaluated biomarkers. Among these, 21 acute diseases and 11 chronic conditions affect large populations of 239,000–235 million and 10–235 million people respectively. Therefore, exploration of these biomarkers may significantly improve the efficiency of the management of these disease conditions.

The diagnostic performance of 88 (or 29.7%) of the 296 diagnostic biomarkers for 43 diseases and the prognostic performance of 24 (25.5%) of the 94 prognostic biomarkers for 14 conditions have been reported in the literature (examples in Tables 3 and 5 and a complete list **in** Supplementary Table S3, S5) Their performances have been typically measured by sensitivities (the rates for positive identification of disease conditions) and specificities (the rates for correct classification of the negatives). The sensitivities and specificities of the majority of these biomarkers are ≥85% and ≥80% for diagnosis, and ≥80% and ≥80% for prognosis respectively, which are roughly at the ≥90% sensitivity and ≥90% specificity levels of the good biomarkers^21^. Therefore, a substantial percentage of these non-invasive biomarkers are expected to be potentially useful for pre-screening patients in need of further evaluations in mhealth applications.

The utility of these biomarkers for mhealth applications also depends on whether they are detectable by the new detection technologies, i.e., whether the levels of these biomarkers in the non-invasive sources from the patients are within the detection range of the new detection technologies. We searched from the literatures the corresponding biomarker levels for 35 diseases (Supplementary Table S5, examples in Table 5) and compared them to the detection limits of the new technologies. Our analysis showed that 26 (or 74.3%) of the 35 disease conditions with searchable information, including 8 disease conditions with large patient populations, have one or more biomarker detectable by the new technologies (Table 5), suggesting that a substantial percentage of the disease conditions including those with large patient populations may be partly covered by the new technologies.

## The potential of the minimally invasive finger-prick biomarker technologies for mhealth applications

**Figure 1:**
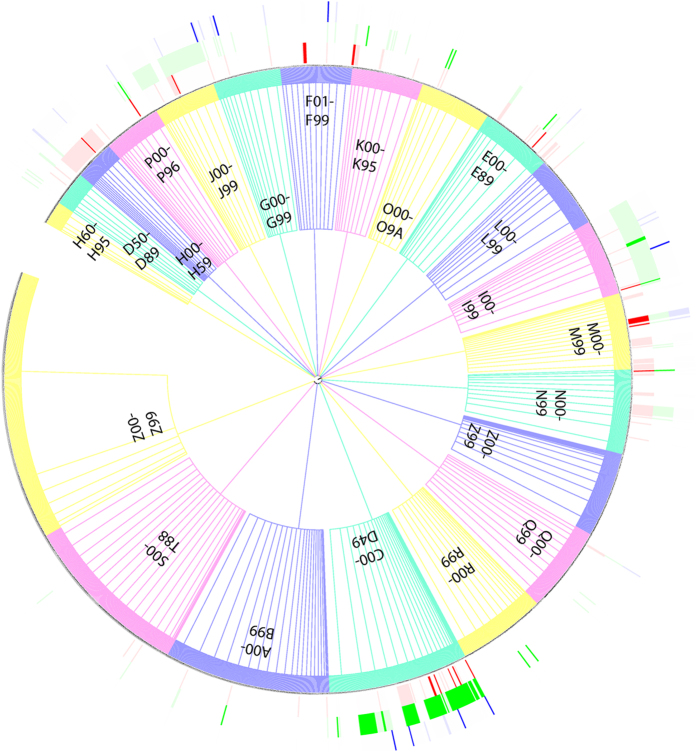
Disease-coverage profiles of the biomarkers. 664 (27 in clinical trial or use) non-invasive molecular biomarkers are colored in light (deep) red. 592 (69 in clinical trial or use) non-invasive molecular biomarkers are colored in light (deep) green. The 94 (13 in clinical trial or use and 73 FDA endorsed apps) physiological and conventional biomarkers are colored in light (deep) blue. Each leaf in the tree represents a specific ICD code as follows: A00-B99: infectious and parasitic diseases, C00-D49: Neoplasms, D50-D89: Diseases of the blood and related organ and immune disorders, E00-E89: Endocrine, nutritional and metabolic diseases, F01-F99: Mental, Behavioral and Neurodevelopmental disorders, G00-G99: nervous system disorders, H00-H59: eye and adnexa diseases, H60-H95: Diseases of the ear and mastoid process, I00-I99: circulatory system disorders, J00-J99: respiratory system disorders, K00-K95: digestive system disorders, L00-L99: skin and subcutaneous tissue disorders, M00-M99: musculoskeletal system and connective tissue disorders, N00-N99: genitourinary system disorders, O00-O9A: Pregnancy, childbirth and the puerperium, P00-P96: conditions originating in the perinatal period, Q00-Q99: Congenital malformations, deformations and chromosomal abnormalities, R00-R99: conditions not elsewhere classified, S00-T88: Injury, poisoning and certain other consequences of external causes, V00-Y99: External causes of morbidity, Z00-Z99: Factors influencing health status and contact with health services

The minimally invasive finger-prick biomarker technologies have been developed for POC applications^11^. Because of their improved detection performance^34^, portability^35^ and ease of use^36^, and because of their decreased detection time^34^, some of these technologies when combined with smartphone-based processing technologies may find potential mhealth applications. Serum biomarkers are known to be detectable at finger-prick albeit at altered concentrations and thus at re-adjusted detection cut-off values^37^,^38^. Therefore, one can hypothesize that most of the serum biomarkers of sufficient level of concentrations may be potentially detectable by finger-prick biomarker technologies. The application of these technologies in mhealth significantly expands the coverage of disease conditions because some biomarkers not found in urine are in the serum (e.g. it has been reported that the blood contains the common markers of liver function that are not found in urine^35^). Our own literature search results showed that the literature-reported serum biomarkers and biomarker candidates cover additional 62 disease conditions beyond those covered by the existing physiological, simple-analyte, and the non-invasive molecular biomarkers and biomarker candidates (Fig. 1 and Supplementary Table S4).

Moreover, the finger-prick biomarker technologies can potentially have more enhanced capabilities in detecting the biomarkers of low concentrations. The levels of biomarkers in blood are typically more concentrated than those biomarkers collected from the non-invasive urine, breath, saliva, tear, feces, sputum or oral mucosa sources{Song, 2014 #89} {Abdalla, 2012 #115}. For those biomarkers with concentrations in the non-invasive and finger-prick sources below and above the detection limit of the mhealth biomarker technologies respectively, some of them are potentially detectable by using finger-prick biomarker technologies even if they are undetectable by the non-invasive biomarker technologies.

Several new technologies have been developed with potential applications for detecting serum biomarkers from a drop of blood (Table 4). To enable the purification and detection of serum biomarkers, specially designed fluid handling and silver reduction devices have been combined with the ELISA microfluidic chip for simplified biomarker detection, which enables the detection of an HIV biomarker from 1 μl of unprocessed whole blood in <15 min^39^. In another design, a microfluidic purification chip was developed for simultaneously capturing multiple biomarkers from blood samples and releasing them into purified buffer for sensing by a silicon nanoribbon detector, which was able to detect two model cancer antigens from a 10 ml sample of whole blood in <20 min^40^. A micropatterned paper device that combines a filter membrane and a patterned paper chip for achieving blood plasma erythrocyte separation and biomarker detection from the blood from a fingerstick, which is capable of detecting protein biomarkers at ~50 g/L concentrations^35^. Progress has been made in developing plasmonic ELISA for the ultrasensitive detection of disease biomarkers with the naked eye with the ability to detect biomarkers in whole serum at the ultralow concentration of 10^−18^ g mL^−1^ 41.

We have found the reports about the detection of 12 serum biomarkers by means of these new technologies (Table 4). Overall, 5 or 42% of the 12 biomarkers are detectable at concentrations of <1.5 ng/mL. Considering that many serum biomarker concentrations are higher than those collected from the urine or other non-invasive sources, the relevant technologies may be extended for the detection of a more variety of low concentration biomarkers than those coverable by the non-invasive biomarker technologies. These technologies enable serum biomarker detection mostly at low sample volumes of 1–10 uL and short time of 12–30 min comparable to those of the non-invasive biomarker technologies. The cost of a microtiterplate based ELISA device coupled with a smartphone is <$660^29^. The per test costs of these technologies are in the range of $0.1–34. Three studies reported the sensitivity and specificity of five serum biomarkers, which are in the range of 82–100% (vast majority >90%) and 78%-100% respectively^38^,^39^,^42^. Therefore, these new technologies are fairly sensitive, efficient, and inexpensive for detecting a substantial percentage of the tested serum biomarkers with potential mhealth applications.

## Coping with the heavy workload in mhealth: Feasibility of automated electronic pre-screening of big mhealth data

**Figure 2:**
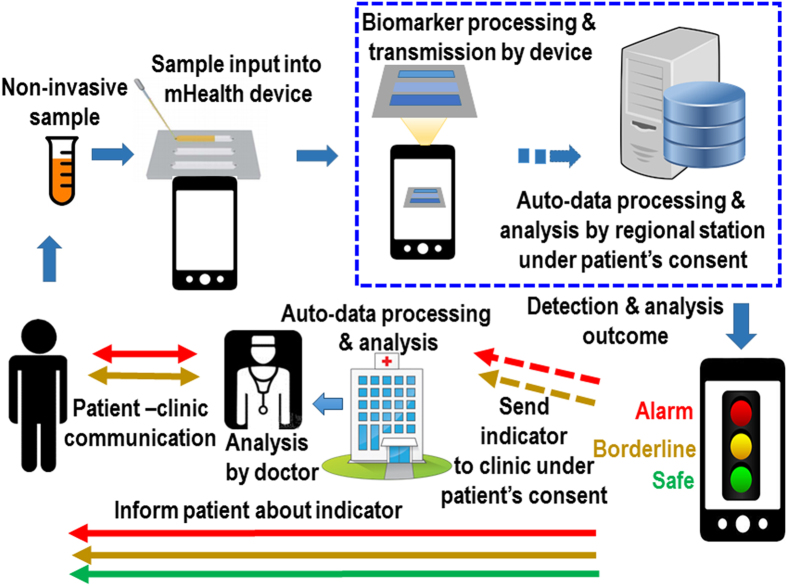
Flow chart of mhealth biomarker detection and automated data processing procedures. (Figure drawn by C.Q.).

There are concerns about the increased workload in processing and analysing the big data arising from widespread use of mhealth devices^1^. On the hand, mhealth devices as digital tools may conveniently facilitate electronic pre-screening of the biomarker readings for filtering potential patients likely in need of further attention and evaluation, which helps to significantly reduce the workload. A digitally-coded biomarker, disease and therapeutic information processing system may be developed for automatically receiving, processing, pre-screening, and dispatching the biomarker readings transmitted from mhealth devices (Fig. 2).

It is feasible to develop such a system using available tools such as the International Classification of Diseases (ICD) codes for defining, studying and managing diseases and treatments^43^, the Systematized nomenclature of medicine for clinical documentation and reporting^44^, the Unified medical language system for biomedical terminology^45^, the Therapeutic target database biomarker and target information and links to the ICD and drug codes^46^, and the Drugbank drug information^47^. Further efforts are needed for additional information refinement and integration, determination and clinical validation of biomarker pre-screening thresholds, and development and education of testing protocols. There are also potential issues arising from missed detection or misidentification by an electronic system, lack of data security and insufficient regulation standards.

## Concluding Remarks

Molecular biomarker-based mobile health technologies have the potential to significantly improve the efficiency and quality of healthcare for a variety disease conditions particularly those with large patient populations that cannot be solely covered by physiological and simple-analyte biomarkers. Some of these biomarkers combined with the new detection technologies are readily applicable for mhealth applications. The increased workload in processing and analyzing high volumes of mhealth data may be efficiently managed by an electronic system that facilitate automatic pre-screening and analysis of the biomarker data for filtering potential patients likely in need of further attention and evaluation.

## Additional Information

**How to cite this article**: Qin, C. *et al.* The Assessment of the Readiness of Molecular Biomarker-Based Mobile Health Technologies for Healthcare Applications. *Sci. Rep.* **5**, 17854; doi: 10.1038/srep17854 (2015).

## Supplementary Material

Supplementary Table S1

Supplementary Table S2

Supplementary Table S3

Supplementary Table S4

Supplementary Table S5

Supplementary Table S6

## Figures and Tables

**Table 1:** Examples of FDA endorsed mobile apps. (For a complete list, please refer to Supplementary Table S1).

**Device Name**	**Applicant**	**510(k) Number**	**Type**	**Measure**	**Disease**
Airstrip Ob	Airstrip Technologies, Lp	K090269	Monitoring	Fetal Heart Tracings; Maternal Contraction Pattern	Obstetrics/Gynecology
Alivecor Heart Monitor For Iphone	Alivecor, Inc.	K122356	Monitoring	Ecg	Cardiovascular
Beam Brush/Beam App	Beam Technologies, Llc	K121165	Monitoring	Brushing Usage Data	Tooth Decay
Bodyguardian System Bodyguardian Control Unit Bodyguardian Connect	Preventice, Inc.	K121197	Monitoring	Ecg; Activity; Heart Rate; Respiration Rate	Cardiovascular
Cg-6108 Arrhythmia Ecg Event Recorder	Card Guard Scientific Survival, Ltd.	K060911	Monitoring	Ecg	Cardiac Arrhythmia
Customized Sound Therapy (Cst)	Tinnitus Otosound Products, Llc	K070599	Treatment		Tinnitus
Freestyle Tracker Diabetes Management System	Abbott Diabetes Care Inc.	K020866	Monitoring	Glucose	Diabetes
Fully Automatic Wireless Blood Pressure Wrist Monitor	Andon Health Co., Ltd	K121470	Monitoring	Blood Pressure	Cardiovascular
Iglucose System	Positiveid Corporation	K111932	Monitoring	Glucose	Diabetes
Intuition	Terarecon, Inc.	K121916	Data Viewer	Ebt, Ct, Pet Or Mri Image	
Kd-936 Fully Automatic Wireless Blood Pressure Monitor	Andon Health Co.,Ltd	K120672	Monitoring	Blood Pressure	Cardivascular
Medicalgorithmics Real-Time Ecg Monitor And Arrhythmia Detector, Model Pocketecg	Medicalgorithmics Sp Z.O.O.	K090037	Monitoring	Heart Beat, Rhythm Abnormalities	Cardivascular
Mobile Mim	Mim Software Inc.	K112930	Data Viewer	Spect, Pet, Ct, Mri, X-Ray And Ultrasound	
Myglucohealth Glucose Monitoring Systems	Entra Health Systems, Ltd.	K081703	Monitoring	Glucose	Diabetes
Myvisiontrack(Tm)	Vital Art And Science Incorporated	K121738	Monitoring	Central 3 Degrees Metamorphopsia (Visual Distortion)	Maculopathy
Proteus Ingestion Confinmation Systems	Proteus Biomedical, Inc.	K113070	Monitoring	Physiological And Behavioral Metrics Including Heart Rate, Activity, Body Angle And Time-Stamped User-Logged Events	General
Rhythmstat Xl	Data Critical Corp.	K971650	Diagnostic	Ecg	Cardiovascular
Sd360 Digital Recorder/Sd360 Holter Digital Recorder	Northeast Monitoring, Inc.	K041901	Monitoring	Heart Beat	Cardiovascular
Silhouette, Model 1000.01	Aranz Medical Limited	K070426	Monitoring	External Wounds	External Wounds
Smartheart	Shl Telemedicine International Ltd.	K113514	Monitoring	Lead Egg And Rhythm Strip	Cardiovascular
Veo Multigas Monitor For Pocket Pc, Model 400221	Weissburg Associates	K051857	Monitoring	Carbon Dioxide; Oxygen	Anesthesiology
Vestibular Analysis Apparatus	Capacity Sports, Llc	K121590	Monitoring	Balance	
Welldoc Diabetes Manager System And Diabetes Manager Rx System	Welldoc, Inc	K120314	Monitoring	Glucose	Diabetes
Withings Blood Pressure Monitor	Withings	K110872	Monitoring	Blood Pressure	Cardiovascular

**Table 2:** Examples of physiological biomarkers. (For a complete list of physiological biomarkers, please refer to Supplementary Table S2).

**Biomarker**	**Biomarker Type**	**Detected Disease**	**Disease ICD Code**	**Clinical status**
Amygdala volume	Prognostic	Parkinson’s disease	G20, F02.3	
Ankle brachial index (ABI)	Diagnostic	Peripheral arterial disease	I73	Used in clinic
Anterior temporal atrophy	Diagnostic	Frontotemporal lobar degeneration	G31.0	
Carotid intima-media thickness (CIMT)	Diagnostic	Coronary disease	I25.1	
Early hypertension	Theragnostic	Pancreatic cancer	C25	Clinical trial
EBC pH	Diagnostic	Asthma	J45	
Electrocardiography (ECG)	Prognostic	Acute coronary syndrome	I20.0	
Hair morphology	Prognostic; Theragnostic	Mucopolysaccharidoses	E76	
Hippocampal volume	Prognostic	Parkinson’s disease	G20, F02.3	
Longitudinal MRI volumetric data	Prognostic	Alzheimer’s disease	G30, F00	Used in clinic
Macrophage migration inhibitory factor (MIF)	Diagnostic	Bronchopulmonary dysplasia	P27.1	
Mammographic density	Diagnostic	Breast cancer	C50	Clinical trial
Mean width of frontal horns of lateral ventricles	Prognostic	Parkinson’s disease	G20, F02.3	
Mean width of third ventricle	Prognostic	Parkinson’s disease	G20, F02.3	
Motor unit number estimation	Monitoring	Amyotrophic lateral sclerosis	G12.2	
Neurophysiological index	Monitoring	Amyotrophic lateral sclerosis	G12.2	
Sclerosis	Prognostic	Follicular lymphoma	C82	Clinical trial
Single-fiber electromyography (SFEMG)	Prognostic	Myasthenia gravis	G70.0	
Sputum cytology	Diagnostic	Lung carcinoma	C33-C34	
Total kidney volume (TKV)	Prognostic	Autosomal-Dominant Polycystic Kidney Disease	Q61	
Unilateral area of substantia nigra hyperechogenicity	Prognostic	Parkinson’s disease	G20, F02.3	
Urine osmolality	Prognostic	Autosomal-Dominant Polycystic Kidney Disease	Q61	
Voxel-based morphometry	Diagnostic	Amyotrophic lateral sclerosis	G12.2	

**Table 3:** Examples of non-invasive molecular biomarkers. For a complete list of non-invasive molecular biomarkers, please refer to Supplementary Table S3.

**Biomarker**	**Detected Disease (ICD code)**	**Type**	**S**	**Detection Sen**	**Detection Spe**	**Biomarker**	**Detected Disease (ICD code)**	**Type**	**S**	**Detection Sen**	**Detection Spe**
17-urine-peptide biomarker panel	M00-M25	Diag	U	~85%	~100%	MEP1A, meprin A	M30.3	Diag	U	~93%	~94%
2-aminoacetophenone	E84	Diag	Br	0.938	0.692	Methylhistamine; interleukin-6	N30.10, N30.11	Diag	U	0.7	0.724
8-hydroxy-2-deoxyguanosine (8-OHdG)	P27.1	Diag	U	0.857	0.611	Monoclonal free immunoglobulin light chains	E85.8	Diag	U	0.813	0.98
ABCA5	D07.5	Diag	U	~100%	N/A	Monocyte chemotactic protein-1 (MCP-1)	Q62.0	Diag	U	~85.0%	~90.0%
Basic fibroblast growth factor	C56	Diag	U	0.7	0.75	N-Acetyl-β-D-glucosamindase (NAG)	N02.2	Prog	U	0.77	N/A
Beta2-microglobulin	N15.0	Diag	U	0.723	0.844	Neutrophil gelatinase-associated lipocalin (NGAL)	M32	Prog	U	~70%	~89%
Calprotectin	K50,K51	Prog	F	0.9	0.83		N14.1	Prog	U	0.8	0.75
DPD	C90.0	Diag	U	0.889	0.833		B20	Moni; Ther	U	0.94	0.71
EL, endothelial lipase protein	C16	Diag	U	0.79	1		N17	Diag	U	1	0.98
Eosinophils	J45	Diag	Sp	0.86	0.88		N14.1	Diag	U	0.73	1
Fibrinopeptide B	I82.4,I82.5	Diag	U	1	0.85	NGF	N30.10, N30.11	Diag	U	0.75	0.655
Fibulin-3	M15-M19,M47	Diag	U	0.746	0.857	Orosomucoid	O11,O14	Prog	U	~56.0%	~73.0%
HLA-DR	T86.1	Diag	U	0.8	0.98	Podocalyxin (PODXL)	C64	Diag	U	1	1
IL-18	N17	Prog	U	>90%	>90%	Pyruvate kinase isoenzyme M2-PK	C18-C21	Diag	F	73–83%	0.82
IL-8	F40-F42	Diag	U	~100%	N/A	S100A12	K50,K51	Diag	F	0.86	0.96
	N21.0-N21.9	Diag; moni	U	0.9	0.68	S100B protein	S06	Prog	U	0.9	0.628
					S100B; lactate/creatinine ratio	G93.4	Diag	U	0.99	0.97	
Kininogen	B55.0	Diag	U	0.9		Tim-3	T86.1	Prog	U	84–87%	95–96%
Lactoferrin	K50,K51	Moni	F	70–100%	44–100%	Trypsinogen	K85	Diag	U	1	0.96
Leucine-rich alpha-2-glycoprotein (LRG)	K35-K37	Diag	U	0.95	1	Trypsinogen activation peptide (TAP)	K85	Prog	U	0.917	0.897
Liver-type fatty acid-binding protein(L-FABP)	N03.2	Prog; Moni	U	0.875	0.905	Trypsinogen-2	K85, K86.0-K86.1	Diag	U	0.81	0.97
Matrix metalloproteinase 9 (MMP 9)	H16.229	Diag; Moni	T	0.85	0.94	Uromodulin	N02.8	Diag	U	1	1
	N13.7	Diag;Prog	U	0.812	0.85						

(Diag: Diagnostic, Prog: Prognotic, Mon, Monitoring, Br: Breath, F: Feces, Sa: Saliva: Sk: Skin, Sp: Sputum, T: Tears, U: Urine, Sen: Sensitivity. Spe: Specificity).

**Table 4:** New biomarker detection technologies.

**Information about the Biomarker used for Testing the Detection Technology**						**Information about the Biomarker Detection Technology**							
**Biomarker**	**Biomarker molecule type**	**Biomarker Source**	**Detected Disease Condition (Detection Type)**	**Biomarker Levels in Patients**	**Biomarker Levels in Normal Population**	**Biomarker Detection Technology**	**Product Cost**	**Lower Limit of Detection**	**Upper Limit of Quantification**	**Minimum Sample Volume**	**Detection Time**	**Technology Readiness for Detecting Biomarker in Non-invasive Source from Patients**	**Reference**
Paper-based and mobile-phone enabled technologies													
Human epididymis protein 4 (HE4)	Protein	Urine	Ovarian cancer (D)	364.5 ng/mL - 458.8 mg/mL	0.0574 ng/mL - 727.1 ug/mL	Paper-based ELISA + smartphone	N/A	19.5 ng/mL	1250 ng/mL	100 μL	5 h (may be cut to 15 min)	Within range	9
Mycobacterium tuberculosis nucleic acids	DNA	N/A	Tuberculosis (D)	N/A	N/A	Paper-based Au-nanoprobes + smartphone	N/A	10 μg/mL	N/A	5 μL	65 min (2h30min including PCR amplification)	N/A	12
MMP9	Protein	Urine	Colorectal cancer (D)	N/A	N/A	Paper lateral flow assay + smartphone/scanner	$2.60 + cost of cellphone	92 ng/mL	644 ng/mL	5 μL	N/A	N/A	15
Thrombin	Protein	Urine	Thrombosis (D)	N/A	N/A	Paper lateral flow assay + smartphone/scanner	$2.60 + cost of cellphone	72 ng/mL	504 ng/mL	5 μL	N/A	N/A	15
Neuropeptide Y	Peptide	Saliva	Post-traumatic stress disorder (P, T)	∼1.7–5.95 pg/mL(plasma)	0.014–0.065 pg/mL (saliva), ∼0.21–2.42 pg/mL (plasma)	Paper-Based ELISA + camera/smartphone/scanner/printer	Low cost	127.59 ng/mL	21.265 μg/mL	3 μL	<60 min	Out of range	22
Hepatitis B virus plasmid DNA	DNA	N/A	Hepatitis B (D)	N/A	N/A	Convective polymerase chain reaction + smartphone	N/A	30 copies per reaction	N/A	3 μL	20 min	N/A	48
VEGF	Protein	Inner eye aqueous humor	Proliferative diabetic retinopathy, age-related macular degeneration, retinal vein occlusion (D)	740.1 ± 267.7 pg/mL, 383 ± 155.5 pg/mL, 219.4 ± 92.1 pg/mL	14.4 ± 8.5 pg/mL	Paper-based ELISA + Smartphone	Cost of paper-ELISA + cost of cellphone	33.7 fg/mL	10 μg/mL	2 μL	44 min	Within range	24
Paper-based technologies													
Chorionic gonadotropin	Protein	Urine	Pregnancy (D)	>2.5 ng/mL	<0.5 ng/mL	Automated paper-based sequential multistep ELISA. + inkjet printing	Low cost	0.81 ng/mL	500 ng/mL	50 μL	15–25 min	Within range	49
HIV-1 envelope antigen gp41	Protein	Serum	HIV infection (P)	N/A	N/A	Paper-based ELISA + scanner	Cost of paper-ELISA + $100 for scanner	N/A	N/A	<20 μL	<60 min	N/A	25
Anti-Leishmania antibodies	Protein	Canine blood	Leishmaniasis (D)	N/A	N/A	Paper-based ELISA + scanner	Cost of paper-ELISA + $100 for scanner	1 mg/mL	N/A	μL range	60 min	N/A	12
Anti-NC16A autoimmune antibodies	Protein	Blister fluid	Bullous pemphigoid (D)	N/A	N/A	Paper-Based ELISA + desktop scanner	Cost of paper-ELISA + $100 for scanner	3 ug/mL	50 μg/mL	2 μL	70 min	N/A	50
Lactoferrin	Protein	Tear	Dry eye syndrome (D)	0.13 ± 0.22 mg/mL	2.05 ± 1.12 mg/mL	An inkjet-printed microfuidic paper-based analytical device + digital camera	$0.0131 per testing sheet + cost of digital camera	5 ng/mL	50 ng/mL	2.5 μL	15 min	Within range after dilution	13,51
Mobile-phone enabled technologies													
Plasmodium falciparum histidine-rich protein 2 (PfHRP2)	Protein	Serum, Saliva	Malaria (D)	17–1167 pg/mL (saliva)	0	A disposable microfluidic chip + smartphone with embedded circuit	N/A	16 ng/mL	1024 ng/mL	0.5 μL	15 min	Out of range	26,52
Bacterial DNA	DNA	N/A	Bacterial infection (D)	N/A	N/A	A disposable micro?uidic chip with primers + a fluorescence detector + smartphone	$350-$600	760 DNA copies per μL	N/A	30 μL	30 min	N/A	33
Interferon-gamma	Protein	N/A	Latent tuberculosis (D)	48.69 ± 28.78 pg/ml (blood)	12.99 ± 5.70 pg/ml (blood)	An opto-acoustic immunoassay + mobile phone technologies ( surface acoustic wave transducer, CMOS camera, LED)	low cost	17.15 pg/mL	17.15 ng/mL	N/A	10 min	Within range	27,53
Adenovirus DNA	DNA	N/A	Viral infection	N/A	N/A	A microfluidic capillary array + an optical signal amplifier (multi-wavelength LEDs) + smartphone	$180 for capillary array + cost of LED and smartphone	0.4 ug/mL	5 μg/mL	10 μL	N/A	N/A	28
Cortisol	Small molecule	Saliva	Stress, anxiety, depression (D)	20.7–37.3 ng/mL	0.4–14.1 ng/mL	Chemiluminescent lateral flow Immunoassay + smartphone with custom-designed 3D printer	Low cost	0.3 ng/mL	60 ng/mL	25 μL	30 min	Within range	54,55
N-terminal proBNP molecule	Peptide	Blood	Heart failure (D,P)	1076 ± 138 pg/mL	38 ± 4 pg/mL	A disposable biomarker sensing element + HDR image acquisition technique + computer screen photo-assisted technique + smartphone	N/A	60 pg/mL	3000 pg/mL	150 μL	12 min	Within range	30,56
IL-6	Protein	Serum	Cancer (P)	300- 3500 pg/mL	<300 pg/mL	ELISA + smartphone	N/A	2 pg/mL	N/A	N/A	2 hour 40 min	Within range	57
Albumin	Protein	Urine	Kidney disease (D)	>30–300 ug/mL	<30 ug/mL	Fluorescent assay in disposable test tubes + smartphone	$190 + cost of phone	5–10 μg/mL	200 μg/mL	25 μL	5 min	Within range	26
Other lab-on-a-chip platform technologies													
Apolipoprotein A1	Protein	Urine	Bladder cancer (D)	207.3 -3754.7 ng/mL	~ 10 ± 8 ng/mL	A negative-pressure-driven microfluidic chip magnetic bead based ELISA + optical measurment device	lower costs than conventional ELISA	10 ng/mL	2000 ng/ml	14.5 μL	40 min	Within range	31,32
Minimally invasive finger-prick biomarker technologies													
C-reactive protein	Protein	Blood	Prostate cancer, colorectal cancer (P),	>3 ug/mL (blood)	<1 ug/mL (blood)	A microtiterplate based ELISA + smartphone	<$660	0.3 ng/mL	81 ng/mL	N/A	<30 min	Within range after dilution	29,58
HIV-1 gp41 and HIV-2 gp36	Protein	Blood	HIV infection (P)	N/A	N/A	A low-power, low-cost and compact smartphone dongle of microfluidic ELISA	$34 + + cost of cellphone	10 μg/mL	N/A	2 μL	15 min	N/A	59,60
N-terminal proBNP molecule	Peptide	Blood	heart failure (D,P)	1076 +_ 138 pg/mL	38 +_ 4 pg/mL	A disposable biomarker sensing element + HDR image acquisition technique + computer screen photo-assisted technique + smartphone	N/A	60 pg/mL	3000 pg/mL	150 uL	12 min	Within range	30,56
Antibodies against HIV	Protein	Blood	HIV (D)	>0	0	A mobile microfluidic chip for immunoassay	$0.1 per cassette + $0.5 light-emitting diodes+ $6 photodetector + cell phone	N/A	N/A	1 uL	20 min	Within range	39
Antibodies against Treponema pallidum	Protein	Blood	syphilis (D)	>0	0	A mobile microfluidic chip for immunoassay	$0.1 per cassette + $0.5 light-emitting diodes+ $6 photodetector + cell phone	N/A	N/A	1 uL	20 min	Within range	39
Prostate-specific antigen (PSA)	Protein	Blood	Prostate cancer (D)	>4 ng/mL	<4 ng/mL	A microfluidic purification step + label-free nanosensor detection	low cost	1.5 ng/mL	N/A	10 uL	20min	Within range	40
Carbohydrate antigen 15.3 (CA15.3)	Protein	Blood	Breast cancer (D)	>30 U/ml	<30 U/ml	A microfluidic purification step + label-free nanosensor detection	low cost	15 U/mL	N/A	10 uL	20min	Within range	40
Haemoglobin	Protein	Blood	Anaemia (D)	N/A	N/A								38
Aspartate aminotransferase (AST)	Protein	Blood	Tuberculosis/HIV (T)	N/A	5−40 U/L	A paper-based, multiplexed microfluidic assay	<$0.10 per test	84 U/L	N/A	15 uL	15 min	Within range	42
Alkaline phosphatase (ALP)	Protein	Blood	Tuberculosis/HIV (T)	N/A	30−120 U/L	A paper-based, multiplexed microfluidic assay	<$0.10 per test	53 U/L	N/A	15 uL	15 min	Within range	42
Aspartate aminotransferase (AST)	Protein	Blood	Hepatitis (D)	Acute : ~400 U/L, Chronic: ~ 160 U/L	5−40 U/L	A micropatterned paper-based microfluidic device + cellphone	low cost	44 U/L	400 U/L	15 uL	15 min	Within range	35
Alkaline phosphatase (ALP)	Protein	Blood	Liver conditions (D)	N/A	30−120 U/L	A micropatterned paper-based microfluidic device + cellphone	low cost	15 U/L	400 U/L	15 uL	15 min	Within range	35

**Table 5:** Examples of common diseases covered by non-invasive molecular biomarkers. For a complete list, please refer to Supplementary Table S5.

**Disease or Disease Class**	**Disease ICD Code**	**Disease Prevalence**	**Biomarker Function Type**	**Biomarker Molecular Type (No of Biomarkers, No in clinical use or trial)**	**Biomarker Source**	**Feasibility of New Tech Based Biomarker Detection**	**Highest Biomarker Detection Sensitivity**	**Highest Biomarker Detection Specificity**	**Disease Form (Acute/ Chronic)**	**Biomarker Level in Patients**	**Biomarker Level in Normal Population**	**Technology Readiness for Detecting Biomarkers from Non-Invasive Sources from Patients**
HIV infection	B20	World (35.3 M),USA (1.15 M),UK (2.2 M)	Prog	P (6)	U	ELISA	94.00%	71.00%	A/C	N/A	N/A	N/A
			Ther	P (6)	U	ELISA	94.00%	71.00%	A/C	N/A	0.2–146.7 ng/mL	Within range
Diabetic Nephropathy	E10.2, E11.2, E12.2, E13.2, E14.2	P:World (20% - 40% of diabetes)	Diag	P (7)	U	ELISA	81.40%	62.50%	C	27.3 ± 3.3 ng/μmol	0–25 ng/mg	Within range
			Prog	P (3)	U	ELISA	N/A	N/A	C	N/A	N/A	N/A
Type 2 diabetes	E11	P:World (), USA (27.85M), Europe ()	Diag	P (11)	U	ELISA	~91%	~78%	C	56.9 ± 19.45 μg/mL	9.7 ±2.35 μg/mL	Within range
			Prog	P (3)	U	ELISA	N/A	N/A	C	N/A	N/A	N/A
Chronic stress	F40-F42	P:World (40 M)	Diag	P (1, CT)	U	ELISA	100.00%	N/A	C	70.9 ± 19.2 pg/mg	18.8 ± 32 pg/mg	Out of range
Parkinson’s disease	G20	P:World (10 M),USA (1 M),UK (6.7 M)	Prog	Sm (1)	U		N/A	N/A	C	N/A	N/A	N/A
Asthma	J45	P:World (235 M),USA (25 M),UK (30 M)	Diag	Sm (4), P (1), Cell (2)	Br, Sp	ELISA	73.6–86.0%	88.00%	C	N/A	N/A	N/A
			Prog	Sm (2), P (1) Sm+P (1, CT), Cell (1), Sm+Cell (1)	Br, Sp	ELISA	N/A	N/A	C	N/A	N/A	N/A
Acute appendicitis	K35-K37	I:USA (680,000 per year)	Diag	P (9)	U		95.00%	100.00%	A	0.9–19.3 μg/mL	0.1–0.8 μg/mL	Within range
Inflammatory Bowel Disease	K50,K51	P:World (0.396% population),USA (1.4 M),UK (2.5–3 M)	Diag	P (12, CU 2), Sm (1)	Br, F	ELISA	80–98%, 94%	82–96%, 76%	C	2.45 ± 1.15 ng/mg	0.006 ± 0.03 ng/mg	N/A
			Prog	P (16, CU 2)	F	ELISA	80–90%, 70–100%	82–83%, 44–100%	C	N/A	8–213 μg/mg	N/A
			Ther	P (2)	F	ELISA	N/A	N/A	C	N/A	N/A	N/A
Psoriasis	L40	P:World (125 M),USA (7.5 M),UK (11 M)	Diag	P (2), miR (4), cell (1)	Sk	ELISA	N/A	N/A	C	N/A	N/A	N/A
Arthritis	M00-M25	P:World (1% of population),USA (52.5 M)	Diag	P (17)	U		~85%	~100%	C	191.7–313.4 ng/mmol	129.25 -486.85 ng/mmol	Within range
			Prog	P (1)	U	ELISA	N/A	N/A	C	N/A	N/A	N/A
Osteoarthritis	M15-M19, M47	P:World (26.9 M)	Diag	P (3), Sm (1), Pep (1), Modified Pep (2, CT 1)	U	ELISA	74.60%	85.70%	C	191.4 pM	144.4 pM	Almost within range
			Prog	Sm (1), Pep (3), Modified Pep (2)	U		N/A	N/A	C	N/A	N/A	N/A
Acute kidney injury	N17	P:USA (1–7.1% of all hospital admissions)	Diag	P (15, CU 2, CT 3)	U	ELISA	69–100%, 73–100%	85–98%	A	50.5–205.9 ng/mL	5.7–17.7 ng/mL	Within range
			Prog	P (2, CT 1)	U	ELISA	>90%	>90%	A	0–955 pg/mL	0–173 pg/mL	Out of range
Urolithiasis	N21.0-N21.9	P:USA (7% of women and 12% of men)	Diag	P (3)	U	ELISA	90.00%	68.00%	C	104.66 ± 159.70 pg/mg	7.76 ± 8.90 pg/mg	Out of range
			Prog	P (1)	U	ELISA	N/A	N/A	C	104.66 ± 159.70 pg/mg	7.76 ± 8.90 pg/mg	Out of range
Interstitial cystitis	N30.10, N30.11	P:USA (8 million women)	Diag	P (7), Sm (2)	U	ELISA	70.00%	72.40%	C	0.25 ± 0.1 pg/mg	0.9 ± 0.4 pg/mg	Out of range
Pre-eclampsia	O11,O14	P:USA (3–4% baby-delivery women)	Diag	P (9)	U	ELISA	N/A	N/A	A	2.11 mg/mL	0.014 mg/mL	Within range after dilution
			Prog	P (4)	U	ELISA	~56%	~73%	A	N/A	N/A	N/A
Traumatic brain injury (TBI)	S06	P:USA (823.7 in 100,000)	Prog	P (1)	U	ELISA	90.00%	62.80%	A/C	0.025 ng/mL	0.02–1.35 ng/mL	Out of range

(Diag, Prog, Br, F, Sa, Sk, Sp, T, U are the same as in Table 3,Ther: Theragnostic, P: Protein, Sm: Small molecule, Pep: Peptide, miR: microRNA, CU: Clinical use, CT: Clinical trial, combi: combination, A: acute, C:Chronic).
