# Supplementary material for: The Assessment of the Readiness of Molecular Biomarker-Based Mobile Health Technologies for Healthcare Applications
Source: Sci Rep. 2015 Dec 8;5:17854. doi: 10.1038/srep17854 (PMC4672303; doi:10.1038/srep17854)
Supplement: Supplementary Table S1 [file srep17854-s1.doc]

Supplementary Table S1: FDA endorsed mobile apps

| **Device name** | **Applicant** | **510(k) number** | **Type** | **Measure** | **Disease** |
| --- | --- | --- | --- | --- | --- |
| Aidera diasend system | Aidera ab | K101806 | Data transmitter | N/A | N/A |
| Airstrip ob | Mp4 solutions, lp | K042082 | Monitoring | fetal heart tracings; maternal contraction pattern | Obstetrics/gynecology |
| Airstrip ob | Airstrip technologies, lp | K090061 | Monitoring | fetal heart tracings; maternal contraction pattern | Obstetrics/gynecology |
| Airstrip ob | Airstrip technologies, lp | K090269 | Monitoring | fetal heart tracings; maternal contraction pattern | Obstetrics/gynecology |
| Airstrip remote patient monitoring (rpm) | Airstrip technologies, lp | K110503 | Data viewer | N/A | N/A |
| Airstrip remote patient monitoring (rpm) remote data viewing | Airstrip technologies, lp | K112235 | Data viewer | N/A | N/A |
| Airstrip remote patient monitoring (rpm) remote data viewing | Airstrip technologies, lp | K121871 | Data viewer | N/A | N/A |
| Airstrip remote patient monitoring (rpm) remote data viewing software, version 3.1 | Airstrip technologies, lp | K100133 | Data viewer | N/A | N/A |
| Alivecor heart monitor for iphone | Alivecor, inc. | K122356 | Monitoring | Ecg | Cardiovascular |
| Asthmapolis system | Reciprocal labs corporation | K121609 | Medical aid | Actuations of prescribed mdi usage | Anesthesiology |
| Avita bluetooth blood pressure monitor, model: bpm656zb | Avita corporation | K072137 | Monitoring | Systolic and diastolic blood pressure; pulse rate | N/A |
| Aycan mobile | Aycan digitalsysteme gmbh | K122260 | Data viewer | Medical images for diagnosis from ct and mri | N/A |
| Beam brush/beam app | Beam technologies, llc | K121165 | Monitoring | Brushing usage data | Tooth decay |
| Bioharness | Zephyr technology corporation | K113045 | Monitoring | Ecg | Cardiovascular |
| Bodyguardian system bodyguardian control unit bodyguardian connect | Preventice, inc. | K121197 | Monitoring | Ecg;activity;heart rate; respiration rate | Cardiovascular |
| Carestream pacs | Carestream health, inc. | K110919 | Data viewer | 3d image | Radiology |
| Cg-5108 act-3l continuous ecg monitor and arrhythmia detector | Card guard scientific survival ltd. | K110499 | Monitoring | Ecg | Cardiovascular |
| Cg-6108 act-3l continuous ecg monitor & arrhythmia detector | Card guard scientific survival, ltd. | K081257 | Monitoring | Ecg | Cardiovascular |
| Cg-6108 act-il continuous ecg monitor and arrythmia detector | Card guard scientific survival, ltd. | K101639 | Monitoring | Ecg | Cardiovascular |
| Cg-6108 arrhythmia ecg event recorder | Card guard scientific survival, ltd. | K060911 | Monitoring | Ecg | Cardiac arrhythmia |
| Cg-6108 continuous ecg monitor and arrhythmia detector | Card guard scientific survival, ltd. | K071995 | Monitoring | Ecg | N/A |
| Confidant 2.5 | Confidant inc. | K072698 | Data transmitter | N/A | N/A |
| Customized sound therapy (cst) | Tinnitus otosound products, llc | K070599 | Treatment | N/A | Tinnitus |
| Dash knee | Brainlab ag | K102251 | Medical aid | N/A | N/A |
| Datex-ohmeda s/5 web viewer, datex-ohmeda s/5 pocket viewer and datex-ohmeda s/5 cellular viewer with l-web04 software | Ge healthcare | K052975 | Data viewer | Real-time patient information | N/A |
| Diabetesmanager system, diabetesmanager-rx system model version 1.1 | Welldoc, inc | K100066 | Monitoring | Glucose | Diabetes |
| Freestyle tracker diabetes management system | Abbott diabetes care inc. | K020866 | Monitoring | Glucose | Diabetes |
| Freestyle tracker diabetes management system | Abbott diabetes care inc. | K020866 | Monitoring | Glucose | Diabetes |
| Fully automatic electronic blood pressure monitor model kd-931 | Andon health co.,ltd | K102939 | Monitoring | Blood pressure | Cardiovascular |
| Fully automatic wireless blood pressure wrist monitor | Andon health co., ltd | K121470 | Monitoring | Blood pressure | Cardiovascular |
| Glucophone blood glucose test system, model igm-0025 | Infopia co., ltd | K091168 | Monitoring | Glucose | Diabetes |
| Ibgstar blood glucose monitoring system, ibgstar diabetes manager application, rev d | Agamatrix inc | K103544 | Monitoring | Glucose | Diabetes |
| Iglucose system | Positiveid corporation | K111932 | Monitoring | Glucose | Diabetes |
| Imco-stat | Imco technologies | K063392 | Data viewer | N/A | N/A |
| Intuition | Terarecon, inc. | K121916 | Data viewer | Ebt, ct, pet or mri image | N/A |
| Kd-936 fully automatic wireless blood pressure monitor | Andon health co.,ltd | K120672 | Monitoring | Blood pressure | Cardivascular |
| Medapps remote patient monitoring, model ma 100 | Medapps, inc. | K062377 | Data transmitter | N/A | N/A |
| Medicalgorithmics real-time ecg monitor and arrhythmia detector, model pocketecg | Medicalgorithmics sp z.o.o. | K090037 | Monitoring | Heart beat, rhythm abnormalities | Cardivascular |
| Mobile mim | Mim software inc. | K103785 | Data viewer | Spect, pet, ct, and mri | N/A |
| Mobile mim | Mim software inc. | K112930 | Data viewer | Spect, pet, ct, mri, x-ray and ultrasound | N/A |
| Mobilect viewer | Nephosity, inc. | K123082 | Data viewer | Ct, mri, x-ray images | N/A |
| Mobile-patient viewer | Data critical corporation | K011436 | Data viewer | N/A | N/A |
| Mobius ultrasound imaging system | Mobisante, inc. | K102153 | Imaging | N/A | N/A |
| Modification to: cg-6108 act-3l continuous ecg monitor and arrhythmia detector, model fg-00084 | Card guard scientific survival, ltd. | K101703 | Monitoring | Ecg | Cardiovascular |
| Modification to: pocketview ecg software | Micromedical industries, ltd. | K013311 | Data viewer | Ecg | Cardiovascular |
| Myglucohealth glucose monitoring systems | Entra health systems, ltd. | K081703 | Monitoring | Glucose | Diabetes |
| Myvisiontrack(tm) | Vital art and science incorporated | K121738 | Monitoring | Central 3 degrees metamorphopsia (visual distortion) | Maculopathy |
| Orthosize | Orthosize llc | K120115 | Medical aid | N/A | Preoperative planning of orthopedic surgery |
| Panoptic | Welch allyn, inc. | K121405 | Imaging | N/A | N/A |
| Pill phone | Vocel | K060298 | Medical aid drug compliance | N/A | N/A |
| Pixel app | Gauss surgical, inc. | K120473 | Medical aid | N/A | Surgery |
| Pixel app | Gauss surgical, inc. | K121274 | Medical aid | N/A | Surgery |
| Proteus ingestion confinmation systems | Proteus biomedical, inc. | K113070 | Monitoring | Physiological and behavioral metrics including heart rate, activity, body angle and time-stamped user-logged events | General |
| Reka e100 | Reka pte ltd | K111438 | Monitoring | Ecg | Cardiovascular |
| Resolutionmd mobile 3.1 model rmd-mob-31 | Calgary scientific, inc. | K123186 | Data viewer | Ct and mr medical images | N/A |
| Resolutionmd mobile model rmb-mob-2x | Calgary scientific, inc. | K111346 | Data viewer | Ct and mr medical images | N/A |
| Rhythmstat xl | Data critical corp. | K971650 | Diagnostic | Ecg | Cardiovascular |
| Sd360 digital recorder/sd360 holter digital recorder | Northeast monitoring, inc. | K041901 | Monitoring | Heart beat | Cardiovascular |
| Silhouette, model 1000.01 | Aranz medical limited | K070426 | Monitoring | External wounds | External wounds |
| Smartheart | Shl telemedicine international ltd. | K113514 | Monitoring | Lead egg and rhythm strip | Cardiovascular |
| Spectrum and spectrum with master drug library | Sigma intl. | K042121 | Medical aid administration | N/A | N/A |
| Surgicase connect | Materialise n.v. | K113599 | Data transmitter | Ct and mr medical images | Cardiovascular |
| Symcare diabetes management program | Symcare personalized health solutions, inc | K083263 | Data transmitter | Glucose | Diabetes |
| Tm2005 personal medical phone center | Card guard scientific survival, ltd. | K024365 | Data viewer | Ecg, and other patient related data, (such as demographics, doctors, medical history and status, diagnoses, etc.) . | Cardiovascular |
| Veo multigas monitor for pocket pc, model 400221 | Weissburg associates | K051857 | Monitoring | Carbon dioxide; oxygen | Anesthesiology |
| Vestibular analysis apparatus | Capacity sports, llc | K121590 | Monitoring | Balance | N/A |
| Wavesense diabetes manager model version 1.3.4 | Agamatrix | K101597 | Data transmitter | Glucose | Diabetes |
| Web viewer, pocket viewer and cellular viewer with l- web05 software | Ge healthcare | K061994 | Data viewer | N/A | N/A |
| Welldoc diabetes manager system and diabetes manager rx system | Welldoc, inc | K112370 | Monitoring | Glucose | Diabetes |
| Welldoc diabetes manager system and diabetes manager rx system | Welldoc, inc | K120314 | Monitoring | Glucose | Diabetes |
| Withings blood pressure monitor | Withings | K110872 | Monitoring | Blood pressure | Cardiovascular |
| Withings, smart body scale | Zhongshan transtek electronics co., ltd. | K121971 | Monitoring | Weight, bmi, body fat | N/A |
