# Supplementary material for: The Assessment of the Readiness of Molecular Biomarker-Based Mobile Health Technologies for Healthcare Applications
Source: Sci Rep. 2015 Dec 8;5:17854. doi: 10.1038/srep17854 (PMC4672303; doi:10.1038/srep17854)
Supplement: Supplementary Table S2 [file srep17854-s2.doc]

Supplementary Table S2: Physiological biomarker

| **Biomarker** | **Type** | **Disease** | **ICD** | **Clinical status** | **Reference** |
| --- | --- | --- | --- | --- | --- |
| Early hypertension (HTN) | theragnostic | Pancreatic cancer | C25 | clinical trial | A332 |
| patient age | prognostic | Non-small cell lung cancer | C33,C34 |  | A301 |
| 18F-fluorodeoxyglucose (FDG) | diagnostic | Lung carcinoma | C33-C34 |  | A596 |
| Sputum cytology | diagnostic | Lung carcinoma | C33-C34 |  | A165 |
| PET/TC | diagnostic | Breast cancer | C50 |  | A642 |
| mammographic density | diagnostic | Breast cancer | C50 | clinical trial | A151 |
| age | prognostic | Breast cancer | C50 | clinical trial | A62 |
| percent S phase | prognostic | Breast cancer | C50 |  | A35 |
| time to progression (TTP) | prognostic | Breast cancer | C50 | clinical trial | A175 |
| IAUC(90) | theragnostic | Renal carcinoma | C64 | clinical trial | 18824708 |
| K(trans) | theragnostic | Renal carcinoma | C64 | clinical trial | 18824708 |
| sclerosis | prognostic | Follicular lymphoma | C82 | clinical trial | 17664481 |
| molecular grade index (MGI) | prognostic | Ductal carcinoma in situ (DCIS) | D05.9 |  | A541 |
| Brain MRI images | prognostic | Mucopolysaccharidoses | E76 |  | 22658917 |
| Hair morphology | prognostic; theragnostic | Mucopolysaccharidoses | E76 |  | 22658917 |
| Brain MRI images | prognostic; theragnostic | Mucopolysaccharidoses | E76 |  | 22658917 |
| extrapyramidal signs | theragnostic | Schizophrenia | F20 |  | 23129338 |
| impairments in cognitive tests | theragnostic | Schizophrenia | F20 |  | 23129338 |
| sedation | theragnostic | Schizophrenia | F20 |  | 23129338 |
| Magnetic resonance spectroscopy | diagnostic | Amyotrophic lateral sclerosis | G12.2 |  | 21989244 |
| Voxel-based morphometry | diagnostic | Amyotrophic lateral sclerosis | G12.2 |  | 21989244 |
| Diffusion tensor imaging (DTI) | monitoring | Amyotrophic lateral sclerosis | G12.2 |  | 21989244 |
| Electrical impedance myography | monitoring | Amyotrophic lateral sclerosis | G12.2 |  | 21989244 |
| Motor unit number estimation | monitoring | Amyotrophic lateral sclerosis | G12.2 |  | 21989244 |
| Neurophysiological index | monitoring | Amyotrophic lateral sclerosis | G12.2 |  | 21989244 |
| FDG network | diagnostic | Parkinson's disease | G20, F02.3 |  | 22982303 |
| MRI (DWI) | diagnostic | Parkinson's disease | G20, F02.3 |  | 22982303 |
| Transcranial sonography (TCS) | diagnostic | Parkinson's disease | G20, F02.3 |  | 22982303 |
| Cardiac autonomic dysfunction | diagnostic | Parkinson's disease | G20, F02.3 |  | 22982303 |
| Amygdala volume | prognostic | Parkinson's disease | G20, F02.3 |  | 23587062 |
| Biceps burst duration variability | prognostic | Parkinson's disease | G20, F02.3 |  | 23587062 |
| Contralateral lateral SN pars compacta R2* | prognostic | Parkinson's disease | G20, F02.3 |  | 23587062 |
| Contralateral phase shift values of SN | prognostic | Parkinson's disease | G20, F02.3 |  | 23587062 |
| Contralateral posterior GP R2* | prognostic | Parkinson's disease | G20, F02.3 |  | 23587062 |
| Contralateral SN R2'' relaxation rate | prognostic | Parkinson's disease | G20, F02.3 |  | 23587062 |
| Delayed H/M ratio of 123I-MIBG uptake | prognostic | Parkinson's disease | G20, F02.3 |  | 23587062 |
| Early H/M ratio of 123I-MIBG uptake | prognostic | Parkinson's disease | G20, F02.3 |  | 23587062 |
| EEG total power | prognostic | Parkinson's disease | G20, F02.3 |  | 23587062 |
| Heart rate variability: low frequency (LF) power spectrum densities | prognostic | Parkinson's disease | G20, F02.3 |  | 23587062 |
| Heart rate variability: very low frequency (VLF) power spectrum densities | prognostic | Parkinson's disease | G20, F02.3 |  | 23587062 |
| Hippocampal volume | prognostic | Parkinson's disease | G20, F02.3 |  | 23587062 |
| Mean amplitude after rare stimuli | prognostic | Parkinson's disease | G20, F02.3 |  | 23587062 |
| Mean area of bilateral substantia nigra hyperechogenicity | prognostic | Parkinson's disease | G20, F02.3 |  | 23587062 |
| Mean intensity score of SN pars compacta | prognostic | Parkinson's disease | G20, F02.3 |  | 23587062 |
| Mean PU R2 | prognostic | Parkinson's disease | G20, F02.3 |  | 23587062 |
| Mean width of frontal horns of lateral ventricles | prognostic | Parkinson's disease | G20, F02.3 |  | 23587062 |
| Mean width of third ventricle | prognostic | Parkinson's disease | G20, F02.3 |  | 23587062 |
| MSNA of peroneal nerve fascicles in right popliteal fossa | prognostic | Parkinson's disease | G20, F02.3 |  | 23587062 |
| P3 amplitude | prognostic | Parkinson's disease | G20, F02.3 |  | 23587062 |
| P3 latency | prognostic | Parkinson's disease | G20, F02.3 |  | 23587062 |
| Percentage of short 1st agonist burst durations | prognostic | Parkinson's disease | G20, F02.3 |  | 23587062 |
| Slope of power law relation (slope of HRV) | prognostic | Parkinson's disease | G20, F02.3 |  | 23587062 |
| SN fractional anisotropy (FA) value | prognostic | Parkinson's disease | G20, F02.3 |  | 23587062 |
| Unilateral area of substantia nigra hyperechogenicity | prognostic | Parkinson's disease | G20, F02.3 |  | 23587062 |
| FDG network | diagnostic | Parkinson's disease | G20, F02.3 |  | 22982303 |
| Transcranial sonography (TCS) | diagnostic | Parkinson's disease | G20, F02.3 | clinical trial | 22982303 |
| Cardiac autonomic dysfunction | diagnostic | Parkinson's disease | G20, F02.3 |  | 22982303 |
| FDG network | monitoring | Parkinson's disease | G20, F02.3 |  | 22982303 |
| MRI (DWI) | monitoring | Parkinson's disease | G20, F02.3 |  | 22982303 |
| Cardiac autonomic dysfunction | monitoring | Parkinson's disease | G20, F02.3 |  | 22982303 |
| 18F-fluorodeoxyglucose (FDG) | prognostic | Parkinson's disease | G20, F02.3 |  | 22814541 |
| Perfusion SPECT | prognostic | Parkinson's disease | G20, F02.3 |  | 22814541 |
| Structural MRI | prognostic | Parkinson's disease | G20, F02.3 |  | 22814541 |
| age | diagnostic | Alzheimer's disease | G30 | clinical trial | PMC3726719 |
| age | prognostic; theragnostic | Alzheimer's disease | G30 | clinical trial | PMC3726719 |
| Arterial spin labeling (ASL) | diagnostic | Alzheimer's disease | G30, F00 |  | 23631871 |
| Diffusion tensor imaging (DTI) | diagnostic | Alzheimer's disease | G30, F00 |  | 23631871 |
| disproportionate atrophy | diagnostic | Alzheimer's disease | G30, F00 |  | 23110863 |
| medial temporal lobe (MTL) | diagnostic | Alzheimer's disease | G30, F00 |  | 23631871 |
| Arterial spin labeling (ASL) | prognostic | Alzheimer's disease | G30, F00 |  | 23631871 |
| longitudinal MRI volumetric data | prognostic | Alzheimer's disease | G30, F00 | used in clinic | 23631871 |
| anterior temporal atrophy | diagnostic | Frontotemporal lobar degeneration | G31.0 |  | 22527778 |
| anterior temporal hypometabolism | diagnostic | Frontotemporal lobar degeneration | G31.0 |  | 22527778 |
| anterior temporal hypoperfusion | diagnostic | Frontotemporal lobar degeneration | G31.0 |  | 22527778 |
| frontal temporal atrophy | diagnostic | Frontotemporal lobar degeneration | G31.0 |  | 22527778 |
| frontal temporal hypoperfusion | diagnostic | Frontotemporal lobar degeneration | G31.0 |  | 22527778 |
| Single-fiber electromyography (SFEMG) | prognostic | Myasthenia gravis | G70.0 |  | 23278584 |
| Natural killer (NK) cell function (NKCC) | diagnostic | Chronic fatigue syndrome/myalgic encephalomyelitis | G93.3 |  | 22732129 |
| proliferative capacity | diagnostic | Dry eye disease | H16.229 |  | 22895048 |
| cytotoxicity | diagnostic | Glaucoma | H40-H42 |  | 22827637 |
| electrocardiography (ECG) | prognostic | Acute coronary syndrome | I20.0 |  | 23331845 |
| Carotid intima-media thickness (CIMT) | diagnostic | Coronary disease | I25.1 |  | 22681965 |
| ankle brachial index (ABI) | diagnostic | Peripheral arterial disease | I73 | used in clinic | 22489720 |
| EBC pH | diagnostic | Asthma | J45 |  | 22796631 |
| knuckle cracking (KC) | diagnostic | Osteoarthritis | M15-M19,M47 |  | 22842200 |
| Postmenopausal women with persistent total hip, femoral neck, or lumbar spine BMD T-scores −1.8 to −4.0, or clinical fracture | theragnostic | Osteoporosis | M15-M19,M47 | clinical trial | PMC2957751 |
| Thymus | diagnostic | Bronchopulmonary dysplasia | P27.1 |  | 23523392 |
| spectrophotometry | diagnostic | Bronchopulmonary dysplasia | P27.1 |  | 23523392 |
| macrophage migration inhibitory factor (MIF) | diagnostic | Bronchopulmonary dysplasia | P27.1 |  | 23523392 |
| urine osmolality | prognostic | Autosomal-Dominant Polycystic Kidney Disease | Q61 |  | 22846584 |
| hypertension (HTN) | prognostic | Autosomal-Dominant Polycystic Kidney Disease | Q61 |  | 22846584 |
| male gender | prognostic | Autosomal-Dominant Polycystic Kidney Disease | Q61 |  | 22846584 |
| total kidney volume (TKV) | prognostic | Autosomal-Dominant Polycystic Kidney Disease | Q61 |  | 22846584 |
| young age | prognostic | Autosomal-Dominant Polycystic Kidney Disease | Q61 |  | 22846584 |
