# Supplementary material for: The Assessment of the Readiness of Molecular Biomarker-Based Mobile Health Technologies for Healthcare Applications
Source: Sci Rep. 2015 Dec 8;5:17854. doi: 10.1038/srep17854 (PMC4672303; doi:10.1038/srep17854)
Supplement: Supplementary Table S3 [file srep17854-s3.doc]

Supplementary table S3: Non-invasive molecular biomarkers. (S: Source. Sen:Sensitivity. Spe: Specificity)

| ICD | Type | S | Protein name | Uniprot ID | Pubmed ID | Sen | Spe | ICD | Type | S | Protein name | Uniprot ID | Pubmed ID | Sen | Spe |
| --- | --- | --- | --- | --- | --- | --- | --- | --- | --- | --- | --- | --- | --- | --- | --- |
| A41.9 | diag | U | IP-10， inducible protein-10 | P02778 | 23539685 |  |  | N02.8 | diag | U | alpha-1-antitrypsin | P01009 | 21595033 |  |  |
| A41.9 | diag | U | IL-8 | P10145 | 23539685 |  |  | N02.8 | diag | U | Angiotensinogen (AGT) | P01019 | 21366514 |  |  |
| A41.9 | diag | U | MCP-1， monocyte chemoattractant protein-1 | P13500 | 23539685 |  |  | N02.8 | diag | U | Kininogen precursor (Alpha-2-thiol proteinase inhibitor) | P01042 | 16372274 |  |  |
| B17.2 | diag | U | alpha-1-microglobulin | P02760 | 19860894 |  |  | N02.8 | diag | U | Hemoglobin epsilon chain | P02100 | 16372274 |  |  |
| B17.2 | diag | U | Serum albumin | P02768 | 19860894 |  |  | N02.8 | diag | U | alpha-1-microglobulin | P02760 | 16372274 |  |  |
| B17.2 | diag | U | zinc alpha glycoprotein | P25311 | 19860894 |  |  | N02.8 | diag | U | Serum albumin precursor | P02768 | 16372274 |  |  |
| B17.2 | diag | U | membrane-associated guanylate kinase 2, ODF2 protein | Q5BJF6 | 19860894 |  |  | N02.8 | diag | U | Serotransferrin precursor | P02787 | 16372274 |  |  |
| B17.2 | diag | U | Prostaglandin D2 synthase 21 kDa (Brain) | Q5SQ09 | 19860894 |  |  | N02.8 | diag | U | Alpha-amylase, pancreatic precursor | P04746 | 16372274 |  |  |
| B17.2 | diag | U | FLJ00133 fragment | Q8TES0 | 19860894 |  |  | N02.8 | diag | U | Insulin receptor precursor | P06213 | 16372274 |  |  |
| B17.2 | diag | U | N2B-Titin Isoform/RasGAP-activating-like protein 1 variant | Q8WZ42 | 19860894 |  |  | N02.8 | diag | U | Bifunctional aminoacyl-tRNA synthetase | P07814 | 16372274 |  |  |
| B17.2 | diag | U | KIAA0216 splice variant 1 | Q92614 | 19860894 |  |  | N02.8 | diag | U | Cathepsin B precursor | P07858 | 16372274 |  |  |
| B20 | moni; ther | U | N-Acetyl-β-D-glucosamindase (NAG) | CID439454 | 22716111 |  |  | N02.8 | diag | U | uromodulin | P07911 | 20552702 | 100.0% | 100.0% |
| B20 | moni; ther | U | Cystatin C | P01034 | 22716111 |  |  | N02.8 | diag | U | Extracellular superoxide dis-mutase [Cu-Zn] precursor | P08294 | 16372274 |  |  |
| B20 | moni; ther | U | Retinol Binding Protein (RBP) | P02753,P10745,P09455,P82980,P50120,Q96R05 | 22716111 |  |  | N02.8 | diag | U | Fructose-bisphosphate aldolase C | P09972 | 16372274 |  |  |
| B20 | moni; ther | U | albumin (ALB) | P43652 | 22716111 |  |  | N02.8 | diag | U | monocyte chemoattractant protein-1 | P13500 | 21034351 |  |  |
| B20 | moni; ther | U | Beta-2-microglobulin (B2M) | P61769 | 22716111 |  |  | N02.8 | diag | U | Myosin heavy chain, skeletal muscle | P13535 | 16372274 |  |  |
| B20 | moni; ther | U | neutrophil gelatinase-associated lipocalin (NGAL) | P80188 | 22716111 | 94.0% | 71.0% | N02.8 | diag | U | HLA class 1 histocompatibility antigen, A-30 alpha chain precursor | P16188 | 16372274 |  |  |
| B20 | moni; ther | U | phosphorus tubular reabsorption (RTP) |  | 22716111 |  |  | N02.8 | diag | U | Eukaryotic translation initiation factor 2 subunit 2 | P20042 | 16372274 |  |  |
| B55.0 | diag | U | kininogen | P01042 | 22562212 | 90.0% |  | N02.8 | diag | U | Tumor necrosis factor, alpha-induced protein 3 | P21580 | 16372274 |  |  |
| C15-C26 | diag | U | S100-A6, S100-A9, S100-P, S100-A12 | P06703, P06702, P25815, P80511 | 21538913 | 86.0% | 80.0% | N02.8 | diag | U | Multifunctional protein ADE2 | P22234 | 16372274 |  |  |
| C16 | diag | U | EL, endothelial lipase protein | Q9Y5X9 | 23510199 | 79.0% | 100.0% | N02.8 | diag | U | Ig heavy chain V-I region V35 precursor | P23083 | 16372274 |  |  |
| C18-C21 | diag | U | 1-N-12-N-di-acetyl spermine (DiAcSpm) | CID132680 | 324 | >60% |  | N02.8 | diag | U | Zinc-alpha-2-glycoprotein precursor | P25311 | 16372274 |  |  |
| C18-C21 | diag | F | a special isoenzyme of pyruvate kinase (M2-PK) | P14618 | 311 | 73-83% | 82.0% | N02.8 | diag | U | Mitogen-activated protein kinase 3 | P27361 | 16372274 |  |  |
| C22.0 | diag | U | Heat Shock Protein 60 (HSP60) | P10809 | 23074380 | 42.0% | 83.0% | N02.8 | diag | U | T-complex protein 1, theta subunit | P50990 | 16372274 |  |  |
| C22.0 | diag | U | Chromatin Assembly Factor-1 (CAF-1) | Q13111 | 23074380 | 58.0% | 82.0% | N02.8 | diag | U | Collagen alpha 4(IV) chain precursor | P53420 | 16372274 |  |  |
| C22.0 | diag | U | Chromatin Assembly Factor-1 (CAF-1); Heat Shock Protein 60 (HSP60) | Q13111; P10809 | 23074380 | 61.0% | 92.0% | N02.8 | diag | U | Clathrin heavy chain 2 | P53675 | 16372274 |  |  |
| C22.0 | diag | U | DJ-1 |  | 23074380 | 42.0% | 83.0% | N02.8 | diag | U | Apolipoprotein C-IV precursor | P55056 | 16372274 |  |  |
| C22.1 | diag | U | interstitial collagens | P03956 | 22580416 | 83.0% | 79.0% | N02.8 | diag | U | Nuclear pore complex protein Nup107 | P57740 | 16372274 |  |  |
| C33,C34 | prog | U | 11α-hydroxy-9,15-dioxo-2,3,4,5-tetranor-prostane-1,20-dioic acid (PGE-M) |  | 245 |  |  | N02.8 | diag | U | Serine/threonine protein phosphatase 2A | P63151 | 16372274 |  |  |
| C33-C34 | diag | Br | nitric oxide (NO) |  | 22796631 |  |  | N02.8 | diag | U | Hemoglobin beta chain | P68871 | 16372274 |  |  |
| C33-C34 | diag | Br | volatile organic compounds (VOCs) |  | 22796631 | 84.6% | 80.0% | N02.8 | diag | U | Hemoglobin alpha chain | P69905 | 16372274 |  |  |
| C56 | diag | U | basic fibroblast growth factor |  | 26 | 70.0% | 75.0% | N02.8 | diag | U | Peroxisome proliferator activated receptor delta | Q03181 | 16372274 |  |  |
| C61 | prog | U | N-telopeptide (NTX) |  | 636 |  |  | N02.8 | diag | U | Zinc finger protein 155 | Q12901 | 16372274 |  |  |
| C61 | prog | U | total deoxypyridinoline (DPD) |  | 636 |  |  | N02.8 | diag | U | oligodendrocyte transcription factor 2 | Q13516 | 16372274 |  |  |
| C64 | diag | U | Syntenin-1 | O00560 | 23511837 | ~66% | ~72% | N02.8 | diag | U | Rho guanine nucleotide exchange factor 7 | Q14155 | 16372274 |  |  |
| C64 | diag | U | Podocalyxin (PODXL) | O00592 | 23511837 | 100.0% | 100.0% | N02.8 | diag | U | Dihydropyrimidinase related protein-1 | Q14194 | 16372274 |  |  |
| C64 | diag | U | Ceruloplasmin (CP) | P00450 | 23511837 | 100.0% | 100.0% | N02.8 | diag | U | Ras-related protein Rab-35 | Q15286 | 16372274 |  |  |
| C64 | diag | U | Neprilysin (CD10) | P08473 | 23511837 | ~75% | ~75% | N02.8 | diag | U | Calponin, acidic isoform | Q15417 | 16372274 |  |  |
| C64 | diag | U | MMP-9, Matrix metalloproteinase 9 | P14780 | 23511837 | ~90% | ~85% | N02.8 | diag | U | Thioredoxin reductase | Q16881 | 16372274 |  |  |
| C64 | diag | U | Dipeptidase 1 | P16444 | 23511837 | ~82% | ~63% | N02.8 | diag | U | vasorin precursor | Q6EMK4 | 21595033 |  |  |
| C64 | diag | U | Aquaporin-1(AQP1) | P29972 | 23511837 | ~85% | ~80% | N02.8 | diag | U | S100 calcium-binding pro-tein A15 | Q86SG5 | 16372274 |  |  |
| C64 | diag | U | 14-3-3 Protein beta/alpha | P31946 | 21553213 | ~90% | ~75% | N02.8 | diag | U | BTB and kelch domain con-taining protein 3 | Q8NAB2 | 16372274 |  |  |
| C64 | diag | U | Carbonic Anhydrase IX (CAIX) | Q16790 | 23511837 | ~78% | ~92% | N02.8 | diag | U | Nesprin 2 | Q8WXH0 | 16372274 |  |  |
| C64 | diag | U | kidney injury molecule-1 | Q96D42 | 15744000 | 82.0% | 90.0% | N02.8 | diag | U | Ankyrin repeat and SOCS box containing protein 13 | Q8WXK3 | 16372274 |  |  |
| C64 | diag | U | Dickkopf related protein 4 (DKK4) | Q9UBT3 | 23511837 | ~92% | ~100% | N02.8 | diag | U | Phosphatidylinositol 3-kinase regulatory gamma subunit | Q92569 | 16372274 |  |  |
| C64 | diag | U | Extracellular Matrix Metalloproteinase Inducer (EMMPRIN) |  | 23511837 | ~90% | ~85% | N02.8 | diag | U | GTP-binding protein Rhes | Q96D21 | 16372274 |  |  |
| C64.9 | diag | U | AQP1 | P29972 | 20375178 | 100.0% | 100.0% | N02.8 | diag | U | Signal-induced proliferation-associated protein 1 | Q96FS4 | 16372274 |  |  |
| C64.9 | diag | U | ADFP | Q6HFZ7 | 20375178 | 100.0% | 100.0% | N02.8 | diag | U | Microtubule-actin crosslinking factor 1, isoform 4 | Q96PK2 | 16372274 |  |  |
| C67 | prog | U | paraliprotein | P01766 | 400 |  |  | N02.8 | diag | U | Sideroflexin 1 | Q9H9B4 | 16372274 |  |  |
| C67 | prog | U | apolipoprotein A-IV | P06727 | 400 |  |  | N02.8 | diag | U | Zinc finger protein Kr 18 | Q9HCG1 | 16372274 |  |  |
| C67 | prog | U | lipid binding protein | P11597 | 400 |  |  | N02.8 | diag | U | DNA polymerase delta subunit 4 | Q9HCU8 | 16372274 |  |  |
| C67 | prog | U | FK506 binding protein 6 isoform | Q5T1M5 | 400 |  |  | N02.8 | diag | U | Exosome complex exonuclease RRP40 | Q9NQT5 | 16372274 |  |  |
| C71 | diag | U | creatine |  | 45 |  |  | N02.8 | diag | U | Spastin | Q9UBP0 | 16372274 |  |  |
| C75, E34.0 | diag | U | 5-hydroxytryptamine (5-HT) |  | 59 |  |  | N02.8 | diag | U | Vesicle transport through interaction with t-SNAREs homolog 1B | Q9UEU0 | 16372274 |  |  |
| C79.51 | diag | U | N-telopeptide (NTX) |  | 275 |  |  | N02.8 | diag | U | aminopeptidase N | Q9UIQ6 | 21595033 |  |  |
| C79.51 | diag | U | pyridinium cross-links pyridinoline (PYD) |  | 79 |  |  | N02.8 | diag | U | Zinc finger protein 221 | Q9UK13 | 16372274 |  |  |
| C79.51 | diag; prog | U | C-telopeptide |  | 275 |  |  | N02.8 | diag | U | Catenin delta-2 | Q9UQB3 | 16372274 |  |  |
| C79.51 | prog | U | N-telopeptide (NTX) |  | 16 |  |  | N02.8 | diag | U | RNA 3-terminal phosphate cyclase-like protein | Q9Y2P8 | 16372274 |  |  |
| C79.51,C50 | diag | U | deoxy-pyridinoline (DPD) |  | 304 |  |  | N02.8 | diag | U | Protein C20orf45 | Q9Y3B1 | 16372274 |  |  |
| C79.51,C50 | diag | U | N-telopeptide (NTX) |  | 304 |  |  | N02.8 | diag | U | IgA-uromodulin complex |  | 22415778 | 81.7% | 73.4% |
| C79.51,C61 | prog | U | Bone specific alkaline phosphatase (bALP) | P05186 | 423 |  |  | N02.8 | prog | U | alpha-1-antitrypsin | P01009 | 23161552 |  |  |
| C79.51,C61 | prog | U | N-telopeptide (NTX) |  | 423 |  |  | N02.8 | prog | U | albumin fragments | P02768 | 23161552 |  |  |
| C90.0 | diag | U | DPD | Q12882 | 330 | 88.9% | 83.3% | N02.8 | prog | U | α-1- β-glycoprotein | P25311 | 23161552 |  |  |
| C90.0,C50 | prog | U | N-telopeptide (NTX) |  | 321 |  |  | N02.8 | prog | U | LG3, laminin G-like 3 fragment of endorepellin | P98160 | 23161552 |  |  |
| D07.5 | diag | U | ABCA5 | Q8WWZ7 | 403 | ~100% |  | N02.8 | prog; ther | U | alpha-1-Antitrypsin precursor |  | 18095357 |  |  |
| D69.0 | prog; moni | U | urinary angiotensinogen (UAGT) |  | 21854508 |  |  | N02.8 | prog; ther | U | Kininogen-1 | P01042 | 18095357 |  |  |
| D89.8 | diag | Sa | Cystatin SN | P01037 | 22927351 |  |  | N02.8 | prog; ther | U | alpha-1-microglobulin | P02760 | 18095357 |  |  |
| D89.8 | diag | Sa | IgA | P01876 | 22927351 |  |  | N02.8 | prog; ther | U | Transthyretin precursor | P02766 | 18095357 |  |  |
| D89.8 | diag | U | Serum albumin | P02768 | 22927351 |  |  | N02.8 | prog; ther | U | CD59 glycoprotein  precursor | P13987 | 18095357 |  |  |
| D89.8 | diag | Sa | Lactoferrin | P02788 | 22927351 |  |  | N02.8 | prog; ther | U | Perlecan (frag) | P98160 | 18095357 |  |  |
| D89.8 | diag | Sa | SLPI | P03973 | 22927351 |  |  | N02.8 | prog; ther | U | Inter-a-trypsininhibitor heavy chain H4 precursor | Q14624 | 18095357 |  |  |
| D89.8 | diag | Sa | Salivary amylase | P04745 | 22927351 |  |  | N02.8 | ther | U | Serum albumin fragment | P02768 | 18095357 |  |  |
| D89.8 | diag | U | Leukotriene A4 hydrolase | P09960 | 22927351 |  |  | N03.2 | prog; moni | U | liver-type fatty acid-binding protein(L-FABP) | P07148 | 21958699 | 87.5% | 90.5% |
| D89.8 | diag | Sk | Elafin | P19957 | 23165480 |  |  | N04 | diag | U | Ig lambda light chain | .EMBL:CAA40949 | 19211645 |  |  |
| D89.8 | diag | U | Collagen α-1 | P20908 | 22927351 |  |  | N04 | diag | U | alpha 1 antitrypsin | P01009 | 19211645 |  |  |
| D89.8 | diag | Sa | albumin (ALB) | P43652 | 22927351 |  |  | N04 | diag | U | Leucine-rich alpha2 glycoprotein | P02750 | 19211645 |  |  |
| D89.8 | prog | Sk | Elafin | P19957 | 23165480 |  |  | N04 | diag | U | albumin | P02768 | 19211645 |  |  |
| E10 | prog; moni | U | Alpha-1 acid glycoprotein; Clusterin; Progranulin; Tamm-Horsfall glycoprotein | P02763; Q15846; P28799; P07911 | 22238279 |  |  | N04 | diag | U | alpha 1-B glycoprotein | P04217 | 21591266 |  |  |
| E10, E11 | diag | U | collagen alpha-1 (I) | P02452 | 20927192 |  |  | N04 | diag | U | Hypothetical protein NEB | P20929 | 19211645 |  |  |
| E10, E11 | diag | U | collagen alpha-1 (III) | P02461 | 20927192 |  |  | N04 | prog; ther | U | beta2-microglobulin | P61769 | 16810512 |  |  |
| E10, E11 | diag | U | a panel of 131 urinary biomarkers |  | 20927192 | ~83% | ~90% | N04.9 | diag; prog | U | monocyte chemoattractant protein-1 | P13500 | 21034351 |  |  |
| E10, E11 | diag | U | a panel of 261 urinary biomarkers |  | 20927192 | ~91% | ~78% | N13.7 | diag | U | tissue inhibitor of metalloproteinase 1 (TIMP1) | P01033 | 21969093 | 74.0% | 65.0% |
| E10.2 | prog | U | CTGF | P29279 | 16373901 |  |  | N13.7 | diag | U | matrix metalloproteinase 9 (MMP 9) | P08253 | 21969093 | 67.0% | 85.0% |
| E10.2, E11.2, E12.2, E13.2, E14.2 | diag | U | podocalyxin | O00592 | 22854890 |  |  | N13.7 | prog | U | tissue inhibitor of metalloproteinase 1 (TIMP1) | P01033 | 21969093 | 75.0% | 90.0% |
| E10.2, E11.2, E12.2, E13.2, E14.2 | diag | U | Nephrin | O60500 | 22698077 |  |  | N13.7 | prog | U | matrix metalloproteinase 9 (MMP 9) | P08253 | 21969093 | 81.2% | 85.0% |
| E10.2, E11.2, E12.2, E13.2, E14.2 | diag | U | heart-type fatty acid-binding protein (H-FABP) | P05413 | 22698077 |  |  | N14.1 | diag | U | Mannan-binding lectin serine protease 2 | O00187 | 19887787 |  |  |
| E10.2, E11.2, E12.2, E13.2, E14.2 | diag | U | Adiponectin | Q15848 | 22698077 |  |  | N14.1 | diag | U | Alpha-1-antitrypsin | P01009 | 19887787 |  |  |
| E10.2, E11.2, E12.2, E13.2, E14.2 | diag | U | Synaptopodin | Q8N3V7 | 22698077 |  |  | N14.1 | diag | U | Kininogen-1 isoform 2 | P01042 | 19887787 |  |  |
| E10.2, E11.2, E12.2, E13.2, E14.2 | diag | U | Podocin | Q9NP85 | 22698077; 21655212 | 81.4% | 62.5% | N14.1 | diag | U | Leucine-rich alpha-2-glycoprotein 1 | P02750 | 19887787 |  |  |
| E10.2, E11.2, E12.2, E13.2, E14.2 | diag | U | exosomal Wilm's tumor-1 protein (WT1) |  | 23544132 | 88.6% | 100.0% | N14.1 | diag | U | Alpha-1-microglobulin | P02760 | 19887787 |  |  |
| E10.2, E11.2, E12.2, E13.2, E14.2 | diag; prog | U | N-Acetyl-β-D-glucosamindase (NAG) | CID439454 | 22698077 |  |  | N14.1 | diag | U | Semenogelin-1 | P04279 | 19887787 |  |  |
| E10.2, E11.2, E12.2, E13.2, E14.2 | diag; prog | U | Type IV collagen | P53420,P29400,P02462,P08572,Q14031,Q01955 | 22698077 |  |  | N14.1 | diag | U | Liver-type fatty acid-binding protein | P07148 | 16490622 |  |  |
| E10.2, E11.2, E12.2, E13.2, E14.2 | prog | U | liver fatty acid-binding protein | P07148 | 22698077 |  |  | N14.1 | diag | U | Prolyl 4-hydroxylase subunit beta | P07237 | 19887787 |  |  |
| E10.2, E11.2, E12.2, E13.2, E14.2 | prog; ther | U | neutrophil gelatinase-associated lipocalin (NGAL) | P80188 | 22698077 |  |  | N14.1 | diag | U | CD59 glycoprotein | P13987 | 19887787 |  |  |
| E10.2, E11.2, E12.2, E13.2, E14.2 | prog; ther | U | kidney injury molecule-1 (KIM-1) | Q96D42 | 22698077 |  |  | N14.1 | diag | U | Zinc-alpha-2-glycoprotein chain B | P25311 | 19887787 |  |  |
| E11 | diag | U | haptoglobin precursor | P00738 | 20735160 |  |  | N14.1 | diag | U | Prostaglandin-H2 D-isomerase | P41222 | 19887787 |  |  |
| E11 | diag | U | transforming growth factor beta-1 | P01137 | 9133555 |  |  | N14.1 | diag | U | HBD-1 | P60022 | 18953418 |  |  |
| E11 | diag | U | retinol binding protein 4 | P02753 | 20735160 |  |  | N14.1 | diag | U | neutrophil gelatinase-associated lipocalin(NGAL) | P80188 | 17874137 | 73.0% | 100.0% |
| E11 | diag | U | alpha-1-microglobulin/bikunin precursor | P02760 | 20735160 |  |  | N14.1 | diag | U | Perlecan | P98160 | 19887787 |  |  |
| E11 | diag | U | transthyretin | P02766 | 20735160 |  |  | N14.1 | diag | U | mannose-binding lectin(MBL) | Q12907 | 19887787 |  |  |
| E11 | diag | U | albumin | P02768 | 20735160 |  |  | N14.1 | diag | U | Interleukin-18 | Q14116 | 18287807 | 69.2% | 76.9% |
| E11 | diag | U | E-cadherin increased | P12830 | 20735160 |  |  | N14.1 | diag | U | CGI-70 protein | Q9Y5K5 | 19887787 |  |  |
| E11 | diag | U | zinc alpha-2 glycoprotein | P25311 | 20735160 |  |  | N14.1 | diag | U | Ig lambda light chain variable region |  | 19887787 |  |  |
| E11 | diag | U | neutrophil-gelatinase-associated lipocalin(NGAL) | P80188 | 22015481 |  |  | N14.1 | prog | U | neutrophil gelatinase-associated lipocalin | P80188 | 20072790 | 80.0% | 75.0% |
| E11 | diag | U | kidney injury molecule-1（KIM-1） | Q96D42 | 22015481 |  |  | N14.1 | prog | U | Interleukin-18 | Q14116 | 18287807 |  |  |
| E11 | moni; prog | U | N-acetyl-D-glucosaminidase (NAG) | O60502 | 21779943 |  |  | N15.0 | diag | U | alpha1-microglobulin | P02760 | 21332340 | 70.2% | 82.6% |
| E11 | moni; prog | U | neutrophil-gelatinase-associated lipocalin(NGAL) | P80188 | 21779943 |  |  | N15.0 | diag | U | Beta2-microglobulin | P61769 | 21332340 | 72.3% | 84.4% |
| E11 | moni; prog | U | kidney injury molecule-1（uKIM-1） | Q96D42 | 21779943 |  |  | N15.0 | diag | U | albumin |  | 21332340 | 65.2% | 80.4% |
| E23.2 | diag | U | aquaporin-2 | P41181 | 7537863 |  |  | N15.0 | diag | U | total protein |  | 21332340 | 65.9% | 92.1% |
| E26.02 | diag | U | Phosphorylated (active) form of the sodium chloride cotransporter (pNCC) | O76030 | 22851731 |  |  | N15.0 | prog | U | Beta2-microglobulin | P61769 | 21332340 |  |  |
| E76 | diag | U | glycosaminoglycans |  | 22658917 |  |  | N17 | diag | U | N-Acetyl-β-D-glucosamindase (NAG) | CID439454 | 22983082 | 99.0% | 100.0% |
| E76 | diag | U | oligosaccharides |  | 22658917 |  |  | N17 | diag | U | cysteine rich protein 61 (CYR 61) | O00622 | 22983082 |  |  |
| E76 | prog | U | oligosaccharides |  | 22658917 |  |  | N17 | diag | U | NETRIN – 1 | O95631 | 22983082 |  |  |
| E76 | prog; ther | U | glycosaminoglycans |  | 22658917 |  |  | N17 | diag | U | cystatin C (CyC) | P01034 | 22731900 | 73.0% | 85.0% |
| E76 | ther | U | oligosaccharides |  | 22658917 |  |  | N17 | diag | U | Retinol Binding Protein (RBP) | P02753,P10745,P09455,P82980,P50120,Q96R05 | 22983082 |  |  |
| E83.52 | diag | U | Monocyte chemotactic protein (MCP-1) | P13500 | 22223141 |  |  | N17 | diag | U | α-1 MICROGLOBULIN | P02760 | 22983082 |  |  |
| E84 | diag | Br | 2-aminoacetophenone |  | 21054900 | 93.8% | 69.2% | N17 | diag | U | Fetuin A | P02765 | 22983082 |  |  |
| E84 | diag | Br | hydrogen cyanide |  | 22796631 |  |  | N17 | diag | U | microalbumin | P02768 | 22983082 |  |  |
| E84 | moni | Br | 8-isoprostane |  | 22796631 |  |  | N17 | diag | U | Fatty acid-binding protein 1 (FABP1) | P07148 | 22731900 |  |  |
| E84 | moni | Br | CO |  | 22796631 |  |  | N17 | diag | U | Osteopontin (OPN) | P10451 | 22983082 |  |  |
| E84 | moni | Br | prostaglandin E2 (PGE2) |  | 22796631 |  |  | N17 | diag | U | Sodium/Hydrogen exchanger isoform (NHE3) | P48764 | 22983082 |  |  |
| E85.8 | diag | U | monoclonal free immunoglobulin light chains |  | 22627261 | 81.3% | 98.0% | N17 | diag | U | β-2 microglobulin (B2M) | P61769 | 22983082 |  |  |
| E88.81 | prog | U | cystatin C | P01034 | 21051748 |  |  | N17 | diag | U | neutrophil gelatinase-associated lipocalin (NGAL) | P80188 | 22731900 | 100.0% | 98.0% |
| F40-F42 | diag | U | IL-8 | P10145 | 23977105 | ~100% |  | N17 | diag | U | Fatty acid binding protein (FABP) | Q01469,P05413,P12104,P07148,P15090,Q0Z7S8,O15540,A6NFH5 | 22983082 |  |  |
| F98.0 | prog | U | aquaporin-2 | P41181 | 11004218 |  |  | N17 | diag | U | IL-18 | Q14116 | 22983082 | 69.0% | 92.0% |
| G20, F02.3 | diag | U | 8-hydroxy-2-deoxyguanosine (8-OHdG) |  | 23587062 |  |  | N17 | diag | U | Clusterin | Q96AJ1 | 22983082 |  |  |
| G35 | diag | U | free immunoglobulin light chain | P01708 | 20171744 |  |  | N17 | diag | U | kidney injury molecule-1 (KIM-1) | Q96D42 | 22983082 | 90.0% | 96.0% |
| G93.4 | diag | U | S100B | P04271 | 19590245 | 90.0% | 92.0% | N17 | prog | U | NETRIN – 1 | O95631 | 22983082 |  |  |
| G93.4 | diag | U | lactate/creatinine ratio |  | 19590245 | 92.0% | 90.0% | N17 | prog | U | IL-18 | Q14116 | 22983082 | >90% | >90% |
| G93.4 | diag | U | S100B; lactate/creatinine ratio |  | 19590245 | 99.0% | 97.0% | N18.9 | diag | U | Monocyte chemoattractant protein-1 (MCP-1) | P13500 | 22609259 |  |  |
| G93.4 | prog | U | S100B | P04271 | 19590245 |  |  | N18.9 | diag | U | Connective tissue growth factor | P29279 | 21292366 |  |  |
| G93.4 | prog | U | S100B; lactate/creatinine ratio |  | 19590245 |  |  | N18.9 | diag; moni | U | platelet-derived growth factor B | P01127 | 17095863 |  |  |
| H00-H59 | diag | T | Vasoactive intestinal peptide (VIP) | P01282 | 22895048 |  |  | N18.9 | diag; moni | U | transforming growth factor beta1 | P01137 | 17095863 |  |  |
| H00-H59 | diag | T | Hemopexin | P02790 | 22895048 |  |  | N18.9 | diag; moni | U | TNF-alpha | P01375 | 17095863 |  |  |
| H00-H59 | diag | T | Calcitonin gene-related petide (CGRP) | P06881,P10092 | 22895048 |  |  | N18.9 | diag; moni | U | intercellular adhesion molecule 1 | P05362 | 17095863 |  |  |
| H00-H59 | diag | T | Eosinophil cationic protein (ECP) | P12724 | 22895048 |  |  | N18.9 | diag; moni | U | matrix metalloproteinase-2 (MMP-2) | P08253 | 17095863 |  |  |
| H00-H59 | diag | T | Substance P (SP) | P20366 | 22895048 |  |  | N18.9 | diag; moni | U | RANTES | P13501 | 17095863 |  |  |
| H00-H59 | moni | T | Hemopexin | P02790 | 22895048 |  |  | N18.9 | diag; moni | U | MMP-9 | P14780 | 17095863 |  |  |
| H16.229 | diag | T | a-antitrypsin | P01009 | 22895048 |  |  | N18.9 | diag; moni | U | vascular endothelial growth factor (VEGF) | P15692 | 17095863 |  |  |
| H16.229 | diag | T | Cystatin SN | P01037 | 22895048 |  |  | N18.9 | diag; moni | U | E-selectin | P16581 | 17095863 |  |  |
| H16.229 | diag | T | Nerve growth factor(NGF) | P01138 | 22895048 |  |  | N18.9 | diag; moni | U | vascular cell adhesion molecule 1 | P19320 | 17095863 |  |  |
| H16.229 | diag | T | Neuropeptide Y (NPY) | P01303 | 22895048 |  |  | N18.9 | diag; moni | U | Fas | P25445 | 17095863 |  |  |
| H16.229 | diag | T | TNF-alpha | P01375 | 22895048 |  |  | N18.9 | diag; moni | U | VE-cadherin | P33151 | 17095863 |  |  |
| H16.229 | diag | T | IFNγ | P01579 | 22895048 |  |  | N18.9 | diag; moni | U | interleukin-2 | P60568 | 17095863 |  |  |
| H16.229 | diag | T | IL-1β | P01584 | 22895048 |  |  | N18.9 | diag; moni | U | tissue inhibitors of metalloproteinase-1 (TIMP-1) | Q58P21 | 17095863 |  |  |
| H16.229 | diag | T | MMP1 | P03956 | 22895048 |  |  | N18.9 | moni | U | Monocyte chemoattractant protein 1(MCP-1) | P13500 | 17095863 |  |  |
| H16.229 | diag | T | IL-6 | P05231 | 22895048 |  |  | N18.9 | prog | U | TNF-alpha | P01375 | 20525973 |  |  |
| H16.229 | diag | T | Calcitonin gene-related petide (CGRP) | P06881,P10092 | 22895048 |  |  | N18.9 | prog | U | retinol-binding protein (RBP) | P02753 | 22981148 |  |  |
| H16.229 | diag | T | MMP-2 | P08253 | 22895048 |  |  | N18.9 | prog | U | Monocyte chemotactic protein (MCP-1) | P13500 | 22981148 |  |  |
| H16.229 | diag | T | Matrix Metalloproteinase 7 (MMP7) | P09237 | 22895048 |  |  | N18.9 | prog | U | NGAL, Urinary neutrophil gelatinase-associated lipocalin | P80188 | 23328709 |  |  |
| H16.229 | diag | T | MMP-10 | P09238 | 22895048 |  |  | N20.0 | diag | U | Ig alpha 1 chain C region | P01876 | 19145410 |  |  |
| H16.229 | diag | T | IL-8 | P10145 | 22895048 |  |  | N20.0 | diag | U | Sequence31 from patent EP115804 | . EMBL CDS:CAD19027 | 19145410 |  |  |
| H16.229 | diag | T | CCL3/MIP1a | P10147 | 22895048 |  |  | N20.0 | diag | U | Alpha-1-antitrypsin precursor | P01009 | 19145410 |  |  |
| H16.229 | diag | T | CCL4/MIP1b | P13236 | 22895048 |  |  | N20.0 | diag | U | Ig kappa V 1-5 protein | P01602 | 19145410 |  |  |
| H16.229 | diag | T | CCL5/RANTES | P13501 | 22895048 |  |  | N20.0 | diag | U | Ig heavy chain V region precursor | P01743 | 19145410 |  |  |
| H16.229 | diag | T | Lipocalin-1 | P31025 | 22895048 |  |  | N20.0 | diag | U | Ig heavy chain Fab fragment | P01825 | 19145410 |  |  |
| H16.229 | diag | T | S100A8 (CalgranulinA) |  | 22895048 |  |  | N20.0 | diag | U | Ig heavy chain variable region | P01834 | 19145410 |  |  |
| H16.229 | diag | T | S100A9 (CalgranulinB) |  | 22895048 |  |  | N20.0 | diag | U | Ig gamma-1 chain C region | P01857 | 19145410 |  |  |
| H16.229 | diag; moni | T | matrix metalloproteinase 9 (MMP 9) | P14780 | 22895048; 23307206 | 85.0% | 94.0% | N20.0 | diag | U | Ig gamma-2 chain C region | P01859 | 19145410 |  |  |
| H16.229 | moni | T | Nerve growth factor(NGF) | P01138 | 22895048 |  |  | N20.0 | diag | U | Ig gamma-3 heavy chain disease proteins | P01860 | 19145410 |  |  |
| H16.229 | moni | T | Neuropeptide Y (NPY) | P01303 | 22895048 |  |  | N20.0 | diag | U | Ig heavy chain constant region gamma 4 (fragment) | P01861 | 19145410 |  |  |
| H16.229 | moni | T | Calcitonin gene-related petide (CGRP) | P06881,P10092 | 22895048 |  |  | N20.0 | diag | U | Serum albumin fragment | P02768 | 19145410 |  |  |
| H40-H42 | diag | eye | glucocorticoid receptor GRβ | P04150 | 22827637 |  |  | N20.0 | diag | U | Serum albumin precursor | P02768 | 19145410 |  |  |
| I15.0 | diag | U | Neutrophil gelatinase-associated lipocalin (NGAL) | P80188 | 22923545 |  |  | N20.0 | diag | U | Transferrin precursor | P02787 | 19145410 |  |  |
| I27.0, I27.2 | moni; ther | Br | fractional exhaled nitric oxide (FeNO) |  | 22796631 |  |  | N20.0 | diag | U | Uromodulin | P07911 | 19145410 |  |  |
| I48 | diag | U | Isoprostanes |  | 22628539 |  |  | N20.0 | diag | U | osteopontin | P10451 | 8761944 |  |  |
| I50 | diag | U | kidney injury molecule-1 (KIM-1) | Q96D42 | 22980054 |  |  | N20.0 | diag | U | Ig alpha heavy chain 1 | Q569J1 | 19145410 |  |  |
| I50 | prog | U | N-Acetyl-β-D-glucosamindase (NAG) | CID439454 | 22489715 |  |  | N20.0 | diag | U | KIAA0266 protein | Q5TAP6 | 19145410 |  |  |
| I50 | prog | U | kidney injury molecule-1 (KIM-1) | Q96D42 | 22980054 |  |  | N20.0 | diag | U | Hypothetical protein | Q6P005 | 19145410 |  |  |
| I82.4,I82.5 | diag | U | fibrinopeptide B (and des-arginine fibrinopeptide B) | P02675 | 12893031 | 100.0% | 85.0% | N20.0 | diag | U | Ig heavy chain (fragment) | Q9UGP3 | 19145410 |  |  |
| J40-J44, J47 | diag | U | desmosine |  | 23361193 |  |  | N20.0 | diag | U | antibody a5b7,chainB |  | 19145410 |  |  |
| J40-J44, J47 | diag | Br | ethane |  | 22796631 |  |  | N21.0-N21.9 | diag | U | IL-6 | P05231 | 20204339 |  |  |
| J40-J44, J47 | diag | Br | pentane |  | 22796631 |  |  | N21.0-N21.9 | diag | U | monocyte chemoattractant protein-1 | P13500 | 20204339 |  |  |
| J40-J44, J47 | moni | Br | CO |  | 22796631 |  |  | N21.0-N21.9 | diag; moni | U | IL-8 | P10145 | 20204339 | 90.0% | 68.0% |
| J40-J44, J47 | moni | Br | ethane |  | 22796631 |  |  | N30.10, N30.11 | diag | U | neutrophil elastase | B2MUD5 | 18609268 |  |  |
| J45 | diag | Br | C-reactive protein (CRP) | P02741 | 22796631 |  |  | N30.10, N30.11 | diag | U | kininogens | P01042 | 18455532 |  |  |
| J45 | diag | Br | cysteinyl leukotrienes (cysLTs) |  | 22796631 | 73.6% |  | N30.10, N30.11 | diag | U | NGF | P01138 | 19751258 | 75.0% | 65.5% |
| J45 | diag | Br | EBC pH |  | 22796631 |  |  | N30.10, N30.11 | diag | U | methylhistamine | P05231 | 17070335 |  |  |
| J45 | diag | Sp | eosinophils |  | 22877617 | 86.0% | 88.0% | N30.10, N30.11 | diag | U | uromodulin | P07911 | 18455532 |  |  |
| J45 | diag | Br | ethane |  | 22796631 |  |  | N30.10, N30.11 | diag | U | inter-alpha-trypsin inhibitor heavy chain H4 | Q14624 | 18455532 |  |  |
| J45 | diag | Br | fragment of exhaled nitric oxide (FENO) |  | 22796631 |  |  | N30.10, N30.11 | diag | U | hepatocarcinoma-intestine-pancreas (HIP)/PAP |  | 19646740 |  |  |
| J45 | diag | Br | H2O2 |  | 22796631 |  |  | N30.10, N30.11 | diag | U | histamine |  | 17070335 |  |  |
| J45 | diag | Br | Nitrate |  | 22796631 |  |  | N30.10, N30.11 | diag | U | methylhistamine; interleukin-6 |  | 17070335 | 70.0% | 72.4% |
| J45 | diag | Br | nitrite |  | 22796631 |  |  | N32.81 | diag; moni | U | Nerve growth factor(NGF) | P01138 | 23314226 |  |  |
| J45 | diag; moni | Br | pentane |  | 22796631 |  |  | N32.81 | diag; moni | U | monocyte chemoattractant protein 1 (MCP-1) | P13500 | 23314226 |  |  |
| J45 | moni | Br | endothelin-1 (ET-1) | P05305 | 22796631 |  |  | N32.81 | diag; moni | U | brain derived neurotrophic factor (BDNF) | P23560 | 23314226 |  |  |
| J45 | moni | Br | CO |  | 22796631 |  |  | N32.81 | diag; moni | U | Fas/TNFRSF6 | P25445 | 23314226 |  |  |
| J45 | moni | Sp | eosinophils |  | 22877617 |  |  | N32.81 | diag; moni | U | PGF2 | P43088 | 23314226 |  |  |
| J45 | moni | Br | hs-CRP; exhaled NO |  | 19148935 |  |  | N32.81 | diag; moni | U | PGE2 | P43116 | 23314226 |  |  |
| J45 | moni | Br | nitric oxide (NO) |  | 22796631 |  |  | N32.81 | diag; moni | U | PARC | P55774 | 23314226 |  |  |
| J45 | moni; ther | Br | fragment of exhaled nitric oxide (FENO) |  | 22796631 |  |  | N32.81 | diag; moni | U | TARC | Q92583 | 23314226 |  |  |
| J45 | prog; ther | Sp | eNO; eosinophils |  | 22877617 |  |  | N32.81,N30.1 | diag; moni | U | Nerve growth factor(NGF) | P01138 | 23314226 |  |  |
| J45 | prog; ther | Sp | eosinophils |  | 22877617 |  |  | N39.0 | diag | U | Fibrinogen alpha chain | P02671 | 22075168 |  |  |
| J84.1 | moni | Br | CO |  | 22796631 |  |  | N39.8 | diag | U | PDGF | P01127;P04085;Q9NRA1 Q9GZP0 | 15140760 |  |  |
| K02 | diag | Sa | A 17 kDa molecular weight protein |  | 23142096 |  |  | N39.8 | diag | U | Chemokine 14 | Q16627;O95715 | 15140760 |  |  |
| K02 | diag | Sa | Basic proline-rich peptide |  | 23142096 |  |  | N39.8 | diag | U | Mannan-binding lectin serine protease 2 | O00187 | 15140760 |  |  |
| K02 | diag | Sa | Total protein (what protein?) |  | 23142096 |  |  | N39.8 | diag | U | Complement factor D | P00746 | 15140760 |  |  |
| K35-K37 | diag | U | Mannan-binding lectin serine protease 2 | O00187 | 19556024 |  |  | N39.8 | diag | U | Urokinase | P00749 | 15140760 |  |  |
| K35-K37 | diag | U | Plasminogen | P00747 | 19556024 |  |  | N39.8 | diag | U | Complement factor B | P00751 | 15140760 |  |  |
| K35-K37 | diag | U | alpha-1-Antichymotrypsin | P01011 | 19556024 |  |  | N39.8 | diag | U | Carbonic anhydrase | P00915 | 15140760 |  |  |
| K35-K37 | diag | U | leucine-rich alpha glycoprotein-1 (LRG) | P02750 | 22221321 | 100.0% | 23.0% | N39.8 | diag | U | Angiotensinogen | P01019 | 15140760 |  |  |
| K35-K37 | diag | U | leucine-rich alpha-2-glycoprotein (LRG) | P02750 | 22305331 | 95.0% | 100.0% | N39.8 | diag | U | Cystatin C | P01034 | 15140760 |  |  |
| K35-K37 | diag | U | alpha-1-Acid glycoprotein 1 | P02763 | 19556024 |  |  | N39.8 | diag | U | Kininogen | P01042 | 15140760 |  |  |
| K35-K37 | diag | U | Apolipoprotein D | P05090 | 19556024 |  |  | N39.8 | diag | U | EGF (urogastrone) | P01133 | 15140760 |  |  |
| K35-K37 | diag | U | S100-A8 | P05109 | 19556024 | ~71% | ~90% | N39.8 | diag | U | IGF-II | P01344 | 15140760 |  |  |
| K35-K37 | diag | U | Zinc-alpha-2-glycoprotein | P25311 | 19556024 |  |  | N39.8 | diag | U | TNF | P01375 | 15140760 |  |  |
| K50 | prog; moni | U | albumin | P02768 | 20653491 |  |  | N39.8 | diag | U | Polymeric-immunoglobulin receptor | P01833 | 15140760 |  |  |
| K50,K51 | diag | F | alpha 1 anti-trypsin | P01009 | 22424434 |  |  | N39.8 | diag | U | Apolipoprotein AI | P02647 | 15140760 |  |  |
| K50,K51 | diag | F | alpha-2-macroglobulin | P01023 | 22424434 |  |  | N39.8 | diag | U | Apolipoprotein E | P02649 | 15140760 |  |  |
| K50,K51 | diag | F | TNF-alpha | P01375 | 22424434 |  |  | N39.8 | diag | U | Apolipoprotein CIII | P02656 | 15140760 |  |  |
| K50,K51 | diag | F | IL-1β | P01584 | 22424434 |  |  | N39.8 | diag | U | beta-2-glycoprotein 1 | P02749 | 15140760 |  |  |
| K50,K51 | diag | F | lactoferrin | P02788 | 22424434 | 80.0% | 82.0% | N39.8 | diag | U | Fibronectin FN70 | P02751 | 15140760 |  |  |
| K50,K51 | diag | F | calprotectin | P05109 | 22424434 | 98.0% | 91.0% | N39.8 | diag | U | Retinol binding protein | P02753 | 15140760 |  |  |
| K50,K51 | diag | F | IL-4 | P05112 | 22424434 |  |  | N39.8 | diag | U | Transthyretin | P02766 | 15140760 |  |  |
| K50,K51 | diag | F | Myeloperoxidase (MPO) | P05164 | 22424434 |  |  | N39.8 | diag | U | Vitamin D binding protein (VDBP) | P02774 | 15140760 |  |  |
| K50,K51 | diag | F | elastase | P08246,P08861,P08217,P09093,Q9UNI1,P08218,P39900 | 22424434 |  |  | N39.8 | diag | U | proplatelet basic peptide | P02775 | 15140760 |  |  |
| K50,K51 | diag | F | leukocyte esterase | P09871 | 22424434 |  |  | N39.8 | diag | U | Hemopexin | P02790 | 15140760 |  |  |
| K50,K51 | diag | F | M2-pyruvate kinase | P14618 | 22424434 |  |  | N39.8 | diag | U | Angiogenin | P03950 | 15140760 |  |  |
| K50,K51 | diag | F | IL-10 | P22301 | 22424434 |  |  | N39.8 | diag | U | Apolipoprotein D | P05090 | 15140760 |  |  |
| K50,K51 | diag | F | lysozyme | P61626 | 22424434 |  |  | N39.8 | diag | U | Myeloperoxidase | P05164 | 15140760 |  |  |
| K50,K51 | diag | F | S100A12 | P80511 | 22424434 | 86.0% | 96.0% | N39.8 | diag | U | Lithostathine | P05451;P48304 | 15140760 |  |  |
| K50,K51 | moni | F | alpha 1 anti-trypsin | P01009 | 22424434 |  |  | N39.8 | diag | U | Complement factor C2 | P06681 | 15140760 |  |  |
| K50,K51 | moni | F | alpha-2-macroglobulin | P01023 | 22424434 |  |  | N39.8 | diag | U | Apolipoprotein A-IV | P06727 | 15140760 |  |  |
| K50,K51 | moni | F | TNF-alpha | P01375 | 22424434 |  |  | N39.8 | diag | U | a-Enolase | P06733 | 15140760 |  |  |
| K50,K51 | moni | F | IL-1β | P01584 | 22424434 |  |  | N39.8 | diag | U | diazepam binding inhibitor | P07108 | 15140760 |  |  |
| K50,K51 | moni | F | lactoferrin | P02788 | 22424434 | 70-100% | 44-100% | N39.8 | diag | U | L-Lactate dehydrogenase B chain | P07195 | 15140760 |  |  |
| K50,K51 | moni | F | calprotectin | P05109 | 22424434 | 70-100% | 44-100% | N39.8 | diag | U | Profilin 1 | P07737 | 15140760 |  |  |
| K50,K51 | moni | F | IL-4 | P05112 | 22424434 |  |  | N39.8 | diag | U | Uromodulin | P07911 | 15140760 |  |  |
| K50,K51 | moni | F | Myeloperoxidase (MPO) | P05164 | 22424434 |  |  | N39.8 | diag | U | Complement factor H | P08603 | 15140760 |  |  |
| K50,K51 | moni | F | elastase | P08246,P08861,P08217,P09093,Q9UNI1,P08218,P39900 | 22424434 |  |  | N39.8 | diag | U | Complement factor H-related | P08603 | 15140760 |  |  |
| K50,K51 | moni | F | leukocyte esterase | P09871 | 22424434 |  |  | N39.8 | diag | U | osteopontin | P10451 | 15140760 |  |  |
| K50,K51 | moni | F | M2-pyruvate kinase | P14618 | 22424434 |  |  | N39.8 | diag | U | Apolipoprotein J | P10909 | 15140760 |  |  |
| K50,K51 | moni | F | IL-10 | P22301 | 22424434 |  |  | N39.8 | diag | U | Heat shock cognate 71-kDa protein | P11142 | 15140760 |  |  |
| K50,K51 | moni | F | lysozyme | P61626 | 22424434 |  |  | N39.8 | diag | U | Urinary protein 1 | P11684 | 15140760 |  |  |
| K50,K51 | moni | F | S100A12 | P80511 | 22424434 |  |  | N39.8 | diag | U | Angiotensin-converting enzyme | P12821 | 15140760 |  |  |
| K50,K51 | prog | F | lactoferrin | P02788 | 22424434 |  |  | N39.8 | diag | U | bone morphogenic protein-1 | P13497 | 15140760 |  |  |
| K50,K51 | prog | F | calprotectin | P05109 | 22424434 | 90.0% | 83.0% | N39.8 | diag | U | CD59 | P13987 | 15140760 |  |  |
| K50,K51 | prog | F | S100A12 | P80511 | 22424434 |  |  | N39.8 | diag | U | Acid phosphatase | P15309 | 15140760 |  |  |
| K50,K51 | ther | F | lactoferrin | P02788 | 22424434 |  |  | N39.8 | diag | U | Lysozyme | P16973 | 15140760 |  |  |
| K50,K51 | ther | F | calprotectin | P05109 | 22424434 |  |  | N39.8 | diag | U | IGF binding protein 2 | P18065 | 15140760 |  |  |
| K85 | diag | U | alpha-1 antitrypsin | P01009 | 18345293 |  |  | N39.8 | diag | U | TNF receptor superfamily | P20333;P19438 | 15140760 |  |  |
| K85 | diag | U | Collagen alpha1(I) precursor | P02452 | 18345293 |  |  | N39.8 | diag | U | IGF binding protein 4 | P22692 | 15140760 |  |  |
| K85 | diag | U | Fibrinogen | P02679 | 18345293 |  |  | N39.8 | diag | U | IGF binding protein 6 | P24592 | 15140760 |  |  |
| K85 | diag | U | trypsinogen | P07477，P35030，P07478，Q8NHM4 | 17119188 | 100.0% | 96.0% | N39.8 | diag | U | IGF binding protein 5 | P24593 | 15140760 |  |  |
| K85 | diag | U | trypsinogen-2 | P07478 | 10606910 | 96.0% | 92.0% | N39.8 | diag | U | CD27 (TNF receptor) | P26842 | 15140760 |  |  |
| K85 | diag | U | Collagen alpha2(I) precursor | P08123 | 18345293 |  |  | N39.8 | diag | U | Pigment epithelium-derived factor (PEDF) | P36955 | 15140760 |  |  |
| K85 | diag | U | Collagen alpha2(IV) precursor | P08572 | 18345293 |  |  | N39.8 | diag | U | Cytoplasmic antiproteinase 2. (CAP2) | P50452 | 15140760 |  |  |
| K85 | diag | U | Collagen alpha(XII) | Q99715 | 18345293 |  |  | N39.8 | diag | U | beta2-microglobulin | P61769 | 15140760 |  |  |
| K85 | prog | U | trypsinogen-2 | P07478 | 11719473 | 62.0% | 87.0% | N39.8 | diag | U | neutrophil gelatinase-associated lipocalin(NGAL) | P80188 | 15140760 |  |  |
| K85 | prog | U | trypsinogen activation peptide(TAP) |  | 14612286 | 91.7% | 89.7% | N39.8 | diag | U | Perlecan | P98160 | 15140760 |  |  |
| K85 | prog | U | trypsinogen activation peptide(TAP); CRP |  | 15286966 | 35.0% | 95.0% | N39.8 | diag | U | Guanylate cyclase soluble subunit beta-1 | Q02153 | 15140760 |  |  |
| K85, K86.0-K86.1 | diag | U | trypsinogen-2 | P07478 | 9255526 | 81.0% | 97.0% | N39.8 | diag | U | Latent TGF-beta BP | Q14766 | 15140760 |  |  |
| K85, K86.0-K86.1 | diag | U | amylase |  | 9255526 | 81.0% | 95.0% | N39.8 | diag | U | IGF binding protein 7 | Q16270 | 15140760 |  |  |
| L40 | diag | Sk | IL-23R | Q5VWK5 | 23532439 |  |  | N39.8 | diag | U | Glutaminyl-peptide cyclotransferase precursor | Q16769 | 15140760 |  |  |
| L40 | diag | Sk | IL-23 | Q9NPF7 | 23532439 |  |  | N39.8 | diag | U | Similar to orosomucoid | Q8N138 | 15140760 |  |  |
| L40 | diag | Sk | miR125b |  | 23532439 |  |  | N39.8 | diag | U | Betaine-homocysteine S-methyltransferase | Q93088 | 15140760 |  |  |
| L40 | diag | Sk | miR-146a |  | 23532439 |  |  | N39.8 | diag | U | Phosphatidylethanolamine binding protein | Q96S96 | 15140760 |  |  |
| L40 | diag | Sk | miR-203 |  | 23532439 |  |  | N39.8 | diag | U | Mdm-1 protein | Q9D067 | 15140760 |  |  |
| L40 | diag | Sk | miR-21 |  | 23532439 |  |  | N39.8 | diag | U | angiomodulin |  | 15140760 |  |  |
| L40 | diag | Sk | Th17 |  | 23532439 |  |  | N80 | diag | U | Cytokeratins-19 | P08727 | 21168580 |  |  |
| M00-M25 | diag | U | FGB | A0JLR9 | 21124648 |  |  | NOPAGE | diag | Br | nitric oxide (NO) |  | 22796631 |  |  |
| M00-M25 | diag | U | A1AT | P01009 | 21124648 |  |  | O11,O14 | diag | U | Serpina 1 | C5J0G2 | 18984079 |  |  |
| M00-M25 | diag | U | COL1A1 | P02452 | 21124648 |  |  | O11,O14 | diag | U | Podocalyxin | O00592 | 22301621 |  |  |
| M00-M25 | diag | U | COL3A1 | P02461 | 21124648 |  |  | O11,O14 | diag | U | Nephrin | O60500 | 22301621 |  |  |
| M00-M25 | diag | U | UMOD | P07911 | 21124648 |  |  | O11,O14 | diag | U | orosomucoid | P02763,P19652,Q5T539,Q5T538 | 20392509 |  |  |
| M00-M25 | diag | U | type II collagen | Q02388 | 20980286 |  |  | O11,O14 | diag | U | albumin | P02768 | 18984079 |  |  |
| M00-M25 | diag | U | COL9A2 | Q14055 | 21124648 |  |  | O11,O14 | diag | U | soluble endoglin | P17813 | 19943826 |  |  |
| M00-M25 | diag | U | FGA | Q6NSD8 | 21124648 |  |  | O11,O14 | diag; prog | U | soluble fms-like tyrosine kinase 1 | P17948 | 19943826 |  |  |
| M00-M25 | diag | U | COL1A2 | Q7KZ71 | 21124648 |  |  | O11,O14 | diag; prog | U | placental growth factor | P49763 | 19943826 |  |  |
| M00-M25 | diag | U | 17-urine-peptide biomarker panel |  | 21124648 | ~85% | ~100% | O11,O14 | diag; prog | U | Beta Ig-h3 | Q15582 | 22301621 |  |  |
| M15-M19,M47 | diag | U | C-telopeptide of type II collagen (uCTX2) | P02458 | 20305824 |  |  | O11,O14 | prog | U | orosomucoid | P02763,P19652,Q5T539,Q5T538 | 17653877 | ~0.56 | ~0.73 |
| M15-M19,M47 | diag | U | Fibulin-3 |  | 22275171 | 74.6% | 85.7% | P27.1 | diag | U | 8-hydroxy-2-deoxyguanosine (8-OHdG) |  | 23523392 | 85.7% | 61.1% |
| M15-M19,M47 | diag | U | C-terminal cross- linked telopeptide of type II collagen (CTX-II) |  | 17538566 |  |  | P27.1 | diag | U | Bombesin-like peptide |  | 23523392 | 54.0% | 90.0% |
| M15-M19,M47 | diag | U | N-terminal cross-linked telopeptide of type I collagen (NTX-I) |  | 17538566 |  |  | P27.1 | diag | Br | Carbon monoxide |  | 23523392 | 50.0% |  |
| M15-M19,M47 | diag | U | pyridinoline (PYD) |  | 17538566 |  |  | P27.1 | diag | Br | nitric oxide (NO) |  | 23523392 | 57.5% |  |
| M15-M19,M47 | prog | U | COL2-3/4C (long) epitope | P02458 | 17538566 |  |  | P27.1 | diag | Oral mucosa | spectrophotometry |  | 23523392 |  |  |
| M15-M19,M47 | prog | U | COL2-3/4C (short) epitope | P02458 | 17538566 |  |  | P77 | diag | U | Claudin-3 | O15551 | 20485148 |  |  |
| M15-M19,M47 | prog | U | C-terminal cross-linked telopeptide of type I collagen (CTX-I) |  | 17538566 |  |  | P77 | diag | U | Calprotectin | P06702 | 20485148 |  |  |
| M15-M19,M47 | prog | U | helical type II collagen (HELIX-II) |  | 17538566 |  |  | P77 | diag | U | Intestinal fatty acid binding protein | P12104 | 20485148 |  |  |
| M15-M19,M47 | prog | U | nine-amino-acid peptide of type II collagen (Coll 2-1) |  | 17538566 |  |  | P77 | prog | U | Intestinal fatty acid binding protein | P12104 | 20485148 |  |  |
| M15-M19,M47 | prog | U | nitrated form of nine-amino-acid peptide of type II collagen (Coll 2-1 NO2) |  | 17538566 |  |  | Q61 | diag | U | heart-type fatty acid-binding protein (H-FABP) | P05413 | 22846584 |  |  |
| M15-M19,M47 | prog | U | Pentosidine |  | 17538566 |  |  | Q61 | diag | U | monocyte chemoattractant protein 1 (MCP-1) | P13500 | 20888104 |  |  |
| M15-M19,M47 | ther | U | COL2-3/4C (long) epitope | P02458 | 17538566 |  |  | Q61 | diag | U | β-2 microglobulin (B2M) | P61769 | 20888104 |  |  |
| M15-M19,M47 | ther | U | COL2-3/4C (short) epitope | P02458 | 17538566 |  |  | Q61 | diag | U | neutrophil gelatinase-associated lipocalin (NGAL) | P80188 | 20888104 |  |  |
| M15-M19,M47 | ther | U | C-terminal cross- linked telopeptide of type II collagen (CTX-II) |  | 17538566 |  |  | Q61 | diag | U | kidney injury molecule-1 (KIM-1) | Q96D42 | 20888104 |  |  |
| M15-M19,M47 | ther | U | glucosyl–galactosyl–pyridinoline (Glc-Gal-PYD) |  | 17538566 |  |  | Q61 | prog | U | sodium |  | 22846584 |  |  |
| M17 | diag | U | C-telopeptide fragments of type II collagen (CTX-II) | Q16299 | 22582493 |  |  | Q62.0 | diag | U | monocyte chemotactic protein-1 (MCP-1) | P13500 | 22744767 | ~0.85 | ~0.9 |
| M30.3 | diag | U | CHL1, L1cell adhesion molecule | O00533 | 23281308 |  |  | Q62.11 | diag | U | Mannan-binding lectin serine peptidase 2 | O00187 | 20639044 |  |  |
| M30.3 | diag | U | FLNB, Filamin B | O75369 | 23281308 |  |  | Q62.11 | diag | U | Serpin peptidase inhibitor, clade A (alpha1-antiproteinase, antitrypsin), member 1 | P01009 | 20639044 |  |  |
| M30.3 | diag | U | THBD, Thrombomodulin | P07204 | 23281308 |  |  | Q62.11 | diag | U | Angiotensinogen | P01019 | 20639044 |  |  |
| M30.3 | diag | U | IL6ST, Interleukin 6 receptor subunit beta | P40189 | 23281308 |  |  | Q62.11 | diag | U | Epidermal growth factor (beta-urogastrone) | P01133 | 20639044 |  |  |
| M30.3 | diag | U | DSG2, Desmoglein-2 | Q14126 | 23281308 |  |  | Q62.11 | diag | U | Fibronectin 1 | P02751 | 20639044 |  |  |
| M30.3 | diag | U | FLNC, filamin C | Q14315 | 23281308 | ~92% | ~95% | Q62.11 | diag | U | alpha-1-Microglobulin/bikunin precursor | P02760 | 20639044 |  |  |
| M30.3 | diag | U | LTBP2, Latent transforming growth factor beta binding protein 2 | Q14767 | 23281308 |  |  | Q62.11 | diag | U | Albumin | P02768 | 20639044 |  |  |
| M30.3 | diag | U | MEP1A, meprin A | Q16819 | 23281308 | ~93% | ~94% | Q62.11 | diag | U | Transferrin | P02787 | 20639044 |  |  |
| M30.3 | diag | U | OBSCN, Obsurin | Q5VST9 | 23281308 |  |  | Q62.11 | diag | U | Lactotransferrin | P02788 | 20639044 |  |  |
| M30.3 | diag | U | CSMD3, CUB and sushi domain-containing protein 3 | Q7Z407 | 23281308 |  |  | Q62.11 | diag | U | Glyceraldehyde-3-phosphate dehydrogenase | P04406 | 20639044 |  |  |
| M30.3 | diag | U | TTN, Titin | Q8WZ42 | 23281308 |  |  | Q62.11 | diag | U | Heat shock 27kDa protein 1 | P04792 | 20639044 |  |  |
| M30.3 | diag | U | PVRL4, Nectin-4 cell adhesion molecular | Q96NY8 | 23281308 |  |  | Q62.11 | diag | U | Apolipoprotein D | P05090 | 20639044 |  |  |
| M30.3 | diag | U | ABCB9, TAP-Like ABC transporter | Q9NP78 | 23281308 |  |  | Q62.11 | diag | U | S100 calcium binding protein A8 | P05109 | 20639044 |  |  |
| M30.3 | diag | U | TLN1, Talin | Q9Y490 | 23281308 |  |  | Q62.11 | diag | U | Myeloperoxidase | P05164 | 20639044 |  |  |
| M32 | diag | U | sICAM-1 | P05362 | 22324942 |  |  | Q62.11 | diag | U | Keratin 8 | P05787 | 20639044 |  |  |
| M32 | diag | U | Monocyte chemoattractant protein 1 (MCP-1) | P13500 | 22857869 |  |  | Q62.11 | diag | U | Gelsolin (amyloidosis, Finnish type) | P06396 | 20639044 |  |  |
| M32 | diag | U | sVCAM-1 | P19320 | 22324942 |  |  | Q62.11 | diag | U | S100 calcium binding protein A9 | P06702 | 20639044 |  |  |
| M32 | prog | U | Neutrophil gelatinase-associated lipocalin | P80188 | 22500106 | ~70% | ~89% | Q62.11 | diag | U | S100 calcium binding protein A6 | P06703 | 20639044 |  |  |
| M32.1, N08.5 | diag | U | Tumor necrosis factor-like weak inducer of apoptosis(TWEAK) | A9Q749 | 22719208 |  |  | Q62.11 | diag | U | Annexin A2 | P07335 | 20639044 |  |  |
| M32.1, N08.5 | diag | U | ICAM-1 | P05362 | 20714774 |  |  | Q62.11 | diag | U | Heat shock protein 90kDa alpha(cytosolic), class A member 1 | P07900 | 20639044 |  |  |
| M32.1, N08.5 | diag | U | intercellular adhesion molecule-1 (ICAM-1) | P05362 | 22685016 |  |  | Q62.11 | diag | U | CD14 molecule | P08571 | 20639044 |  |  |
| M32.1, N08.5 | diag | U | Monocyte Chemoattractant Protein-1(MCP-1) | P13500 | 23653850 | 88.5% | 46.3% | Q62.11 | diag | U | Glutathione S-transferase pi 1 | P09211 | 20639044 |  |  |
| M32.1, N08.5 | diag | U | VCAM-1 | P19320 | 20714774 |  |  | Q62.11 | diag | U | Lectin, galactoside-binding, soluble, 3 binding protein | P09382 | 20639044 |  |  |
| M32.1, N08.5 | diag | U | Prostaglandin H2 D-isomerase | P41222 | 22498882 | 78.0% | 73.0% | Q62.11 | diag | U | Secreted phosphoprotein 1 | P10451 | 20639044 |  |  |
| M32.1, N08.5 | diag | U | miR-221 |  | 22685016 |  |  | Q62.11 | diag | U | Cadherin 1, type 1, E-cadherin (epithelial) | P12830 | 20639044 |  |  |
| M32.1, N08.5 | diag | U | miR-222 |  | 22685016 |  |  | Q62.11 | diag | U | Myosin, heavy chain 7, cardiac muscle,beta | P12883 | 20639044 |  |  |
| M32.1, N08.5 | diag | U | monocytic chemoattractant protein-1 (UMCP-1) |  | 22759858 |  |  | Q62.11 | diag | U | Macrophage migration inhibitory factor (glycosylation-inhibiting factor) | P14174 | 20639044 |  |  |
| M32.1, N08.5 | moni | U | liver-type fatty acid-binding protein (L-FABP) | P07148 | 21993584 | 65.0% | 83.0% | Q62.11 | diag | U | Pyruvate kinase, muscle | P14618 | 20639044 |  |  |
| M32.1, N08.5 | moni | U | Monocyte chemoattractant protein 1(MCP-1) | P13500 | 21993584 | 83.0% | 85.0% | Q62.11 | diag | U | Matrix metallopeptidase 9 (gelatinase B, 92kDa gelatinase, 92kDa type IV collagenase) | P14780 | 20639044 |  |  |
| M32.1, N08.5 | moni | U | Monocyte chemoattractant protein 1(MCP-1); Hepcidin; liver-type fatty acid-binding protein (L-FABP) | P13500; P81172; P07148 | 21993584 | 100.0% | 81.0% | Q62.11 | diag | U | CD44 molecule (Indian blood group) | P16070 | 20639044 |  |  |
| M32.1, N08.5 | moni | U | Hepcidin | P81172 | 21993584 | 83.0% | 60.0% | Q62.11 | diag | U | Cadherin 2, type 1, N-cadherin (neuronal) | P19022 | 20639044 |  |  |
| M32.1, N08.5 | prog | U | Monocyte chemotactic protein (MCP-1) | P13500 | 22942263 | 50.0% | 90.0% | Q62.11 | diag | U | S100 calcium binding protein A7 | P31151 | 20639044 |  |  |
| M32.1, N08.5 | prog | U | monocytic chemoattractant protein-1 (UMCP-1) |  | 22759858 |  |  | Q62.11 | diag | U | Keratin 9 | P35527 | 20639044 |  |  |
| M32.10 | prog | U | Transforming growth factor beta1 | P01137 | 21563925 |  |  | Q62.11 | diag | U | Cadherin 11, type 2, OB-cadherin (osteoblast) | P55287 | 20639044 |  |  |
| N00.0,N01.0,N02.0,N03.0,N04.0,N05.0,N06.0,N07.0,N08.0 | diag | U | CD80 (B7.1) | P33681 | 23013941 |  |  | Q62.11 | diag | U | Triosephosphate isomerase 1 | P60174 | 20639044 |  |  |
| N00.1,N01.1,N02.1,N03.1,N04.1,N05.1,N06.1,N07.1 | diag | U | monocyte chemoattractant protein-1 | P13500 | 21034351 |  |  | Q62.11 | diag | U | beta-2-Microglobulin | P61769 | 20639044 |  |  |
| N00.7,N01.7,N02.7,N03.7,N04.7,N05.7,N06.7,N07.7 | diag | U | Fibroblast-specific protein 1 (FSP1) |  | 22095943 | 91.7% | 90.2% | S06 | prog | U | S100B protein | P04271 | 23031665 | 90.0% | 62.8% |
| N02.2 | prog | U | N-Acetyl-β-D-glucosamindase (NAG) | CID439454 | 23013941; 12401843 | 77.0% |  | T86 | moni | Br | carbonyl sulfide |  | 22796631 |  |  |
| N02.2 | prog | U | μ2-microglobulin | P61769 | 23013941 |  |  | T86.1 | diag | U | IP-10 | P02778 | 22914685 | 63-100% | 78-83% |
| N02.8 | diag | U | cGMP-dependent 3,5-cyclic phosphodiesterase | O00408 | 16372274 |  |  | T86.1 | diag | U | HLA-DR | P04233 | 22914685 | 80.0% | 98.0% |
| N02.8 | diag | U | Phosphomannomutase 2 | O15305 | 16372274 |  |  | T86.1 | diag | U | IL-6 | P05231 | 22914685 | 92.0% | 63.0% |
| N02.8 | diag | U | Zinc finger protein 324 | O75467 | 16372274 |  |  | T86.1 | diag | U | vascular endothelial growth factor (VEGF) | P15692,P49765,P49767,O43915 | 22914685 | 85.0% | 75.0% |
| N02.8 | diag | U | NADH-ubiquinone oxidore-ductase 30 kDa subunit | O75489 | 16372274 |  |  | T86.1 | diag | U | CXCR3 | P49682 | 22914685 | 63-100% | 78-83% |
| N02.8 | diag | U | GRB2-related adaptor protein 2 (Gads) | O75791 | 16372274 |  |  | T86.1 | diag | U | Peptide/proteome panels |  | 22914685 | 90-91% | 77-83% |
| N02.8 | diag | U | ceruloplasmin | P00450 | 21595033 |  |  | T86.1 | prog | U | Tim-3 | Q8TDQ0 | 22914685 | 84-87% | 95-96% |
