# Supplementary material for: The Assessment of the Readiness of Molecular Biomarker-Based Mobile Health Technologies for Healthcare Applications
Source: Sci Rep. 2015 Dec 8;5:17854. doi: 10.1038/srep17854 (PMC4672303; doi:10.1038/srep17854)
Supplement: Supplementary Table S4 [file srep17854-s4.doc]

Supplementary Table S4: List of molecular biomarkers from blood.

| Biomarker | Uniprot ID | Disease | ICD10 | Status | Biomarker type | Source | Reference |
| --- | --- | --- | --- | --- | --- | --- | --- |
| MR-proadrenomedullin |  | sepsis | A40,A41 |  | prognostic |  | 21440204 |
| albumin (ALB) | P43652 | acute graft-versus-host disease (aGVHD);sepsis | A40-A41,D89.8 |  | diagnostic | plasma | 23165480 |
| Leukotriene A4 hydrolase | P09960 | acute graft-versus-host disease (aGVHD);sepsis | A40-A41,D89.8 |  | diagnostic | plasma | 23165480 |
| phosphate |  | HIV Infection | B20 |  | monitoring; theragnostic | serum; tubule | 22716111 |
| sand fly saliva antibodies |  | visceral leishmaniasis | B55.0 | clinical trial | marker | serum | 21931871 |
| sVCAM | P19320 | solid tumors treated with Angiozyme | C00-C75,C7A,C7B | clinical trial | marker | serum | 194 |
| vascular endothelial growth factor (VEGF) | P15692,P49765,P49767,O43915 | advanced solid tumor | C00-C75,C7A,C7B |  | surrogate | plasma; urine | 234 |
| vascular endothelial growth factor (VEGF) | P15692,P49765,P49767,O43915 | solid tumors treated with Angiozyme | C00-C75,C7A,C7B | clinical trial | marker | serum | 194 |
| von Willebrand factor antigen (vWf) | P04275 | solid tumors treated with Angiozyme | C00-C75,C7A,C7B | clinical trial | surrogate | serum | 194 |
| IL-1Rα | P14778 | cancer | C00-C96 |  | diagnostic | plasma | 22732129 |
| IL-6 | P05231 | cancer | C00-C96 |  | diagnostic | plasma | 22732129 |
| TNF-alpha | P01375 | advanced cancer | C00-C96 |  | diagnostic | plasma | 22732129 |
| Procalcitonin (PCT) |  | Cancer patients with febrile neutropenia (FN) | C00-C96,D70 |  | diagnostic; prognostic |  | 246 |
| CA 19.9 | Q969X2 | metastatic or recurrent gastric cancer | C16 |  | prognostic | serum | 620 |
| CA 72.4 |  | curatively resected advanced gastric cancer | C16 |  | prognostic | serum | 524 |
| CA 72.4 |  | gastric cancer | C16 |  | prognostic; theragnostic | serum | 623 |
| carcinoembryonic antigen (CEA) | P06731 | curatively resected advanced gastric cancer | C16 |  | prognostic | serum | 524 |
| preoperative plasma levels of CEA | P06731 | gastric cancer | C16 |  | prognostic | plasma | 27 |
| CA 19.9 | Q969X2 | Dukes'' B or C colorectal cancer | C18-C21 |  | marker | serum | 220 |
| carcinoembryonic antigen (CEA) | P06731 | Dukes'' B or C colorectal cancer | C18-C21 |  | marker | serum | 220 |
| CEA-TPA | P06731,P00750 | operated colorectal cancer | C18-C21 |  | marker | serum | 342 |
| CEA-TPA-CA19.9-CA72.4 | P06731,P00750 | operated colorectal cancer | C18-C21 |  | marker | serum | 342 |
| Cytokeratin 18 (CK18) | P05783 | metastatic colorectal cancer (mCRC) | C18-C21 |  | prognostic; theragnostic | plasma | 549 |
| DcR3 (DD-C248) | O95407 | colon cancer | C18-C21 |  | diagnostic | serum | 419 |
| glycodelin | P09466 | metastatic colorectal cancer | C18-C21 | clinical trial | marker | serum | 386 |
| IGF-binding protein-3 (IGFBP-3) | P17936 | metastatic colorectal cancer | C18-C21 | clinical trial | prognostic; theragnostic | plasma | 19073970 |
| MASP-2 | O00187 | Dukes''B or Dukes''C colon cancer | C18-C21 |  | classification; theragnostic | serum; plasma | 357 |
| metalloproteinases 1 (TIMP-1) | P01033 | advanced colorectal cancer | C18-C21 | clinical trial | monitoring | plasma | 417 |
| p53 antigen | P04637 | colon cancer | C18-C21 |  | detective | serum | 49 |
| pro-enzyme form of human collagenase-1 (ProMMP-1) | P03956 | Colon Cancer | C18-C21 |  | marker | serum | 108 |
| Reg IV (DD-C101) | Q9BYZ8 | colon cancer | C18-C21 |  | diagnostic | serum | 419 |
| Spondin-2 (DD-P108) | Q9BUD6 | colon cancer | C18-C21 |  | diagnostic | serum | 419 |
| TRAIL-R2 | O14763 | colon cancer | C18-C21 |  | diagnostic | serum | 419 |
| tumor type M2 pyruvate kinase (TuM2-PK) | P14618 | advanced colorectal cancer | C18-C21 | clinical trial | monitoring | plasma | 417 |
| vascular endothelial growth factor (VEGF) | P15692,P49765,P49767,O43915 | Colorectal Carcinoma | C18-C21 |  | prognostic | serum | 85 |
| tissue inhibitor of metalloproteinase (TIMP 1) |  | Dukes''B or Dukes''C colon cancer | C18-C21 |  | classification; theragnostic | serum; plasma | 357 |
| Carboxyethylpyrrole (CEP) |  | metastatic colorectal cancer (mCRC) | C18-C21 |  | prognostic |  | 600 |
| sialyl Lewis a (sLea) |  | colorectal cancer (CCR) | C18-C21 |  | prognostic |  | 592 |
| sialyl Lewis x (sLex) |  | colorectal cancer (CCR) | C18-C21 |  | prognostic |  | 592 |
| D-dimer |  | metastatic colorectal cancer | C18-C21 |  | prognostic |  | 568 |
| urokinase-type plasminogen activator (uPA) | P00749 | BREAST CANCER, COLON CANCER | C18-C21,C50 |  | marker | plasma | 67 |
| PlGF | P49763 | rectal cancer (RC) | C20 | clinical trial | prognostic | plasma | 480 |
| vascular endothelial growth factor (VEGF) | P15692,P49765,P49767,O43915 | rectal cancer (RC) | C20 | clinical trial | prognostic | plasma | 480 |
| alpha-fetoprotein (AFP) | P02771 | hepatocellular carcinoma | C22.0 | phase III | prognostic; monitoring; theragnostic | serum | 19064965 |
| glypican 3 (GPC3) |  | hepatocellular cancer (HCC) | C22.0 |  | detective |  | 450 |
| CA 19.9 | Q969X2 | advanced pancreatic cancer | C25 | clinical trial | surrogate; monitoring; theragnostic | serum | 171 |
| Cytokeratin 18 (CK18) | P05783 | pancreatic cancer | C25 |  | prognostic | plasma | 488 |
| regenerating islet-derived 4 (REG4) | Q9BYZ8 | Pancreatic cancer (PC) | C25 |  | diagnostic | serum | 466 |
| ACE | P12821 | stage I-III NSCLC | C33,C34 | clinical trial | prognostic | serum | 378 |
| bFGF | P09038 | stage I non-small cell lung cancer | C33,C34 |  | prognostic | serum | 305 |
| CA 125 | Q8WXI7 | non-small cell lung cancer | C33,C34 |  | prognostic; theragnostic | serum | 51 |
| carcinoembryonic antigen (CEA) | P06731 | non-small cell lung cancer | C33,C34 |  | prognostic; theragnostic | serum | 51 |
| CYFRA |  | non-small cell lung cancer | C33,C34 |  | prognostic; theragnostic | serum | 51 |
| IL-10 | P22301 | non-small-cell lung cancer with concurrent chemoradiation | C33,C34 |  | diagnostic | plasma | 22732129 |
| IL-6 | P05231 | non-small-cell lung cancer with concurrent chemoradiation | C33,C34 |  | diagnostic | plasma | 22732129 |
| NSE | Q96KN4 | non-small cell lung cancer | C33,C34 |  | prognostic; theragnostic | serum | 51 |
| Osteopontin (OPN) | P10451 | advanced non-small cell lung cancer (NSCLC) | C33,C34 | clinical trial | prognostic | plasma | 244 |
| plasminogen activator inhibitor 1 (PAI-1) | P05121 | advanced non-small cell lung cancer (NSCLC) | C33,C34 | clinical trial | prognostic | plasma | 244 |
| sTNF-R1 | P19438,P20333 | non-small-cell lung cancer with concurrent chemoradiation | C33,C34 |  | diagnostic | plasma | 22732129 |
| UPAR | Q03405 | stage I non-small cell lung cancer | C33,C34 |  | prognostic | serum | 305 |
| vascular endothelial growth factor (VEGF) | P15692,P49765,P49767,O43915 | advanced non-small cell lung cancer (NSCLC) | C33,C34 | clinical trial | prognostic | plasma | 244 |
| vascular endothelial growth factor (VEGF) | P15692,P49765,P49767,O43915 | stage I non-small cell lung cancer | C33,C34 |  | prognostic | serum | 305 |
| cross-linked carboxyterminal telopeptide of Type I collagen (ICTP) |  | Primary Lung Cancer | C33,C34 |  | marker | serum | 154 |
| antigen A |  | RESECTED STAGE I AND II NON-SMALL CELL LUNG CANCER | C33,C34 |  | prognostic |  | 64 |
| neuron specific enolase | P09104 | small cell lung cancer | C34.90 | phase III | prognostic | serum | 11 |
| Pro-gastrin-releasing peptide (31-98) |  | small cell lung cancer | C34.90 |  | diagnostic | serum | 12 |
| Melanoma-Inhibiting Activity (MIA) | Q16674 | Metastatic Melanoma | C43 | clinical trial | marker | serum | 102 |
| S100B | P04271 | Metastatic Melanoma | C43 | clinical trial | marker | serum | 102 |
| sIL-2R | P01589,P14784,P31785 | melanoma | C43 | clinical trial | prognostic | serum | 20454974 |
| TNF-alpha | P01375 | melanoma | C43 | clinical trial | prognostic | serum | 20454974 |
| YKL-40 | P36222 | metastatic malignant melanoma | C43 |  | prognostic | serum | 327 |
| β-2 microglobulin (B2M) | P61769 | melanoma | C43 | clinical trial | prognostic | serum | 20454974 |
| NKG2D ligand |  | metastatic melanoma | C43 | phase II | associative |  | 602 |
| NY-ESO-1 |  | response to anti-CTLA-4 (Ipimilumab) treatment | C43 |  | prognostic; theragnostic |  | 514 |
| Osteopontin (OPN) | P10451 | head and neck squamous cell carcinomas (HNSCC) | C44 |  | prognostic | serum | 174 |
| CA 125 | Q8WXI7 | Malignant Pleural Mesothelioma | C45 |  | prognostic | serum | 114 |
| carcinoembryonic antigen (CEA) | P06731 | Malignant Pleural Mesothelioma | C45 |  | marker | serum | 114 |
| CYFRA 21-1 |  | Malignant Pleural Mesothelioma | C45 |  | prognostic | serum | 114 |
| epidermal growth factor (EGF) | P01133 | malignant pleural mesothelioma | C45 |  | prognostic | serum | 445 |
| megakaryocyte potentiating factor (MPF) | Q13421 | malignant pleural mesothelioma | C45 |  | monitoring | serum | 649 |
| Osteopontin (OPN) | P10451 | malignant pleural mesothelioma | C45 |  | prognostic | plasma | 649 |
| platelet-derived growth factor (PDGF-AB) | P16234,P09619 | malignant pleural mesothelioma | C45 |  | prognostic | serum | 445 |
| Soluble mesothelin (SM) | Q13421 | malignant pleural mesothelioma | C45 |  | monitoring | serum | 649 |
| BONE SIALOPROTEIN (BSP) | P21815 | PRIMARY BREAST CANCER | C50 |  | marker | serum | 70 |
| CA 15.3 | P15941 | Metastatic Breast Cancer | C50 | clinical trial | prognostic; monitoring | serum | 159 |
| carcinoembryonic antigen (CEA) | P06731 | metastatic breast cancer patients treated with 1st-line hormone therapy | C50 |  | prognostic | serum | 215 |
| carcinoembryonic antigen (CEA) | P06731 | breast cancer | C50 |  | prognostic | serum | 394 |
| CEA-TPA-CA15.3 | P06731,P00750,P15941 | breast cancer | C50 |  | marker | serum | 268 |
| Colony-stimulating factor-1 (CSF-1) | P09603 | breast cancer | C50 |  | marker | serum | 560 |
| dynein |  | breast cancer | C50 |  | surrogate; theragnostic | peripheral blood | 537 |
| folliculin | Q8NFG4 | breast cancer | C50 |  | surrogate; theragnostic | peripheral blood | 537 |
| HER2/neu | P04626 | Metastatic Breast Cancer | C50 | clinical trial | prognostic | serum | 159 |
| HER2/neu | P04626 | metastatic breast cancer patients treated with 1st-line hormone therapy | C50 |  | prognostic | serum | 215 |
| IL-6 | P05231 | breast cancer | C50 |  | marker | serum | 90 |
| IL-8 | P10145 | metastatic breast cancer | C50 |  | prognostic | plasma | 572 |
| Krebs Von Den Lungen 6 (KL-6) | P15941 | Breast Cancer | C50 |  | marker | serum | 143 |
| Osteopontin (OPN) | P10451 | metastatic breast cancer | C50 |  | prognostic | plasma | 243 |
| Tartrate-resistant acid phosphatase 5b (TRAP 5b) | P13686 | breast carcinoma | C50 | clinical trial | surrogate | serum | 272 |
| TNF receptor associated protein 1 | Q12931 | breast cancer | C50 |  | surrogate; theragnostic | peripheral blood | 537 |
| trefoil factor 1 | P04155 | breast cancer | C50 |  | surrogate; theragnostic | peripheral blood | 537 |
| glucocorticoids |  | breast cancer survivors | C50 |  | diagnostic |  | 22732129 |
| N-terminal pro-B-type natriuretic peptide (NT-pro-BNP) |  | HER-2-positive metastatic breast cancer | C50 |  | prognostic |  | 289 |
| the amino-terminal procollagen propeptides of type I collagen (P1NP) |  | Advanced breast cancer | C50 | phase III | marker |  | 343 |
| C-terminal telopeptide of type I collagen in serum (S-CTX) |  | Advanced breast cancer | C50 | phase III | marker |  | 343 |
| vascular endothelial growth factor (VEGF) | P15692,P49765,P49767,O43915 | squamous cell carcinoma of the uterine cervix | C53 |  | prognostic | serum | 340 |
| VEGF-C | P49767 | squamous cell carcinoma of the uterine cervix | C53 |  | prognostic | serum | 340 |
| YKL-40 | P36222 | adenocarcinoma of the uterine cervix | C53 |  | diagnostic | serum | 456 |
| EGFR | P00533 | persistent or recurrent endometrial cancer | C54.1 | clinical trial | prognostic | serum | 554 |
| BETA 2-MICROGLOBULIN | P61769 | advanced epithelial ovarian cancer | C56 |  | prognostic; theragnostic | serum | 53 |
| CA 125 | Q8WXI7 | ovarian cancer | C56 | clinical trial | prognostic | serum | 150 |
| Cln101 |  | ovarian cancer | C56 |  | detective | serum | 254 |
| E-selectin | P16581 | ovarian cancer | C56 | clinical trial | surrogate | serum | 278 |
| Ovr110 |  | ovarian cancer | C56 |  | detective | serum | 254 |
| p55 | Q8N3R9 | ovarian cancer | C56 |  | prognostic | serum | 50 |
| p75 | P14317 | ovarian cancer | C56 |  | prognostic | serum | 50 |
| vascular cell adhesion molecule-1 (VCAM-1) | P19320 | ovarian cancer | C56 | clinical trial | surrogate | serum | 278 |
| YKL-40 | P36222 | Recurrent Ovarian Cancer | C56 |  | prognostic | plasma | 136 |
| CA 19-9 |  | epithelial ovarian cancer stage I and II | C56 |  | prognostic | serum | 30 |
| Serum Tumor Markers (STM) pre-RPLND |  | PN1, N2 and N3 Nonseminomatous Germ Cell Tumor (NSGCT) | C56, C62, D27, D29.2 |  | prognostic | serum | 74 |
| human chorionic gonadotrophin (hCG) | P01215,P01233 | placental tumor | C58 |  | marker | serum | 94 |
| Bone specific alkaline phosphatase (bALP) | P05186 | advanced prostate cancer | C61 |  | marker | serum | 216 |
| fPSA/tPSA | P07288 | prostate cancer | C61 |  | diagnostic | serum | 494 |
| IGF-binding protein-3 (IGFBP-3) | P17936 | Metastatic Prostate Cancer (PCA) | C61 |  | marker | serum | 144 |
| IL-6 | P05231 | ANDROGEN-INDEPENDENT PROSTATE CANCER (AIPC) | C61 |  | prognostic | serum | 56 |
| matrix metalloproteinase 9 (MMP 9) | P14780 | metastatic hormone resistant prostatic cancer (HRPC) | C61 | clinical trial | marker | serum | 285 |
| osteocalcin (OC) | P02818 | prostate cancer | C61 | clinical trial | prognostic | serum | 636 |
| OVX1 |  | androgen-independent prostate cancer | C61 |  | prognostic | serum | 6 |
| P1NP |  | prostate cancer | C61 |  | associative | serum | 528 |
| PAP | P51003 | metastatic hormone resistant prostatic cancer (HRPC) | C61 | clinical trial | marker | serum | 285 |
| prostate-specific antigen (PSA) | P07288 | prostate cancer | C61 | clinical trial | antecedent | serum | 18006214 |
| prostate-specific antigen (PSA) | P07288 | metastatic hormone resistant prostatic cancer (HRPC) | C61 | clinical trial | marker | serum | 285 |
| TRAP | P13686 | prostate cancer | C61 |  | associative | serum | 528 |
| amino-terminal procollagen propeptides of type 1 collagen (PINP) |  | prostate cancer | C61 |  | diagnostic | serum | 425 |
| Pyridinoline cross-linked carboxyterminal telopeptide of type I collagen (1CTP) |  | prostate cancer | C61 |  | associative | serum | 528 |
| procollagen amino-terminal propeptide 1 (P1NP) |  | prostate cancer (PC) | C61 |  | marker | serum | 564 |
| chromogranin A (CGA) |  | metastatic hormone resistant prostatic cancer (HRPC) | C61 | clinical trial | marker | serum | 285 |
| deoxy-pyridinoline (DPD) |  | hormone refractory prostate cancer (HRPC) | C61 | Phase II | prognostic |  | 316 |
| pyridinoline [PYD] |  | hormone refractory prostate cancer (HRPC) | C61 | Phase II | prognostic |  | 316 |
| N-telopeptide (NTX) |  | hormone refractory prostate cancer (HRPC) | C61 | Phase II | prognostic |  | 316 |
| procollagen amino-terminal propeptide 1 (P1NP) |  | hormone refractory prostate cancer (HRPC) | C61 | Phase II | prognostic |  | 316 |
| D-dimer |  | chemotherapy refractory prostate cancer (CRefracPC) | C61 |  | marker |  | 446 |
| N-telopeptide (NTX) |  | advanced prostate cancer | C61 |  | marker |  | 216 |
| N-terminal propeptide of type I collagen (NTX) |  | breast cancer; prostate cancer | C61,C50 |  | diagnostic | serum | 486 |
| N-terminal propeptide of type I collagen (NTX) |  | breast cancer; prostate cancer | C61,C50 |  | diagnostic | serum | 531 |
| peptide-bound collagen type I cross-links C-telopeptides (ICTP) |  | breast cancer; prostate cancer | C61,C50 |  | diagnostic | serum | 531 |
| peptide-bound collagen type I cross-links C-telopeptides (ICTP) |  | breast cancer; prostate cancer | C61,C50 |  | diagnostic | serum | 486 |
| deoxy-pyridinoline (DPD) |  | bone metastatic prostate cancer | C61,C79.51 |  | marker |  | 100 |
| deoxy-pyridinoline (DPD) |  | bone metastatic prostate cancer | C61,C79.51 |  | prognostic |  | 100 |
| C-reactive protein (CRP) | P02741 | testcular cancer, long term survivors | C62 |  | diagnostic | plasma | 22732129 |
| IL-1Rα | P14778 | testcular cancer, long term survivors | C62 |  | diagnostic | plasma | 22732129 |
| alpha-fetoprotein (AFP) | P02771 | non-seminomatous germ-cell tumors (NSGCT) | C62,D29.2 | clinical trial | prognostic | serum | 185 |
| alpha-fetoprotein (AFP) | P02771 | Primary mediastinal non-seminomatous germ cell tumors (PMNSGT) | C62,D29.2 |  | prognostic | serum | 501 |
| alpha-fetoprotein (AFP) | P02771 | primary mediastinal nonseminomatous germ cell tumor (PMNSGCT) | C62,D29.2 |  | prognostic | serum | 479 |
| human chorionic gonadotrophin (hCG) | P01215,P01233 | non-seminomatous germ-cell tumors (NSGCT) | C62,D29.2 | clinical trial | prognostic | serum | 185 |
| human chorionic gonadotrophin (hCG) | P01215,P01233 | Primary mediastinal non-seminomatous germ cell tumors (PMNSGT) | C62,D29.2 |  | prognostic | serum | 501 |
| human chorionic gonadotrophin (hCG) | P01215,P01233 | primary mediastinal nonseminomatous germ cell tumor (PMNSGCT) | C62,D29.2 |  | prognostic | serum | 479 |
| human chorionic gonadotrophin (hCG) | P01215,P01233 | clinical Stage A testicular nonseminomatous germ cell tumors (NSGCT) | C62,D29.2 |  | classification | serum | 20 |
| a-Fetoprotein (AFP) | P02771 | advanced seminoma | C62.10; C62.90 | Clinical Society Recommended | monitoring | serum | 21037873 |
| human chorionic gonadotrophin (hCG) | P01215,P01233 | advanced seminoma | C62.10; C62.90 | Clinical Society Recommended | monitoring | serum | 21037873 |
| matrix metalloproteinase 9 (MMP 9) | P14780 | metastatic renal cell carcinoma (MRCC) | C64 |  | prognostic | serum | 530 |
| TNF-alpha | P01375 | metastatic renal cell carcinoma (MRCC) | C64 |  | prognostic | serum | 530 |
| beta HCG | P01233 | advanced bladder cancer | C67 |  | prognostic; theragnostic | serum | 63 |
| CA 125 | Q8WXI7 | advanced bladder cancer | C67 |  | prognostic; theragnostic | serum | 63 |
| CA 19.9 | Q969X2 | advanced bladder cancer | C67 |  | prognostic; theragnostic | serum | 63 |
| carcinoembryonic antigen (CEA) | P06731 | advanced bladder cancer | C67 |  | prognostic; theragnostic | serum | 63 |
| Serum tissue polypeptide antigen (S-TPA) |  | bladder carcinoma | C67 |  | prognostic | serum | 47 |
| C-reactive protein (CRP) | P02741 | breast & prostate cancer with radiation | C67,C50 |  | diagnostic | plasma | 22732129 |
| IL-1Rα | P14778 | breast & prostate cancer with radiation | C67,C50 |  | diagnostic | plasma | 22732129 |
| pamidronate |  | breast and prostate cancer | C67,C50 |  | marker |  | 34 |
| IGFBP-2 | P18065 | Glioblastoma multiforme (GBM) | C71 |  | diagnostic | serum | 402 |
| matrix metalloproteinase 9 (MMP 9) | P14780 | GBM;anaplastic astrocytoma (AA) | C71 |  | monitoring | serum | 506 |
| urokinase-type plasminogen activator (uPA) | P00749 | recurrent malignant glioma | C71 |  | prognostic | serum | 562 |
| YKL-40 | P36222 | high-grade gliomas (HGG) | C71 |  | prognostic | serum | 506 |
| ferritin | P02792,P02794,Q8N4E7 | Patients >=18 months of age at diagnosis of INSS Stage 3 MYCN-NA NB | C74 |  | prognostic | serum | 472 |
| 5-hydroxytryptamine (5-HT) |  | carcinoid tumor | C75, E34.0 |  | marker |  | 59 |
| Serum bone-specific alkaline phosphatase (B-AP) | P05186 | Extent of Metastatic Bone Disease | C79.51 |  | marker | serum | 79 |
| peptide-bound collagen type I cross-links C-telopeptides (ICTP) |  | Extent of Metastatic Bone Disease | C79.51 |  | marker | serum | 79 |
| carboxyterminal telopeptide (ICTP) |  | bone metastases (BM) | C79.51 |  | prognostic | serum | 370 |
| N-telopeptide (NTX) |  | bone metastases | C79.51 |  | prognostic | serum; plasma; urine | 16 |
| deoxy-pyridinoline (DPD) |  | bone metastase | C79.51 | clinical trial | prognostic |  | 211 |
| pyridinoline (PYD) |  | bone metastase | C79.51 | clinical trial | prognostic |  | 211 |
| β-crosslaps |  | bone metastases | C79.51 | phase II | prognostic |  | 458 |
| N-telopeptide (NTX) |  | bone metastase | C79.51 | clinical trial | prognostic |  | 211 |
| N-telopeptide of type I collagen (NTX) |  | patients (pts) with bone metastase | C79.51 | clinical trial | marker |  | 437 |
| Procollagen-I-propeptide |  | bone metastases | C79.51 | phase II | prognostic |  | 458 |
| N-terminal propeptide of type I collagen (NTX) |  | lung cancer with bone metastasis | C79.51,C33-C34 |  | monitoring; theragnostic |  | 601 |
| Bone specific alkaline phosphatase (bALP) | P05186 | breast cancer with bone metastases | C79.51,C50 |  | associative | serum | 622 |
| MMP1 | P03956 | breast cancer patients (BC) with bone metastases (BM) | C79.51,C50 |  | prognostic | serum | 471 |
| peptide-bound collagen type I cross-links C-telopeptides (ICTP) |  | breast cancer patients (BC) with bone metastases (BM) | C79.51,C50 |  | prognostic | serum | 471 |
| cross-linked N-telopeptide of type I collagen (NTX) |  | breast cancer with bone metastases | C79.51,C50 |  | monitoring; theragnostic | serum | 582 |
| cortisol |  | ambulatory breast cancer patients with bone metastase | C79.51,C50 | clinical trial | marker | serum; urine | 304 |
| deoxy-pyridinoline (DPD) |  | ambulatory breast cancer patients with bone metastase | C79.51,C50 | clinical trial | marker | serum; urine | 304 |
| N-telopeptide (NTX) |  | ambulatory breast cancer patients with bone metastase | C79.51,C50 | clinical trial | marker | serum; urine | 304 |
| a cross-linked, beta-aspartate-isomerized form of the epitope EKAHDGGR derived from the carboxyterminal telopeptide region of type I collagen alpha(1) chain |  | ambulatory breast cancer patients with bone metastase | C79.51,C50 | clinical trial | marker | serum; urine | 304 |
| vascular endothelial growth factor (VEGF) | P15692,P49765,P49767,O43915 | Hodgkin''s diseas | C81 |  | prognostic | serum | 399 |
| Soluble interleukin-2 receptor | P01589,P14784 | malignant lymphomas | C81-C96 |  | prognostic | serum | 18 |
| Immunoglobulin free light chain | P01708 | Waldenstrom''s macroglobulinemia | C88.0 | clinical trial | prognostic; theragnostic | serum | 478 |
| CA 15.3 | P15941 | multiple myeloma (MM) | C90.0 | clinical trial | prognostic | serum | 166 |
| DPD | Q12882 | myeloma bone disease | C90.0 |  | diagnostic | serum;urine | 330 |
| Hepatocyte Growth Factor (HGF) | P14210 | multiple myeloma | C90.0 |  | associative | peripheral blood; bone marrow | 281 |
| Osteopontin (OPN) | P10451 | multiple myeloma | C90.0 |  | prognostic | serum | 499 |
| a cross-linked, beta-aspartate-isomerized form of the epitope EKAHDGGR derived from the carboxyterminal telopeptide region of type I collagen alpha(1) chain |  | myeloma bone disease | C90.0 |  | diagnostic | serum;urine | 330 |
| Cardiac Troponin T | P45379 | Childhood Acute Lymphoblastic Leukemia | C91.0 |  | marker | serum | 73 |
| N-terminal pro-B-type natriuretic peptide (NT-pro-BNP) |  | children with Acute Lymphoblastic Leukemia (ALL) | C91.0 |  | diagnostic |  | 598 |
| serum iron |  | Iron deficiency caused anemia | D50 |  | marker | serum | 23687454 |
| albumin (ALB) | P43652 | graft-versus-host disease (GVHD) | D89.8 |  | prognostic | plasma | 23165480 |
| albumin (ALB) | P43652 | acute graft-versus-host disease (aGVHD) | D89.8 |  | diagnostic | plasma; serum | 22927351 |
| albumin (ALB) | P43652 | acute graft-versus-host disease (aGVHD) | D89.8 |  | monitoring | plasma; serum | 22927351 |
| albumin (ALB) | P43652 | acute graft-versus-host disease (aGVHD) | D89.8 |  | diagnostic | plasma; serum | 22927351 |
| CCL5 | P13501 | acute graft-versus-host disease (aGVHD) | D89.8 |  | diagnostic | plasma; serum | 22927351 |
| CCL8 | P80075 | acute graft-versus-host disease (aGVHD) | D89.8 |  | diagnostic | plasma; serum | 22927351 |
| CCL8 | P80075 | acute graft-versus-host disease (aGVHD) | D89.8 |  | antecedent | plasma; serum | 22927351 |
| CCL8 | P80075 | acute graft-versus-host disease (aGVHD) | D89.8 |  | prognostic | plasma; serum | 22927351 |
| CK18Fs | P05783 | acute graft-versus-host disease (aGVHD) | D89.8 |  | diagnostic | plasma; serum | 22927351 |
| CK18Fs | P05783 | acute graft-versus-host disease (aGVHD) | D89.8 |  | diagnostic | plasma; serum | 22927351 |
| Collagen α-1 | P20908 | acute graft-versus-host disease (aGVHD) | D89.8 |  | diagnostic | plasma | 23165480 |
| C-reactive protein (CRP) | P02741 | graft-versus-host disease (GVHD) | D89.8 |  | prognostic | plasma | 23165480 |
| CXCL10 | P02778 | acute graft-versus-host disease (aGVHD) | D89.8 |  | diagnostic | plasma | 23165480 |
| CXCL10 | P02778 | acute graft-versus-host disease (aGVHD) | D89.8 |  | diagnostic | plasma; serum | 22927351 |
| CXCL10 TNFR1 | P02778,P19438 | acute graft-versus-host disease (aGVHD) | D89.8 |  | diagnostic | plasma; serum | 22927351 |
| Elafin | P19957 | acute graft-versus-host disease (aGVHD) | D89.8 |  | diagnostic | plasma; serum | 22927351 |
| Elafin | P19957 | acute graft-versus-host disease (aGVHD) | D89.8 |  | monitoring | plasma; serum | 22927351 |
| Elafin | P19957 | acute graft-versus-host disease (aGVHD) | D89.8 |  | diagnostic | plasma; serum | 22927351 |
| GRO-α | P09341 | acute graft-versus-host disease (aGVHD) | D89.8 |  | diagnostic | plasma; serum | 22927351 |
| GRO-α | P09341 | acute graft-versus-host disease (aGVHD) | D89.8 |  | diagnostic | plasma; serum | 22927351 |
| HGF | P14210 | acute graft-versus-host disease (aGVHD) | D89.8 |  | prognostic | plasma | 23165480 |
| HGF | P14210 | acute graft-versus-host disease (aGVHD) | D89.8 |  | diagnostic | plasma | 23165480 |
| HGF | P14210 | acute graft-versus-host disease (aGVHD) | D89.8 |  | diagnostic | plasma | 23165480 |
| HGF | P14210 | graft-versus-host disease (GVHD) | D89.8 |  | prognostic | plasma | 23165480 |
| HGF | P14210 | acute graft-versus-host disease (aGVHD) | D89.8 |  | diagnostic | plasma; serum | 22927351 |
| HGF | P14210 | acute graft-versus-host disease (aGVHD) | D89.8 |  | diagnostic | plasma; serum | 22927351 |
| HGF | P14210 | acute graft-versus-host disease (aGVHD) | D89.8 |  | monitoring | plasma; serum | 22927351 |
| IFNγ | P01579 | acute graft-versus-host disease (aGVHD) | D89.8 |  | antecedent | plasma; serum | 22927351 |
| IFNγ | P01579 | acute graft-versus-host disease (aGVHD) | D89.8 |  | diagnostic | plasma; serum | 22927351 |
| IL-10 | P22301 | acute graft-versus-host disease (aGVHD) | D89.8 |  | diagnostic | plasma; serum | 22927351 |
| IL-10 | P22301 | acute graft-versus-host disease (aGVHD) | D89.8 |  | monitoring | plasma; serum | 22927351 |
| IL-10 | P22301 | aGVHD, chronic GVHD (cGVHD), infection, relapse | D89.8 |  | diagnostic | plasma | 23165480 |
| IL-12 | Q99665 | acute graft-versus-host disease (aGVHD) | D89.8 |  | diagnostic | plasma; serum | 22927351 |
| IL-12 | Q99665 | acute graft-versus-host disease (aGVHD) | D89.8 |  | diagnostic | plasma | 23165480 |
| IL-12 | Q99665 | acute graft-versus-host disease (aGVHD) | D89.8 |  | antecedent | plasma; serum | 22927351 |
| IL-12 | Q99665 | acute graft-versus-host disease (aGVHD) | D89.8 |  | prognostic | plasma; serum | 22927351 |
| IL-15 | P40933 | acute graft-versus-host disease (aGVHD) | D89.8 |  | antecedent | plasma; serum | 22927351 |
| IL-15 | P40933 | acute graft-versus-host disease (aGVHD) | D89.8 |  | monitoring | plasma; serum | 22927351 |
| IL-15 | P40933 | acute graft-versus-host disease (aGVHD) | D89.8 |  | diagnostic | plasma; serum | 22927351 |
| IL-18 | Q14116 | acute graft-versus-host disease (aGVHD) | D89.8 |  | diagnostic | plasma; serum | 22927351 |
| IL-18 | Q14116 | acute graft-versus-host disease (aGVHD) | D89.8 |  | monitoring | plasma; serum | 22927351 |
| IL-18 | Q14116 | acute graft-versus-host disease (aGVHD) | D89.8 |  | antecedent | plasma; serum | 22927351 |
| IL-18 | Q14116 | acute graft-versus-host disease (aGVHD) | D89.8 |  | prognostic | plasma; serum | 22927351 |
| IL-18 | Q14116 | acute graft-versus-host disease (aGVHD) | D89.8 |  | diagnostic | plasma | 23165480 |
| IL2R | P01589 | acute graft-versus-host disease (aGVHD) | D89.8 |  | antecedent | plasma; serum | 22927351 |
| IL2R | P01589 | acute graft-versus-host disease (aGVHD) | D89.8 |  | diagnostic | plasma; serum | 22927351 |
| IL2R | P01589 | acute graft-versus-host disease (aGVHD) | D89.8 |  | monitoring | plasma; serum | 22927351 |
| IL2R | P01589 | acute graft-versus-host disease (aGVHD) | D89.8 |  | diagnostic | plasma; serum | 22927351 |
| IL2R | P01589 | acute graft-versus-host disease (aGVHD) | D89.8 |  | prognostic | plasma; serum | 22927351 |
| IL-2Ra | P01589 | acute graft-versus-host disease (aGVHD) | D89.8 |  | prognostic | plasma | 23165480 |
| IL-2Ra | P01589 | acute graft-versus-host disease (aGVHD) | D89.8 |  | diagnostic | plasma | 23165480 |
| IL-6 | P05231 | graft-versus-host disease (GVHD) | D89.8 |  | prognostic | plasma | 23165480 |
| IL-8 | P10145 | acute graft-versus-host disease (aGVHD) | D89.8 |  | diagnostic | plasma; serum | 22927351 |
| IL-8 | P10145 | acute graft-versus-host disease (aGVHD) | D89.8 |  | prognostic | plasma | 23165480 |
| IL-8 | P10145 | acute graft-versus-host disease (aGVHD) | D89.8 |  | diagnostic | plasma | 23165480 |
| IL-8 | P10145 | graft-versus-host disease (GVHD) | D89.8 |  | prognostic | plasma | 23165480 |
| IL-8 | P10145 | acute graft-versus-host disease (aGVHD) | D89.8 |  | diagnostic | plasma; serum | 22927351 |
| M-CSF | P09603 | acute graft-versus-host disease (aGVHD) | D89.8 |  | monitoring | plasma; serum | 22927351 |
| M-CSF | P09603 | acute graft-versus-host disease (aGVHD) | D89.8 |  | diagnostic | plasma; serum | 22927351 |
| M-CSF | P09603 | acute graft-versus-host disease (aGVHD) | D89.8 |  | antecedent | plasma; serum | 22927351 |
| REG3a | Q06141 | graft-versus-host disease (GVHD) | D89.8 |  | differentiation | plasma | 23165480 |
| REG3a | Q06141 | graft-versus-host disease (GVHD) | D89.8 |  | prognostic | plasma | 23165480 |
| REG3a | Q06141 | GI GVHD | D89.8 |  | diagnostic | plasma | 23165480 |
| REG3a | Q06141 | graft-versus-host disease (GVHD) | D89.8 |  | prognostic; theragnostic | plasma | 23165480 |
| REG3a | Q06141 | acute graft-versus-host disease (aGVHD) | D89.8 |  | diagnostic | plasma; serum | 22927351 |
| REG3a | Q06141 | acute graft-versus-host disease (aGVHD) | D89.8 |  | diagnostic | plasma; serum | 22927351 |
| sc-kit | P10721 | acute graft-versus-host disease (aGVHD) | D89.8 |  | diagnostic | plasma; serum | 22927351 |
| sc-kit | P10721 | acute graft-versus-host disease (aGVHD) | D89.8 |  | diagnostic | plasma; serum | 22927351 |
| sFas |  | acute graft-versus-host disease (aGVHD) | D89.8 |  | diagnostic | plasma; serum | 22927351 |
| sFas |  | acute graft-versus-host disease (aGVHD) | D89.8 |  | antecedent | plasma; serum | 22927351 |
| sICAM-1 | P05362 | acute graft-versus-host disease (aGVHD) | D89.8 |  | diagnostic | plasma; serum | 22927351 |
| Syndecan-1 | P18827 | acute graft-versus-host disease (aGVHD) | D89.8 |  | diagnostic | plasma | 23165480 |
| Syndecan-I | P18827 | acute graft-versus-host disease (aGVHD) | D89.8 |  | diagnostic | plasma; serum | 22927351 |
| Syndecan-I | P18827 | acute graft-versus-host disease (aGVHD) | D89.8 |  | monitoring | plasma; serum | 22927351 |
| TGF-β1 | P01137 | acute graft-versus-host disease (aGVHD) | D89.8 |  | diagnostic | plasma; serum | 22927351 |
| TGF-β1 | P01137 | acute graft-versus-host disease (aGVHD) | D89.8 |  | diagnostic | plasma; serum | 22927351 |
| TNF-alpha | P01375 | acute graft-versus-host disease (aGVHD) | D89.8 |  | diagnostic | plasma; serum | 22927351 |
| TNF-alpha | P01375 | graft-versus-host disease (GVHD) | D89.8 |  | prognostic | plasma | 23165480 |
| TNF-alpha | P01375 | acute graft-versus-host disease (aGVHD) | D89.8 |  | diagnostic | plasma; serum | 22927351 |
| TNFR1 | P19438 | acute graft-versus-host disease (aGVHD) | D89.8 |  | diagnostic | plasma; serum | 22927351 |
| TNFR1 | P19438 | acute graft-versus-host disease (aGVHD) | D89.8 |  | monitoring | plasma; serum | 22927351 |
| TNFR1 | P19438 | acute graft-versus-host disease (aGVHD) | D89.8 |  | diagnostic | plasma | 23165480 |
| TNFR1 | P19438 | graft-versus-host disease (GVHD) | D89.8 |  | prognostic | plasma | 23165480 |
| TNFR1 | P19438 | graft-versus-host disease (GVHD) | D89.8 |  | prognostic | plasma | 23165480 |
| TNFR1 | P19438 | acute graft-versus-host disease (aGVHD) | D89.8 |  | diagnostic | plasma; serum | 22927351 |
| TNFR1 | P19438 | acute graft-versus-host disease (aGVHD) | D89.8 |  | antecedent | plasma; serum | 22927351 |
| TNFR1 | P19438 | acute graft-versus-host disease (aGVHD) | D89.8 |  | prognostic | plasma; serum | 22927351 |
| TNFR2 | Q12933 | acute graft-versus-host disease (aGVHD) | D89.8 |  | diagnostic | plasma; serum | 22927351 |
| TNFR2 | Q12933 | acute graft-versus-host disease (aGVHD) | D89.8 |  | monitoring | plasma; serum | 22927351 |
|  |  | acute graft-versus-host disease (aGVHD) | D89.8 |  | diagnostic | plasma; serum | 22927351 |
| Adiponectin | Q15848 | Diabetic Nephropathy | E10.2, E11.2, E12.2, E13.2, E14.2 |  | diagnostic | urine; serum | 22698077 |
| fibroblast growth factor (FGF-23) | Q9GZV9 | Diabetic Nephropathy | E10.2, E11.2, E12.2, E13.2, E14.2 |  | prognostic | serum | 22698077 |
| a-Fetoprotein (AFP) | P02771 | testicular non-seminoma | E29.9 | Clinical Society Recommended | classification | serum | 21037873 |
| human chorionic gonadotrophin (hCG) | P01215,P01233 | testicular non-seminoma | E29.9 | Clinical Society Recommended | classification | serum | 21037873 |
| LDH | P07864 | testicular non-seminoma | E29.9 | Clinical Society Recommended | classification | serum | 21037873 |
| glycosaminoglycans |  | mucopolysaccharidoses (MPSs) | E76 |  | antecedent | serum; plasma | 22658917 |
| glycosaminoglycans |  | mucopolysaccharidoses (MPSs) | E76 |  | diagnostic | serum; plasma | 22658917 |
| glycosaminoglycans |  | mucopolysaccharidoses (MPSs) | E76 |  | prognostic; theragnostic | serum; plasma | 22658917 |
| oligosaccharides |  | mucopolysaccharidoses (MPSs) | E76 |  | diagnostic | urine; plasma | 22658917 |
| oligosaccharides |  | mucopolysaccharidoses (MPSs) | E76 |  | prognostic; theragnostic | urine; plasma | 22658917 |
| oligosaccharides |  | mucopolysaccharidoses (MPSs) | E76 |  | prognostic | urine; plasma | 22658917 |
| Bis(monoacylglycero)phosphate |  | mucopolysaccharidoses (MPSs) | E76 |  | antecedent |  | 22658917 |
| Ganglioside 2 (GM2) |  | mucopolysaccharidoses (MPSs) | E76 |  | prognostic; theragnostic |  | 22658917 |
| Ganglioside 2 (GM2) |  | mucopolysaccharidoses (MPSs) | E76 |  | prognostic |  | 22658917 |
| Ganglioside 3 (GM3) |  | mucopolysaccharidoses (MPSs) | E76 |  | prognostic; theragnostic |  | 22658917 |
| Ganglioside 3 (GM3) |  | mucopolysaccharidoses (MPSs) | E76 |  | prognostic |  | 22658917 |
| prolactin | P01236 | Schizophrenia | F20 |  | pharmacodynamic; theragnostic | plasma | 23129338 |
| amyloid peptide |  | schizophrenia | F20 |  | classification |  | 23129339 |
| glucose |  | schizophrenia | F20 |  | monitoring; theragnostic | blood | 23129340 |
| lipid |  | schizophrenia | F20 |  | monitoring; theragnostic | blood | 23129340 |
| brain derived neurotrophic factor (BDNF) | P23560 | Huntington''s Disease | G10, F02.2 |  | prognostic | serum | 21882408 |
| C-reactive protein (CRP) | P02741 | Huntington''s Disease | G10, F02.2 |  | marker | plasma | 21882408 |
| CC‐chemokine ligand 11 (CCL11) | P51671 | amyotrophic lateral sclerosis (ALS) | G12.2 |  | diagnostic | CSF; blood | 21989244 |
| CCL24 | O00175 | amyotrophic lateral sclerosis (ALS) | G12.2 |  | diagnostic | CSF; blood | 21989244 |
| CCL26 | Q9Y258 | amyotrophic lateral sclerosis (ALS) | G12.2 |  | diagnostic | CSF; blood | 21989244 |
| Complement factors C3 | P01024 | amyotrophic lateral sclerosis (ALS) | G12.2 |  | diagnostic | blood | 21989244 |
| IL-8 | P10145 | amyotrophic lateral sclerosis (ALS) | G12.2 |  | diagnostic | CSF; blood | 21989244 |
| monocyte chemoattractant protein 1 (MCP-1) | P13500 | amyotrophic lateral sclerosis (ALS) | G12.2 |  | diagnostic | CSF; blood | 21989244 |
| phosphorylated neurofilament heavy chain (pNfH) | P12036 | amyotrophic lateral sclerosis (ALS) | G12.2 |  | diagnostic | CSF; blood | 21989244 |
| 102 plasma proteins | Q969N4 | Parkinson''s disease | G20, F02.3 |  | monitoring | plasma | 22814541 |
| Ceruloplasmin | P00450 | Parkinson''s disease | G20, F02.3 |  | associative | blood | 23587062 |
| High sensitivity C-reactive protein (Hs-CRP) | P02741 | Parkinson''s disease | G20, F02.3 |  | associative | blood | 23587062 |
| IL-6 | P05231 | Parkinson''s disease | G20, F02.3 |  | associative | blood | 23587062 |
| S100B | P04271 | Parkinson''s disease | G20, F02.3 |  | associative | blood | 23587062 |
| Arginine |  | Parkinson''s disease | G20, F02.3 |  | associative | blood | 23587062 |
| cholesterol |  | Parkinson''s disease | G20, F02.3 |  | associative | blood | 23587062 |
| Coenzyme Q10 |  | Parkinson''s disease | G20, F02.3 |  | associative | blood | 23587062 |
| Copper |  | Parkinson''s disease | G20, F02.3 |  | associative | blood | 23587062 |
| F2 isoprostanes |  | Parkinson''s disease | G20, F02.3 |  | associative | blood | 23587062 |
| glycine |  | Parkinson''s disease | G20, F02.3 |  | associative | blood | 23587062 |
| Homocysteine |  | Parkinson''s disease | G20, F02.3 |  | associative | blood | 23587062 |
| Hydroxyeicosatetraenoic acid products (HETEs) |  | Parkinson''s disease | G20, F02.3 |  | associative | blood | 23587062 |
| Leucocyte 8-hydroxy-2-deoxygyanosine (8-OHdG) |  | Parkinson''s disease | G20, F02.3 |  | associative | blood | 23587062 |
| levodopa |  | Parkinson''s disease | G20, F02.3 |  | associative | blood | 23587062 |
| Malondialdehyde (MDA) |  | Parkinson''s disease | G20, F02.3 |  | associative | blood | 23587062 |
| Nitrate |  | Parkinson''s disease | G20, F02.3 |  | associative | blood | 23587062 |
| Non-Ceruloplasmin bound Copper (NCBC) |  | Parkinson''s disease | G20, F02.3 |  | associative | blood | 23587062 |
| HMPAO |  | Parkinson''s disease | G20, F02.3 |  | associative |  | 23587062 |
| N-isopropyl-P[123I]-iodoamphetamine ([123I]IMP) |  | Parkinson''s disease | G20, F02.3 |  | associative |  | 23587062 |
| chemokine C–C motif ligand 2 (CCL2; monocyte chemotactic protein-1; MCP-1 ) | P13500 | Alzheimer''s disease | G30, F00 |  | diagnostic | CSF; plasma | 23631871 |
| chemokine | O00590 | Frontotemporal lobar degeneration (FTLD) | G31.0 |  | diagnostic | CSF; blood | 22527778 |
| cytokines |  | Frontotemporal lobar degeneration (FTLD) | G31.0 |  | diagnostic | CSF; blood | 22527778 |
| albumin (ALB) | P43652 | neuromyelitis optica (NMO) | G36.0 |  | diagnostic | serum | 22570066 |
| IgG |  | neuromyelitis optica (NMO) | G36.0 |  | diagnostic | serum | 22570066 |
| NMO-IgG |  | neuromyelitis optica (NMO) | G36.0 |  | diagnostic | serum | 22570066 |
| sTNF-R1 | P19438,P20333 | Obstructive sleep apnea | G47.3 |  | diagnostic | plasma | 22732129 |
| dipeptidyl peptidase IV | P27487 | chronic fatigue syndrome/myalgic encephalomyelitis (CFS/ME) | G93.3 |  | diagnostic | plasma | 22732129 |
| IFNγ | P01579 | chronic fatigue syndrome/myalgic encephalomyelitis (CFS/ME) | G93.3 |  | diagnostic | plasma | 22732129 |
| IL-12 | Q99665 | chronic fatigue syndrome/myalgic encephalomyelitis (CFS/ME) | G93.3 |  | diagnostic | plasma | 22732129 |
| IL-13 | P35225 | chronic fatigue syndrome/myalgic encephalomyelitis (CFS/ME) | G93.3 |  | diagnostic | plasma | 22732129 |
| IL-15 | P40933 | chronic fatigue syndrome/myalgic encephalomyelitis (CFS/ME) | G93.3 |  | diagnostic | plasma | 22732129 |
| IL-17 | Q16552 | chronic fatigue syndrome/myalgic encephalomyelitis (CFS/ME) | G93.3 |  | diagnostic | plasma | 22732129 |
| IL-1a | P01583 | chronic fatigue syndrome/myalgic encephalomyelitis (CFS/ME) | G93.3 |  | diagnostic | plasma | 22732129 |
| IL-1β | P01584 | chronic fatigue syndrome/myalgic encephalomyelitis (CFS/ME) | G93.3 |  | diagnostic | plasma | 22732129 |
| IL-2 | P60568 | chronic fatigue syndrome/myalgic encephalomyelitis (CFS/ME) | G93.3 |  | diagnostic | plasma | 22732129 |
| IL-23 | Q9NPF7 | chronic fatigue syndrome/myalgic encephalomyelitis (CFS/ME) | G93.3 |  | diagnostic | plasma | 22732129 |
| IL-4 | P05112 | chronic fatigue syndrome/myalgic encephalomyelitis (CFS/ME) | G93.3 |  | diagnostic | plasma | 22732129 |
| IL-5 | P05113 | chronic fatigue syndrome/myalgic encephalomyelitis (CFS/ME) | G93.3 |  | diagnostic | plasma | 22732129 |
| IL-6 | P05231 | chronic fatigue syndrome/myalgic encephalomyelitis (CFS/ME) | G93.3 |  | diagnostic | plasma | 22732129 |
| IL-8 | P10145 | chronic fatigue syndrome/myalgic encephalomyelitis (CFS/ME) | G93.3 |  | diagnostic | plasma | 22732129 |
| Lymphotoxin alpha (LTα) | P01374 | chronic fatigue syndrome/myalgic encephalomyelitis (CFS/ME) | G93.3 |  | diagnostic | plasma | 22732129 |
| TNF-alpha | P01375 | chronic fatigue syndrome/myalgic encephalomyelitis (CFS/ME) | G93.3 |  | diagnostic | plasma | 22732129 |
| vitamin E |  | chronic fatigue syndrome/myalgic encephalomyelitis (CFS/ME) | G93.3 |  | diagnostic | serum | 22732129 |
| 8-iso-prostaglandin F(2 alpha) isoprostane |  | chronic fatigue syndrome/myalgic encephalomyelitis (CFS/ME) | G93.3 |  | diagnostic |  | 22732129 |
| cortisol |  | chronic fatigue syndrome/myalgic encephalomyelitis (CFS/ME) | G93.3 |  | prognostic; theragnostic |  | 22732129 |
| nitric oxide (NO) |  | glaucoma | H40-H42 |  | marker |  | 22827637 |
| atrial natriuretic peptide (ANP) |  | cardiac disease in infants | I00-I99 |  | diagnostic |  | 22797141 |
| B-type natriuretic peptide (BNP) |  | Cardiovascular disease (CVD) in patients with chronic kidney disease (CKD) | I00-I99 |  | prognostic |  | 22489717 |
| B-type natriuretic peptide (BNP) |  | cardiovascular disease | I00-I99 |  | antecedent |  | 22681965 |
| calcium |  | Cardiovascular disease (CVD) in patients with chronic kidney disease (CKD) | I00-I99 |  | prognostic |  | 22489717 |
| Creatinine |  | Cardiovascular disease (CVD) in patients with chronic kidney disease (CKD) | I00-I99 |  | prognostic |  | 22489717 |
| Homocysteine |  | Cardiovascular disease (CVD) in patients with chronic kidney disease (CKD) | I00-I99 |  | prognostic |  | 22489717 |
| N-terminal pro-B-type natriuretic peptide (NT-pro-BNP) |  | Cardiovascular disease (CVD) in patients with chronic kidney disease (CKD) | I00-I99 |  | prognostic |  | 22489717 |
| N-terminal pro-B-type natriuretic peptide (NT-pro-BNP) |  | critically ill cardiac patient | I00-I99 |  | prognostic |  | 21440204 |
| parathyroid hormone (iPTH) |  | Cardiovascular disease (CVD) in patients with chronic kidney disease (CKD) | I00-I99 |  | prognostic |  | 22489717 |
| Vitamin D |  | Cardiovascular disease (CVD) in patients with chronic kidney disease (CKD) | I00-I99 |  | prognostic |  | 22489717 |
| B-type natriuretic peptide (BNP) |  | acute coronary syndrome (ACS) | I20.0 |  | prognostic |  | 23331845 |
| N-terminal pro-B-type natriuretic peptide (NT-pro-BNP) |  | acute coronary syndrome (ACS) | I20.0 |  | prognostic |  | 23331845 |
| B-type natriuretic peptide (BNP) |  | Myocardial infarction | I21,I22 |  | diagnostic |  | 23331845 |
| N-terminal pro-B-type natriuretic peptide (NT-pro-BNP) |  | Myocardial infarction | I21,I22 |  | diagnostic |  | 23331845 |
| adrenomedullin |  | acute myocardial infarction; heart failure | I21,I22,I50 |  | prognostic |  | 21440204 |
| MR-proadrenomedullin |  | acute myocardial infarction; heart failure | I21,I22,I50 |  | prognostic |  | 21440204 |
| Myeloid-related protein-14 (MIM 123886 S100A9, Mrp-14) | P06702 | acute myocardial infarction | I21-I22 |  | diagnostic | plasma | 23068427 |
|  |  | acute myocardial infarction | I21-I22 |  | diagnostic | plasma | 23068427 |
| B-type natriuretic peptide (BNP) |  | pulmonary embolism | I26 |  | prognostic |  | 21440204 |
| N-terminal pro-B-type natriuretic peptide (NT-pro-BNP) |  | pulmonary embolism | I26 |  | prognostic |  | 21440204 |
| N-terminal pro-B-type natriuretic peptide (NT-pro-BNP) |  | Pulmonary hypertension in Systemic sclerosis (SSc-PH) | I27.0, I27.2 |  | diagnostic | serum | 22988462 |
| brain natriuretic peptide (BNP) |  | Pulmonary hypertension in Systemic sclerosis (SSc-PH) | I27.0, I27.2 |  | diagnostic | serum | 22988462 |
| B-type natriuretic peptide (BNP) |  | pulmonary artery hypertension | I27.0, I27.2 |  | prognostic |  | 21440204 |
| N-terminal pro-B-type natriuretic peptide (NT-pro-BNP) |  | pulmonary artery hypertension | I27.0, I27.2 |  | prognostic |  | 21440204 |
| Fetuin A | P02765 | Calcific Aortic Valve Disease | I35 |  | prognostic | serum | 22489716 |
| Osteopontin (OPN) | P10451 | Calcific Aortic Valve Disease | I35 |  | monitoring | plasma | 22489716 |
| lipid |  | Calcific Aortic Valve Disease | I35 |  | prognostic |  | 22489716 |
| Galectin-3 | P17931 | Heart Failure (HF) | I50 | FDA approved | prognostic | plasma | 22980054 |
| hs-C-reactive protein | P02741 | Heart Failure (HF) | I50 |  | prognostic | serum | 22980054 |
| neutrophil gelatinase-associated lipocalin (NGAL) | P80188 | Heart Failure (HF) | I50 | FDA approved | prognostic | serum | 22980054 |
| homocysteine |  | Heart Failure (HF) | I50 |  | prognostic | serum | 22980054 |
| B-type natriuretic peptide (BNP) |  | heart failure | I50 | FDA approved | prognostic |  | 22489715 |
| B-type natriuretic peptide (BNP) |  | heart failure | I50 |  | diagnostic; prognostic |  | 22681965 |
| N-terminal brain natriuretic peptide (BNP) |  |  | I50 | clinical trial | prognostic |  | 19176440 |
| NT-pro-ANP |  | heart failure | I50 | clinical trial | prognostic |  | 22489715 |
| N-terminal Pro-BNP (NP) |  | acute congestive HF (CHF) | I50 | FDA approved | diagnostic |  | 22980054 |
| Aldosterone |  | heart failure | I50 |  | prognostic |  | 22489715 |
| Arginine vasopressin (AVP) |  | heart failure | I50 |  | prognostic |  | 22489715 |
| F2 isoprostanes |  | heart failure | I50 |  | prognostic |  | 22489715 |
| Norepinephrine |  | heart failure | I50 |  | prognostic |  | 22489715 |
| N-terminal Pro-BNP (NP) |  | Heart Failure (HF) | I50 |  | prognostic |  | 22980054 |
| N-terminal Pro-BNP (NP) |  | Heart Failure (HF) | I50 |  | antecedent |  | 22980054 |
| N-terminal Pro-BNP (NP) |  | Heart Failure (HF) | I50 |  | diagnostic |  | 22980054 |
| MR-pro-ANP |  | heart failure | I50 | clinical trial | prognostic |  | 22489715 |
| brain-type natriuretic peptide (BNP) |  |  | I50 | phase IV | prognostic |  | 23821090 |
| MR-proadrenomedullin |  | heart failure | I50 |  | prognostic |  | 22489715 |
| natriuretic peptide |  | heart failure (HF) | I50 |  | prognostic |  | 23143510 |
| tissue inhibitor of metalloproteinase (TIMP 1) |  | heart failure | I50 |  | prognostic |  | 22489715 |
| brain natriuretic peptide (BNP) |  | Heart Failure (HF) | I50 |  | diagnostic |  | 22980054 |
| brain natriuretic peptide (BNP) |  | Heart Failure (HF) | I50 |  | prognostic |  | 22980054 |
| C-terminal pro-endothelin-1 |  | heart failure | I50 |  | prognostic |  | 22489715 |
| microRNA (which microRNA?) |  | acute coronary syndrome,acute myocardial infarction, type 2 diabetes, hypertension, heart failure | I50,I10,I11,I12,I13,I15,E11,I21,I22,I20.0 |  | diagnostic | plasma | 22824111 |
| Cathepsin S | P25774 | Premature Atherosclerosis | I70 |  | diagnostic; antecedent | plasma | 22489712 |
| Cystatin C | P01034 | Premature Atherosclerosis | I70 |  | diagnostic; antecedent | plasma | 22489712 |
| Lipoprotein-associated phospholipase A2 (Lp- PLA2) | Q13093 | Premature Atherosclerosis | I70 |  | diagnostic; antecedent | serum | 22489712 |
| Myeloid-related protein-14 (MIM 123886 S100A9, Mrp-14) | P06702 | carotid artery atherosclerosis | I70 |  | antecedent | plasma | 23068427 |
| Myeloid-related protein-8 (MIM 123885 S100A8, Mrp-8) | P05109 | carotid artery atherosclerosis | I70 |  | antecedent | plasma | 23068427 |
| B-type natriuretic peptide (BNP) |  | Atherosclerosis | I70 |  | diagnostic |  | 22577298 |
| 3-Nitrotyrosine (3-NT) |  | carotid artery atherosclerosis | I70 |  | diagnostic |  | 23068427 |
| F2 isoprostanes |  | Atherosclerosis | I70 |  | diagnostic |  | 22577298 |
| homocysteine |  | Premature Atherosclerosis | I70 |  | diagnostic; antecedent |  | 22489712 |
| Asymmetric dimethylarginine (ADMA) |  | Atherosclerosis | I70 |  | diagnostic |  | 22577298 |
| Asymmetric dimethylarginine (ADMA) |  | Atherosclerosis | I70 |  | prognostic |  | 22577298 |
| adhesion molecule-1 |  | Peripheral arterial disease (PAD) | I73 |  | antecedent |  | 22489720 |
| apolipoprotein A (ApoA) | P06727,P02647,P02652,Q6Q788 | Atherothrombosis | I80-I82 |  | antecedent | blood | 23630624 |
| apolipoprotein B (ApoB) | Q0VD83 | Atherothrombosis | I80-I82 |  | antecedent | blood | 23630624 |
| cardiac troponin I (cTnI) | P19429 | Atherothrombosis | I80-I82 | used in clinic | antecedent | blood | 23630624 |
| cardiac troponin T (cTnT) | P45379 | Atherothrombosis | I80-I82 | used in clinic | antecedent | blood | 23630624 |
| C-reactive protein (CRP) | P02741 | Atherothrombosis | I80-I82 | used in clinic | antecedent | blood | 23630624 |
| cystatin C | P01034 | Atherothrombosis | I80-I82 |  | antecedent | plasma | 23630624 |
| fibrinogen | P02679,P02671,P02675 | Atherothrombosis | I80-I82 | used in clinic | antecedent | blood | 23630624 |
| fibrinogen | P02679,P02671,P02675 | Atherothrombosis | I80-I82 |  | antecedent | blood | 23630624 |
| high density lipoprotein (HDL) | Q00341 | Atherothrombosis | I80-I82 |  | antecedent | serum | 23630624 |
| high sensitivity C-reactive protein (hs CRP) | P02741 | Atherothrombosis | I80-I82 | used in clinic | antecedent | blood | 23630624 |
| cholesterol |  | Atherothrombosis | I80-I82 | used in clinic | antecedent | blood | 23630624 |
| Creatinine |  | Atherothrombosis | I80-I82 |  | antecedent | serum | 23630624 |
| uric acid |  | Atherothrombosis | I80-I82 |  | antecedent | serum | 23630624 |
| Vitamin D |  | Atherothrombosis | I80-I82 |  | antecedent | serum | 23630624 |
| D-dimer |  | venous thromboembolism (VTE) | I80-I82 |  | prognostic |  | 22528325 |
| D-dimer |  | deep venous thrombosis (DVT) | I82.4 |  | antecedent |  | 22528325 |
| D-dimer |  | deep venous thrombosis (DVT) | I82.4 |  | prognostic |  | 22528325 |
| MR-pro-ANP |  | lower respiratory tract infections | J09-J99 |  | classification |  | 21440204 |
| Pro-ANP |  | lower respiratory tract infections | J09-J99 |  | classification |  | 21440204 |
| MR-proadrenomedullin |  | community-acquired pneumonia | J12, J13, J14, J15, J16, J17, J18, P23 |  | classification |  | 21440204 |
| Aa-Val360 |  | Chronic obstructive pulmonary disease (COPD) | J40-J44, J47 |  | diagnostic | plasma | 23361193 |
| C-reactive protein (CRP) | P02741 | Chronic obstructive pulmonary disease (COPD) | J40-J44, J47 |  | diagnostic | plasma | 23361193 |
| fibrinogen | P02679,P02671,P02675 | Chronic obstructive pulmonary disease (COPD) | J40-J44, J47 |  | prognostic | plasma | 23361193 |
| immunoglobulin light-chain |  | Chronic obstructive pulmonary disease (COPD) | J40-J44, J47 |  | diagnostic | plasma | 23361193 |
| PARC/CCL18 | P55774 | Chronic obstructive pulmonary disease (COPD) | J40-J44, J47 |  | diagnostic | plasma | 23361193 |
| PARC/CCL18 | P55774 | Chronic obstructive pulmonary disease (COPD) | J40-J44, J47 |  | prognostic | plasma | 23361193 |
| desmosine |  | Chronic obstructive pulmonary disease (COPD) | J40-J44, J47 |  | diagnostic | urine; plasma | 23361193 |
| b-defensins |  | Chronic obstructive pulmonary disease (COPD) | J40-J44, J47 |  | diagnostic |  | 23361193 |
| brain natriuretic peptide (BNP) |  | chronic lung disease | J40-J44,J45,J47,P27.1 |  | prognostic | serum | 22988462 |
| Aa-Val360 |  | emphysema | J43 |  | prognostic | plasma | 23361193 |
| high-sensitivity C-reactive protein (hs-CRP) | P02741 | asthma | J45 |  | diagnostic | serum; EBC | 22796631 |
| YKL-40 | P36222 | asthma | J45 |  | diagnostic | serum | 19532094 |
| YKL-40 | P36222 | asthma | J45 |  | monitoring | serum | 19532094 |
| exhaled nitric oxide (eNO) |  | asthma | J45 |  | diagnostic |  | 22877617 |
| pro-ANP |  | acute lung injury | J80 |  | prognostic |  | 21440204 |
| CC Chemokine Ligand 18 (CCL18) | P55774 | Systemic sclerosis associated interstitial lung disease (SSc-ILD) | J80,J81,J82,J84,M34 |  | prognostic | serum | 22988462 |
| CC Chemokine Ligand 2 (CCL2 | O00590 | Systemic sclerosis associated interstitial lung disease (SSc-ILD) | J80,J81,J82,J84,M34 |  | diagnostic | serum; BAL | 22988462 |
| connective tissue growth factor (CTGF) | P29279 | Systemic sclerosis associated interstitial lung disease (SSc-ILD) | J80,J81,J82,J84,M34 |  | diagnostic | serum | 22988462 |
| CXC Chemokine Ligand 10 (CXCL10) | P02778 | Systemic sclerosis associated interstitial lung disease (SSc-ILD) | J80,J81,J82,J84,M34 |  | diagnostic | serum | 22988462 |
| IL-10 | P22301 | Systemic sclerosis associated interstitial lung disease (SSc-ILD) | J80,J81,J82,J84,M34 |  | diagnostic | serum | 22988462 |
| IL-13 | P35225 | Systemic sclerosis associated interstitial lung disease (SSc-ILD) | J80,J81,J82,J84,M34 |  | diagnostic | serum | 22988462 |
| IL-17 | Q16552 | Systemic sclerosis associated interstitial lung disease (SSc-ILD) | J80,J81,J82,J84,M34 |  | diagnostic | serum | 22988462 |
| IL-22 | Q9GZX6 | Systemic sclerosis associated interstitial lung disease (SSc-ILD) | J80,J81,J82,J84,M34 |  | diagnostic | serum | 22988462 |
| IL-4 | P05112 | Systemic sclerosis associated interstitial lung disease (SSc-ILD) | J80,J81,J82,J84,M34 |  | diagnostic | serum | 22988462 |
| IL-6 | P05231 | Systemic sclerosis associated interstitial lung disease (SSc-ILD) | J80,J81,J82,J84,M34 |  | diagnostic | serum | 22988462 |
| Krebs Von Den Lungen 6 (KL-6) | P15941 | Systemic sclerosis associated interstitial lung disease (SSc-ILD) | J80,J81,J82,J84,M34 |  | diagnostic | serum | 22988462 |
| Krebs Von Den Lungen 6 (KL-6) | P15941 | Systemic sclerosis associated interstitial lung disease (SSc-ILD) | J80,J81,J82,J84,M34 |  | monitoring; theragnostic | serum | 22988462 |
| Krebs Von Den Lungen 6 (KL-6) | P15941 | Systemic sclerosis associated interstitial lung disease (SSc-ILD) | J80,J81,J82,J84,M34 |  | prognostic | serum | 22988462 |
| Matrix Metalloproteinase 7 (MMP7) | P09237 | Systemic sclerosis associated interstitial lung disease (SSc-ILD) | J80,J81,J82,J84,M34 |  | diagnostic | serum | 22988462 |
| matrix metalloproteinase 9 (MMP 9) | P14780 | Systemic sclerosis associated interstitial lung disease (SSc-ILD) | J80,J81,J82,J84,M34 |  | diagnostic | serum; BAL | 22988462 |
| monocyte chemoattractant protein 1 (MCP-1) | P13500 | Systemic sclerosis associated interstitial lung disease (SSc-ILD) | J80,J81,J82,J84,M34 |  | diagnostic | serum; BAL | 22988462 |
| Surfactant Protein A (SP-A) | Q8IWL2,Q8IWL1 | Systemic sclerosis associated interstitial lung disease (SSc-ILD) | J80,J81,J82,J84,M34 |  | diagnostic | serum | 22988462 |
| Surfactant Protein D (SP-D) | P35247 | Systemic sclerosis associated interstitial lung disease (SSc-ILD) | J80,J81,J82,J84,M34 |  | diagnostic | serum | 22988462 |
| isoprostane |  | Systemic sclerosis associated interstitial lung disease (SSc-ILD) | J80,J81,J82,J84,M34 |  | diagnostic | serum | 22988462 |
| albumin (ALB) | P43652 | idiopathic pulmonary fibrosis (IPF) | J84.1 |  | prognostic | serum | 22424426 |
| CCL18 | P55774 | idiopathic pulmonary fibrosis (IPF) | J84.1 |  | monitoring | blood | 22424426 |
| CCL18 | P55774 | idiopathic pulmonary fibrosis (IPF) | J84.1 |  | prognostic | blood | 22424426 |
| CD28 | P10747 | idiopathic pulmonary fibrosis (IPF) | J84.1 |  | monitoring | blood | 22424426 |
| CD28 | P10747 | idiopathic pulmonary fibrosis (IPF) | J84.1 |  | prognostic | blood | 22424426 |
| CD4 | P01730 | idiopathic pulmonary fibrosis (IPF) | J84.1 |  | monitoring | blood | 22424426 |
| CD4 | P01730 | idiopathic pulmonary fibrosis (IPF) | J84.1 |  | prognostic | blood | 22424426 |
| Krebs Von Den Lungen 6 (KL-6) | P15941 | idiopathic pulmonary fibrosis (IPF) | J84.1 |  | prognostic | blood | 22424426 |
| Krebs Von Den Lungen 6 (KL-6) | P15941 | idiopathic pulmonary fibrosis (IPF) | J84.1 |  | differentiation | blood | 22424426 |
| Matrix Metalloproteinase 7 (MMP7) | P09237 | idiopathic pulmonary fibrosis (IPF) | J84.1 |  | differentiation | blood | 22424426 |
| Matrix Metalloproteinase 7 (MMP7) | P09237 | idiopathic pulmonary fibrosis (IPF) | J84.1 |  | prognostic | blood | 22424426 |
| Matrix Metalloproteinase 7 (MMP7) | P09237 | idiopathic pulmonary fibrosis (IPF) | J84.1 |  | monitoring | blood | 22424426 |
| MMP1 | P03956 | idiopathic pulmonary fibrosis (IPF) | J84.1 |  | differentiation | blood | 22424426 |
| Osteopontin (OPN) | P10451 | idiopathic pulmonary fibrosis (IPF) | J84.1 |  | monitoring | blood | 22424426 |
| Periostin | Q15063 | idiopathic pulmonary fibrosis (IPF) | J84.1 |  | differentiation | blood | 22424426 |
| Periostin | Q15063 | idiopathic pulmonary fibrosis (IPF) | J84.1 |  | prognostic | blood | 22424426 |
| SP-D | P35247 | idiopathic pulmonary fibrosis (IPF) | J84.1 |  | differentiation | blood | 22424426 |
| SP-D | P35247 | idiopathic pulmonary fibrosis (IPF) | J84.1 |  | prognostic | blood | 22424426 |
| Surfactant Protein A (SP-A) | Q8IWL2,Q8IWL1 | idiopathic pulmonary fibrosis (IPF) | J84.1 |  | prognostic | blood | 22424426 |
| Surfactant Protein A (SP-A) | Q8IWL2,Q8IWL1 | idiopathic pulmonary fibrosis (IPF) | J84.1 |  | differentiation | blood | 22424426 |
| vascular endothelial growth factor (VEGF) | P15692,P49765,P49767,O43915 | idiopathic pulmonary fibrosis (IPF) | J84.1 |  | prognostic | blood | 22424426 |
| YKL-40 | P36222 | idiopathic pulmonary fibrosis (IPF) | J84.1 |  | prognostic | blood | 22424426 |
| YKL-40 | P36222 | idiopathic pulmonary fibrosis (IPF) | J84.1 |  | monitoring | blood | 22424426 |
| Brain natriuretic peptide (BNP) |  | idiopathic pulmonary fibrosis (IPF) | J84.1 |  | prognostic | serum | 22424426 |
| CYFRA 21-1 |  | malignant pleural effusion | J91.0 |  | diagnostic | serum; pleural effusion | 8 |
| eosinophil-derived neurotoxin (EDN) | P10153 | Eosinophilic esophagitis (EoE) | K20.0 |  | monitoring | tissue; serum | 22920068 |
| IL-13 | P35225 | Eosinophilic esophagitis (EoE) | K20.0 |  | surrogate | tissue; serum | 22920068 |
| kynurenine |  | Crohn''s disease (CD) | K50 |  | monitoring | serum | 22424434 |
| tryptophan |  | Crohn''s disease (CD) | K50 |  | monitoring | serum | 22424434 |
| antibodies to Cbir1 |  | Inflammatory Bowel Disease (IBD) | K50,K51 |  | prognostic | serum | 22424434 |
| antibodies to Fla- X |  | Inflammatory Bowel Disease (IBD) | K50,K51 |  | prognostic | serum | 22424434 |
| antibodies to Fla- X |  | Inflammatory Bowel Disease (IBD) | K50,K51 |  | diagnostic | serum | 22424434 |
| antibodies to Fla- X |  | Inflammatory Bowel Disease (IBD) | K50,K51 |  | differentiation | serum | 22424434 |
| antibodies to Flagellin A4-Fla2 |  | Inflammatory Bowel Disease (IBD) | K50,K51 |  | prognostic | serum | 22424434 |
| antibodies to Flagellin A4-Fla2 |  | Inflammatory Bowel Disease (IBD) | K50,K51 |  | diagnostic | serum | 22424434 |
| antibodies to Flagellin A4-Fla2 |  | Inflammatory Bowel Disease (IBD) | K50,K51 |  | differentiation | serum | 22424434 |
| Antibodies to outer membrane porin (Anti-OmpC) |  | Inflammatory Bowel Disease (IBD) | K50,K51 |  | diagnostic | serum | 22424434 |
| Antibodies to outer membrane porin (Anti-OmpC) |  | Inflammatory Bowel Disease (IBD) | K50,K51 |  | differentiation | serum | 22424434 |
| Antibodies to outer membrane porin (Anti-OmpC) |  | Inflammatory Bowel Disease (IBD) | K50,K51 |  | prognostic | serum | 22424434 |
| antichitobioside carbohydrate IgA (ACCA) |  | Inflammatory Bowel Disease (IBD) | K50,K51 |  | diagnostic | serum | 22424434 |
| antichitobioside carbohydrate IgA (ACCA) |  | Inflammatory Bowel Disease (IBD) | K50,K51 |  | differentiation | serum | 22424434 |
| Anti-I2 |  | Inflammatory Bowel Disease (IBD) | K50,K51 |  | prognostic | serum | 22424434 |
| antilaminaribioside carbohydrate IgG (ALCA) |  | Inflammatory Bowel Disease (IBD) | K50,K51 |  | diagnostic | serum | 22424434 |
| antilaminaribioside carbohydrate IgG (ALCA) |  | Inflammatory Bowel Disease (IBD) | K50,K51 |  | differentiation | serum | 22424434 |
| Anti-neutrophil cytoplasmic antibodies (ANCAs) |  | Inflammatory Bowel Disease (IBD) | K50,K51 |  | diagnostic | serum | 22424434 |
| Anti-neutrophil cytoplasmic antibodies (ANCAs) |  | Inflammatory Bowel Disease (IBD) | K50,K51 |  | differentiation | serum | 22424434 |
| anti-Saccharomyces cerevisiae antibodies (ASCA) |  | Inflammatory Bowel Disease (IBD) | K50,K51 |  | diagnostic | serum | 22424434 |
| anti-Saccharomyces cerevisiae antibodies (ASCA) |  | Inflammatory Bowel Disease (IBD) | K50,K51 |  | differentiation | serum | 22424434 |
| anti-Saccharomyces cerevisiae antibodies (ASCA) |  | Inflammatory Bowel Disease (IBD) | K50,K51 |  | prognostic | serum | 22424434 |
| anti-synthetic mannoside antibodies (AΣMA or AMCA) |  | Inflammatory Bowel Disease (IBD) | K50,K51 |  | diagnostic | serum | 22424434 |
| anti-synthetic mannoside antibodies (AΣMA or AMCA) |  | Inflammatory Bowel Disease (IBD) | K50,K51 |  | differentiation | serum | 22424434 |
| Flagellin (Anti-Cbir1) |  | Inflammatory Bowel Disease (IBD) | K50,K51 |  | diagnostic | serum | 22424434 |
| Flagellin (Anti-Cbir1) |  | Inflammatory Bowel Disease (IBD) | K50,K51 |  | differentiation | serum | 22424434 |
| Pseudomonas flourescens-associated sequence I–2 (Anti-I2) |  | Inflammatory Bowel Disease (IBD) | K50,K51 |  | diagnostic | serum | 22424434 |
| Pseudomonas flourescens-associated sequence I–2 (Anti-I2) |  | Inflammatory Bowel Disease (IBD) | K50,K51 |  | differentiation | serum | 22424434 |
| L-arginine |  | ulcerative colitis (UC) | K51 |  | diagnostic | serum | 22424434 |
| CK-18 | P05783 | steatohepatitis | K70.1, K76.0 |  | diagnostic | serum; plasma | 23227839 |
| alanine aminotransferase (ALT) | Q8TD30,P24298 | nonalcoholic steatohepatitis (NASH) | K76.0 |  | diagnostic | serum | 23227839 |
| aspartate aminotransferase (AST) | P17174 | nonalcoholic steatohepatitis (NASH) | K76.0 |  | diagnostic | serum | 23227839 |
| CK-18 | P05783 | nonalcoholic steatohepatitis (NASH) | K76.0 |  | differentiation | serum | 23227839 |
| CK-18 | P05783 | non-alcoholic steatohepatitis (NASH) | K76.0 |  | diagnostic | serum | 22567408 |
| C-reactive protein (CRP) | P02741 | nonalcoholic steatohepatitis (NASH) | K76.0 |  | diagnostic | serum | 23227839 |
| FAS (CD95) | P25445 | nonalcoholic steatohepatitis (NASH) | K76.0 |  | diagnostic | serum | 23227839 |
| IL-6 | P05231 | nonalcoholic steatohepatitis (NASH) | K76.0 |  | diagnostic | serum | 23227839 |
| M-30 | P05783 | nonalcoholic steatohepatitis (NASH) | K76.0 |  | diagnostic | serum | 23227839 |
| M-65 | P05783 | nonalcoholic steatohepatitis (NASH) | K76.0 |  | diagnostic | serum | 23227839 |
| TNF-alpha | P01375 | nonalcoholic steatohepatitis (NASH) | K76.0 |  | diagnostic | serum | 23227839 |
| Type IV collagen 7S | P53420,P29400,P02462,P08572,Q14031,Q01955 | nonalcoholic steatohepatitis (NASH) | K76.0 |  | prognostic | serum | 23227839 |
| uncleaved CK-18 | P05783 | non-alcoholic steatohepatitis (NASH) | K76.0 |  | differentiation | serum | 22567408 |
| Fasting glucose |  | nonalcoholic steatohepatitis (NASH) | K76.0 |  | diagnostic | serum | 23227839 |
| Hyaluronic acid |  | nonalcoholic steatohepatitis (NASH) | K76.0 |  | diagnostic | serum | 23227839 |
| N-terminal propeptide of type III collagen (PIIINP) |  | nonalcoholic steatohepatitis (NASH) | K76.0 |  | diagnostic | serum | 23227839 |
| IL-8 | P10145 | acute graft-versus-host disease (aGVHD); rejection; liver fibrosis in thalassemic patients; sepsis;veno-occlusive disease | K76.5,A40-A41,D56,K70.3, K71.7, K74,T86,D89.8 |  | diagnostic | plasma | 23165480 |
| IL-2Ra | P01589 | acute graft-versus-host disease (aGVHD);sepsis;veno-occlusive disease | K76.5,A40-A41,D89.8 |  | diagnostic | plasma | 23165480 |
| IL-6 | P05231 | acute graft-versus-host disease (aGVHD);sepsis;veno-occlusive disease | K76.5,A40-A41,D89.8 |  | diagnostic | plasma | 23165480 |
| C-reactive protein (CRP) | P02741 | acute graft-versus-host disease (aGVHD);microangiopathic hemolytic anemia;veno-occlusive disease | K76.5,D59.4,D89.8 |  | diagnostic | plasma | 23165480 |
| TNF-alpha | P01375 | acute graft-versus-host disease (aGVHD);microangiopathic hemolytic anemia;veno-occlusive disease | K76.5,D59.4,D89.8 |  | diagnostic | plasma | 23165480 |
| IgA anti-DGP (antideaminated forms of gliadin peptides antibodies) |  | Celiac Disease | K90.0 | used in clinic | diagnostic | serum | 23388848 |
| IgA anti-EMA (antiendomysium antibody) |  | Celiac Disease | K90.0 | used in clinic | diagnostic | serum | 23388848 |
| IgA anti-tTG |  | Celiac Disease | K90.0 | used in clinic | diagnostic | serum | 23388848 |
| Adiponectin | Q15848 | psoriasis | L40 |  | diagnostic | serum | 23532439 |
| alpha 2 -antiplasmin | P08697 | psoriasis | L40 |  | diagnostic | blood | 23532439 |
| C4 |  | psoriasis | L40 |  | diagnostic | blood | 23532439 |
| C-reactive protein (CRP) | P02741 | psoriasis | L40 |  | diagnostic | serum | 23532439 |
| fibrinogen | P02679,P02671,P02675 | psoriasis | L40 |  | diagnostic | blood | 23532439 |
| haptoglobin | P00738 | psoriasis | L40 |  | diagnostic | serum | 23532439 |
| IFNγ | P01579 | psoriasis | L40 |  | diagnostic | serum | 23532439 |
| IL-12 | Q99665 | psoriasis | L40 |  | diagnostic | serum | 23532439 |
| IL-18 | Q14116 | psoriasis | L40 |  | diagnostic | serum | 23532439 |
| IL-6 | P05231 | psoriasis | L40 |  | diagnostic | serum | 23532439 |
| IL-6 | P05231 | psoriasis | L40 |  | differentiation | serum | 23532439 |
| IL-8 | P10145 | psoriasis | L40 |  | diagnostic | serum | 23532439 |
| leptin | P41159 | psoriasis | L40 |  | diagnostic | serum | 23532439 |
| lipocalin | Q6UWW0 | psoriasis | L40 |  | diagnostic | serum | 23532439 |
| Plasminogen | P00747 | psoriasis | L40 |  | diagnostic | blood | 23532439 |
| Protein C |  | psoriasis | L40 |  | diagnostic | blood | 23532439 |
| prothrombin fragments 1+2 | P00734 | psoriasis | L40 |  | diagnostic | blood | 23532439 |
| P-selectin | P16109 | psoriasis | L40 |  | diagnostic | serum | 23532439 |
| resistin | Q9HD89 | psoriasis | L40 |  | diagnostic | serum | 23532439 |
| TNF | P01375 | psoriasis | L40 |  | diagnostic | serum | 23532439 |
| 8-hydroxy guanosine |  | psoriasis | L40 |  | diagnostic | blood | 23532439 |
| Fibrinopeptide A |  | psoriasis | L40 |  | diagnostic | blood | 23532439 |
| Bβ and D-dimer |  | psoriasis | L40 |  | diagnostic | blood | 23532439 |
| KOA1 (TGLESGHGPGDS) |  | knee osteoarthritis | M15-M19, M47 |  | marker | serum | 22842200 |
| cartilage glycoprotein 39 (YKL-40) | P36222 | osteoarthritis | M15-M19,M47 |  | diagnostic | serum;Synovial fluid | 17538566 |
| cartilage glycoprotein 39 (YKL-40) | P36222 | osteoarthritis | M15-M19,M47 |  | pharmacodynamic; theragnostic | serum;Synovial fluid | 17538566 |
| cartilage oligomeric protein (COMP) | P49747 | osteoarthritis | M15-M19,M47 |  | diagnostic; prognostic | serum;Synovial fluid | 17538566 |
| cartilage oligomeric protein (COMP) | P49747 | osteoarthritis | M15-M19,M47 |  | diagnostic | serum;Synovial fluid | 17538566 |
| COL2-3/4C (long) epitope | P02458 | osteoarthritis | M15-M19,M47 |  | prognostic | urine; serum | 17538566 |
| COL2-3/4C (long) epitope | P02458 | osteoarthritis | M15-M19,M47 |  | pharmacodynamic; theragnostic | urine; serum | 17538566 |
| COL2-3/4C (short) epitope | P02458 | osteoarthritis | M15-M19,M47 |  | prognostic | urine; serum | 17538566 |
| COL2-3/4C (short) epitope | P02458 | osteoarthritis | M15-M19,M47 |  | pharmacodynamic; theragnostic | urine; serum | 17538566 |
| Follistatin-like protein 1 (FSTL1) | Q12841 | osteoarthritis | M15-M19,M47 |  | monitoring | serum | 22842200 |
| lumican | P51884 | osteoarthritis | M15-M19,M47 |  | diagnostic | serum | 22842200 |
| matrix metalloproteinase (MMP) | P50281,P45452,O75900,P51511,Q99542,P51512,P03956,Q8N119,Q9NRE1,O60882,Q9H239,Q9ULZ9,P08254,P22894,P39900,P08253,P14780,Q9H306,P09238,P24347,Q9NPA2,P09237,Q9Y5R2 | osteoarthritis | M15-M19,M47 |  | prognostic | serum | 17538566 |
| matrix metalloproteinase (MMP) | P50281,P45452,O75900,P51511,Q99542,P51512,P03956,Q8N119,Q9NRE1,O60882,Q9H239,Q9ULZ9,P08254,P22894,P39900,P08253,P14780,Q9H306,P09238,P24347,Q9NPA2,P09237,Q9Y5R2 | osteoarthritis | M15-M19,M47 |  | pharmacodynamic; theragnostic | serum | 17538566 |
| matrix metalloproteinase (MMP) | P50281,P45452,O75900,P51511,Q99542,P51512,P03956,Q8N119,Q9NRE1,O60882,Q9H239,Q9ULZ9,P08254,P22894,P39900,P08253,P14780,Q9H306,P09238,P24347,Q9NPA2,P09237,Q9Y5R2 | osteoarthritis | M15-M19,M47 |  | diagnostic | serum | 17538566 |
| osteocalcin (OC) | P02818 | osteoarthritis | M15-M19,M47 |  | prognostic | serum | 17538566 |
| tetranectin | P05452 | osteoarthritis | M15-M19,M47 |  | diagnostic | serum | 22842200 |
| tissue inhibitor of matrix metalloproteinase (TIMP) | P16035,P35625,P01033 | osteoarthritis | M15-M19,M47 |  | diagnostic | serum | 17538566 |
| tissue inhibitor of matrix metalloproteinase (TIMP) | P16035,P35625,P01033 | osteoarthritis | M15-M19,M47 |  | prognostic | serum | 17538566 |
| von Willebrand factor | P04275 | osteoarthritis | M15-M19,M47 |  | diagnostic | serum | 22842200 |
| Hyaluronic acid |  | osteoarthritis | M15-M19,M47 |  | diagnostic | serum | 17538566 |
| Hyaluronic acid |  | osteoarthritis | M15-M19,M47 |  | prognostic | serum | 17538566 |
| N-propeptide IIA of collagen type II (PIIANP) |  | osteoarthritis | M15-M19,M47 |  | prognostic | serum | 17538566 |
| C-propeptide of collagen type II (PIICP) |  | osteoarthritis | M15-M19,M47 |  | diagnostic; prognostic | serum;Synovial fluid | 17538566 |
| N-terminal cross-linked telopeptide of type I collagen (NTX-I) |  | osteoarthritis | M15-M19,M47 |  | diagnostic | urine; serum | 17538566 |
| Pentosidine |  | osteoarthritis | M15-M19,M47 |  | prognostic | urine; serum | 17538566 |
| nine-amino-acid peptide of type II collagen (Coll 2-1) |  | osteoarthritis | M15-M19,M47 |  | prognostic | urine; serum | 17538566 |
| nitrated form of nine-amino-acid peptide of type II collagen (Coll 2-1 NO2) |  | osteoarthritis | M15-M19,M47 |  | prognostic | urine; serum | 17538566 |
| C-terminal cross-linked telopeptide of type I collagen (CTX-I) |  | osteoarthritis | M15-M19,M47 |  | prognostic | urine; serum | 17538566 |
| Fibulin-3 peptide 1 |  | osteoarthritis | M15-M19,M47 |  | diagnostic |  | 22842200 |
| Fibulin-3 peptide 2 |  | osteoarthritis | M15-M19,M47 |  | diagnostic |  | 22842200 |
| N-propeptide II of collagen type II (PIINP) |  | osteoarthritis | M15-M19,M47 |  | diagnostic |  | 17538566 |
| IFNα | P01563,P01562,P32881,P01571,P05014,P01568,P01567,P01566,P01569,P01570,P05013,P05015 | Systemic lupus erythematosus | M32 |  | diagnostic | serum | 22732129 |
| endothelin-1 (ET-1) | P05305 | Systemic sclerosis (SSc) | M34 |  | diagnostic | serum | 22988462 |
| soluble E-selectin (sE-selectin) | P16581 | Systemic sclerosis (SSc) | M34 |  | diagnostic | serum | 22988462 |
| soluble vascular cell adhesion molecule- 1 (sVCAM-1) | P19320 | Systemic sclerosis (SSc) | M34 |  | diagnostic | serum | 22988462 |
| vascular endothelial growth factor (VEGF) | P15692,P49765,P49767,O43915 | Systemic sclerosis (SSc) | M34 |  | diagnostic | serum | 22988462 |
| Haemopexin | P02790 | minimal change nephropathy (MCN) | N00.0,N01.0,N02.0,N03.0,N04.0,N05.0,N06.0,N07.0,N08.0 |  | diagnostic | serum; urine | 23013941 |
| IL-13 | P35225 | minimal change nephropathy (MCN) | N00.0,N01.0,N02.0,N03.0,N04.0,N05.0,N06.0,N07.0,N08.0 |  | prognostic | Plasma; lymphocytes | 23013941 |
| sIL-2R | P01589,P14784,P31785 | minimal change nephropathy (MCN) ; Focal segmental glomerulonephritis (FSG) | N00.1,N01.1,N02.1,N03.1,N04.1,N05.1,N06.1,N07.1,N08.1,N00.0,N01.0,N02.0,N03.0,N04.0,N05.0,N06.0,N07.0,N08.0 |  | diagnostic | serum | 23013941 |
| soluble urokinase-type plasminogen activator receptor (suPAR) | Q03405 | Focal segmental glomerulonephritis (FSG) | N00-N08 |  | diagnostic | serum | 23013941 |
| anti aldose-reductase antibodies (Anti-AR antibodies) |  | membranous nephropathy (MN) | N02.2 |  | diagnostic | serum | 23013941 |
| anti-M type phospholipase A2 receptor antibodies (Anti-PLA2R antibodies) |  | membranous nephropathy (MN) | N02.2 |  | diagnostic | serum | 23013941 |
| anti-M type phospholipase A2 receptor antibodies (Anti-PLA2R antibodies) |  | membranous nephropathy (MN) | N02.2 |  | differentiation | serum | 23013941 |
| Anti-SOD2 antibodies |  | membranous nephropathy (MN) | N02.2 |  | diagnostic | serum | 23013941 |
| Cystatin C | P01034 | ACUTE KIDNEY INJURY (AKI) | N17 |  | diagnostic | serum | 22983082 |
| cystatin C (CyC) | P01034 | acute kidney injury in pediatric cardiac patients | N17 |  | diagnostic | urine;serum | 22731900 |
| IL-18 | Q14116 | acute kidney injury in pediatric cardiac patients | N17 |  | diagnostic | urine;serum | 22731900 |
| neutrophil gelatinase-associated lipocalin (NGAL) | P80188 | acute kidney injury in pediatric cardiac patients | N17 | clinical trial | diagnostic | urine;serum | 22731900 |
| neutrophil gelatinase-associated lipocalin (NGAL) | P80188 | ACUTE KIDNEY INJURY (AKI) | N17 |  | diagnostic | plasma | 22983082 |
| urea nitrogen |  | ACUTE KIDNEY INJURY (AKI) | N17 |  | diagnostic | blood | 22983082 |
| Creatinine |  | ACUTE KIDNEY INJURY (AKI) | N17 |  | diagnostic | serum | 22983082 |
| Creatinine |  | kidney injury | N17 |  | diagnostic |  | 22731900 |
| ProBNP |  | chronic kidney disease | N18 |  | prognostic |  | 22914685 |
| C-reactive protein (CRP) | P02741 | overactive bladder | N32.81 |  | diagnostic; monitoring | serum | 23314226 |
| C-reactive protein (CRP) | P02741 | overactive bladder | N32.81 |  | prognostic | serum | 23314226 |
| Nerve growth factor(NGF) | P01138 | overactive bladder | N32.81 |  | diagnostic; monitoring | serum | 23314226 |
| Cystatin C | P01034 | renal alteration |  |  | surrogate | serum; Glomerulus | 22716111 |
| Eosinophil cationic protein (ECP) | P12724 | allergic and eosinophilic diseases |  |  | diagnostic; monitoring | serum | 22683541 |
| eosinophil peroxidase (EPO) | P11678 | allergic and eosinophilic diseases |  |  | diagnostic; monitoring | serum | 22683541 |
| eosinophil-derived neurotoxin (EDN) | P10153 | allergic and eosinophilic diseases |  |  | diagnostic; monitoring | serum | 22683541 |
| IL-6 | P05231 | risks of death and of SPC |  | clinical trial | prognostic | serum | 518 |
| major basic protein (MBP) | P13727 | allergic and eosinophilic diseases |  |  | diagnostic; monitoring | serum | 22683541 |
| neutrophil gelatinase-associated lipocalin (NGAL) | P80188 | renal alteration |  |  | surrogate | serum; Glomerulus | 22716111 |
| Asymmetric dimethylarginine (ADMA) | CID501 | pre-eclampsia | O11, O14 |  | monitoring | serum | 22943702 |
| Placental protein 13 (PP13) |  | pre-eclampsia | O11, O14 | clinical trial | antecedent | serum | 22943702 |
| Pregnancy associated plasma protein-A (PAPP-A) | Q13219 | pre-eclampsia | O11, O14 | clinical trial | antecedent | serum | 22943702 |
| Pregnancy associated plasma protein-A (PAPP-A) | Q13219 | pre-eclampsia | O11, O14 |  | monitoring | serum | 22943702 |
| soluble Fms-like tyrosine kinase 1 (sFlt-1) | P17948 | pre-eclampsia | O11, O14 |  | monitoring | serum | 22943702 |
| homocysteine |  | pre-eclampsia | O11, O14 |  | monitoring | serum | 22943702 |
| homocysteine |  | pre-eclampsia | O11, O14 |  | diagnostic |  | 22943702 |
| Inhibin A | P05111 | subsequent preeclampsia in patients with previous PE and/or chronic hypertension (CHTN) | O11.9 | clinical trial | antecedent | serum | 18771979 |
| placenta growth factor (PLGF) | P49763 | subsequent preeclampsia in patients with previous PE and/or chronic hypertension (CHTN) | O11.9 | clinical trial | antecedent | serum | 18771979 |
| soluble fms-like tyrosine kinase-1 (sFlt-1) | P17948 | subsequent preeclampsia in patients with previous PE and/or chronic hypertension (CHTN) | O11.9 | clinical trial | antecedent | serum | 18771979 |
| Angiopoietin 1 | Q15389 | bronchopulmonary dysplasia | P27.1 |  | antecedent | Cord blood | 23523392 |
| Clara cell secretory protein | P17559 | bronchopulmonary dysplasia | P27.1 |  | antecedent | Cord blood; Infant blood | 23523392 |
| Endostatin | P39060 | bronchopulmonary dysplasia | P27.1 |  | antecedent | Cord blood | 23523392 |
| Eosinophil cationic protein (ECP) | P12724 | bronchopulmonary dysplasia | P27.1 |  | antecedent | Infant blood | 23523392 |
| granulocyte colony stimulating factor (G-CSF) | Q99062 | bronchopulmonary dysplasia | P27.1 |  | antecedent | Infant blood | 23523392 |
| IFNγ | P01579 | bronchopulmonary dysplasia | P27.1 | clinical trial | antecedent | Infant blood | 23523392 |
| IL-10 | P22301 | bronchopulmonary dysplasia | P27.1 | clinical trial | antecedent | Infant blood | 23523392 |
| IL-17 | Q16552 | bronchopulmonary dysplasia | P27.1 |  | antecedent | Infant blood | 23523392 |
| IL-1β | P01584 | bronchopulmonary dysplasia | P27.1 |  | antecedent | Infant blood | 23523392 |
| IL-6 | P05231 | bronchopulmonary dysplasia | P27.1 | clinical trial | antecedent | Infant blood | 23523392 |
| IL-8 | P10145 | bronchopulmonary dysplasia | P27.1 | clinical trial | antecedent | Infant blood | 23523392 |
| Krebs Von Den Lungen 6 (KL-6) | P15941 | bronchopulmonary dysplasia | P27.1 |  | antecedent | Cord blood; Infant blood | 23523392 |
| matrix metalloproteinase 9 (MMP 9) | P14780 | bronchopulmonary dysplasia | P27.1 |  | antecedent | Cord blood | 23523392 |
| monocyte chemoattractant protein 1 (MCP-1) | P13500 | bronchopulmonary dysplasia | P27.1 | clinical trial | antecedent | Infant blood | 23523392 |
| placental growth factor (PlGF) | P49763 | bronchopulmonary dysplasia | P27.1 |  | antecedent | Cord blood | 23523392 |
| platelet derived growth factor-BB isoform (PDGF-BB) | P04085,P01127 | bronchopulmonary dysplasia | P27.1 |  | antecedent | Infant blood | 23523392 |
| soluble E-selectin | P16581 | bronchopulmonary dysplasia | P27.1 | clinical trial | antecedent | Cord blood; Infant blood | 23523392 |
| soluble L-selectin | P14151 | bronchopulmonary dysplasia | P27.1 |  | antecedent | Infant blood | 23523392 |
| transforming growth factor beta 1 (TGFb1) | P01137 | bronchopulmonary dysplasia | P27.1 | clinical trial | antecedent | Infant blood | 23523392 |
| tumor necrosis factor beta (TNFb) | P01374 | bronchopulmonary dysplasia | P27.1 |  | antecedent | Infant blood | 23523392 |
| type IV collagen (C-IV) | P53420,P29400,P02462,P08572,Q14031,Q01955 | bronchopulmonary dysplasia | P27.1 |  | antecedent | Infant blood | 23523392 |
| vascular endothelial growth factor (VEGF) | P15692,P49765,P49767,O43915 | bronchopulmonary dysplasia | P27.1 | clinical trial | antecedent | Infant blood | 23523392 |
| tissue inhibitor of metalloproteinase (TIMP 1) |  | bronchopulmonary dysplasia | P27.1 |  | antecedent | Cord blood | 23523392 |
| B-type natriuretic peptide (BNP) |  | persistent pulmonary hypertension of the newborn | P29.3 |  | diagnostic |  | 22797141 |
| N-terminal pro-B-type natriuretic peptide (NT-pro-BNP) |  | persistent pulmonary hypertension of the newborn | P29.3 |  | diagnostic |  | 22797141 |
| B-type natriuretic peptide (BNP) |  | patent ductus arteriosus | Q25.0 |  | diagnostic |  | 22797141 |
| N-terminal pro-B-type natriuretic peptide (NT-pro-BNP) |  | patent ductus arteriosus | Q25.0 |  | diagnostic |  | 22797141 |
| HDL-cholesterol |  | Autosomal-Dominant Polycystic Kidney Disease | Q61 |  | prognostic | serum | 22846584 |
| vascular endothelial growth factor (VEGF) | P15692,P49765,P49767,O43915 | Von Hippel-Lindau disease | Q85.8 |  | marker | serum | 262 |
| B-type natriuretic peptide (BNP) |  | asphyxia | R09.0, T71 |  | prognostic |  | 22797141 |
| N-terminal pro-B-type natriuretic peptide (NT-pro-BNP) |  | asphyxia | R09.0, T71 |  | prognostic |  | 22797141 |
| IL-1Rα | P14778 | Hypoxia | R09.02 |  | diagnostic | plasma | 22732129 |
| IL-1β | P01584 | Hypoxia | R09.02 |  | diagnostic | plasma | 22732129 |
| Osteopontin (OPN) | P10451 | tumor-related hypoxia | R09.02 | clinical trial | surrogate | plasma | 173 |
| plasminogen activator inhibitor 1 (PAI-1) | P05121 | tumor-related hypoxia | R09.02 | clinical trial | surrogate | plasma | 173 |
| vascular endothelial growth factor (VEGF) | P15692,P49765,P49767,O43915 | tumor-related hypoxia | R09.02 | clinical trial | surrogate | plasma | 173 |
| calcium |  | hemodialysis | R88.0 | clinical trial | marker | serum | 20650654 |
| phosphorus |  | hemodialysis | R88.0 | clinical trial | marker | serum | 20650654 |
| potassium |  | hemodialysis | R88.0 | clinical trial | marker | serum | 20650654 |
| IL-6 | P05231 | rejection of renal transplants | T86.1 |  | diagnostic | urine;blood | 22914685 |
| soluble CD30 | P28908 | rejection of renal transplants | T86.1 |  | prognostic | plasma | 22914685 |
