# Supplementary material for: The Assessment of the Readiness of Molecular Biomarker-Based Mobile Health Technologies for Healthcare Applications
Source: Sci Rep. 2015 Dec 8;5:17854. doi: 10.1038/srep17854 (PMC4672303; doi:10.1038/srep17854)
Supplement: Supplementary Table S5 [file srep17854-s5.doc]

Supplementary Table 5: Diseases covered by non-invasive molecular biomarkers

| Disease or Disease Class | Disease ICD Code | Disease Prevalence | Biomarker Molecular Type (No of Biomarkers, No in clinical use or trial) | Biomarker Source | Feasibility of New Tech Based Biomarker Detection | Highest Biomarker Detection Sensitivity | Highest Biomarker Detection Specificity | AUC | Disease Form (Acute / Chronic) | Rare/Common | Biomarker Level in Patients | Biomarker Level in Normal Population | Biomarker Detection Limit with Respect to Biomarker Levels in Patients |
| --- | --- | --- | --- | --- | --- | --- | --- | --- | --- | --- | --- | --- | --- |
| Pulmonary tuberculosis | A15.0 | P:World(8.6 M),USA(9,945),UK(0.5 M) | P (1) | Sa |  | 81.80% | 81.40% |  | C | Ra |  |  |  |
| Sepsis | A41.9 | P:USA(660,000) | P (3) | U | ELISA |  |  |  | A/C |  | 1662.6 ± 1823.8 pg/mL | 632.6 ± 495 pg/mL | Almost within range |
| Acute hepatitis E | B17.2 | I:World(3 M) | P (8) | U | ELISA |  |  | 0.89 | A | Ra | >0 | 0 | Within range |
| HIV infection | B20 | P:World(35.3 M),USA(1.15 M),UK(2.2 M) | P (6) | U | ELISA | 94.00% | 71.00% |  | A/C | Co |  |  |  |
| HIV infection | B20 | P:World(35.3 M),USA(1.15 M),UK(2.2 M) | P (6) | U | ELISA | 94.00% | 71.00% |  | A/C | Co |  | 0.2 - 146.7 ng/mL | Within range |
| Kala-azar | B55.0 | I:World(0.5 M) | P (1) | U | ELISA |  |  |  | C | Ra |  |  |  |
| Upper gastrointestinal cancer | C15-C26 | I:USA(Esophageal Cancer 17,990) | P (4) | U |  | 86.00% | 80.00% |  | C | Ra |  |  |  |
| Gastric cancer | C16 | I:World(951,594),USA(21,155),UK(139,667) | P (1) | U |  | 79.00% | 100.00% | 0.97 | C | Ra |  |  |  |
| Colorectal cancer | C18-C21 | I:World(1360602),USA(134,349),UK(447,136) | P (1), Sm (1) | F, U | ELISA | 73.0-83.0% | 82.00% |  | C |  | 0.3 - 620 U/mL | 0.1 - 17.3 U/mL |  |
| Hepatocellular Carcinoma | C22.0 | I:World(782,451),USA(30,449),UK(63462) | P (2), Sm (1) | U |  | 61.00% | 92.00% |  | C | Ra |  |  |  |
| Cholangiocarcinoma | C22.1 | I:USA(1.67 in 100,000 ) | P (1) | U |  | 83.00% | 79.00% | 0.87 | C | Ra |  |  |  |
| Lung cancer | C33-C34 | P:USA(214,226) | Sm (2) | Br |  | 84.50% | 80.00% |  | C |  |  |  |  |
| Lung cancer NSCLC type | C33-C34 | P:USA(214,226) | Sm (1, CT) | U |  |  |  |  | C |  |  |  |  |
| Oral squamous cell carcinoma | C44.02 | I:World(640,000),USA(54,000) | Sm (2), P(2) | Sa |  | 92.30% | 91.70% |  | C |  |  |  |  |
| Oral squamous cell carcinoma | C44.02 | I:World(640,000),USA(54,000) | P (1) | Sa, Sk |  |  |  |  | C |  |  |  |  |
| Breast cancer | C50 | I:World(1676633),USA(232,714),UK(464,202) | Pep (1) | U | ELISA |  |  |  | C | Co |  |  |  |
| Ovarian cancer | C56 | I:World(238,719),USA(20,874),UK(65,584) | Sm (1) | U | ELISA | 70.00% | 75.00% |  | C | Ra |  |  |  |
| Prostate cancer | C61 | I:World(1111689),USA(233,159),UK(417,137) | Sm (1, CT), Pep (1, CT) | U | ELISA |  |  |  | C | Co |  |  |  |
| Renal cell carcinoma | C64 | I:World(245,000),USA(65,000),UK(91,000) | P (12) | U | ELISA | 100.00% | 100.00% | 1 | C |  |  | 29 ± 9 ng/mL | Within range |
| Kidney cancer | C64.9 | I:World(337860),USA(58,222),UK(115,252) | P (2, CT 2) | U | ELISA | 100.00% | 100.00% | 1 | C |  |  |  |  |
| Bladder cancer | C67 | I:World(429,793),USA(68,639),UK(151,297) | P (1) | U | ELISA | 85.70% |  |  | C |  |  |  |  |
| Bladder cancer | C67 | I:World(429,793),USA(68,639),UK(151,297) | P (4) | U | ELISA |  |  |  | C |  |  |  |  |
| Malignant primary brain tumor | C71 | I:World(256,000),USA(69,720),UK(57,100) | Sm (1, CT) | U | ELISA |  |  |  | C |  |  |  |  |
| Carcinoid tumor | C75, E34.0 | I:World(12,000) | Sm (1) | U |  | 35.00% | 88.00% |  | C | Ra |  |  |  |
| Bone metastases | C79.51 |  | P (1, CT), Pep (1, CT), Sm (1, CT) | U | ELISA |  |  |  | C |  |  |  |  |
| Multiple myeloma | C90.0 | I:World(114,000),USA(24,050),UK(38,900) | P (1) | U | ELISA | 88.90% | 83.30% |  | C | Ra |  |  |  |
| Multiple myeloma | C90.0 | I:World(114,000),USA(24,050),UK(38,900) | Pep (1) | U | ELISA |  |  |  | C | Ra | 14.54 nmol/mmol | 5.04 nmol/mmol |  |
| Henoch-Schonlein purpura | D69.0 | I:World(10-22 in 100,000) | P (1) | U | ELISA |  |  |  | A |  | 32.02 ± 3.95 ng/mg | 25.31 ± 4.11 ng/mg | Within range |
| Acute graft-versus-host disease | D89.8 | I:World(5500) | P (9) | Sa, U, Sk | ELISA |  |  |  | A | Ra | 68.8 ± 10.1 ug/mL | 101.3 ± 12.7 ug/mL | Within range |
| Type 1 diabetes | E10 | P:World(11-22 M),USA(3 M),UK(112,000) | P (2) | U | ELISA |  |  |  | C | Co |  |  |  |
| Type 1 diabetes | E10 | P:World(11-22 M),USA(3 M),UK(112,000) | P (1, combi 4) | U | ELISA |  |  | 0.89 | C | Co |  |  |  |
| Diabetes | E10, E11 | P:World(), USA(29.1M), Europe() | P (2, combi 261) | U |  | ~91% | ~78% |  | C | Co |  |  |  |
| Diabetic Nephropathy | E10.2, E11.2, E12.2, E13.2, E14.2 | P:World(20% - 40% of diabetes) | P (7) | U | ELISA | 81.40% | 62.50% |  | C | Co | 27.3 ± 3.3 ng/μmol | 0-25 ug/g | Within range |
| Diabetic Nephropathy | E10.2, E11.2, E12.2, E13.2, E14.2 | P:World(20% - 40% of diabetes) | P (3) | U | ELISA |  |  |  | C | Co |  |  |  |
| Type 2 diabetes | E11 | P:World(), USA(27.85M), Europe() | P (11) | U | ELISA |  |  |  | C | Co | 56.9 ± 19.45 ug/mL | 9.7 ± 2.35 ug/mL | Within range |
| Type 2 diabetes | E11 | P:World(), USA(27.85M), Europe() | P (3) | U | ELISA |  |  |  | C | Co |  |  |  |
| Diabetes insipidus | E23.2 | I:World(3 per 100,000) | P (1) | U |  |  |  |  | A/C |  |  |  |  |
| Aldosteronism | E26.02 | P:,USA(<200,000) | P (1) | U |  |  |  |  | C | Ra |  |  |  |
| Mucopolysaccharidoses | E76 | P:USA(200) | Sm (2) | U | ELISA |  |  |  | C | Ra |  |  |  |
| Mucopolysaccharidoses | E76 | P:USA(200) | Sm (2) | U | ELISA |  |  |  | C | Ra |  |  |  |
| Idiopathic hypercalciuria(IH) | E83.52 |  | P (1) | U | ELISA |  |  |  | C |  |  |  |  |
| Cystic fibrosis | E84 | P:World(70,000),USA(30,000) | Sm (2) | Br |  | 93.80% | 69.20% |  | C | Ra |  |  |  |
| Cystic fibrosis | E84 | P:World(70,000),USA(30,000) | Sm (3) | Br | ELISA |  |  |  | C | Ra |  |  |  |
| Renal light-chain amyloidosis (AL) | E85.8 | I:,USA(1200-3200) | P (1) | U |  | 81.30% | 98.00% |  | C | Ra |  |  |  |
| Metabolic syndrome | E88.81 | P:,USA(22.9% of population) | P (1) | U |  |  |  |  | C | Co |  |  |  |
| Chronic stress | F40-F42 | P:World(40 M) | P (1, CT) | U | ELISA | 100.00% |  |  | C | Co | 70.9 ± 19.2 pg/mg | 18.8 ± 32 pg/mg | Out of range |
| Enuresis | F98.0 | P:,USA(4-4.5% of childen) | P (1) | U |  |  |  |  | C | Co |  |  |  |
| Parkinson's disease | G20 | P:World(10 M),USA(1 M),UK(6.7 M) | Sm (1) | U |  |  |  |  | C | Co |  |  |  |
| Multiple sclerosis | G35 | P:World(30 in 100 000),USA(400,000),UK(80 in 100 000) | P (1) | U | ELISA |  |  |  | A/C* |  |  |  |  |
| Obstructive sleep apnea syndrome | G47.33 | P:World(3%-7%),USA( 4% in men, 2% in women) | Sm (1) | Sa |  |  |  |  | C | Co |  |  |  |
| Encephalopathy | G93.4 |  | Sm (1), P (1) | U | ELISA | 99.00% | 97.00% |  | A |  | 0.47 ng/mL | 0.02 - 1.35 ng/mL | Out of range |
| Encephalopathy | G93.4 |  | Sm (1), P (1) | U | ELISA |  |  |  | A |  |  |  |  |
| Ocular allergy | H00-H59 | P:World(2 M) | Pep (2), P (3) | T | ELISA |  |  |  | A*/C | Ra |  |  |  |
| Ocular allergy | H00-H59 | P:World(2 M) | P (1) | T | ELISA |  |  |  | A*/C | Ra |  |  |  |
| Dry eye disease | H16.229 | P:World(4.88 M) | Pep (2, CT 1), P (19) | T | ELISA | 85.00% | 94.00% |  | C |  | >40 ng/mL | 3 - 40 ng/mL | Within range |
| Dry eye disease | H16.229 | P:World(4.88 M) | Pep (2), P (1) | T | ELISA |  |  |  | C |  |  |  |  |
| Glaucoma | H40-H42 | P:World(60 M),USA(2.2 M) | P (1) | Eye |  |  |  |  | A/C* | Co |  |  |  |
| Chronic renovascular hypertension | I15.0 |  | P (1) | U | ELISA |  |  |  | C |  |  |  |  |
| Pulmonary arterial hypertension | I27.0, I27.2 | P:,USA(260,000) | Sm (1) | Br |  |  |  |  | A*/C |  |  |  |  |
| Atrial fibrillation | I48 | P:World(33.5 M),USA(2.66 M) | Sm (1) | U |  |  |  |  | A/C | Co |  |  |  |
| Heart failure | I50 | P:World(26 M),USA(5.1 M),UK(3.5 M) | P (1, CU), Sm (1) | Hair, U |  |  |  |  | A/C | Co |  |  |  |
| Heart failure | I50 | P:World(26 M),USA(5.1 M),UK(3.5 M) | P (2) | U |  |  |  |  | A/C | Co |  |  |  |
| Kidney function decline with atherosclerosis | I75.81 |  | P (1) | U |  |  |  |  | C |  |  |  |  |
| Deep vein thrombosis(DVT) and pulmonary embolism(PE) | I82.4,I82.5 | P:USA(300,000-600,000) | P (1) | U | ELISA | 100.00% | 85.00% | 0.97 | C |  | 78.4 ± 35.2 ng/ml | 2.2 ± 0.4 ng/mL | Within range |
| Chronic obstructive pulmonary disease | J40-J44, J47 | P:World(64 M),USA(12.7 M),UK(1.5-10% of population) | Sm (3) | Br, U |  |  |  |  | C | Co |  |  |  |
| Chronic obstructive pulmonary disease | J40-J44, J47 | P:World(64 M),USA(12.7 M),UK(1.5-10% of population) | Sm (2) | Br |  |  |  |  | C | Co |  |  |  |
| Asthma | J45 | P:World(235 M),USA(25 M),UK(30 M) | Sm (4), P (1), Cell (2) | Br, Sp | ELISA | 73.6-86.0% | 88.00% |  | C |  |  |  |  |
| Asthma | J45 | P:World(235 M),USA(25 M),UK(30 M) | Sm (2), P (1) Sm+P (1, CT), Cell (1), Sm+Cell (1) | Br, Sp | ELISA |  |  |  | C |  |  |  |  |
| Fibrosing alveolitis | J84.1 | P:USA(14-27.9 in 100,000),UK(1.25-23.4 per 100,000) | Sm (1) | Br |  |  |  |  | C*/A | Ra |  |  |  |
| Dental caries | K02 | P:World(23.7% adult),USA(15.6% children),UK( 59% population) | P (1), Pep (1) | Sa |  |  |  |  | C | Co |  |  |  |
| Acute appendicitis | K35-K37 | I:USA(680,000) | P (9) | U |  | 95.00% | 100.00% |  | A*/C | Co | 0.9 - 19.3 ug/mL | 0.1 - 0.8 ug/mL | Within range |
| Acute appendicitis | K35-K37 | I:USA(680,000) | P (2) | U | ELISA | ~82% | ~68% | 0.8 | A*/C | Co |  |  |  |
| Crohn's disease | K50 | P:World(0.1-16 in 100,000 ) | P (2) | U |  |  |  |  | C | Ra |  |  |  |
| Inflammatory Bowel Disease | K50,K51 | P:World( 0.396% population),USA(1.4 M),UK(2.5-3 M) | P (12, CU 2), Sm (1) | Br, F | ELISA | 80-98%, 94% | 82-96%, 76% |  | C | Co | 2.45 ± 1.15 ng/mg | 0.006 ± 0.03 ng/mg |  |
| Inflammatory Bowel Disease | K50,K51 | P:World( 0.396% population),USA(1.4 M),UK(2.5-3 M) | P (16, CU 2) | F | ELISA | 80-90%, 70-100% | 82-83%, 44-100% |  | C | Co |  | 8 - 213 ng/mg |  |
| Inflammatory Bowel Disease | K50,K51 | P:World( 0.396% population),USA(1.4 M),UK(2.5-3 M) | P (2) | F | ELISA |  |  |  | C | Co |  |  |  |
| Acute pancreatitis | K85 | I:USA(32-44 in 100,000) | P (8) | U | ELISA | 100.00% | 96.00% |  | A |  | 2000 ng/mL | 50 ng/mL | Within range |
| Acute pancreatitis | K85 | I:USA(32-44 in 100,000) | P (11) | U | ELISA | 91.70% | 89.70% | 0.81 | A |  |  |  |  |
| Pancreatitis | K85, K86.0-K86.1 | I:USA(13-45 acute + 5-12 xhronic in 100,000) | P (2) | U |  | 81.00% | 97.00% |  | A/C |  | 29 - 10,700 ng/mL | 0.1 - 3390 ng/mL | Within range |
| Psoriasis | L40 | P:World(125 M),USA(7.5 M),UK(11 M) | P (2), miR (4), cell (1) | Sk | ELISA |  |  |  | C | Co |  |  |  |
| Arthritis | M00-M25 | P:World(1% of population),USA(52.5 M) | P (17) | U |  | ~85% | ~100% |  | C | Co | 191.7 - 313.4 ng/mmol | 129.25 - 486.85 ng/mmol | Within range |
| Arthritis | M00-M25 | P:World(1% of population),USA(52.5 M) | P (1) | U | ELISA |  |  |  | C | Co |  |  |  |
| Osteoarthritis | M15-M19, M47 | P:World(26.9 M) | P (3), Sm (1), Pep (1), Modified Pep (2, CT 1) | U | ELISA | 74.60% | 85.70% |  | C | Co | 191.4 pM | 144.4 pM | Almost within range |
| Osteoarthritis | M15-M19,M47 | P:World(26.9 M) | Sm (1), Pep (3), Modified Pep (2) | U |  |  |  |  | C | Co |  |  |  |
| Knee osteoarthritis | M17 |  | P (1) | U | ELISA |  |  |  | C |  |  |  |  |
| Kawasaki disease | M30.3 | P:USA(9-19 in 100,000 children ?5 years) | P (14) | U | ELISA | ~92% | ~95% | 0.98 | A | Ra | 50.2 ± 24.4 ng/mL | 5.6 ± 5.7 ng/mL | Within range |
| Systemic lupus erytematosus | M32 | P:USA(161,000-322,000) | P (2) | U | ELISA |  |  |  | C |  |  |  |  |
| Systemic lupus erytematosus | M32 | P:USA(161,000-322,000) | P (3) | U | ELISA | ~70% | ~89% | 0.76 | C |  |  |  |  |
| Lupus nephritis | M32.1, N08.5 | | P (3) | U | ELISA | 88.50% | 46.30% | 0.73 | C |  | ~830 ± 100 ng/mL | ~380 ± 130 ng/mL | Within range |
| Lupus nephritis | M32.1, N08.5 | | P (5, combi 3), miR (2) | U | ELISA | 100.00% | 81.00% | 0.92 | C |  |  |  |  |
| Focal segmental glomerulosclerosis (FSGS) | N00.1,N01.1,N02.1,N03.1,N04.1,N05.1,N06.1,N07.1 | P:USA(70,000) | P (1) | U | ELISA |  |  |  | C | Ra | 2.91 - 8.03 ng/mg | 0 ng/mg | Almost within range |
| Crescentic Glomerulonephritis(GN) | N00.7,N01.7,N02.7,N03.7,N04.7,N05.7,N06.7,N07.7 | | P (1) | U | ELISA | 91.70% | 90.20% |  | A |  |  |  |  |
| Membranous nephropathy | N02.2 | I:USA(2000) | P (1) | U | ELISA | 86.00% |  |  | C | Ra |  |  |  |
| IgA nephritis | N02.8 | P:USA(1 in 100,000) | P (65) | U | ELISA | 81.70% | 73.40% |  | C | Ra | >0 | 0 | Within range |
| IgA nephritis | N02.8 | P:USA(1 in 100,000) | P (9) | U | ELISA | 100.00% | 100.00% | 1 | C | Ra |  |  |  |
| IgA nephritis | N02.8 | P:USA(1 in 100,000) | P (8) | U | ELISA |  |  |  | C | Ra |  |  |  |
| Chronic glomerulonephritis | N03.2 |  | P (1) | U | ELISA | 87.50% | 90.50% | 0.95 | C |  | 76.58 ± 17.3 ng/mg | 20.12 ± 3.69 ng/mg | Within range |
| Nephrotic syndrome | N04 | P:USA(15 in 100,000 children) | P (6) | U |  |  |  |  | A | Ra |  |  |  |
| Nephrotic syndrome | N04 | P:USA(15 in 100,000 children) | P (1) | U |  |  |  |  | A | Ra |  |  |  |
| Nephrotic syndrome | N04 | P:USA(15 in 100,000 children) | P (1) | U |  |  |  |  | A | Ra |  |  |  |
| Minimal change nephropathy | N04.0 | P:USA(1.5-2.3 per 100,000) | P (1) | U | ELISA |  |  |  | A | Ra |  |  |  |
| Idiopathic nephrotic syndrome (INS) | N04.9 |  | P (1) | U |  |  |  |  | A |  |  |  |  |
| Idiopathic nephrotic syndrome (INS) | N04.9 |  | P (1) | U |  |  |  |  | A |  |  |  |  |
| Vesicoureteral Reflux | N13.7 | P:World(1%-2% of children) | P (2) | U | ELISA | 67.00% | 85.00% | 0.77 | C |  | 509.8 - 5912 pg/mL | 29.5 - 993.6 pg/mL | Almost within range |
| Vesicoureteral Reflux | N13.7 | P:World(1%-2% of children) | P (2) | U | ELISA | 81.20% | 85.00% | 0.88 | C |  | 1994.5 - 5922.5 pg/mL | 412 - 5908 pg/mL | Within range |
| Contrast-induced nephropathy | N14.1 | P:World(<2% of population) | P (18) | U | ELISA | 73.00% | 100.00% | 0.92 | A |  | 135 ± 32 ng/mL | 11.6 ± 2 ng/mL | Within range |
| Contrast-induced nephropathy | N14.1 | P:World(<2% of population) | P (2) | U | ELISA | 80.00% | 75.00% |  | A |  |  |  |  |
| Balkan endemic nephropathy | N15.0 | P:World(0.5-4.4% of population),USA(<200,000) | P (4) | U | ELISA | 72.30% | 84.40% | 0.83 | C | Ra | 3.0 - 49.8 mg/mmol | 1.7 - 5.5 mg/mmol | Within range |
| Balkan endemic nephropathy | N15.0 | P:World(0.5-4.4% of population),USA(<200,000) | P (1) | U | ELISA |  |  |  | C | Ra |  |  |  |
| Acute kidney injury | N17 | P:USA(1-7.1% of all hospital admissions) | P (15, CU 2, CT 3) | U | ELISA | 69-100%, 73-100% | 85-98% |  | A | Co | 50.5–205.9 ng/mL | 5.7–17.7 ng/mL | Within range |
| Acute kidney injury | N17 | P:USA(1-7.1% of all hospital admissions) | P (2, CT 1) | U | ELISA | >90% | >90% |  | A | Co | 0 - 955 pg/mL | 0 - 173 pg/mL | Out of range |
| Chronic kidney disease | N18.9 | P:World(8-16% of population),USA(20 million) | P (2) | U | ELISA |  |  |  | C | Co |  |  |  |
| Chronic kidney disease | N18.9 | P:World(8-16% of population),USA(20 million) | P (18) | U | ELISA |  |  |  | C | Co |  |  |  |
| Kidney calculi | N20.0 | P:USA(1 in 11) | P (20) | U |  |  |  |  | C | Co |  |  |  |
| Urolithiasis | N21.0-N21.9 | P:USA( 7% of women and 12% of men) | P (3) | U | ELISA | 90.00% | 68.00% |  | C | Co | 104.66 ± 159.70 pg/mg | 7.76 ± 8.90 pg/mg | Out of range |
| Urolithiasis | N21.0-N21.9 | P:USA( 7% of women and 12% of men) | P (1) | U | ELISA |  |  |  | C | Co | 104.66 ± 159.70 pg/mg | 7.76 ± 8.90 pg/mg | Out of range |
| Interstitial cystitis | N30.10, N30.11 | P:USA( 8 million women ) | P (7), Sm (2) | U | ELISA | 70.00% | 72.40% |  | C | Co | 0.25 +_ 0.1 pg/mg | 0.9 +_ 0.4 pg/mg | Out of range |
| Overactive bladder | N32.81 | P:World(33 M),USA(22 M) | Sm (1), P (4) | U | ELISA |  |  |  | C | Co | 20.609 ± 23.932 pg/mg | 1.779 ± 0.729 pg/mg | Out of range |
| Overactive bladder | N32.81 | P:World(33 M),USA(22 M) | Sm (1), P (4) | U | ELISA |  |  |  | C | Co |  |  |  |
| Urinary tract infection | N39.0 | P:USA(1 in 5 women) | P (1) | U | ELISA |  |  |  | A*/C | Co |  |  |  |
| Dents disease | N39.8 | P:World(250) | P (66) | U |  |  |  |  | C | Ra |  |  |  |
| Endometriosis | N80 | P:World(6û10% of women) | P (1) | U |  |  |  |  | C | Co |  |  |  |
| Pre-eclampsia | O11,O14 | P:USA(3-4% baby-delivery women) | P (9) | U | ELISA |  |  |  | A | Co | 2.11 mg/mL | 0.014 mg/mL | Within range after dilution |
| Pre-eclampsia | O11,O14 | P:USA(3-4% baby-delivery women) | P (4) | U | ELISA | ~56% | ~73% |  | A | Co |  |  |  |
| Bronchopulmonary dysplasia | P27.1 | I:World(12,000) | Sm (3), Pep (1, CT) | Br, U | ELISA | 50-85% | 61.1-90.0% |  | C | Ra |  |  |  |
| Necrotizing enterocolitis | P77 | P:World(1-3 in 1,000 infants) | P (3) | U | ELISA |  |  |  | A | Ra |  |  |  |
| Necrotizing enterocolitis | P77 | P:World(1-3 in 1,000 infants) | P (1) | U | ELISA |  |  |  | A | Ra |  |  |  |
| Primary ciliary dySesia | Q34.8 | P:USA(25000) | Sm (1) | Br |  |  |  |  | C | Ra |  |  |  |
| Autosomal dominant polycystic kidney disease | Q61 | P:World(12.5 million),USA(0.6 M) | Sm (1), P (5) | U | ELISA |  |  |  | C |  | 18 ng/mL | 0.31-79.8 ng/mL | Within range |
| Congenital hydronephrosis | Q62.0 |  | P (1) | U | ELISA | ~85% | ~90% | 0.86 | A/C |  |  |  |  |
| Ureteropelvic junction obstruction | Q62.11 | P:World( 0.5-1 in 1000 newborns) | P (36) | U |  |  |  |  | C | Ra |  |  |  |
| Traumatic brain injury (TBI) | S06 | P:USA(823.7 in 100,000) | P (1) | U | ELISA | 90.00% | 62.80% |  | A/C | Co | 0.025 ng/mL | 0.02-1.35 ng/mL | Out of range |
| Rejection of renal transplants | T86.1 |  | P (5), P+Pep (1) | U | ELISA | 80-92%, 63-100% | 77-83%, 63-98% |  | A |  |  |  |  |
| Rejection of renal transplants | T86.1 |  | P (1) | U | ELISA | 84-87% | 95-96% |  | A |  |  |  |  |
