# Supplementary material for: The Assessment of the Readiness of Molecular Biomarker-Based Mobile Health Technologies for Healthcare Applications
Source: Sci Rep. 2015 Dec 8;5:17854. doi: 10.1038/srep17854 (PMC4672303; doi:10.1038/srep17854)
Supplement: Supplementary Table S6 [file srep17854-s6.doc]

Supplementary Table S6: List of evaluated literature.

Part A: 253 review and research papers on biomarkers

1: Pathologists' Guideline Recommendations for Immunohistochemical Testing of

Estrogen and Progesterone Receptors in Breast Cancer. Breast Care (Basel).

2010;5(3):185-187. Epub 2010 Jun 8. PubMed PMID: 21049068; PubMed Central PMCID:

PMC2931060.

2: Abd-Elkareem MI, Al Tamimy HM, Khamis OA, Abdellatif SS, Hussein MR. Increased

urinary levels of the leukocyte adhesion molecules ICAM-1 and VCAM-1 in human

lupus nephritis with advanced renal histological changes: preliminary findings.

Clin Exp Nephrol. 2010 Dec;14(6):548-57. doi: 10.1007/s10157-010-0322-z. Epub

2010 Aug 17. PubMed PMID: 20714774.

3: Abdalla MA, Haj-Ahmad Y. Promising Urinary Protein Biomarkers for the Early

Detection of Hepatocellular Carcinoma among High-Risk Hepatitis C Virus Egyptian

Patients. J Cancer. 2012;3:390-403. doi: 10.7150/jca.4280. Epub 2012 Sep 18.

PubMed PMID: 23074380; PubMed Central PMCID: PMC3471080.

4: Adiyanti SS, Loho T. Acute Kidney Injury (AKI) biomarker. Acta Med Indones.

2012 Jul;44(3):246-55. Review. PubMed PMID: 22983082.

5: Allgood VE, Powell-Oliver FE, Cidlowski JA. Vitamin B6 influences

glucocorticoid receptor-dependent gene expression. J Biol Chem. 1990 Jul

25;265(21):12424-33. PubMed PMID: 2373699.

6: Ariga T, Kusunoki S, Asano K, Oshima M, Asano M, Mannen T, Yu RK. Localization

of sulfated glucuronyl glycolipids in human dorsal root and sympathetic ganglia.

Brain Res. 1990 Jun 11;519(1-2):57-64. PubMed PMID: 2204469.

7: Bachorzewska-Gajewska H, Malyszko J, Sitniewska E, Malyszko JS, Dobrzycki S.

Neutrophil-gelatinase-associated lipocalin and renal function after percutaneous

coronary interventions. Am J Nephrol. 2006;26(3):287-92. Epub 2006 Jun 13. PubMed

PMID: 16772710.

8: Barbado J, Martin D, Vega L, Almansa R, Gonçalves L, Nocito M, Jimeno A, Ortiz

de Lejarazu R, Bermejo-Martin JF. MCP-1 in urine as biomarker of disease activity

in Systemic Lupus Erythematosus. Cytokine. 2012 Nov;60(2):583-6. doi:

10.1016/j.cyto.2012.07.009. Epub 2012 Aug 3. PubMed PMID: 22857869.

9: Bautista DS, Denstedt J, Chambers AF, Harris JF. Low-molecular-weight variants

of osteopontin generated by serine proteinases in urine of patients with kidney

stones. J Cell Biochem. 1996 Jun 1;61(3):402-9. PubMed PMID: 8761944.

10: Bazzi C, Petrini C, Rizza V, Arrigo G, Napodano P, Paparella M, D'Amico G.

Urinary N-acetyl-beta-glucosaminidase excretion is a marker of tubular cell

dysfunction and a predictor of outcome in primary glomerulonephritis. Nephrol

Dial Transplant. 2002 Nov;17(11):1890-6. PubMed PMID: 12401843.

11: Bennett MR, Ravipati N, Ross G, Nguyen MT, Hirsch R, Beekman RH, Rovner L,

Devarajan P. Using proteomics to identify preprocedural risk factors for contrast

induced nephropathy. Proteomics Clin Appl. 2008;2(7-8):1058-1064. PubMed PMID:

18953418; PubMed Central PMCID: PMC2572074.

12: Bhandari A, Bhandari V. Biomarkers in bronchopulmonary dysplasia. Paediatr

Respir Rev. 2013 Sep;14(3):173-9. doi: 10.1016/j.prrv.2013.02.008. Epub 2013 Mar

21. Review. PubMed PMID: 23523392.

13: Bhardwaj N, Ghaffari G. Biomarkers for eosinophilic esophagitis: a review.

Ann Allergy Asthma Immunol. 2012 Sep;109(3):155-9. doi:

10.1016/j.anai.2012.06.014. Epub 2012 Jul 12. Review. PubMed PMID: 22920068.

14: Bhatia A, Dey P, Kumar Y, Gautam U, Kakkar N, Srinivasan R, Nijhawan R.

Expression of cytokeratin 20 in urine cytology smears: a potential marker for the

detection of urothelial carcinoma. Cytopathology. 2007 Apr;18(2):84-6. PubMed

PMID: 17397492.

15: Bhide AA, Cartwright R, Khullar V, Digesu GA. Biomarkers in overactive

bladder. Int Urogynecol J. 2013 Jul;24(7):1065-72. doi:

10.1007/s00192-012-2027-1. Epub 2013 Jan 12. Review. Erratum in: Int Urogynecol

J. 2013 Oct;24(10):1775-6. PubMed PMID: 23314226.

16: Bianco C, Castro NP, Baraty C, Rollman K, Held N, Rangel MC, Karasawa H,

Gonzales M, Strizzi L, Salomon DS. Regulation of human Cripto-1 expression by

nuclear receptors and DNA promoter methylation in human embryonal and breast

cancer cells. J Cell Physiol. 2013 Jun;228(6):1174-88. doi: 10.1002/jcp.24271.

PubMed PMID: 23129342; PubMed Central PMCID: PMC3573215.

17: Bowser R, Turner MR, Shefner J. Biomarkers in amyotrophic lateral sclerosis:

opportunities and limitations. Nat Rev Neurol. 2011 Oct 11;7(11):631-8. doi:

10.1038/nrneurol.2011.151. Review. PubMed PMID: 21989244.

18: Braido F, Bagnasco D, Scichilone N, Santus P, Solidoro P, Di Marco F, Corsico

A, Canonica GW. Biomarkers in obstructive respiratory diseases: an update.

Panminerva Med. 2012 Jun;54(2):119-27. Review. PubMed PMID: 22525566.

19: Braun MS, Richman SD, Thompson L, Daly CL, Meade AM, Adlard JW, Allan JM,

Parmar MK, Quirke P, Seymour MT. Association of molecular markers with toxicity

outcomes in a randomized trial of chemotherapy for advanced colorectal cancer:

the FOCUS trial. J Clin Oncol. 2009 Nov 20;27(33):5519-28. doi:

10.1200/JCO.2008.21.6283. Epub 2009 Oct 26. PubMed PMID: 19858398.

20: Braun MS, Richman SD, Quirke P, Daly C, Adlard JW, Elliott F, Barrett JH,

Selby P, Meade AM, Stephens RJ, Parmar MK, Seymour MT. Predictive biomarkers of

chemotherapy efficacy in colorectal cancer: results from the UK MRC FOCUS trial.

J Clin Oncol. 2008 Jun 1;26(16):2690-8. doi: 10.1200/JCO.2007.15.5580. Erratum

in: J Clin Oncol. 2008 Sep 10;26(26):4363. PubMed PMID: 18509181.

21: Brown PF 3rd, Larsen CP, Symbas PN. Management of the asymptomatic patient

with a stab wound to the chest. South Med J. 1991 May;84(5):591-3. PubMed PMID:

2035078.

22: Bröker ME, Lalmahomed ZS, Roest HP, van Huizen NA, Dekker LJ, Calame W,

Verhoef C, Ijzermans JN, Luider TM. Collagen peptides in urine: a new promising

biomarker for the detection of colorectal liver metastases. PLoS One. 2013 Aug

16;8(8):e70918. doi: 10.1371/journal.pone.0070918. eCollection 2013. PubMed PMID:

23976965; PubMed Central PMCID: PMC3745414.

23: Buhimschi CS, Baumbusch MA, Dulay AT, Lee S, Wehrum M, Zhao G, Bahtiyar MO,

Pettker CM, Ali UA, Funai EF, Buhimschi IA. The role of urinary soluble endoglin

in the diagnosis of pre-eclampsia: comparison with soluble fms-like tyrosine

kinase 1 to placental growth factor ratio. BJOG. 2010 Feb;117(3):321-30. doi:

10.1111/j.1471-0528.2009.02434.x. Epub 2009 Nov 26. PubMed PMID: 19943826.

24: Buhimschi IA, Zhao G, Funai EF, Harris N, Sasson IE, Bernstein IM, Saade GR,

Buhimschi CS. Proteomic profiling of urine identifies specific fragments of

SERPINA1 and albumin as biomarkers of preeclampsia. Am J Obstet Gynecol. 2008

Nov;199(5):551.e1-16. doi: 10.1016/j.ajog.2008.07.006. PubMed PMID: 18984079;

PubMed Central PMCID: PMC2679897.

25: Cameron D, Casey M, Oliva C, Newstat B, Imwalle B, Geyer CE. Lapatinib plus

capecitabine in women with HER-2-positive advanced breast cancer: final survival

analysis of a phase III randomized trial. Oncologist. 2010;15(9):924-34. doi:

10.1634/theoncologist.2009-0181. Epub 2010 Aug 24. PubMed PMID: 20736298; PubMed

Central PMCID: PMC3228041.

26: Cangemi R, Celestini A, Calvieri C, Carnevale R, Pastori D, Nocella C,

Vicario T, Pignatelli P, Violi F. Different behaviour of NOX2 activation in

patients with paroxysmal/persistent or permanent atrial fibrillation. Heart. 2012

Jul;98(14):1063-6. doi: 10.1136/heartjnl-2012-301952. Epub 2012 May 23. PubMed

PMID: 22628539.

27: Canter MP, Graham CA, Heit MH, Blackwell LS, Wilkey DW, Klein JB, Merchant

ML. Proteomic techniques identify urine proteins that differentiate patients with

interstitial cystitis from asymptomatic control subjects. Am J Obstet Gynecol.

2008 May;198(5):553.e1-6. doi: 10.1016/j.ajog.2008.01.052. PubMed PMID: 18455532.

28: Cazzato S, Ridolfi L, Bernardi F, Faldella G, Bertelli L. Lung function

outcome at school age in very low birth weight children. Pediatr Pulmonol. 2013

Aug;48(8):830-7. doi: 10.1002/ppul.22676. Epub 2012 Nov 5. PubMed PMID: 23129340.

29: Chakraborty JB, Oakley F, Walsh MJ. Mechanisms and biomarkers of apoptosis in

liver disease and fibrosis. Int J Hepatol. 2012;2012:648915. doi:

10.1155/2012/648915. Epub 2012 Apr 9. PubMed PMID: 22567408; PubMed Central

PMCID: PMC3332069.

30: Chan SL, Mo FK, Johnson PJ, Hui EP, Ma BB, Ho WM, Lam KC, Chan AT, Mok TS,

Yeo W. New utility of an old marker: serial alpha-fetoprotein measurement in

predicting radiologic response and survival of patients with hepatocellular

carcinoma undergoing systemic chemotherapy. J Clin Oncol. 2009 Jan

20;27(3):446-52. doi: 10.1200/JCO.2008.18.8151. Epub 2008 Dec 8. PubMed PMID:

19064965.

31: Charakida M, Masi S, Tousoulis D. Functional, genetic and biochemical

biomarkers of peripheral arterial disease. Curr Med Chem. 2012;19(16):2497-503.

Review. PubMed PMID: 22489720.

32: Chowdhury P, Choudhary R, Maisel A. The appropriate use of biomarkers in

heart failure. Med Clin North Am. 2012 Sep;96(5):901-13. doi:

10.1016/j.mcna.2012.07.002. Review. PubMed PMID: 22980054.

33: Christiansen MS, Hesse D, Ekbom P, Hesse U, Damm P, Hommel E, Feldt-Rasmussen

B, Mathiesen E. Increased urinary orosomucoid excretion predicts preeclampsia in

pregnant women with pregestational type 1 diabetes. Diabetes Res Clin Pract. 2010

Jul;89(1):16-21. doi: 10.1016/j.diabres.2010.03.018. Epub 2010 Apr 14. PubMed

PMID: 20392509.

34: Clarke LA, Winchester B, Giugliani R, Tylki-Szymańska A, Amartino H.

Biomarkers for the mucopolysaccharidoses: discovery and clinical utility. Mol

Genet Metab. 2012 Aug;106(4):395-402. doi: 10.1016/j.ymgme.2012.05.003. Epub 2012

May 14. Review. PubMed PMID: 22658917.

35: Cobo M, Isla D, Massuti B, Montes A, Sanchez JM, Provencio M, Viñolas N,

Paz-Ares L, Lopez-Vivanco G, Muñoz MA, Felip E, Alberola V, Camps C, Domine M,

Sanchez JJ, Sanchez-Ronco M, Danenberg K, Taron M, Gandara D, Rosell R.

Customizing cisplatin based on quantitative excision repair cross-complementing 1

mRNA expression: a phase III trial in non-small-cell lung cancer. J Clin Oncol.

2007 Jul 1;25(19):2747-54. PubMed PMID: 17602080.

36: Colligris B, Crooke A, Gasull X, Escribano J, Herrero-Vanrell R,

Benitez-del-Castillo JM, García-Feijoo J, Pintor J. Recent patents and

developments in glaucoma biomarkers. Recent Pat Endocr Metab Immune Drug Discov.

2012 Sep;6(3):224-34. Review. PubMed PMID: 22827637.

37: Cutillas PR, Chalkley RJ, Hansen KC, Cramer R, Norden AG, Waterfield MD,

Burlingame AL, Unwin RJ. The urinary proteome in Fanconi syndrome implies

specificity in the reabsorption of proteins by renal proximal tubule cells. Am J

Physiol Renal Physiol. 2004 Sep;287(3):F353-64. Epub 2004 May 12. PubMed PMID:

15140760.

38: De Serres SA, Varghese JC, Levin A. Biomarkers in native and transplant

kidneys: opportunities to improve prediction of outcomes in chronic kidney

disease. Curr Opin Nephrol Hypertens. 2012 Nov;21(6):619-27. doi:

10.1097/MNH.0b013e32835846e3. Review. PubMed PMID: 22914685.

39: del Palacio M, Romero S, Casado JL. The use of biomarkers for assessing

HAART-associated renal toxicity in HIV-infected patients. Curr HIV Res. 2012

Sep;10(6):521-31. Review. PubMed PMID: 22716111.

40: Dobson R, Miller RF, Palmer HE, Feldmann M, Thompson EJ, Thompson AJ, Miller

DH, Giovannoni G. Increased urinary free immunoglobulin light chain excretion in

patients with multiple sclerosis. J Neuroimmunol. 2010 Mar 30;220(1-2):99-103.

doi: 10.1016/j.jneuroim.2010.01.012. Epub 2010 Feb 19. PubMed PMID: 20171744.

41: Dong X, Wang G, Zhang G, Ni Z, Suo J, Cui J, Cui A, Yang Q, Xu Y, Li F. The

endothelial lipase protein is promising urinary biomarker for diagnosis of

gastric cancer. Diagn Pathol. 2013 Mar 19;8:45. doi: 10.1186/1746-1596-8-45.

PubMed PMID: 23510199; PubMed Central PMCID: PMC3621381.

42: Dutheil F, Trousselard M, Perrier C, Lac G, Chamoux A, Duclos M, Naughton G,

Mnatzaganian G, Schmidt J. Urinary interleukin-8 is a biomarker of stress in

emergency physicians, especially with advancing age--the JOBSTRESS* randomized

trial. PLoS One. 2013 Aug 19;8(8):e71658. doi: 10.1371/journal.pone.0071658.

eCollection 2013. PubMed PMID: 23977105; PubMed Central PMCID: PMC3747272.

43: Eirin A, Gloviczki ML, Tang H, Rule AD, Woollard JR, Lerman A, Textor SC,

Lerman LO. Chronic renovascular hypertension is associated with elevated levels

of neutrophil gelatinase-associated lipocalin. Nephrol Dial Transplant. 2012

Nov;27(11):4153-61. doi: 10.1093/ndt/gfs370. Epub 2012 Aug 23. PubMed PMID:

22923545; PubMed Central PMCID: PMC3616756.

44: El Shamieh S, Visvikis-Siest S. Genetic biomarkers of hypertension and future

challenges integrating epigenomics. Clin Chim Acta. 2012 Dec 24;414:259-65. doi:

10.1016/j.cca.2012.09.018. Epub 2012 Sep 23. Review. PubMed PMID: 23010416.

45: Enderby B, Smith D, Carroll W, Lenney W. Hydrogen cyanide as a biomarker for

Pseudomonas aeruginosa in the breath of children with cystic fibrosis. Pediatr

Pulmonol. 2009 Feb;44(2):142-7. doi: 10.1002/ppul.20963. PubMed PMID: 19148935.

46: Enríquez-de-Salamanca A, Bonini S, Calonge M. Molecular and cellular

biomarkers in dry eye disease and ocular allergy. Curr Opin Allergy Clin Immunol.

2012 Oct;12(5):523-33. doi: 10.1097/ACI.0b013e328357b488. Review. PubMed PMID:

22895048.

47: Evennett NJ, Hall NJ, Pierro A, Eaton S. Urinary intestinal fatty

acid-binding protein concentration predicts extent of disease in necrotizing

enterocolitis. J Pediatr Surg. 2010 Apr;45(4):735-40. doi:

10.1016/j.jpedsurg.2009.09.024. PubMed PMID: 20385280.

48: Everhart JE, Wright EC. Association of γ-glutamyl transferase (GGT) activity

with treatment and clinical outcomes in chronic hepatitis C (HCV). Hepatology.

2013 May;57(5):1725-33. doi: 10.1002/hep.26203. Epub 2013 Apr 5. PubMed PMID:

23258530; PubMed Central PMCID: PMC3624035.

49: Finn RS, Gagnon R, Di Leo A, Press MF, Arbushites M, Koehler M. Prognostic

and predictive value of HER2 extracellular domain in metastatic breast cancer

treated with lapatinib and paclitaxel in a randomized phase III study. J Clin

Oncol. 2009 Nov 20;27(33):5552-8. doi: 10.1200/JCO.2008.21.1763. Epub 2009 Oct

26. PubMed PMID: 19858400.

50: Finn RS, Press MF, Dering J, Arbushites M, Koehler M, Oliva C, Williams LS,

Di Leo A. Estrogen receptor, progesterone receptor, human epidermal growth factor

receptor 2 (HER2), and epidermal growth factor receptor expression and benefit

from lapatinib in a randomized trial of paclitaxel with lapatinib or placebo as

first-line treatment in HER2-negative or unknown metastatic breast cancer. J Clin

Oncol. 2009 Aug 20;27(24):3908-15. doi: 10.1200/JCO.2008.18.1925. Epub 2009 Jul

20. PubMed PMID: 19620495; PubMed Central PMCID: PMC2799151.

51: Finne P, Auvinen A, Määttänen L, Tammela TL, Ruutu M, Juusela H, Martikainen

P, Hakama M, Stenman UH. Diagnostic value of free prostate-specific antigen among

men with a prostate-specific antigen level of <3.0 microg per liter. Eur Urol.

2008 Aug;54(2):362-70. Epub 2007 Nov 5. PubMed PMID: 18006214.

52: Flint RS, Phillips AR, Farrant GJ, McKay D, Buchanan CM, Cooper GS, Windsor

JA. Probing the urinary proteome of severe acute pancreatitis. HPB (Oxford).

2007;9(6):447-55. doi: 10.1080/13651820701721744. PubMed PMID: 18345293; PubMed

Central PMCID: PMC2215359.

53: Freeston JE, Garnero P, Wakefield RJ, Hensor EM, Conaghan PG, Emery P.

Urinary type II collagen C-terminal peptide is associated with synovitis and

predicts structural bone loss in very early inflammatory arthritis. Ann Rheum

Dis. 2011 Feb;70(2):331-3. doi: 10.1136/ard.2010.129304. Epub 2010 Oct 26. PubMed

PMID: 20980286.

54: Freynhofer MK, Tajsić M, Wojta J, Huber K. Biomarkers in acute coronary

artery disease. Wien Med Wochenschr. 2012 Nov;162(21-22):489-98. doi:

10.1007/s10354-012-0148-2. Epub 2012 Nov 10. Review. PubMed PMID: 23143510.

55: Fu CP, Lee IT, Sheu WH, Lee WJ, Liang KW, Lee WL, Lin SY. The levels of

circulating and urinary monocyte chemoattractant protein-1 are associated with

chronic renal injury in obese men. Clin Chim Acta. 2012 Oct 9;413(19-20):1647-51.

doi: 10.1016/j.cca.2012.05.008. Epub 2012 May 15. PubMed PMID: 22609259.

56: Fu WJ, Li BL, Wang SB, Chen ML, Deng RT, Ye CQ, Liu L, Fang AJ, Xiong SL, Wen

S, Tang HH, Chen ZX, Huang ZH, Peng LF, Zheng L, Wang Q. Changes of the tubular

markers in type 2 diabetes mellitus with glomerular hyperfiltration. Diabetes Res

Clin Pract. 2012 Jan;95(1):105-9. doi: 10.1016/j.diabres.2011.09.031. Epub 2011

Oct 20. PubMed PMID: 22015481.

57: Fu WJ, Xiong SL, Fang YG, Wen S, Chen ML, Deng RT, Zheng L, Wang SB, Pen LF,

Wang Q. Urinary tubular biomarkers in short-term type 2 diabetes mellitus

patients: a cross-sectional study. Endocrine. 2012 Feb;41(1):82-8. doi:

10.1007/s12020-011-9509-7. Epub 2011 Jul 21. PubMed PMID: 21779943.

58: Fuchs CS, Goldberg RM, Sargent DJ, Meyerhardt JA, Wolpin BM, Green EM, Pitot

HC, Pollak M. Plasma insulin-like growth factors, insulin-like binding protein-3,

and outcome in metastatic colorectal cancer: results from intergroup trial N9741.

Clin Cancer Res. 2008 Dec 15;14(24):8263-9. doi: 10.1158/1078-0432.CCR-08-0480.

Epub 2008 Dec 10. PubMed PMID: 19073970; PubMed Central PMCID: PMC2855207.

59: Galimberti D, Scarpini E. Clinical phenotypes and genetic biomarkers of FTLD.

J Neural Transm. 2012 Jul;119(7):851-60. doi: 10.1007/s00702-012-0804-0. Epub

2012 Apr 19. Review. PubMed PMID: 22527778.

60: Ge Y, Wang TJ. Circulating, imaging, and genetic biomarkers in cardiovascular

risk prediction. Trends Cardiovasc Med. 2011 May;21(4):105-12. doi:

10.1016/j.tcm.2012.03.007. Review. PubMed PMID: 22681965; PubMed Central PMCID:

PMC3376748.

61: Gidwani K, Picado A, Rijal S, Singh SP, Roy L, Volfova V, Andersen EW, Uranw

S, Ostyn B, Sudarshan M, Chakravarty J, Volf P, Sundar S, Boelaert M, Rogers ME.

Serological markers of sand fly exposure to evaluate insecticidal nets against

visceral leishmaniasis in India and Nepal: a cluster-randomized trial. PLoS Negl

Trop Dis. 2011 Sep;5(9):e1296. doi: 10.1371/journal.pntd.0001296. Epub 2011 Sep

13. PubMed PMID: 21931871; PubMed Central PMCID: PMC3172194.

62: Gilbert RE, Akdeniz A, Weitz S, Usinger WR, Molineaux C, Jones SE, Langham

RG, Jerums G. Urinary connective tissue growth factor excretion in patients with

type 1 diabetes and nephropathy. Diabetes Care. 2003 Sep;26(9):2632-6. PubMed

PMID: 12941731.

63: Gilligan TD, Hayes DF, Seidenfeld J, Temin S. ASCO Clinical Practice

Guideline on Uses of Serum Tumor Markers in Adult Males With Germ Cell Tumors. J

Oncol Pract. 2010 Jul;6(4):199-202. doi: 10.1200/JOP.777010. PubMed PMID:

21037873; PubMed Central PMCID: PMC2900872.

64: Goodacre S, Thokala P, Carroll C, Stevens JW, Leaviss J, Al Khalaf M,

Collinson P, Morris F, Evans P, Wang J. Systematic review, meta-analysis and

economic modelling of diagnostic strategies for suspected acute coronary

syndrome. Health Technol Assess. 2013;17(1):v-vi, 1-188. doi: 10.3310/hta17010.

Review. PubMed PMID: 23331845.

65: Guan J, Wang G, Tam LS, Kwan BC, Li EK, Chow KM, Li PK, Szeto CC. Urinary

sediment ICAM-1 level in lupus nephritis. Lupus. 2012 Oct;21(11):1190-5. doi:

10.1177/0961203312451334. Epub 2012 Jun 8. PubMed PMID: 22685016.

66: Hahn OM, Yang C, Medved M, Karczmar G, Kistner E, Karrison T, Manchen E,

Mitchell M, Ratain MJ, Stadler WM. Dynamic contrast-enhanced magnetic resonance

imaging pharmacodynamic biomarker study of sorafenib in metastatic renal

carcinoma. J Clin Oncol. 2008 Oct 1;26(28):4572-8. doi: 10.1200/JCO.2007.15.5655.

PubMed PMID: 18824708; PubMed Central PMCID: PMC2736992.

67: Han WK, Alinani A, Wu CL, Michaelson D, Loda M, McGovern FJ, Thadhani R,

Bonventre JV. Human kidney injury molecule-1 is a tissue and urinary tumor marker

of renal cell carcinoma. J Am Soc Nephrol. 2005 Apr;16(4):1126-34. Epub 2005 Mar

2. PubMed PMID: 15744000; PubMed Central PMCID: PMC1307501.

68: Hara M, Yamagata K, Tomino Y, Saito A, Hirayama Y, Ogasawara S, Kurosawa H,

Sekine S, Yan K. Urinary podocalyxin is an early marker for podocyte injury in

patients with diabetes: establishment of a highly sensitive ELISA to detect

urinary podocalyxin. Diabetologia. 2012 Nov;55(11):2913-9. doi:

10.1007/s00125-012-2661-7. Epub 2012 Aug 2. PubMed PMID: 22854890; PubMed Central

PMCID: PMC3464371.

69: Harris L, Fritsche H, Mennel R, Norton L, Ravdin P, Taube S, Somerfield MR,

Hayes DF, Bast RC Jr; American Society of Clinical Oncology. American Society of

Clinical Oncology 2007 update of recommendations for the use of tumor markers in

breast cancer. J Clin Oncol. 2007 Nov 20;25(33):5287-312. Epub 2007 Oct 22.

Review. PubMed PMID: 17954709.

70: Hartl D, Lee CG, Da Silva CA, Chupp GL, Elias JA. Novel biomarkers in asthma:

chemokines and chitinase-like proteins. Curr Opin Allergy Clin Immunol. 2009

Feb;9(1):60-6. doi: 10.1097/ACI.0b013e32831f8ee0. Review. PubMed PMID: 19532094.

71: Heeringa M, Hastings A, Yamazaki S, de Koning P. Serum biomarkers in

nonalcoholic steatohepatitis: value for assessing drug effects? Biomark Med. 2012

Dec;6(6):743-57. doi: 10.2217/bmm.12.87. Review. PubMed PMID: 23227839.

72: Helal I, Reed B, Schrier RW. Emergent early markers of renal progression in

autosomal-dominant polycystic kidney disease patients: implications for

prevention and treatment. Am J Nephrol. 2012;36(2):162-7. doi: 10.1159/000341263.

Epub 2012 Jul 26. Review. PubMed PMID: 22846584.

73: Henrotin Y, Gharbi M, Mazzucchelli G, Dubuc JE, De Pauw E, Deberg M. Fibulin

3 peptides Fib3-1 and Fib3-2 are potential biomarkers of osteoarthritis.

Arthritis Rheum. 2012 Jul;64(7):2260-7. doi: 10.1002/art.34392. PubMed PMID:

22275171.

74: Hersch SM, Rosas HD. Biomarkers to Enable the Development of Neuroprotective

Therapies for Huntington’s Disease. In: Lo DC, Hughes RE, editors. Neurobiology

of Huntington's Disease: Applications to Drug Discovery. Boca Raton (FL): CRC

Press; 2011. Chapter 11.

PubMed PMID: 21882408.

75: Herzel H, Ebeling W. Effects of noise and inhomogeneous attractors in

biochemical systems. Biomed Biochim Acta. 1990;49(8-9):941-9. PubMed PMID:

2082932.

76: Hirsch R, Dent C, Pfriem H, Allen J, Beekman RH 3rd, Ma Q, Dastrala S,

Bennett M, Mitsnefes M, Devarajan P. NGAL is an early predictive biomarker of

contrast-induced nephropathy in children. Pediatr Nephrol. 2007

Dec;22(12):2089-95. Epub 2007 Sep 14. PubMed PMID: 17874137.

77: Hofmann MA, Kiecker F, Küchler I, Kors C, Trefzer U. Serum TNF-α, B2M and

sIL-2R levels are biological correlates of outcome in adjuvant IFN-α2b treatment

of patients with melanoma. J Cancer Res Clin Oncol. 2011 Mar;137(3):455-62. doi:

10.1007/s00432-010-0900-1. Epub 2010 May 9. PubMed PMID: 20454974.

78: Hong CS, Cui J, Ni Z, Su Y, Puett D, Li F, Xu Y. A computational method for

prediction of excretory proteins and application to identification of gastric

cancer markers in urine. PLoS One. 2011 Feb 18;6(2):e16875. doi:

10.1371/journal.pone.0016875. PubMed PMID: 21365014; PubMed Central PMCID:

PMC3041827.

79: Hou H, Ge Z, Ying P, Dai J, Shi D, Xu Z, Chen D, Jiang Q. Biomarkers of deep

venous thrombosis. J Thromb Thrombolysis. 2012 Oct;34(3):335-46. Review. PubMed

PMID: 22528325.

80: Howe HS, Kong KO, Thong BY, Law WG, Chia FL, Lian TY, Lau TC, Chng HH, Leung

BP. Urine sVCAM-1 and sICAM-1 levels are elevated in lupus nephritis. Int J Rheum

Dis. 2012 Feb;15(1):13-6. doi: 10.1111/j.1756-185X.2012.01720.x. PubMed PMID:

22324942.

81: Husi H, Stephens N, Cronshaw A, MacDonald A, Gallagher I, Greig C, Fearon KC,

Ross JA. Proteomic analysis of urinary upper gastrointestinal cancer markers.

Proteomics Clin Appl. 2011 Jun;5(5-6):289-99. doi: 10.1002/prca.201000107. Epub

2011 Apr 28. PubMed PMID: 21538913.

82: Idasiak-Piechocka I, Oko A, Pawliczak E, Kaczmarek E, Czekalski S. Urinary

excretion of soluble tumour necrosis factor receptor 1 as a marker of increased

risk of progressive kidney function deterioration in patients with primary

chronic glomerulonephritis. Nephrol Dial Transplant. 2010 Dec;25(12):3948-56.

doi: 10.1093/ndt/gfq310. Epub 2010 Jun 4. PubMed PMID: 20525973.

83: Imanishi T, Akasaka T. Biomarkers associated with vulnerable atheromatous

plaque. Curr Med Chem. 2012;19(16):2588-96. Review. PubMed PMID: 22489722.

84: Iskandar HN, Ciorba MA. Biomarkers in inflammatory bowel disease: current

practices and recent advances. Transl Res. 2012 Apr;159(4):313-25. doi:

10.1016/j.trsl.2012.01.001. Epub 2012 Feb 1. Review. PubMed PMID: 22424434;

PubMed Central PMCID: PMC3308116.

85: Iwano M, Yamaguchi Y, Iwamoto T, Nakatani K, Matsui M, Kubo A, Akai Y, Mori

T, Saito Y. Urinary FSP1 is a biomarker of crescentic GN. J Am Soc Nephrol. 2012

Feb;23(2):209-14. doi: 10.1681/ASN.2011030229. Epub 2011 Nov 17. PubMed PMID:

22095943; PubMed Central PMCID: PMC3269173.

86: Jang HR, Kim SM, Lee YJ, Lee JE, Huh W, Kim DJ, Oh HY, Kim YG. The origin and

the clinical significance of urinary angiotensinogen in proteinuric IgA

nephropathy patients. Ann Med. 2012 Aug;44(5):448-57. doi:

10.3109/07853890.2011.558518. Epub 2011 Mar 3. PubMed PMID: 21366514.

87: Jang T, Uzbielo A, Sineff S, Naunheim R, Scott MG, Lewis LM. Point-of-care

urine trypsinogen testing for the diagnosis of pancreatitis. Acad Emerg Med. 2007

Jan;14(1):29-34. Epub 2006 Nov 21. PubMed PMID: 17119188.

88: Jensen LH, Lindebjerg J, Ploen J, Hansen TF, Jakobsen A. Phase II

marker-driven trial of panitumumab and chemotherapy in KRAS wild-type biliary

tract cancer. Ann Oncol. 2012 Sep;23(9):2341-6. doi: 10.1093/annonc/mds008. Epub

2012 Feb 23. PubMed PMID: 22367707.

89: Jim B, Santos J, Spath F, Cijiang He J. Biomarkers of diabetic nephropathy,

the present and the future. Curr Diabetes Rev. 2012 Sep;8(5):317-28. Review.

PubMed PMID: 22698077.

90: Johnson CD, Lempinen M, Imrie CW, Puolakkainen P, Kemppainen E, Carter R,

McKay C. Urinary trypsinogen activation peptide as a marker of severe acute

pancreatitis. Br J Surg. 2004 Aug;91(8):1027-33. PubMed PMID: 15286966.

91: Kalai E, Bahlous A, Charni N, Bouzid K, Sahli H, Chelly M, Meddeb M, Zouari

B, Abdelmoula J, Sellami S. Increased urinary type II collagen C-telopeptide

levels in Tunisian patients with knee osteoarthritis. Clin Lab.

2012;58(3-4):209-15. PubMed PMID: 22582493.

92: Kalani A, Mohan A, Godbole MM, Bhatia E, Gupta A, Sharma RK, Tiwari S. Wilm's

tumor-1 protein levels in urinary exosomes from diabetic patients with or without

proteinuria. PLoS One. 2013;8(3):e60177. doi: 10.1371/journal.pone.0060177. Epub

2013 Mar 27. PubMed PMID: 23544132; PubMed Central PMCID: PMC3609819.

93: Kaminski HJ, Kusner LL, Wolfe GI, Aban I, Minisman G, Conwit R, Cutter G.

Biomarker development for myasthenia gravis. Ann N Y Acad Sci. 2012

Dec;1275:101-6. doi: 10.1111/j.1749-6632.2012.06787.x. Review. PubMed PMID:

23278584; PubMed Central PMCID: PMC3539232.

94: Kampoli AM, Tousoulis D, Papageorgiou N, Pallatza Z, Vogiatzi G, Briasoulis

A, Androulakis E, Toutouzas C, Stougianos P, Tentolouris C, Stefanadis C.

Clinical utility of biomarkers in premature atherosclerosis. Curr Med Chem.

2012;19(16):2521-33. Review. PubMed PMID: 22489712.

95: Kanno K, Sasaki S, Hirata Y, Ishikawa S, Fushimi K, Nakanishi S, Bichet DG,

Marumo F. Urinary excretion of aquaporin-2 in patients with diabetes insipidus. N

Engl J Med. 1995 Jun 8;332(23):1540-5. PubMed PMID: 7537863.

96: Keedy VL, Temin S, Somerfield MR, Beasley MB, Johnson DH, McShane LM, Milton

DT, Strawn JR, Wakelee HA, Giaccone G. American Society of Clinical Oncology

provisional clinical opinion: epidermal growth factor receptor (EGFR) Mutation

testing for patients with advanced non-small-cell lung cancer considering

first-line EGFR tyrosine kinase inhibitor therapy. J Clin Oncol. 2011 May

20;29(15):2121-7. doi: 10.1200/JCO.2010.31.8923. Epub 2011 Apr 11. PubMed PMID:

21482992.

97: Kemppainen E, Hedström J, Puolakkainen P, Halttunen J, Sainio V, Haapiainen

R, Stenman UH. Urinary trypsinogen-2 test strip in detecting ERCP-induced

pancreatitis. Endoscopy. 1997 May;29(4):247-51. PubMed PMID: 9255526.

98: Kengne-Momo RP, Lagarde F, Daniel P, Pilard JF, Durand MJ, Thouand G.

Polythiophene synthesis coupled to quartz crystal microbalance and Raman

spectroscopy for detecting bacteria. Biointerphases. 2012 Dec;7(1-4):67. doi:

10.1007/s13758-012-0067-1. Epub 2012 Nov 6. PubMed PMID: 23129343.

99: Kentsis A, Shulman A, Ahmed S, Brennan E, Monuteaux MC, Lee YH, Lipsett S,

Paulo JA, Dedeoglu F, Fuhlbrigge R, Bachur R, Bradwin G, Arditi M, Sundel RP,

Newburger JW, Steen H, Kim S. Urine proteomics for discovery of improved

diagnostic markers of Kawasaki disease. EMBO Mol Med. 2013 Feb;5(2):210-20. doi:

10.1002/emmm.201201494. Epub 2012 Dec 20. PubMed PMID: 23281308; PubMed Central

PMCID: PMC3569638.

100: Kentsis A, Ahmed S, Kurek K, Brennan E, Bradwin G, Steen H, Bachur R.

Detection and diagnostic value of urine leucine-rich α-2-glycoprotein in children

with suspected acute appendicitis. Ann Emerg Med. 2012 Jul;60(1):78-83.e1. doi:

10.1016/j.annemergmed.2011.12.015. Epub 2012 Feb 2. PubMed PMID: 22305331; PubMed

Central PMCID: PMC3726720.

101: Kentsis A, Lin YY, Kurek K, Calicchio M, Wang YY, Monigatti F, Campagne F,

Lee R, Horwitz B, Steen H, Bachur R. Discovery and validation of urine markers of

acute pediatric appendicitis using high-accuracy mass spectrometry. Ann Emerg

Med. 2010 Jan;55(1):62-70.e4. doi: 10.1016/j.annemergmed.2009.04.020. Epub 2009

Jun 25. PubMed PMID: 19556024; PubMed Central PMCID: PMC4422167.

102: Khan Z, Vlodov J, Horovitz J, Jose RM, Iswara K, Smotkin J, Brown A, Tenner

S. Urinary trypsinogen activation peptide is more accurate than hematocrit in

determining severity in patients with acute pancreatitis: a prospective study. Am

J Gastroenterol. 2002 Aug;97(8):1973-7. PubMed PMID: 12190163.

103: Kharbanda AB, Rai AJ, Cosme Y, Liu K, Dayan PS. Novel serum and urine

markers for pediatric appendicitis. Acad Emerg Med. 2012 Jan;19(1):56-62. doi:

10.1111/j.1553-2712.2011.01251.x. Epub 2012 Jan 5. PubMed PMID: 22221321; PubMed

Central PMCID: PMC3261304.

104: Khurana M, Traum AZ, Aivado M, Wells MP, Guerrero M, Grall F, Libermann TA,

Schachter AD. Urine proteomic profiling of pediatric nephrotic syndrome. Pediatr

Nephrol. 2006 Sep;21(9):1257-65. Epub 2006 Jun 30. PubMed PMID: 16810512; PubMed

Central PMCID: PMC1538636.

105: Klapper W, Hoster E, Rölver L, Schrader C, Janssen D, Tiemann M, Bernd HW,

Determann O, Hansmann ML, Möller P, Feller A, Stein H, Wacker HH, Dreyling M,

Unterhalt M, Hiddemann W, Ott G; German Low Grade Lymphoma Study Group. Tumor

sclerosis but not cell proliferation or malignancy grade is a prognostic marker

in advanced-stage follicular lymphoma: the German Low Grade Lymphoma Study Group.

J Clin Oncol. 2007 Aug 1;25(22):3330-6. PubMed PMID: 17664481.

106: Klimas NG, Broderick G, Fletcher MA. Biomarkers for chronic fatigue. Brain

Behav Immun. 2012 Nov;26(8):1202-10. doi: 10.1016/j.bbi.2012.06.006. Epub 2012

Jun 23. Review. PubMed PMID: 22732129.

107: Korthout M, De Bock R, van Bockstaele D, Peetermans M. Bone marrow cultures

and prognosis in primary myelodysplastic syndromes. Leuk Res. 1990;14(1):85-9.

PubMed PMID: 2304356.

108: Kraus VB, Kepler TB, Stabler T, Renner J, Jordan J. First qualification

study of serum biomarkers as indicators of total body burden of osteoarthritis.

PLoS One. 2010 Mar 17;5(3):e9739. doi: 10.1371/journal.pone.0009739. PubMed PMID:

20305824; PubMed Central PMCID: PMC2840035.

109: Kronborg CS, Allen J, Vittinghus E, Knudsen UB. Pre-symptomatic increase in

urine-orosomucoid excretion in pre-eclamptic women. Acta Obstet Gynecol Scand.

2007;86(8):930-7. PubMed PMID: 17653877.

110: Kumar V, Mishra M, Rajput SK, Bajpai S, Singh RK. Detection and diagnostic

applicability of human urinary kininogen in kala-azar patients. Parasitol Res.

2012 Oct;111(4):1851-5. Epub 2012 May 5. PubMed PMID: 22562212.

111: Kuromitsu S, Yokota H, Hiramoto M, Morita S, Mita H, Yamada T. Increased

concentration of neutrophil elastase in urine from patients with interstitial

cystitis. Scand J Urol Nephrol. 2008;42(5):455-61. doi:

10.1080/00365590802025881. PubMed PMID: 18609268.

112: Kwiatkowski DM, Goldstein SL, Krawczeski CD. Biomarkers of acute kidney

injury in pediatric cardiac patients. Biomark Med. 2012 Jun;6(3):273-82. doi:

10.2217/bmm.12.27. Review. PubMed PMID: 22731900.

113: Kylänpää-Bäck M, Kemppainen E, Puolakkainen P, Hedström J, Haapiainen R,

Perhoniemi V, Kivilaakso E, Korvuo A, Stenman U. Reliable screening for acute

pancreatitis with rapid urine trypsinogen-2 test strip. Br J Surg. 2000

Jan;87(1):49-52. PubMed PMID: 10606910.

114: La Thangue NB, Kerr DJ. Predictive biomarkers: a paradigm shift towards

personalized cancer medicine. Nat Rev Clin Oncol. 2011 Aug 23;8(10):587-96. doi:

10.1038/nrclinonc.2011.121. Review. PubMed PMID: 21862978.

115: Lamale LM, Lutgendorf SK, Zimmerman MB, Kreder KJ. Interleukin-6, histamine,

and methylhistamine as diagnostic markers for interstitial cystitis. Urology.

2006 Oct;68(4):702-6. PubMed PMID: 17070335.

116: Latsios G, Tousoulis D, Androulakis E, Papageorgiou N, Synetos A, Tsioufis

C, Toutouzas K, Stefanadis C. Monitoring calcific aortic valve disease: the role

of biomarkers. Curr Med Chem. 2012;19(16):2548-54. Review. PubMed PMID: 22489716.

117: Ledwidge M, Gallagher J, Conlon C, Tallon E, O'Connell E, Dawkins I, Watson

C, O'Hanlon R, Bermingham M, Patle A, Badabhagni MR, Murtagh G, Voon V, Tilson L,

Barry M, McDonald L, Maurer B, McDonald K. Natriuretic peptide-based screening

and collaborative care for heart failure: the STOP-HF randomized trial. JAMA.

2013 Jul 3;310(1):66-74. doi: 10.1001/jama.2013.7588. PubMed PMID: 23821090.

118: Lempinen M, Stenman UH, Finne P, Puolakkainen P, Haapiainen R, Kemppainen E.

Trypsinogen-2 and trypsinogen activation peptide (TAP) in urine of patients with

acute pancreatitis. J Surg Res. 2003 May 15;111(2):267-73. PubMed PMID: 12850473.

119: Lempinen M, Kylänpää-Bäck ML, Stenman UH, Puolakkainen P, Haapiainen R,

Finne P, Korvuo A, Kemppainen E. Predicting the severity of acute pancreatitis by

rapid measurement of trypsinogen-2 in urine. Clin Chem. 2001 Dec;47(12):2103-7.

PubMed PMID: 11719473.

120: Levesque JP, Winkler IG, Rasko JE. Nichotherapy for stem cells: there goes

the neighborhood. Bioessays. 2013 Mar;35(3):183-90. doi: 10.1002/bies.201200111.

Epub 2012 Nov 5. Review. PubMed PMID: 23129341.

121: Li CR, Yang XQ, Shen J, Li YB, Jiang LP. Immunoglobulin G subclasses in

serum and circulating immune complexes in patients with Kawasaki syndrome.

Pediatr Infect Dis J. 1990 Aug;9(8):544-7. PubMed PMID: 2235169.

122: Ling W, Zhaohui N, Ben H, Leyi G, Jianping L, Huili D, Jiaqi Q. Urinary

IL-18 and NGAL as early predictive biomarkers in contrast-induced nephropathy

after coronary angiography. Nephron Clin Pract. 2008;108(3):c176-81. doi:

10.1159/000117814. Epub 2008 Feb 21. PubMed PMID: 18287807.

123: Ling XB, Lau K, Deshpande C, Park JL, Milojevic D, Macaubas C, Xiao C,

Lopez-Avila V, Kanegaye J, Burns JC, Cohen H, Schilling J, Mellins ED. Urine

Peptidomic and Targeted Plasma Protein Analyses in the Diagnosis and Monitoring

of Systemic Juvenile Idiopathic Arthritis. Clin Proteomics. 2010

Dec;6(4):175-193. Epub 2010 Sep 30. PubMed PMID: 21124648; PubMed Central PMCID:

PMC2970804.

124: Liu BC, Zhang L, Lv LL, Wang YL, Liu DG, Zhang XL. Application of antibody

array technology in the analysis of urinary cytokine profiles in patients with

chronic kidney disease. Am J Nephrol. 2006;26(5):483-90. Epub 2006 Nov 7. PubMed

PMID: 17095863.

125: Liu CC, Kanekiyo T, Xu H, Bu G. Apolipoprotein E and Alzheimer disease:

risk, mechanisms and therapy. Nat Rev Neurol. 2013 Feb;9(2):106-18. doi:

10.1038/nrneurol.2012.263. Epub 2013 Jan 8. Erratum in: Nat Rev Neurol. 2013.

doi: 10.1038/nmeurol.2013.32. Liu, Chia-Chan [corrected to Liu, Chia-Chen].

PubMed PMID: 23296339; PubMed Central PMCID: PMC3726719.

126: Liu HT, Tyagi P, Chancellor MB, Kuo HC. Urinary nerve growth factor but not

prostaglandin E2 increases in patients with interstitial cystitis/bladder pain

syndrome and detrusor overactivity. BJU Int. 2010 Dec;106(11):1681-5. doi:

10.1111/j.1464-410X.2009.08851.x. PubMed PMID: 19751258.

127: Liu L, Zheng CX, Peng SF, Zhou HY, Su ZY, He L, Ai T. Evaluation of urinary

S100B protein level and lactate/creatinine ratio for early diagnosis and

prognostic prediction of neonatal hypoxic-ischemic encephalopathy. Neonatology.

2010;97(1):41-4. doi: 10.1159/000227292. Epub 2009 Jul 7. PubMed PMID: 19590245.

128: Liu ZS, Jiang CQ, Qian Q, Sun Q, Fan LF, Ai ZL. Early prediction of severe

acute pancreatitis by urinary trypsinogen activation peptide. Hepatobiliary

Pancreat Dis Int. 2002 May;1(2):285-9. PubMed PMID: 14612286.

129: Lochmanová G, Jedličková L, Potěšil D, Tomancová A, Verner J, Pospíšilová S,

Doubek M, Mayer J, Zdráhal Z. Potential biomarkers for early detection of acute

graft-versus-host disease. Proteomics Clin Appl. 2012 Aug;6(7-8):351-63. doi:

10.1002/prca.201100104. Review. PubMed PMID: 22927351.

130: Locker GY, Hamilton S, Harris J, Jessup JM, Kemeny N, Macdonald JS,

Somerfield MR, Hayes DF, Bast RC Jr; ASCO. ASCO 2006 update of recommendations

for the use of tumor markers in gastrointestinal cancer. J Clin Oncol. 2006 Nov

20;24(33):5313-27. Epub 2006 Oct 23. PubMed PMID: 17060676.

131: Lota HK, Renzoni EA. Circulating biomarkers of interstitial lung disease in

systemic sclerosis. Int J Rheumatol. 2012;2012:121439. doi: 10.1155/2012/121439.

Epub 2012 Sep 3. PubMed PMID: 22988462; PubMed Central PMCID: PMC3439977.

132: Maahs DM, Siwy J, Argilés A, Cerna M, Delles C, Dominiczak AF, Gayrard N,

Iphöfer A, Jänsch L, Jerums G, Medek K, Mischak H, Navis GJ, Roob JM, Rossing K,

Rossing P, Rychlík I, Schiffer E, Schmieder RE, Wascher TC, Winklhofer-Roob BM,

Zimmerli LU, Zürbig P, Snell-Bergeon JK. Urinary collagen fragments are

significantly altered in diabetes: a link to pathophysiology. PLoS One. 2010 Sep

28;5(9). pii: e13051. doi: 10.1371/journal.pone.0013051. PubMed PMID: 20927192;

PubMed Central PMCID: PMC2946909.

133: Magistroni R, Ligabue G, Lupo V, Furci L, Leonelli M, Manganelli L, Masellis

M, Gatti V, Cavazzini F, Tizzanini W, Albertazzi A. Proteomic analysis of urine

from proteinuric patients shows a proteolitic activity directed against albumin.

Nephrol Dial Transplant. 2009 May;24(5):1672-81. doi: 10.1093/ndt/gfp020. Epub

2009 Feb 11. PubMed PMID: 19211645.

134: Makino T, Kawashima H, Konishi H, Nakatani T, Kiyama H. Elevated urinary

levels and urothelial expression of

hepatocarcinoma-intestine-pancreas/pancreatitis-associated protein in patients

with interstitial cystitis. Urology. 2010 Apr;75(4):933-7. doi:

10.1016/j.urology.2009.05.044. Epub 2009 Jul 30. PubMed PMID: 19646740.

135: Mannoia K, Boskovic DS, Slater L, Plank MS, Angeles DM, Gollin G.

Necrotizing enterocolitis is associated with neonatal intestinal injury. J

Pediatr Surg. 2011 Jan;46(1):81-5. doi: 10.1016/j.jpedsurg.2010.09.069. PubMed

PMID: 21238645.

136: Mao YN, Liu W, Li YG, Jia GC, Zhang Z, Guan YJ, Zhou XF, Liu YF. Urinary

angiotensinogen levels in relation to renal involvement of Henoch-Schonlein

purpura in children. Nephrology (Carlton). 2012 Jan;17(1):53-7. doi:

10.1111/j.1440-1797.2011.01515.x. PubMed PMID: 21854508.

137: Markó L, Szigeti N, Szabó Z, Böddi K, Takátsy A, Ludány A, Koszegi T, Molnár

GA, Wittmann I. Potential urinary biomarkers of disease activity in Crohn's

disease. Scand J Gastroenterol. 2010 Dec;45(12):1440-8. doi:

10.3109/00365521.2010.505658. Epub 2010 Jul 26. PubMed PMID: 20653491.

138: Martins C, Buczynski AK, Maia LC, Siqueira WL, Castro GF. Salivary proteins

as a biomarker for dental caries--a systematic review. J Dent. 2013

Jan;41(1):2-8. doi: 10.1016/j.jdent.2012.10.015. Epub 2012 Nov 9. Review. PubMed

PMID: 23142096.

139: Martínez-Murcia A, Beaz-Hidalgo R, Svec P, Saavedra MJ, Figueras MJ,

Sedlacek I. Aeromonas cavernicola sp. nov., isolated from fresh water of a brook

in a cavern. Curr Microbiol. 2013 Feb;66(2):197-204. doi:

10.1007/s00284-012-0253-x. Epub 2012 Nov 6. PubMed PMID: 23129345.

140: Masoura S, Kalogiannidis IA, Gitas G, Goutsioulis A, Koiou E, Athanasiadis

A, Vavatsi N. Biomarkers in pre-eclampsia: a novel approach to early detection of

the disease. J Obstet Gynaecol. 2012 Oct;32(7):609-16. doi:

10.3109/01443615.2012.709290. Review. PubMed PMID: 22943702.

141: McGhee DJ, Royle PL, Thompson PA, Wright DE, Zajicek JP, Counsell CE. A

systematic review of biomarkers for disease progression in Parkinson's disease.

BMC Neurol. 2013 Apr 12;13:35. doi: 10.1186/1471-2377-13-35. Review. PubMed PMID:

23587062; PubMed Central PMCID: PMC3637496.

142: Meijer E, Boertien WE, Nauta FL, Bakker SJ, van Oeveren W, Rook M, van der

Jagt EJ, van Goor H, Peters DJ, Navis G, de Jong PE, Gansevoort RT. Association

of urinary biomarkers with disease severity in patients with autosomal dominant

polycystic kidney disease: a cross-sectional analysis. Am J Kidney Dis. 2010

Nov;56(5):883-95. doi: 10.1053/j.ajkd.2010.06.023. PubMed PMID: 20888104.

143: Mesrobian HG, Mitchell ME, See WA, Halligan BD, Carlson BE, Greene AS, Wakim

BT. Candidate urinary biomarker discovery in ureteropelvic junction obstruction:

a proteomic approach. J Urol. 2010 Aug;184(2):709-14. doi:

10.1016/j.juro.2010.03.061. PubMed PMID: 20639044.

144: Metzger J, Negm AA, Plentz RR, Weismüller TJ, Wedemeyer J, Karlsen TH, Dakna

M, Mullen W, Mischak H, Manns MP, Lankisch TO. Urine proteomic analysis

differentiates cholangiocarcinoma from primary sclerosing cholangitis and other

benign biliary disorders. Gut. 2013 Jan;62(1):122-30. doi:

10.1136/gutjnl-2012-302047. Epub 2012 May 12. PubMed PMID: 22580416.

145: Millar EK, Graham PH, O'Toole SA, McNeil CM, Browne L, Morey AL, Eggleton S,

Beretov J, Theocharous C, Capp A, Nasser E, Kearsley JH, Delaney G, Papadatos G,

Fox C, Sutherland RL. Prediction of local recurrence, distant metastases, and

death after breast-conserving therapy in early-stage invasive breast cancer using

a five-biomarker panel. J Clin Oncol. 2009 Oct 1;27(28):4701-8. doi:

10.1200/JCO.2008.21.7075. Epub 2009 Aug 31. PubMed PMID: 19720911.

146: Minamida S, Iwamura M, Kodera Y, Kawashima Y, Tabata K, Matsumoto K, Fujita

T, Satoh T, Maeda T, Baba S. 14-3-3 protein beta/alpha as a urinary biomarker for

renal cell carcinoma: proteomic analysis of cyst fluid. Anal Bioanal Chem. 2011

Jul;401(1):245-52. doi: 10.1007/s00216-011-5057-5. Epub 2011 May 8. PubMed PMID:

21553213.

147: Mirfeizi Z, Mahmoudi M, Naghibi M, Hatef M, Sharifipour F, Jokar M, Zeraati

A, Shariati Sarabi Z, Azarian A. Urine Monocyte Chemoattractant Protein-1(UMCP-1)

as a Biomarker of Renal Involvement in Systemic Lupus Erythematosus. Iran J Basic

Med Sci. 2012 Nov;15(6):1191-5. PubMed PMID: 23653850; PubMed Central PMCID:

PMC3646231.

148: Misu T, Takahashi T, Nakashima I, Fujihara K. [Biomarkers in neuromyelitis

optica]. Brain Nerve. 2012 May;64(5):525-35. Review. Japanese. PubMed PMID:

22570066.

149: Mobasheri A. Osteoarthritis year 2012 in review: biomarkers. Osteoarthritis

Cartilage. 2012 Dec;20(12):1451-64. doi: 10.1016/j.joca.2012.07.009. Epub 2012

Jul 25. Review. PubMed PMID: 22842200.

150: Moller MJ, Qin Z, Toursarkissian B. Tissue markers in human atherosclerotic

carotid artery plaque. Ann Vasc Surg. 2012 Nov;26(8):1160-5. doi:

10.1016/j.avsg.2012.06.008. Review. PubMed PMID: 23068427.

151: Moon PG, Lee JE, You S, Kim TK, Cho JH, Kim IS, Kwon TH, Kim CD, Park SH,

Hwang D, Kim YL, Baek MC. Proteomic analysis of urinary exosomes from patients of

early IgA nephropathy and thin basement membrane nephropathy. Proteomics. 2011

Jun;11(12):2459-75. doi: 10.1002/pmic.201000443. Epub 2011 May 18. PubMed PMID:

21595033.

152: Morris TA, Marsh JJ, Burrows CM, Chiles PG, Konopka RG, Pedersen CA. Urine

and plasma levels of fibrinopeptide B in patients with deep vein thrombosis and

pulmonary embolism. Thromb Res. 2003 May 1;110(2-3):159-65. PubMed PMID:

12893031.

153: Morrissey JJ, London AN, Luo J, Kharasch ED. Urinary biomarkers for the

early diagnosis of kidney cancer. Mayo Clin Proc. 2010 May;85(5):413-21. doi:

10.4065/mcp.2009.0709. Epub 2010 Apr 7. PubMed PMID: 20375178; PubMed Central

PMCID: PMC2861969.

154: Mou S, Wang Q, Li J, Shi B, Ni Z. Urinary excretion of liver-type fatty

acid-binding protein as a marker of progressive kidney function deterioration in

patients with chronic glomerulonephritis. Clin Chim Acta. 2012 Jan

18;413(1-2):187-91. doi: 10.1016/j.cca.2011.09.018. Epub 2011 Sep 17. PubMed

PMID: 21958699.

155: Na HJ, Hamilton RG, Klion AD, Bochner BS. Biomarkers of eosinophil

involvement in allergic and eosinophilic diseases: review of phenotypic and serum

markers including a novel assay to quantify levels of soluble Siglec-8. J Immunol

Methods. 2012 Sep 28;383(1-2):39-46. doi: 10.1016/j.jim.2012.05.017. Epub 2012

Jun 6. PubMed PMID: 22683541; PubMed Central PMCID: PMC3411856.

156: Nakamura T, Sugaya T, Node K, Ueda Y, Koide H. Urinary excretion of

liver-type fatty acid-binding protein in contrast medium-induced nephropathy. Am

J Kidney Dis. 2006 Mar;47(3):439-44. PubMed PMID: 16490622.

157: Neoptolemos JP, Kemppainen EA, Mayer JM, Fitzpatrick JM, Raraty MG, Slavin

J, Beger HG, Hietaranta AJ, Puolakkainen PA. Early prediction of severity in

acute pancreatitis by urinary trypsinogen activation peptide: a multicentre

study. Lancet. 2000 Jun 3;355(9219):1955-60. PubMed PMID: 10859041.

158: Nguyen TQ, Tarnow L, Andersen S, Hovind P, Parving HH, Goldschmeding R, van

Nieuwenhoven FA. Urinary connective tissue growth factor excretion correlates

with clinical markers of renal disease in a large population of type 1 diabetic

patients with diabetic nephropathy. Diabetes Care. 2006 Jan;29(1):83-8. PubMed

PMID: 16373901.

159: Nicholas BL. Search for biomarkers in chronic obstructive pulmonary disease:

current status. Curr Opin Pulm Med. 2013 Mar;19(2):103-8. doi:

10.1097/MCP.0b013e32835ceee5. Review. PubMed PMID: 23361193.

160: Noel-Storr AH, Flicker L, Ritchie CW, Nguyen GH, Gupta T, Wood P, Walton J,

Desai M, Solomon DF, Molena E, Worrall R, Hayen A, Choudhary P, Ladds E, Lanctôt

KL, Verhey FR, McCleery JM, Mead GE, Clare L, Fioravanti M, Hyde C, Marcus S,

McShane R. Systematic review of the body of evidence for the use of biomarkers in

the diagnosis of dementia. Alzheimers Dement. 2013 May;9(3):e96-e105. doi:

10.1016/j.jalz.2012.01.014. Epub 2012 Oct 27. Review. PubMed PMID: 23110863.

161: Northrop-Clewes CA, Thurnham DI. Biomarkers for the differentiation of

anemia and their clinical usefulness. J Blood Med. 2013 Mar 20;4:11-22. doi:

10.2147/JBM.S29212. Print 2013. PubMed PMID: 23687454; PubMed Central PMCID:

PMC3610441.

162: O'Seaghdha CM, Hwang SJ, Bhavsar NA, Köttgen A, Coresh J, Astor BC, Fox CS.

Lower urinary connective tissue growth factor levels and incident CKD stage 3 in

the general population. Am J Kidney Dis. 2011 Jun;57(6):841-9. doi:

10.1053/j.ajkd.2010.11.022. Epub 2011 Feb 3. PubMed PMID: 21292366; PubMed

Central PMCID: PMC3100386.

163: Obara T, Mizoguchi S, Shimozuru Y, Sato T, Hotta O. The complex of

immunoglobulin A and uromodulin as a diagnostic marker for immunoglobulin A

nephropathy. Clin Exp Nephrol. 2012 Oct;16(5):713-21. doi:

10.1007/s10157-012-0617-3. Epub 2012 Mar 14. PubMed PMID: 22415778; PubMed

Central PMCID: PMC3465549.

164: Oxley D, Wilkinson SG. A common structure for neutral polymers isolated from

the lipopolysaccharides of reference strains for Serratia marcescens serogroups

O17 and O19. Carbohydr Res. 1990 Apr 2;198(1):168-72. PubMed PMID: 2191776.

165: Pacchiarotta T, Hensbergen PJ, Wuhrer M, van Nieuwkoop C, Nevedomskaya E,

Derks RJ, Schoenmaker B, Koeleman CA, van Dissel J, Deelder AM, Mayboroda OA.

Fibrinogen alpha chain O-glycopeptides as possible markers of urinary tract

infection. J Proteomics. 2012 Jan 4;75(3):1067-73. doi:

10.1016/j.jprot.2011.10.021. Epub 2011 Oct 31. PubMed PMID: 22075168.

166: Paczesny S. Discovery and validation of graft-versus-host disease

biomarkers. Blood. 2013 Jan 24;121(4):585-94. doi: 10.1182/blood-2012-08-355990.

Epub 2012 Nov 19. Review. PubMed PMID: 23165480; PubMed Central PMCID:

PMC3557644.

167: Park MR, Wang EH, Jin DC, Cha JH, Lee KH, Yang CW, Kang CS, Choi YJ.

Establishment of a 2-D human urinary proteomic map in IgA nephropathy.

Proteomics. 2006 Feb;6(3):1066-76. PubMed PMID: 16372274.

168: Pedrotty DM, Morley MP, Cappola TP. Transcriptomic biomarkers of

cardiovascular disease. Prog Cardiovasc Dis. 2012 Jul-Aug;55(1):64-9. doi:

10.1016/j.pcad.2012.06.003. Review. PubMed PMID: 22824111; PubMed Central PMCID:

PMC4131429.

169: Peleg-Raibstein D, Feldon J, Meyer U. Behavioral animal models of

antipsychotic drug actions. Handb Exp Pharmacol. 2012;(212):361-406. doi:

10.1007/978-3-642-25761-2_14. Review. PubMed PMID: 23129339.

170: Peralta CA, Katz R, Bonventre JV, Sabbisetti V, Siscovick D, Sarnak M,

Shlipak MG. Associations of urinary levels of kidney injury molecule 1 (KIM-1)

and neutrophil gelatinase-associated lipocalin (NGAL) with kidney function

decline in the Multi-Ethnic Study of Atherosclerosis (MESA). Am J Kidney Dis.

2012 Dec;60(6):904-11. doi: 10.1053/j.ajkd.2012.05.014. Epub 2012 Jun 30. PubMed

PMID: 22749388; PubMed Central PMCID: PMC3690926.

171: Petersson U, Appelros S, Borgström A. Different patterns in immunoreactive

anionic and cationic trypsinogen in urine and serum in human acute pancreatitis.

Int J Pancreatol. 1999 Jun;25(3):165-70. PubMed PMID: 10453418.

172: Pfisterer J, Du Bois A, Bentz EK, Kommoss F, Harter P, Huober J, Schmalfeldt

B, Burchardi N, Arnold N, Hilpert F. Prognostic value of human epidermal growth

factor receptor 2 (Her-2)/neu in patients with advanced ovarian cancer treated

with platinum/paclitaxel as first-line chemotherapy: a retrospective evaluation

of the AGO-OVAR 3 Trial by the AGO OVAR Germany. Int J Gynecol Cancer. 2009

Jan;19(1):109-15. doi: 10.1111/IGC.0b013e3181991a7c. PubMed PMID: 19258951.

173: Pfisterer M, Buser P, Rickli H, Gutmann M, Erne P, Rickenbacher P,

Vuillomenet A, Jeker U, Dubach P, Beer H, Yoon SI, Suter T, Osterhues HH,

Schieber MM, Hilti P, Schindler R, Brunner-La Rocca HP; TIME-CHF Investigators.

BNP-guided vs symptom-guided heart failure therapy: the Trial of Intensified vs

Standard Medical Therapy in Elderly Patients With Congestive Heart Failure

(TIME-CHF) randomized trial. JAMA. 2009 Jan 28;301(4):383-92. doi:

10.1001/jama.2009.2. PubMed PMID: 19176440.

174: Pich EM, Vargas G, Domenici E. Biomarkers for antipsychotic therapies. Handb

Exp Pharmacol. 2012;(212):339-60. doi: 10.1007/978-3-642-25761-2_13. Review.

PubMed PMID: 23129338.

175: Piyaphanee N, Ma Q, Kremen O, Czech K, Greis K, Mitsnefes M, Devarajan P,

Bennett MR. Discovery and initial validation of α 1-B glycoprotein fragmentation

as a differential urinary biomarker in pediatric steroid-resistant nephrotic

syndrome. Proteomics Clin Appl. 2011 Jun;5(5-6):334-42. doi:

10.1002/prca.201000110. Epub 2011 May 18. PubMed PMID: 21591266.

176: Qian YB, Zhang JB, Wu WZ, Fang HB, Jia WD, Zhuang PY, Zhang BH, Pan Q, Xu Y,

Wang L, Tang ZY, Sun HC. P48 is a predictive marker for outcome of postoperative

interferon-alpha treatment in patients with hepatitis B virus infection-related

hepatocellular carcinoma. Cancer. 2006 Oct 1;107(7):1562-9. PubMed PMID:

16948122.

177: Raimondo F, Morosi L, Corbetta S, Chinello C, Brambilla P, Della Mina P,

Villa A, Albo G, Battaglia C, Bosari S, Magni F, Pitto M. Differential protein

profiling of renal cell carcinoma urinary exosomes. Mol Biosyst. 2013

Jun;9(6):1220-33. doi: 10.1039/c3mb25582d. Epub 2013 Mar 19. PubMed PMID:

23511837.

178: Raman K, Chong M, Akhtar-Danesh GG, D'Mello M, Hasso R, Ross S, Xu F, Paré

G. Genetic markers of inflammation and their role in cardiovascular disease. Can

J Cardiol. 2013 Jan;29(1):67-74. doi: 10.1016/j.cjca.2012.06.025. Epub 2012 Sep

19. Review. PubMed PMID: 22999193.

179: Ravandi F, Alattar ML, Grunwald MR, Rudek MA, Rajkhowa T, Richie MA, Pierce

S, Daver N, Garcia-Manero G, Faderl S, Nazha A, Konopleva M, Borthakur G, Burger

J, Kadia T, Dellasala S, Andreeff M, Cortes J, Kantarjian H, Levis M. Phase 2

study of azacytidine plus sorafenib in patients with acute myeloid leukemia and

FLT-3 internal tandem duplication mutation. Blood. 2013 Jun 6;121(23):4655-62.

doi: 10.1182/blood-2013-01-480228. Epub 2013 Apr 23. PubMed PMID: 23613521;

PubMed Central PMCID: PMC3674666.

180: Riaz S, Alam SS, Srai SK, Skinner V, Riaz A, Akhtar MW. Proteomic

identification of human urinary biomarkers in diabetes mellitus type 2. Diabetes

Technol Ther. 2010 Dec;12(12):979-88. doi: 10.1089/dia.2010.0078. Epub 2010 Aug

24. PubMed PMID: 20735160.

181: Roberts JD, Wells GA, Le May MR, Labinaz M, Glover C, Froeschl M, Dick A,

Marquis JF, O'Brien E, Goncalves S, Druce I, Stewart A, Gollob MH, So DY.

Point-of-care genetic testing for personalisation of antiplatelet treatment

(RAPID GENE): a prospective, randomised, proof-of-concept trial. Lancet. 2012 May

5;379(9827):1705-11. doi: 10.1016/S0140-6736(12)60161-5. Epub 2012 Mar 29. PubMed

PMID: 22464343.

182: Robinson D, Sandblom G, Johansson R, Garmo H, Stattin P, Mommsen S,

Varenhorst E; Scandinavian Prostate Cancer Group (SPCG)-5. Prediction of survival

of metastatic prostate cancer based on early serial measurements of prostate

specific antigen and alkaline phosphatase. J Urol. 2008 Jan;179(1):117-22;

discussion 122-3. Epub 2007 Nov 12. PubMed PMID: 17997442.

183: Rocchetti MT, Centra M, Papale M, Bortone G, Palermo C, Centonze D, Ranieri

E, Di Paolo S, Gesualdo L. Urine protein profile of IgA nephropathy patients may

predict the response to ACE-inhibitor therapy. Proteomics. 2008 Jan;8(1):206-16.

PubMed PMID: 18095357.

184: Rodríguez-Rodríguez A, Egea-Guerrero JJ, León-Justel A, Gordillo-Escobar E,

Revuelto-Rey J, Vilches-Arenas A, Carrillo-Vico A, Domínguez-Roldán JM,

Murillo-Cabezas F, Guerrero JM. Role of S100B protein in urine and serum as an

early predictor of mortality after severe traumatic brain injury in adults. Clin

Chim Acta. 2012 Dec 24;414:228-33. doi: 10.1016/j.cca.2012.09.025. Epub 2012 Sep

29. PubMed PMID: 23031665.

185: Rodríguez-Rodríguez L, López-Mejías R, García-Bermúdez M, González-Juanatey

C, González-Gay MA, Martín J. Genetic markers of cardiovascular disease in

rheumatoid arthritis. Mediators Inflamm. 2012;2012:574817. doi:

10.1155/2012/574817. Epub 2012 Aug 2. Review. PubMed PMID: 22927710; PubMed

Central PMCID: PMC3419432.

186: Rodón J, Saura C, Dienstmann R, Vivancos A, Ramón y Cajal S, Baselga J,

Tabernero J. Molecular prescreening to select patient population in early

clinical trials. Nat Rev Clin Oncol. 2012 Apr 3;9(6):359-66. doi:

10.1038/nrclinonc.2012.48. PubMed PMID: 22473105.

187: Rosa RF, Takei K, Araújo NC, Loduca SM, Szajubok JC, Chahade WH. Monocyte

chemoattractant-1 as a urinary biomarker for the diagnosis of activity of lupus

nephritis in Brazilian patients. J Rheumatol. 2012 Oct;39(10):1948-54. doi:

10.3899/jrheum.110201. Epub 2012 Sep 1. PubMed PMID: 22942263.

188: Rousseau JC, Delmas PD. Biological markers in osteoarthritis. Nat Clin Pract

Rheumatol. 2007 Jun;3(6):346-56. Review. PubMed PMID: 17538566.

189: Sabbagh JJ, Kinney JW, Cummings JL. Alzheimer's disease biomarkers:

correspondence between human studies and animal models. Neurobiol Dis. 2013

Aug;56:116-30. doi: 10.1016/j.nbd.2013.04.010. Epub 2013 Apr 28. Review. PubMed

PMID: 23631871.

190: Sackett MK, Bairati I, Meyer F, Jobin E, Lussier S, Fortin A, Gélinas M,

Nabid A, Brochet F, Têtu B. Prognostic significance of cyclooxygenase-2

overexpression in glottic cancer. Clin Cancer Res. 2008 Jan 1;14(1):67-73. doi:

10.1158/1078-0432.CCR-07-2028. PubMed PMID: 18172254.

191: Sambursky R, Davitt WF 3rd, Latkany R, Tauber S, Starr C, Friedberg M, Dirks

MS, McDonald M. Sensitivity and specificity of a point-of-care matrix

metalloproteinase 9 immunoassay for diagnosing inflammation related to dry eye.

JAMA Ophthalmol. 2013 Jan;131(1):24-8. doi: 10.1001/jamaophthalmol.2013.561.

Erratum in: JAMA Ophthalmol. 2013 Mar 1;131(3):364. PubMed PMID: 23307206.

192: Sand-Dejmek J, Ehrnström R, Berglund P, Andersson T, Ryden L. The prognostic

significance of Wnt-5a expression in primary breast cancer is extended to

premenopausal women. PLoS One. 2013 Aug 22;8(8):e70890. doi:

10.1371/journal.pone.0070890. eCollection 2013. PubMed PMID: 23990917; PubMed

Central PMCID: PMC3750047.

193: Santos AC Jr, Lima EM, Penido MG, Silveira KD, Teixeira MM, Oliveira EA,

Simões E Silva AC. Plasma and urinary levels of cytokines in patients with

idiopathic hypercalciuria. Pediatr Nephrol. 2012 Jun;27(6):941-8. doi:

10.1007/s00467-011-2094-4. Epub 2012 Jan 7. PubMed PMID: 22223141.

194: Satoh-Asahara N, Suganami T, Majima T, Kotani K, Kato Y, Araki R, Koyama K,

Okajima T, Tanabe M, Oishi M, Himeno A, Kono S, Sugawara A, Hattori M, Ogawa Y,

Shimatsu A; Japan Obesity and Metabolic Syndrome Study (JOMS) Group. Urinary

cystatin C as a potential risk marker for cardiovascular disease and chronic

kidney disease in patients with obesity and metabolic syndrome. Clin J Am Soc

Nephrol. 2011 Feb;6(2):265-73. doi: 10.2215/CJN.04830610. Epub 2010 Nov 4. PubMed

PMID: 21051748; PubMed Central PMCID: PMC3052215.

195: Savel'ev SV, Ivanov AI, Gulimova VI, Korochkin LI. [Changes in amphibian

behavior after transplantation into the brain of neural anlage cells from

Drosophila]. Dokl Akad Nauk SSSR. 1991;316(3):735-8. Russian. PubMed PMID:

1905993.

196: Schlatzer D, Maahs DM, Chance MR, Dazard JE, Li X, Hazlett F, Rewers M,

Snell-Bergeon JK. Novel urinary protein biomarkers predicting the development of

microalbuminuria and renal function decline in type 1 diabetes. Diabetes Care.

2012 Mar;35(3):549-55. doi: 10.2337/dc11-1491. Epub 2012 Jan 11. PubMed PMID:

22238279; PubMed Central PMCID: PMC3322681.

197: Schorr M, Manns BJ, Culleton B, Walsh M, Klarenbach S, Tonelli M, Sauve L,

Chin R, Barnieh L, Hemmelgarn BR; Alberta Kidney Disease Network. The effect of

nocturnal and conventional hemodialysis on markers of nutritional status: results

from a randomized trial. J Ren Nutr. 2011 May;21(3):271-6. doi:

10.1053/j.jrn.2010.04.004. Epub 2010 Jul 21. PubMed PMID: 20650654.

198: Scott-Thomas AJ, Syhre M, Pattemore PK, Epton M, Laing R, Pearson J,

Chambers ST. 2-Aminoacetophenone as a potential breath biomarker for Pseudomonas

aeruginosa in the cystic fibrosis lung. BMC Pulm Med. 2010 Nov 7;10:56. doi:

10.1186/1471-2466-10-56. PubMed PMID: 21054900; PubMed Central PMCID: PMC2989937.

199: Segarra-Medrano A, Carnicer-Cáceres C, Arbós-Via MA, Quiles-Pérez MT,

Agraz-Pamplona I, Ostos-Roldán E. Biological markers of nephrotic syndrome: a few

steps forward in the long way. Nefrologia. 2012;32(5):558-72. doi:

10.3265/Nefrologia.pre2012.Jun.11396. Review. English, Spanish. PubMed PMID:

23013941.

200: Sharma K, Ziyadeh FN, Alzahabi B, McGowan TA, Kapoor S, Kurnik BR, Kurnik

PB, Weisberg LS. Increased renal production of transforming growth factor-beta1

in patients with type II diabetes. Diabetes. 1997 May;46(5):854-9. PubMed PMID:

9133555.

201: Siasos G, Tousoulis D, Michalea S, Oikonomou E, Kolia C, Kioufis S, Synetos

A, Vlasis K, Papavassiliou AG, Stefanadis C. Biomarkers determining

cardiovascular risk in patients with kidney disease. Curr Med Chem.

2012;19(16):2555-71. Review. PubMed PMID: 22489717.

202: Sibai BM, Koch MA, Freire S, Pinto e Silva JL, Rudge MV, Martins-Costa S,

Bartz J, de Barros Santos C, Cecatti JG, Costa R, Ramos JG, Spinnato JA 2nd.

Serum inhibin A and angiogenic factor levels in pregnancies with previous

preeclampsia and/or chronic hypertension: are they useful markers for prediction

of subsequent preeclampsia? Am J Obstet Gynecol. 2008 Sep;199(3):268.e1-9. doi:

10.1016/j.ajog.2008.06.071. PubMed PMID: 18771979.

203: Simma B, Höliner I, Luetschg J. Therapy in pediatric stroke. Eur J Pediatr.

2013 Jul;172(7):867-75. doi: 10.1007/s00431-012-1863-9. Epub 2012 Nov 6. Review.

PubMed PMID: 23129344.

204: Singh RG, Usha, Rathore SS, Behura SK, Singh NK. Urinary MCP-1 as diagnostic

and prognostic marker in patients with lupus nephritis flare. Lupus. 2012

Oct;21(11):1214-8. doi: 10.1177/0961203312452622. Epub 2012 Jun 27. PubMed PMID:

22759858.

205: Smith ER, Lee D, Cai MM, Tomlinson LA, Ford ML, McMahon LP, Holt SG. Urinary

neutrophil gelatinase-associated lipocalin may aid prediction of renal decline in

patients with non-proteinuric Stages 3 and 4 chronic kidney disease (CKD).

Nephrol Dial Transplant. 2013 Jun;28(6):1569-79. doi: 10.1093/ndt/gfs586. Epub

2013 Jan 16. PubMed PMID: 23328709.

206: Somparn P, Hirankarn N, Leelahavanichkul A, Khovidhunkit W, Thongboonkerd V,

Avihingsanon Y. Urinary proteomics revealed prostaglandin H(2)D-isomerase, not

Zn-α2-glycoprotein, as a biomarker for active lupus nephritis. J Proteomics. 2012

Jun 18;75(11):3240-7. doi: 10.1016/j.jprot.2012.03.034. Epub 2012 Mar 30. PubMed

PMID: 22498882.

207: Spahn JD. Asthma biomarkers in sputum. Immunol Allergy Clin North Am. 2012

Aug;32(3):387-99. Review. PubMed PMID: 22877617.

208: Stefanović V, Djukanović L, Cukuranović R, Bukvić D, Ležaić V, Marić I,

Ogrizovic SS, Jovanović I, Vlahovic P, Pešić I, Djordjević V. Beta2-microglobulin

and alpha1-microglobulin as markers of Balkan endemic nephropathy, a worldwide

disease. Ren Fail. 2011;33(2):176-83. doi: 10.3109/0886022X.2011.552152. PubMed

PMID: 21332340.

209: Suen JL, Liu CC, Lin YS, Tsai YF, Juo SH, Chou YH. Urinary

chemokines/cytokines are elevated in patients with urolithiasis. Urol Res. 2010

Apr;38(2):81-7. doi: 10.1007/s00240-010-0260-y. Epub 2010 Mar 4. PubMed PMID:

20204339.

210: Suguna Narasimhulu S, Hendricks-Muñoz KD, Borkowsky W, Mally P. Usefulness

of urinary immune biomarkers in the evaluation of neonatal sepsis: a pilot

project. Clin Pediatr (Phila). 2013 Jun;52(6):520-6. doi:

10.1177/0009922813482751. Epub 2013 Mar 28. PubMed PMID: 23539685.

211: Surin B, Sachon E, Rougier JP, Steverlynck C, Garreau C, Lelongt B, Ronco P,

Piedagnel R. LG3 fragment of endorepellin is a possible biomarker of severity in

IgA nephropathy. Proteomics. 2013 Jan;13(1):142-52. doi: 10.1002/pmic.201200267.

Epub 2012 Dec 18. PubMed PMID: 23161552.

212: Svenningsson P, Westman E, Ballard C, Aarsland D. Cognitive impairment in

patients with Parkinson's disease: diagnosis, biomarkers, and treatment. Lancet

Neurol. 2012 Aug;11(8):697-707. doi: 10.1016/S1474-4422(12)70152-7. Review.

PubMed PMID: 22814541.

213: Sáez J, Martínez J, Trigo C, Sánchez-Payá J, Griñó P, Compañy L, Laveda R,

Penalva JC, García C, Pérez-Mateo M. A comparative study of the activation

peptide of carboxypeptidase B and trypsinogen as early predictors of the severity

of acute pancreatitis. Pancreas. 2004 Jul;29(1):e9-14. PubMed PMID: 15211118.

214: Taneja S, Sen S, Gupta VK, Aggarwal R, Jameel S. Plasma and urine biomarkers

in acute viral hepatitis E. Proteome Sci. 2009 Oct 27;7:39. doi:

10.1186/1477-5956-7-39. PubMed PMID: 19860894; PubMed Central PMCID: PMC2773234.

215: Taranta-Janusz K, Wasilewska A, Dębek W, Waszkiewicz-Stojda M. Urinary

cytokine profiles in unilateral congenital hydronephrosis. Pediatr Nephrol. 2012

Nov;27(11):2107-13. doi: 10.1007/s00467-012-2230-9. Epub 2012 Jun 29. PubMed

PMID: 22744767; PubMed Central PMCID: PMC3461208.

216: Tarasiuk J, Kułakowska A, Drozdowski W, Kornhuber J, Lewczuk P. CSF markers

in amyotrophic lateral sclerosis. J Neural Transm. 2012 Jul;119(7):747-57. doi:

10.1007/s00702-012-0806-y. Epub 2012 May 4. Review. PubMed PMID: 22555610.

217: Thuijls G, Derikx JP, van Wijck K, Zimmermann LJ, Degraeuwe PL, Mulder TL,

Van der Zee DC, Brouwers HA, Verhoeven BH, van Heurn LW, Kramer BW, Buurman WA,

Heineman E. Non-invasive markers for early diagnosis and determination of the

severity of necrotizing enterocolitis. Ann Surg. 2010 Jun;251(6):1174-80. doi:

10.1097/SLA.0b013e3181d778c4. PubMed PMID: 20485148.

218: Titan SM, Vieira JM Jr, Dominguez WV, Moreira SR, Pereira AB, Barros RT,

Zatz R. Urinary MCP-1 and RBP: independent predictors of renal outcome in

macroalbuminuric diabetic nephropathy. J Diabetes Complications. 2012

Nov-Dec;26(6):546-53. doi: 10.1016/j.jdiacomp.2012.06.006. Epub 2012 Sep 12.

PubMed PMID: 22981148.

219: Tokushige N, Markham R, Crossett B, Ahn SB, Nelaturi VL, Khan A, Fraser IS.

Discovery of a novel biomarker in the urine in women with endometriosis. Fertil

Steril. 2011 Jan;95(1):46-9. doi: 10.1016/j.fertnstert.2010.05.016. Epub 2010 Jun

18. PubMed PMID: 21168580.

220: Tousoulis D, Oikonomou E, Siasos G, Chrysohoou C, Charakida M, Trikas A,

Siasou Z, Limperi M, Papadimitriou ED, Papavassiliou AG, Stefanadis C. Predictive

value of biomarkers in patients with heart failure. Curr Med Chem.

2012;19(16):2534-47. Review. PubMed PMID: 22489715.

221: Valenti G, Laera A, Pace G, Aceto G, Lospalluti ML, Penza R, Selvaggi FP,

Chiozza ML, Svelto M. Urinary aquaporin 2 and calciuria correlate with the

severity of enuresis in children. J Am Soc Nephrol. 2000 Oct;11(10):1873-81.

PubMed PMID: 11004218.

222: Valero V, Forbes J, Pegram MD, Pienkowski T, Eiermann W, von Minckwitz G,

Roche H, Martin M, Crown J, Mackey JR, Fumoleau P, Rolski J, Mrsic-Krmpotic Z,

Jagiello-Gruszfeld A, Riva A, Buyse M, Taupin H, Sauter G, Press MF, Slamon DJ.

Multicenter phase III randomized trial comparing docetaxel and trastuzumab with

docetaxel, carboplatin, and trastuzumab as first-line chemotherapy for patients

with HER2-gene-amplified metastatic breast cancer (BCIRG 007 study): two highly

active therapeutic regimens. J Clin Oncol. 2011 Jan 10;29(2):149-56. doi:

10.1200/JCO.2010.28.6450. Epub 2010 Nov 29. PubMed PMID: 21115860.

223: van der Lubbe N, Jansen PM, Salih M, Fenton RA, van den Meiracker AH, Danser

AH, Zietse R, Hoorn EJ. The phosphorylated sodium chloride cotransporter in

urinary exosomes is superior to prostasin as a marker for aldosteronism.

Hypertension. 2012 Sep;60(3):741-8. doi: 10.1161/HYPERTENSIONAHA.112.198135. Epub

2012 Jul 30. PubMed PMID: 22851731.

224: van Holten TC, Waanders LF, de Groot PG, Vissers J, Hoefer IE, Pasterkamp G,

Prins MW, Roest M. Circulating biomarkers for predicting cardiovascular disease

risk; a systematic review and comprehensive overview of meta-analyses. PLoS One.

2013 Apr 22;8(4):e62080. doi: 10.1371/journal.pone.0062080. Print 2013. Review.

PubMed PMID: 23630624; PubMed Central PMCID: PMC3632595.

225: Vassiliadis E, Barascuk N, Didangelos A, Karsdal MA. Novel cardiac-specific

biomarkers and the cardiovascular continuum. Biomark Insights. 2012;7:45-57. doi:

10.4137/BMI.S9536. Epub 2012 May 2. PubMed PMID: 22577298; PubMed Central PMCID:

PMC3347891.

226: Ventetuolo CE, Levy MM. Cardiac biomarkers in the critically ill. Crit Care

Clin. 2011 Apr;27(2):327-43. doi: 10.1016/j.ccc.2010.12.004. Review. PubMed PMID:

21440204.

227: Viale G, Regan MM, Dell'Orto P, Mastropasqua MG, Maiorano E, Rasmussen BB,

MacGrogan G, Forbes JF, Paridaens RJ, Colleoni M, Láng I, Thürlimann B, Mouridsen

H, Mauriac L, Gelber RD, Price KN, Goldhirsch A, Gusterson BA, Coates AS; BIG

1-98 Collaborative and International Breast Cancer Study Groups. Which patients

benefit most from adjuvant aromatase inhibitors? Results using a composite

measure of prognostic risk in the BIG 1-98 randomized trial. Ann Oncol. 2011

Oct;22(10):2201-7. doi: 10.1093/annonc/mdq738. Epub 2011 Feb 18. PubMed PMID:

21335417; PubMed Central PMCID: PMC3179413.

228: Vij R, Noth I. Peripheral blood biomarkers in idiopathic pulmonary fibrosis.

Transl Res. 2012 Apr;159(4):218-27. doi: 10.1016/j.trsl.2012.01.012. Epub 2012

Feb 2. Review. PubMed PMID: 22424426; PubMed Central PMCID: PMC3308120.

229: Vijlbrief DC, Benders MJ, Kemperman H, van Bel F, de Vries WB. Use of

cardiac biomarkers in neonatology. Pediatr Res. 2012 Oct;72(4):337-43. doi:

10.1038/pr.2012.88. Epub 2012 Jul 13. Review. PubMed PMID: 22797141.

230: Villanova F, Di Meglio P, Nestle FO. Biomarkers in psoriasis and psoriatic

arthritis. Ann Rheum Dis. 2013 Apr;72 Suppl 2:ii104-10. doi:

10.1136/annrheumdis-2012-203037. Review. PubMed PMID: 23532439.

231: Vilmar AC, Santoni-Rugiu E, Sørensen JB. Class III β-tubulin in advanced

NSCLC of adenocarcinoma subtype predicts superior outcome in a randomized trial.

Clin Cancer Res. 2011 Aug 1;17(15):5205-14. doi: 10.1158/1078-0432.CCR-11-0658.

Epub 2011 Jun 20. PubMed PMID: 21690572.

232: Vives-Pi M, Takasawa S, Pujol-Autonell I, Planas R, Cabre E, Ojanguren I,

Montraveta M, Santos AL, Ruiz-Ortiz E. Biomarkers for diagnosis and monitoring of

celiac disease. J Clin Gastroenterol. 2013 Apr;47(4):308-13. doi:

10.1097/MCG.0b013e31827874e3. Review. PubMed PMID: 23388848.

233: Wai-Hoe L, Wing-Seng L, Ismail Z, Lay-Harn G. Proteomics and detection of

uromodulin in first-time renal calculi patients and recurrent renal calculi

patients. Appl Biochem Biotechnol. 2009 Oct;159(1):221-32. doi:

10.1007/s12010-008-8503-x. Epub 2009 Jan 15. PubMed PMID: 19145410.

234: Wang J, Hoekstra JG, Zuo C, Cook TJ, Zhang J. Biomarkers of Parkinson's

disease: current status and future perspectives. Drug Discov Today. 2013

Feb;18(3-4):155-62. doi: 10.1016/j.drudis.2012.09.001. Epub 2012 Sep 11. Review.

PubMed PMID: 22982303; PubMed Central PMCID: PMC3557745.

235: Wang L, Ni Z, Xie Z, Yang F, He B, Liu J, Dai H, Qian J, Jia M. Analysis of

the urine proteome of human contrast-induced kidney injury using two-dimensional

fluorescence differential gel electrophoresis/matrix-assisted laser desorption

time-of-flight mass spectrometry/liquid chromatography mass spectrometry. Am J

Nephrol. 2010;31(1):45-52. doi: 10.1159/000255439. Epub 2009 Nov 2. PubMed PMID:

19887787.

236: Wang LW, Han XM, Chen CH, Ma Y, Hai B. Urinary brain-derived neurotrophic

factor: a potential biomarker for objective diagnosis of overactive bladder. Int

Urol Nephrol. 2014 Feb;46(2):341-7. doi: 10.1007/s11255-013-0540-x. Epub 2013 Aug

28. PubMed PMID: 23982767.

237: Wang Y, Zhao S, Loyd S, Groome LJ. Increased urinary excretion of nephrin,

podocalyxin, and βig-h3 in women with preeclampsia. Am J Physiol Renal Physiol.

2012 May 1;302(9):F1084-9. doi: 10.1152/ajprenal.00597.2011. Epub 2012 Feb 1.

PubMed PMID: 22301621.

238: Wasilewska A, Zoch-Zwierz W, Taranta-Janusz K, Kołodziejczyk Z. Urinary

monocyte chemoattractant protein-1 excretion in children with glomerular

proteinuria. Scand J Urol Nephrol. 2011 Feb;45(1):52-9. doi:

10.3109/00365599.2010.526140. Epub 2010 Nov 1. PubMed PMID: 21034351.

239: Wasilewska A, Zoch-Zwierz W, Taranta-Janusz K, Michaluk-Skutnik J.

Neutrophil gelatinase-associated lipocalin (NGAL): a new marker of cyclosporine

nephrotoxicity? Pediatr Nephrol. 2010 May;25(5):889-97. doi:

10.1007/s00467-009-1397-1. PubMed PMID: 20072790.

240: Wenzel SE. Tissue-based and bronchoalveolar lavage-based biomarkers in

asthma. Immunol Allergy Clin North Am. 2012 Aug;32(3):401-11. doi:

10.1016/j.iac.2012.06.011. Epub 2012 Jul 12. Review. PubMed PMID: 22877618.

241: Wilson CP, McNulty H, Ward M, Strain JJ, Trouton TG, Hoeft BA, Weber P, Roos

FF, Horigan G, McAnena L, Scott JM. Blood pressure in treated hypertensive

individuals with the MTHFR 677TT genotype is responsive to intervention with

riboflavin: findings of a targeted randomized trial. Hypertension. 2013

Jun;61(6):1302-8. doi: 10.1161/HYPERTENSIONAHA.111.01047. Epub 2013 Apr 22.

PubMed PMID: 23608654.

242: Wolff AC, Hammond ME, Hicks DG, Dowsett M, McShane LM, Allison KH, Allred

DC, Bartlett JM, Bilous M, Fitzgibbons P, Hanna W, Jenkins RB, Mangu PB, Paik S,

Perez EA, Press MF, Spears PA, Vance GH, Viale G, Hayes DF; American Society of

Clinical Oncology; College of American Pathologists. Recommendations for human

epidermal growth factor receptor 2 testing in breast cancer: American Society of

Clinical Oncology/College of American Pathologists clinical practice guideline

update. J Clin Oncol. 2013 Nov 1;31(31):3997-4013. doi: 10.1200/JCO.2013.50.9984.

Epub 2013 Oct 7. PubMed PMID: 24101045.

243: Wu J, Wang N, Wang J, Xie Y, Li Y, Liang T, Wang J, Yin Z, He K, Chen X.

Identification of a uromodulin fragment for diagnosis of IgA nephropathy. Rapid

Commun Mass Spectrom. 2010 Jul 30;24(14):1971-8. doi: 10.1002/rcm.4601. PubMed

PMID: 20552702.

244: Xing Q, Su H, Cui J, Wang B. Role of Treg cells and TGF-β1 in patients with

systemic lupus erythematosus: a possible relation with lupus nephritis. Immunol

Invest. 2012;41(1):15-27. doi: 10.3109/08820139.2011.578189. Epub 2011 May 12.

PubMed PMID: 21563925.

245: Xuejing Z, Jiazhen T, Jun L, Xiangqing X, Shuguang Y, Fuyou L. Urinary TWEAK

level as a marker of lupus nephritis activity in 46 cases. J Biomed Biotechnol.

2012;2012:359647. doi: 10.1155/2012/359647. Epub 2012 Jun 6. PubMed PMID:

22719208; PubMed Central PMCID: PMC3375113.

246: Yang CC, Hsieh SC, Li KJ, Wu CH, Lu MC, Tsai CY, Yu CL. Urinary neutrophil

gelatinase-associated lipocalin is a potential biomarker for renal damage in

patients with systemic lupus erythematosus. J Biomed Biotechnol.

2012;2012:759313. doi: 10.1155/2012/759313. Epub 2012 Feb 15. PubMed PMID:

22500106; PubMed Central PMCID: PMC3303691.

247: Yilmaz A, Bilge I, Kiyak A, Gedikbasi A, Sucu A, Aksu B, Emre S, Sirin A.

Matrix metalloproteinase 9 and tissue inhibitor of metalloproteinase 1 in

vesicoureteral reflux. Pediatr Nephrol. 2012 Mar;27(3):435-41. doi:

10.1007/s00467-011-2026-3. Epub 2011 Oct 5. PubMed PMID: 21969093.

248: Younes A, Berry DA. From drug discovery to biomarker-driven clinical trials

in lymphoma. Nat Rev Clin Oncol. 2012 Nov;9(11):643-53. doi:

10.1038/nrclinonc.2012.156. Epub 2012 Sep 11. Review. PubMed PMID: 22965151.

249: Zhang X, Nagaraja HN, Nadasdy T, Song H, McKinley A, Prosek J, Kamadana S,

Rovin BH. A composite urine biomarker reflects interstitial inflammation in lupus

nephritis kidney biopsies. Kidney Int. 2012 Feb;81(4):401-6. doi:

10.1038/ki.2011.354. Epub 2011 Oct 12. PubMed PMID: 21993584; PubMed Central

PMCID: PMC3640330.

250: Zheng M, Lv LL, Ni J, Ni HF, Li Q, Ma KL, Liu BC. Urinary

podocyte-associated mRNA profile in various stages of diabetic nephropathy. PLoS

One. 2011;6(5):e20431. doi: 10.1371/journal.pone.0020431. Epub 2011 May 31.

PubMed PMID: 21655212; PubMed Central PMCID: PMC3105067.

251: Zhou FD, Zhang LX, Yao Y, Wang SX, Zou WZ, Liu G, Chen M, Zhao MH.

Immunofixation electrophoresis was highly specific for the diagnosis of renal

light-chain amyloidosis. Am J Med Sci. 2013 Jan;345(1):18-21. doi:

10.1097/MAJ.0b013e31824e0ec5. PubMed PMID: 22627261.

252: Zhou M, Liu Y, Duan Y. Breath biomarkers in diagnosis of pulmonary diseases.

Clin Chim Acta. 2012 Nov 12;413(21-22):1770-80. doi: 10.1016/j.cca.2012.07.006.

Epub 2012 Jul 14. Review. PubMed PMID: 22796631.

253: Shoji M. Biomarkers of the Dementia. Int J Alzheimers Dis. 2011;2011. doi:10.4061/2011/564321

Part B: 658 biomarker conference abstracts from American Society of Clinical Oncology annual meeting in 1995-2013

| 1 | Alpha-fetoprotein-concanavalin a binding as a marker to discriminate between germ cell tumors (gct) and liver diseases (Meeting abstract). JM Tabernero, JR Germa, J Mora, N Gascon, C Sola, E Marcuello, J Brunet, R Mesia, P Maroto, A Lopez, JJ Lopez 1995 ASCO Annual Meeting ,26. |
| --- | --- |
| 2 | Keratin 19 as a marker for non-small cell lung cancer (NSCLC) and small cell lung cancer (SCLC) (Meeting abstract). AM Dingemans, van Ark-Otte J, van der Valk P, PE Postmus, G Giaccone . 1995 ASCO Annual Meeting ,1147. |
| 3 | Multiple marker polymerase chain reaction assay for evaluating circulating melanoma cells (Meeting abstract). PS Dale, Y Wang, A Conrad, L Foshag, C Kuo, D Garrison, A Nize, DL Morton, DS Hoon . 1995 ASCO Annual Meeting ,1308. |
| 4 | Mini- and microsatellite DNA markers reveal clonal intratumor heterogeneity in gastrointestinal cancers (Meeting abstract). S Nagel, A Tobler, M Oestreicher, B Borisch, F Nothiger, MF Fey . 1995 ASCO Annual Meeting ,313. |
| 5 | Evaluation of markers of bone metabolism after pamidronate (APD) infusion in patients with metastatic bone disease (Meeting abstract). J Vinholes, CY Guo, OP Purohit, R Eastell, R Coleman . 1995 ASCO Annual Meeting ,50. |
| 6 | OVX1, a potential prognostic marker for prostate cancer (Meeting abstract). FJ Xu, JT Hsieh, YH Yu, C Logothetis, Bast RC Jr . 1995 ASCO Annual Meeting ,610. |
| 7 | Post-therapy changes in biochemical markers in patients with androgen independent prostate cancer (Meeting abstract). WK Kelly, G Steineck, M Mazumdar, V Vlamis, A Dnistrian, H Scher . 1995 ASCO Annual Meeting ,606. |
| 8 | CYFRA 21.1, a diagnostic marker in malignant pleural effusion (Meeting abstract). H Saka, K Shimokata, H Minami, T Shibagaki, A Watanabe, H Saito, S Sakai . 1995 ASCO Annual Meeting ,32. |
| 9 | MDR1 gene expression as predictive markers of clinical response in breast cancer treated by primary chemotherapy (Meeting abstract). S Chevillard, P Vielh, JY Pierga, P Pouillart . 1995 ASCO Annual Meeting ,41. |
| 10 | Tumor marker decay as prognostic factor during treatment of male germ cell tumors. A retrospective analysis (Meeting abstract). E Mickiewicz, C Brosio, P Giglio, A Alvarez, N Piris, G Cinat, L Ezcurdia . 1995 ASCO Annual Meeting ,49. |
| 11 | Utility of the serum tumor marker neuron specific enolase (NSE) in small cell lung cancer (SCLC) (Meeting abstract). E Shaw, G Klee, J Grill, J Jett, K Rowland . 1995 ASCO Annual Meeting ,29. |
| 12 | Pro-gastrin-releasing peptide (31-98) as a tumor marker of small-cell lung cancer: comparative evaluation with neuron-specific enolase (Meeting abstract). M Takada, Y Kusunoki, N Masuda, K Matui, T Yana, S Ushijima, K Iida, K Tamura, T Komiya, I Kawase, al et . 1995 ASCO Annual Meeting ,1161. |
| 13 | Comparison between two groups of breast cancer patients' submitted or not to a rational postoperative follow-up with tumor markers (Meeting abstract). A Nicolini, L Anselmi, G Piacentini, F Ducci . 1995 ASCO Annual Meeting ,199. |
| 14 | Salvage therapy with ifosfamide, cisplatin and vinblastine (VeIP) or etoposide (VIP) for germ cell carcinoma patients initially treated with PEB: a tumor marker analysis (Meeting abstract). WC Mertens, MJ Taylor . 1995 ASCO Annual Meeting ,666. |
| 15 | Poor survival in plasmablastic myeloma in Eastern Cooperative Oncology Group study E9487: cell kinetic, ploidy, biological marker, and clinical correlations (Meeting abstract). PR Greipp, JM Bennett, JP Gaillard, B Klein, T Leong, MM Oken, NE Kay, Van Ness B, RA Kyle . 1995 ASCO Annual Meeting ,1355. |
| 16 | Biochemical markers of bone metastases (Meeting abstract). LM Demers, L Costa, V Chinchilli, L Gaydos, E Curley, H Harvey, A Lipton . 1995 ASCO Annual Meeting ,33. |
| 17 | Evaluation of prostate specific antigen (PSA) as a surrogate marker for response of hormone refractory prostate cancer (HRPC) to suramin therapy (Meeting abstract). R Sridhara, M Eisenberger, V Sinibaldi, L Reyno, M Egorin . 1995 ASCO Annual Meeting ,40. |
| 18 | Soluble interleukin-2 receptor is a powerful prognostic marker in patients with malignant lymphomas (Meeting abstract). M Gnant, RM Mader, M Djavanmard, R Jakesz, GG Steger . 1995 ASCO Annual Meeting ,1214. |
| 19 | The 'virulence' and 'metastagenicity' of breast carcinoma as studied using biochemical markers (Meeting abstract). R Heimann, D Ferguson, C Powers, W Recant, R Weichselbaum, S Hellman . 1995 ASCO Annual Meeting ,221. |
| 20 | The management of patients with clinical Stage A testicular nonseminomatous germ cell tumors (NSGCT) and persistently elevated serologic markers (Meeting abstract). SB Saxman, CR Nichols, RS Foster, JE Messemer, JP Donohue, LH Einhorn . 1995 ASCO Annual Meeting ,595. |
| 21 | Identification of biological markers selecting patients candidates to primary hormonotherapy (HT) for operable breast cancer (Meeting abstract). Di Leo A, E Bajetta, C Bartoli, L Biganzoli, S Pilotti, G Vicario, S Orefice, R Saccozzi, L Celio . 1996 ASCO Annual Meeting ,45. |
| 22 | Increased p53 immunoreactivity is a marker for increased survival and paclitaxel/radiation sensitivity in patients (pts) with glioblastoma multiforme (GBM) (Meeting abstract). MH Friedberg, CH Rhodes, M Glantz, BF Cole, L Glantz, W Akerley, L Recht, P Mills, H Choy . 1996 ASCO Annual Meeting ,301. |
| 23 | A rational noninvasive schedule with tumor markers, bone scanning (BS) and 'aimed' computed tomography (CT) or skeletal x-ray for 'early' detection of bone metastases in breast cancer (Meeting abstract). A Nicolini, P Ferrari, L Anselmi, MR Metelli, C Colombini, M Ferdeghini . 1996 ASCO Annual Meeting ,189. |
| 24 | Tumor marker half-life (MHL) complements pretreatment risk stratification in metastatic nonseminomatous germ cell tumors (NSGCT) (Meeting abstract). A Gerl, R Lamerz, C Clemm, K Mann, R Hartenstein, M Hentrich, W Wilmanns . 1996 ASCO Annual Meeting ,599. |
| 25 | Immunohistochemical staining for markers of future neoplastic progression in the larynx (Meeting abstract). D Uhlman, G Adams, D Knapp, G Niehans . 1996 ASCO Annual Meeting ,878. |
| 26 | Urine basic fibroblast growth factor as a tumor marker for ovarian cancer (Meeting abstract). Van Le L, X He, WC Fowler, LA Walton . 1996 ASCO Annual Meeting ,790. |
| 27 | Could preoperative plasma level of tumor-associated antigens (tumor markers) be a useful independent predictor of prognosis for gastric cancer patients undergoing curative resection? (Meeting abstract). J Sakamoto, H Nakazato, S Maetani, S Teramukai, Y Ohashi, Y Takahashi, M Mai, T Toge, H Okura, S Kodaira, K Okajima, K Inokuchi . 1996 ASCO Annual Meeting ,1610. |
| 28 | Nasal non-Hodgkin's lymphomas (NHLs): expression of the natural killer (NK) cell marker CD56 predicts a worse prognosis (Meeting abstract). M Cheung, JK Chan, WH Lau, PT Chan, W Foo, R Ngan . 1996 ASCO Annual Meeting ,1305. |
| 29 | Detection of melanoma cells in bone marrow by multiple-marker polymerase chain reaction assay (Meeting abstract). T Sarantou, DL Morton, D Garrison, A Conrad, DS Hoon . 1996 ASCO Annual Meeting ,1375. |
| 30 | Proper surgery in epithelial ovarian cancer stage I and II. Value of serum tumor markers (STM) (Meeting abstract). B Ojeda, P Maroto, F Cruz, MC Alonso, C Sola, R Salazar, M Bellet, E Delgado, J Badia, Lopez Lopez JJ . 1996 ASCO Annual Meeting ,788. |
| 31 | HER-2/neu and p53 expression in breast cancer: valid prognostic markers when assessed by direct immunoassay, but not by immunohistochemistry (Meeting abstract). TJ Hieken, RR Mehta, A Shilkaitis, LD Wild, Das Gupta TK . 1996 ASCO Annual Meeting ,113. |
| 32 | Effect of neoadjuvant chemotherapy in breast cancer with expression of the molecular markers P53 and HER2/neu and correlation with clinical outcome (Meeting abstract). A Mangalik, L Dressler, L Lafrado . 1996 ASCO Annual Meeting ,1818. |
| 33 | Clinical relevance of serum tumor markers in malignant melanoma: a comparison between S-100 and CD44 (Meeting abstract). F. Grossi, P. Queirolo, F. Cafiero, M. Bergaglio, A. Peressini, S. Vecchio, I. Ribizzi, S. Bertoglio, M.R. Sertoli, P. Marroni and M. Paganuzzi . 1997 ASCO Annual Meeting ,1771. |
| 34 | Effects of pamidronate on tumor marker levels in breast and prostate cancer: correlation with clinical and biochemical response (Meeting abstract). R Coleman, J Vinholes, O Purohit, A Milford-Ward . 1997 ASCO Annual Meeting ,1179. |
| 35 | Effect of selected prognostic markers on node-negative breast cancer patients with ER-positive tumors: 10-year results from NSABP B-14 (Meeting abstract). B Fisher, J Costantino, J Bryant, N Gunduz, E Fisher, E Mamounas, A DeCillis, DL Wickerham, S Paik, N Dimitrov, D Bowman, R Margolese, M Kavanah, H Shibata . 1997 ASCO Annual Meeting ,455. |
| 36 | Bile acid-induced apoptosis: a promising marker for colorectal cancer risk (Meeting abstract). H. Garewal, C. Bernstein, H. Bernstein, P. Dinning, R. Jabi, R. Sampliner, P. Krasnovich, C. Payne . 1997 ASCO Annual Meeting ,1996. |
| 37 | A multiple marker RT-PCR assay for detection of occult metastasis in frozen sections of sentinel nodes from breast cancer patients (Meeting abstract). P Bostick, T Sarantou, K Huynh, A Conrad, S Chawla, A Giuliano, D Hoon . 1997 ASCO Annual Meeting ,536. |
| 38 | Tumor markers status at relapse in patients with germ cell tumors (Meeting abstract). JM Trigo, JM Tabernero, L Paz-Ares, JL Garcia-Llano, J Mora, P Lianes, E Esteban, R Salazar, J Lopez-Lopez, H Cortes-Funes . 1997 ASCO Annual Meeting ,1238. |
| 39 | Interleukin-6 (IL-6): a marker of androgen-independent (AI) growth in prostatic carcinoma (PC) (Meeting abstract). D Daliani, L Finn, S Hodges, C Wood, C Logothetis . 1997 ASCO Annual Meeting ,1183. |
| 40 | Multivariate analysis of tissue-based prognostic markers in stage II-III colorectal carcinoma (Meeting abstract). JM Jessup, IC Summerhayes, D Shibata, G Cangi, PT Lavin, AM Mercurio, F Fogt, M Loda . 1997 ASCO Annual Meeting ,910. |
| 41 | Molecular markers in male breast cancer (Meeting abstract). D Rayson, C Erlichman, LE Wold, PC Roche, VJ Suman, GA Croghan, JH Donohue, JN Ingle . 1997 ASCO Annual Meeting ,477. |
| 42 | Stratified multivariate survival analysis (SMSA) of prognostic markers in breast cancer (BrCa) (Meeting abstract). M Younes, M Lane, CC Miller, R Laucirica . 1997 ASCO Annual Meeting ,698. |
| 43 | Correlation between serum tumor markers (TM) and stage: study in 75 patients (pts) with non-small cell lung cancer (NSCLC) (Meeting abstract). JA Barbuto, RN Younes, EA Teixeira, NR Takimoto, JL Gross, A Anelli, D Deheinzelin . 1997 ASCO Annual Meeting ,1659. |
| 44 | Performance of serum tumor markers in detection of metastatic breast cancer: does lack of positive markers indicate poor prognosis? (Meeting abstract). JM Mayo, FP Arena . 1997 ASCO Annual Meeting ,638. |
| 45 | Elevated urinary creatine excretion (% CR) is a marker of steroid-induced myopathy in patients with malignant gliomas (Meeting abstract). GJ Lesser, KS O'Rourke . 1997 ASCO Annual Meeting ,1442. |
| 46 | Molecular detection of pancreatic carcinoma (PC) with a multiple marker RT-PCR assay (Meeting abstract). MC Kelley, P Bostick, A Bilchik, LJ Foshag, R Essner, C Kuo, A Conrad, PD Boasberg, DS Hoon . 1997 ASCO Annual Meeting ,1041. |
| 47 | Serum tissue polypeptide antigen (S-TPA) in bladder cancer as a tumor marker: a prospective study (Meeting abstract). C Maulard-Durdux, ME Toubert, C Hennequin, M Housset . 1997 ASCO Annual Meeting ,1204. |
| 48 | C-POB for germ cell tumor (GCT) patients (pts) with prolonged half-life of markers on BEP induction chemotherapy (Meeting abstract). S Tjulandin, D Titov, A Sokolov, V Sholokhov, D Nosov, V Goutnik, A Garin . 1997 ASCO Annual Meeting ,1237. |
| 49 | Serum level of the p53 antigen as a marker during follow-up of colon cancer patients (Meeting abstract). A Shani, B Sandler, P Smirnoff, R Idelevich, R Pfefferman, I Zusman . 1997 ASCO Annual Meeting ,1085. |
| 50 | Elevated soluble tumor necrosis factor receptors: a powerful marker predicting relapse and poor prognosis of ovarian cancer patients after normalization of the Ca-125 following adjuvant chemotherapy (Meeting abstract). D Aderka, G Sabag, V Granot, D Wallach, Y Ovadia, H Levavi . 1997 ASCO Annual Meeting ,1282. |
| 51 | Tumor markers (CEA, SCC, CYFRA, CA 125, NSE) in response monitoring and prognosis of non-small cell lung cancer (Meeting abstract). Vi帽olas N, Molina R, Gal谩n MC, Callejas MA, Casas F, Montserrat JM, Filella X, Grau JJ, Estap茅 J . 1997 ASCO Annual Meeting ,1751. |
| 52 | Proliferating cell nuclear antigen (PCNA) and tumor grade as markers of virulence in node-negative breast carcinoma (NNBC) (Meeting abstract). R Heimann, D Ferguson, C Powers, W Recant, S Hellman . 1997 ASCO Annual Meeting ,604. |
| 53 | SERUM BETA 2-MICROGLOBULIN: A PREDICTIVE MARKER OF OUTCOME IN EPITHELIAL OVARIAN CANCER (Meeting abstract). M Marinaccio, Rocca AMV L, S Geusa, V Pinto, G Lanzilotti, M Panarelli, S Selvaggio, Marino E D . 1998 ASCO Annual Meeting ,1420. |
| 54 | GAGE: A NOVEL MARKER FOR MICROSCOPIC HUMAN CANCERS (Meeting abstract). IY Cheung, NK Cheung, R Ghossein, D Coit, J Rosai . 1998 ASCO Annual Meeting ,2117. |
| 55 | TUMOR MARKER EXPRESSION IN PATIENTS WITH MULTIPLE GYNECOLOGIC MALIGNANCIES (Meeting abstract). SJ Yang, S Rafla, S Hirschman, K Parikh, J Pedersen . 1998 ASCO Annual Meeting ,1455. |
| 56 | NEUROENDOCRINE MARKER AS A PREDICTOR OF EARLY PROGRESSION IN ANDROGEN-INDEPENDENT PROSTATE CANCER (AIPC) (Meeting abstract). J Kim, L Finn, S Hodges, N Hoosein, L Deftos, C Logothetis . 1998 ASCO Annual Meeting ,1213. |
| 57 | C-ERBB2 IS A MARKER OF RESISTANCE TO ENDOCRINE THERAPY IN ADVANCED BREAST CANCER (Meeting abstract). TA Plunkett, SJ Houston, DM Barnes, RD Rubens, DW Miles . 1998 ASCO Annual Meeting ,397. |
| 58 | PREDICTIVE MOLECULAR MARKERS FOR CLINICAL OUTCOME FOLLOWING PRIMARY CHEMOTHERAPY FOR OPERABLE BREAST CANCER (Meeting abstract). J Chang, TJ Powles, DC Allred, GM Clark, A Makris, RK Gregory, SE Ashley, CK Osborne, M Dowsett . 1998 ASCO Annual Meeting ,384. |
| 59 | PLATELET SEROTONIN IS THE MOST SENSITIVE MARKER FOR DIAGNOSIS OF CARCINOID TUMORS (Meeting abstract). WG Meijer, IP Kema, PH Willemse, M Volmer, Vries EGE d . 1998 ASCO Annual Meeting ,1143. |
| 60 | MOLECULAR MARKERS IN HEREDITARY BREAST CANCER (Meeting abstract). R Nanda, M Adeyanju, S Cummings, L Sveen, S Turner-Thompson, Q Gao, OI Olopade . 1998 ASCO Annual Meeting ,2115. |
| 61 | MUTATED KRAS DNA IN PLASMA AS A MARKER IN EARLY COLORECTAL NEOPLASIA (Meeting abstract). MS Kopreski, FA Benko, DJ Borys, A Khan, CM Kwee, TJ McGarrity, CD Gocke . 1998 ASCO Annual Meeting ,1033. |
| 62 | EFFECT OF SELECTED PROGNOSTIC MARKERS ON HIGH RISK STAGE II AND III BREAST CANCER TREATED IN A RANDOMISED DOSE-INTENSIFIED ADJUVANT PROTOCOL (Meeting abstract). G Konecny, C Thomssen, A Lebeau, S Kahlert, D Sattler, E Piche, P Dettmar, W Kuhn, F Janicke . 1998 ASCO Annual Meeting ,584. |
| 63 | THE UTILITY OF TUMOUR MARKERS IN ASSESSING THE RESPONSE TO CHEMOTHERAPY IN ADVANCED BLADDER CANCER (Meeting abstract). AM Cook, RA Huddart, G Jay, A Norman, DP Dearnaley, A Horwich . 1998 ASCO Annual Meeting ,1199. |
| 64 | PROGNOSTIC MARKERS IN RESECTED STAGE I AND II NON-SMALL CELL LUNG CANCER: AN ANALYSIS OF 260 PATIENTS WITH 5 YEAR FOLLOW-UP (Meeting abstract). SL Graziano, SA Mehdi, A Tatum, NB Newman, BJ Poiesz, J Kern, G Gamble, J Etzel, N Weidner, LJ Kohman . 1998 ASCO Annual Meeting ,1772. |
| 65 | THE EVALUATION OF p53, Her-2/neu AND SERIAL MDR PROTEIN EXPRESSION AS POSSIBLE MARKERS OF CHEMORESISTANCE AND THEIR USE AS PROGNOSTIC MARKERS IN INFLAMMATORY BREAST CANCER (Meeting abstract). B Pro, M Cristofanilli, AU Buzdar, N Sneige, B Wasaff, L Asmar, R Theriault, SE Singletary, M McNeese, E Strom, E Rivera, GN Hortobagyi . 1998 ASCO Annual Meeting ,2119. |
| 66 | INTERMEDIATE MARKERS IN ASSESSING RESPONSE TO VACCINE THERAPIES (Meeting abstract). HI Scher, SF Slovin, WK Kelly, PO Livingston, S Danishefsky, M Fazzari, K Terry, G Heller . 1998 ASCO Annual Meeting ,1247. |
| 67 | PLASMA UROKINASE PLASMINOGEN ACTIVATOR (UPA) MAY BE USED AS A TUMOR MARKER FOR BREAST CANCER AND COLON CANCER (Meeting abstract). M Al-Bassam, Y Wu, R Chillar, JV Vadgama . 1998 ASCO Annual Meeting ,2171. |
| 68 | BREAST CANCER (BC) MICROMETASTASES AS A POTENTIAL SURROGATE MARKER FOR EFFICACY OF ADJUVANT TREATMENT: DOCETAXEL-EPIRUBICIN (DE) COMBINATION vs EC/CMF, A PHASE II RANDOMIZED STUDY (Meeting abstract). S Braun, CRM Kentenich, W Janni, F Hepp, J de Waal, HL Sommer . 1998 ASCO Annual Meeting ,499. |
| 69 | EFFECTS OF THE BISPHOSPHONATES PAMIDRONATE, CLODRONATE AND IBANDRONATE ON CD 69, AN EARLY T-LYMPHOCYTE ACTIVATION MARKER (Meeting abstract). M Pecherstorfer, B Hammerl, R Jilch, E Horn, H Ludwig . 1998 ASCO Annual Meeting ,1719. |
| 70 | ELEVATED SERUM BONE SIALOPROTEIN IN PRIMARY BREAST CANCER PATIENTS IS A POTENT MARKER FOR BONE METASTASES (Meeting abstract). IJ Diel, EF Solomayer, H Meisenbacher, Ch Gollan, R Conradi, D Wallwiener, G Bastert . 1998 ASCO Annual Meeting ,467. |
| 71 | PHASE I TRIAL OF PYRAZOLOACRIDINE (PZA), A POTENT DNA BINDING AGENT, GIVEN AS A WEEKLY 24 HOUR CONTINUOUS INTRAVENOUS INFUSION (CI) IN ADULT PATIENTS WITH REFRACTORY SOLID TUMORS (Meeting abstract). B Monahan, M Quinn, C Takimoto, A Chen, JM Hamilton, N Harold, G Morrison, D Nguyen, J Grem . 1998 ASCO Annual Meeting ,736. |
| 72 | Is erbB-2 a Predictive Marker for Response to Primary Chemotherapy (CT) for Operable Breast Cancer? (BC) Prospective Study in a Phase II Randomized, Parallel Study of Doxorubicin/Cyclophosphamide (AC) and Doxorubicin/Taxol[trade] (Paclitaxel) (AT). (Meeting abstract). Veroniqu Dieras, Cremoux Patricia D, Doussal V L, Ann Vincent-Salomon, M Tubiana-Hulin, JY Pierga, F Spyratos, Pierr Pouillart . 1999 ASCO Annual Meeting ,322. |
| 73 | Elevated Serum Cardiac Troponin-T as a Marker for Active Cardiac Injury During Therapy for Childhood Acute Lymphoblastic Leukemia (ALL) (Meeting abstract). S Lipshultz, S Sallan, V Dalton, S Arslanian, G Zou, S Shaikh, B Asselin, R Gelber, N Rifai . 1999 ASCO Annual Meeting ,2191. |
| 74 | Elevated ([Up Arrow]) Serum Tumor Markers (STM) Prior to 1[deg] RPLND Predicts Clinical Outcome and Requirements for Systemic Chemotherapy in Patients with PN1, N2 and N3 Nonseminomatous Germ Cell Tumor (NSGCT) (Meeting abstract). J Sheinfeld, F Rabbani, H Mohseni, H Herr, D Bajorin, R Motzer, G Bosl . 1999 ASCO Annual Meeting ,1184. |
| 75 | Plasma 2'Deoxyruidine (dUrd) as a Surrogate Marker of Thymidylate Synthase (TS) Inhibition in Patients Treated with ZD9331. (Meeting abstract). AL Jackman, F Mitchell, S Lynn, C Noyce, C Rees, IR Judson, AH Calvert, R Plummer, M Hutchison, M Smith . 1999 ASCO Annual Meeting ,654. |
| 76 | Evaluation of a New Immunological Marker TGT (TURTEST[Superscript [trade]]) in the Diagnosis of Lung Cancer (Meeting abstract). A Berlin, C Chiaffitelli, V Erkhov, V Maximenko, I Bakhlaev, E Oleinik, A Luongo . 1999 ASCO Annual Meeting ,1837. |
| 77 | Concordance of Biological Markers and Presentation in Bilateral Breast Cancer (Meeting abstract). H Ashamalla, A Youssef, SJ Yang, E Toni, M Khalil . 1999 ASCO Annual Meeting ,330. |
| 78 | In Breast Cancer Patients the Post-Operative Follow-Up with CEA-TPA-CA15.3 Tumor Marker Panel Is Cost Effective (Meeting abstract). A Nicolini, A Carpi, P Ferrari, L Anselmi . 1999 ASCO Annual Meeting ,373. |
| 79 | Biochemical Markers of Bone Turnover Correlate with The Extent of Metastatic Bone Disease (Meeting abstract). L Costa, LM Demers, T Speicher, A Gouveia, E Curley, H Harvey, EB Costa, A Lipton . 1999 ASCO Annual Meeting ,2375. |
| 80 | Molecular Markers of Treatment Resistance or Sensitivity in Esophageal Cancer Patients. (Meeting abstract). Harpole David , Moore Mary-Beth , Aloia Thomas , Sporn Thomas , Conlon Debbi , Wolfe Walter , D'Amico Thomas . 1999 ASCO Annual Meeting ,1497. |
| 81 | Molecular Markers To Predict Survival and Benefit from Adjuvant Intraportal 5FU in Colon Cancer. (Meeting abstract). Barratt Paula , Seymour Matt , Sall Stenning, Birbeck Kevin , Phi Quirke . 1999 ASCO Annual Meeting ,1030. |
| 82 | Relationship Between Biological Markers and Response to Primary Chemotherapy in Breast Cancer. (Meeting abstract). Blasio Beatrice D, Rit Nizzoli, Marcell Flora, Robert Camisa, Luis Savoldi, Beretta Myriam , Caterin Caminiti, Corrad Boni, Giorgi Cocconi . 1999 ASCO Annual Meeting ,2425. |
| 83 | Reevaluation of Red Blood Cell Polyamines as a Tumor Activity Marker for Glioma Disease. (Meeting abstract). Valer Nasser, Emmanue Chirpaz, Mireill Mousseau, Edmon Chambaz, Ren Schaerer, Alim-Loui Benabid, Francoi Berger . 1999 ASCO Annual Meeting ,584. |
| 84 | Circulating Neuroendocrine Markers in Prostate Cancer Patients (Meeting abstract). Marc Tampellini, Alfred Berruti, Alessandr Mosca, Gabriell Gorzegno, Mirell Torta, Michel Stivanello, Luig Dogliotti . 1999 ASCO Annual Meeting ,1217. |
| 85 | The Preoperative Soluble VEGF Level Is a Prognostic Marker in Patients with Colorectal Carcinoma. (Meeting abstract). K Werther, IJ Christensen, HJ Nielsen . 1999 ASCO Annual Meeting ,791a . |
| 86 | PCR-Positive mRNAs in Peripheral Blood of Patients with Malignant Melanoma as an Effective Tumor Progression Marker (Meeting abstract). G Palmieri, M Strazzullo, PA Ascierto, SM Satriano, M Pisano, A Daponte, C Caraco, N Mozzillo, G Castello . 1999 ASCO Annual Meeting ,2077. |
| 87 | Phase I Study of CT-2584 in Patients with Solid Tumors. Clinical Results Correlate with a Novel Surrogate Marker: Plasma Oxidized Free Fatty Acids (OFFA) Measured as Conjugated Dienes. (Meeting abstract). Roge Waltzman, Stuar Bursten, Jac Singer, Davi Spriggs . 1999 ASCO Annual Meeting ,893. |
| 88 | Carcinoembryonic Antigen Level in Peritoneal Washing Is a Useful Marker for Peritoneal Metastasis in Patients with Gastric Cancer (Meeting abstract). Masanor Terashima, Akinor Takagane, Kaor Abe, Munemits Araya, Hitosh Yonezawa, Takash Irinoda, Tsutom Nakaya, Osam Shimooki, Kennich Oyama, Hisatak Fujiwara, Naok Sasaki, Kazuyosh Saito . 1999 ASCO Annual Meeting ,1156. |
| 89 | A Novel Anti-Apoptosis Gene: Re-Expression of survivin Messenger RNA as a Prognosis Marker in Non-Small-Cell Lung Cancers (Meeting abstract). R Rosell, M Monzo, E Felip, J Astudillo, JJ Sanchez, I Rosas, C Martin, A Font, A Barnadas, A Abad . 1999 ASCO Annual Meeting ,1901. |
| 90 | Serum Interleukin-6 (IL-6) Concentration Correlates with Serum Tumor Marker CA15-3 Concentration in Breast Cancer Patients (Meeting abstract). JF Head, XP Jiang, DC Yang, RL Elliott . 1999 ASCO Annual Meeting ,. |
| 91 | Microphthalmia Transcription Factor: A Highly Sensitive and Specific Diagnostic Marker for Melanoma (Meeting abstract). Weilbaecher Katherine , Ro King, Gae McGill, Edwar Cooley, Marti Mihm, Fisher David . 1999 ASCO Annual Meeting ,2084. |
| 92 | Phase I Trial of ZD9331 in Adult Patients with Refractory Solid Malignancies Administered by 30-Minute Infusion on Days 1 and 8 with Cycle Repeated Every 3 Weeks. (Meeting abstract). R Plummer, C Rees, I Judson, H Calvert, A Hughes, M Highley, P Beale, J Trigo, A Jackman, R Smith, M Hutchison, M Smith . 1999 ASCO Annual Meeting ,861. |
| 93 | Ig V Gene Mutations and CD38 Expression Are Prognostic Markers in Chronic Lymphocytic Leukemia (Meeting abstract). T Wasil, Damle R , F Fais, A Valetto, Allen S , Lichtman S , P Schulman, Vinciguerra V , Rai K , M Ferrarini, N Chiorazzi . 1999 ASCO Annual Meeting ,25. |
| 94 | Serum Levels of Inhibins a And B Are Not Markers of Placental Tumors (Meeting abstract). C Lhomme, P Pautier, S Ghione, F Petraglia, JM Bidart . 1999 ASCO Annual Meeting ,2448. |
| 95 | LEA.135 Expression: Its Comparison with Other Prognostic Markers for Patients with Primary Breast Carcinoma (Meeting abstract). A Battayan, WY Naritoku, SS Imam, D Tsao-Wei, SL Groshen, LJ Young, RD Cardiff, CR Taylor, SA Imam . 1999 ASCO Annual Meeting ,2377. |
| 96 | P53 and BCL-2, but Not BCL-6 Are Poor Prognostic Molecular Markers in Diffuse Large B-Cell Lymphomas. (Meeting abstract). Kang Yoon , Jooryun Huh, Lim So , Park Sung , Lee Seung , Kim Bong , Kim Tae , Ryu Min , Ryu Baek , Lee Kyu , Heo Dae , Kim Chul . 1999 ASCO Annual Meeting ,2442. |
| 97 | Analysis at More Than 10 Year Follow-Up of Prognostic Markers Including P53 in Node Negative Breast Cancer Patients (Meeting abstract). JM Ferrero, A Ramaioli, JL Formento, M Francoual, MC Etienne, F Ettore, E Teissier, P Leblanc-Talent, M Namer, G Milano . 1999 ASCO Annual Meeting ,2379. |
| 98 | The Value of MIB-1, C-ERB and p53 in Early Breast Cancer -- Correlation with Conventional Prognostic Markers and Disease Free Interval (DFS). (Meeting abstract). Shailendr Verma, Simon Dahrouge, Vivie Frenkel, Susa Robertson, Christin Pratt, Louis Pigeon . 1999 ASCO Annual Meeting ,2474. |
| 99 | Thymidylate Synthase and MRP MRNA Expression in Tumor and Healthy Tissude as Markers for Response Prediction in Head and Neck Squamous Cell Carcinoma. (Meeting abstract). M Barberi-Heyob, S Drouard, JL Merlin, D Bagrel, M Dauca, G Dolivet, C Dubessy, L Geoffrois, C Vigneron, S Wagner, M Wellman, T Conroy . 1999 ASCO Annual Meeting ,1510. |
| 100 | Incidence of Skeletal Complications in Prostate Cancer Patients with Hormone Refractory Disease. Predictive Role of Bone Resorption and Formation Markers Evaluated at Baseline (Meeting abstract). Alfred Berruti, Raffaell Bitossi, Gabriell Gorzegno, Mirell Torta, Marc Tampellini, Luig Dogliotti, Albert Angeli . 1999 ASCO Annual Meeting ,1205. |
| 101 | Sunbelt Melanoma Trial: Sensitivity and Specificity of Reverse Transcriptase-Polymerase Chain Reaction (RT-PCR) Markers for Sentinel Lymph Nodes (SLN) (Meeting abstract). KM McMasters, DS Reintgen, MI Ross, A Conrad, J Albrecht, DN Krag, V Viar, PB Cerrito, MJ Edwards . 1999 ASCO Annual Meeting ,2073. |
| 102 | An International Study on the Utility of "Melanoma-Inhibiting Activity"- (MIA-) and S-100B-Protein as Valid Tumor Markers of Metastatic Melanoma in Serum (Meeting abstract). A Hauschild, Bonfrer J , P Stieber, L- Hannson . 1999 ASCO Annual Meeting ,2055. |
| 103 | PCNA and KI-67 as Markers of Parathyroid Carcinomas. H Takami, K Kameyama, Y Ikeda, M Niimi, K Ito . 2000 ASCO Annual Meeting ,1681. |
| 104 | CEA: An Irrelevant Serum Marker for Breast Cancer. M Roselli, P Ferroni, S Carlini, M Abbolito, S Mariotti, A Spila, S Aloe, R D'Alessandro, M Carone, A Cicchetti, A Ricciotti, F Di Filippo . 2000 ASCO Annual Meeting ,376. |
| 105 | Micropthalmia Transcription Factor: A New Prognostic Marker in Intermediate-Thickness Cutaneous Malignant Melanoma. G. Salti, T. Manougian, M. Farolan, A. Shilkaitis, TK. Das Gupta . 2000 ASCO Annual Meeting ,2195. |
| 106 | Utility of 99mTc-Sestamibi as a Marker of Multidrug Resistance (MDR) in Patients with Metastatic Medullary Thyroid Cancer (MTC). Said Saleh, David Goldenberg, David Gold, Rosalyn Blumenthal, Dion Yeldell, Rhona Stein, George Hajjar, Malik Juweid . 2000 ASCO Annual Meeting ,702. |
| 107 | Microsatellite Instability Is a Strong Molecular Predictive Marker for Sensitivity to Adjuvant Chemotherapy in Dukes鈥?C Colorectal Carcinoma. Hany Elsaleh, David Joseph, Barry Iacopetta . 2000 ASCO Annual Meeting ,922. |
| 108 | ProMMP-1 Levels as Marker for Tumor Aggressiveness in Colon Cancer Patients. Baruch Klein, B Kfir, E Sadikov, M Mishaeli, T Klein . 2000 ASCO Annual Meeting ,1071. |
| 109 | Modulation of Molecular Marker Expression by Induction Chemotherapy in Locally Advanced Breast Cancer: Correlation with the Response to Treatment and Axillary Node Invasion. Jos茅 Schneider, Alvaro Ruibal, Armando Tejerina, Ra煤l Lucas, Jaime S谩nchez, Miguel Mart铆n . 2000 ASCO Annual Meeting ,2607. |
| 110 | Limitations of Serum Tumor Marker Measurement in Evaluating the Response to Chemotherapy in Patients with Advanced Gastric Cancer. Tatsu Shimoyama, Yasuhide Yamada, Kuniaki Shirao, Yasuhiro Matsumura, Kei Muro, Masahiro Gotoh, Tetsuya Hamaguchi, William Hrushesky, Atsushi Ohtsu, Narikazu Boku . 2000 ASCO Annual Meeting ,1263. |
| 111 | HER-2 as a Predictive Marker in Node-Positive (N+) Breast Cancer (BC) Patients (Pts) Randomly Treated with CMF or an Anthracycline-Based Regimen. Angelo Di Leo, Denis Larsimont, David Gancberg, Tero J盲rvinen, Marc Beauduin, Stella Dolci, Marianne Paesmans, Jean-Pierre Lobelle, Jorma Isola, Martine J. Piccart . 2000 ASCO Annual Meeting ,371. |
| 112 | Presence of Activation Markers of EBV and CMV in Myelodysplasia. Suneel Mundle, K Allampallam, B Mativi, B Dangerfield, J Cartlidge, S Alvi, C Shetty, S Dar, E Broderick, P Vengopal, S Gregory, A Raza . 2000 ASCO Annual Meeting ,110. |
| 113 | Manganese Superoxide Dismutase Expression as Predictive Marker for Disease-Free Survival in Head and Neck Squamous Cell Carcinoma. Muriel Barberi-Heyob, St茅phane Drouard, Jean-Louis Merlin, Denise Bagrel, Philippe Becuwe, Arnaud Bianchi, Michel Dauca, Gilles Dolivet, Lionel Geoffrois, C Vigneron, Maya Wellman, Thierry Conroy . 2000 ASCO Annual Meeting ,1694H . |
| 114 | Serum Tumor Markers CA-125, CEA and CYFRA 21-1 in Malignant Pleural Mesothelioma. P Berzinec, H Zuffova, I Tudik . 2000 ASCO Annual Meeting ,2622. |
| 115 | Significance of Biological Markers in Patients (PTS) with Advanced Gastric Cancer Treated with 5-FU + CDDP (FP) and 5-FU Continuous Infusion (CI). Narikazu Boku, Atsushi Ohtsu, Kuniaki Shirao, Yasuhiro Shimada, Shigeaki Yoshida . 2000 ASCO Annual Meeting ,1022. |
| 116 | The Effects of Raltitrexed ('Tomudex'), ZD9331 ('Vamidex') And 5-FU On Plasma dUrd, A Surrogate Marker of Thymidylate Synthase Inhibition. Ann Jackman, Hugo Ford, David Farrugia, Fraser Mitchell, Mark Hill, Ian Judson, David Cunningham . 2000 ASCO Annual Meeting ,712. |
| 117 | Cytokeratin 20 Expression by Reverse Transcription Polymerase Chain Reaction (RT-PCR) as an Early Marker for Gastrointestinal Tumor Metastasis : Correlation with Clinical Staging. Concepcion Risue帽o, Marcelo Garrido, Eric Orellana, Maribel Capriles, Manuel Alvarez . 2000 ASCO Annual Meeting ,2589. |
| 118 | Neoadjuvant Arimidex or Tamoxifen, Alone or Combined, for Breast Cancer (IMPACT): PgR-Related Reductions in Proliferation Marker KI67. Irene Boeddinghaus, M. Dowsett, I. Smith, M. Verrill, T. Hickish, T. Skene, S. Ebbs, W. Allum . 2000 ASCO Annual Meeting ,360. |
| 119 | D5S679 Allele 6 Seems to Be a Specific Microsatellite Marker for Small Cell Lung Cancer (SCLC). Sacide Pehlivan, Ugur Yilmaz, Mustafa Pehlivan, Meral Koyuncuoglu, Sacide Karacali, Atilla Akkoclu . 2000 ASCO Annual Meeting ,1930. |
| 120 | The IgM Antibody Response to Melanoma Vaccine Immunization Is an Intermediate Marker of Clinical Effectiveness. Zhong Xie, Sandra Reynold, Ruth Oratz, Richard Shapiro, Matthew Harris, Daniel Roses, Jean-Claude Busreyn . 2000 ASCO Annual Meeting ,2192. |
| 121 | Assessment of XIAP (X-Linked Inhibitor of Apoptosis) as a Novel Prognostic Marker in Radically Resected Non-Small-Cell Lung Cancer (NSCLC) Patients. , Carlos Ferreira, Paul Valk, Inge Ludwig, Simone Span, Giuseppe Giaccone . 2000 ASCO Annual Meeting ,1945. |
| 122 | Comparison of Antigalactosil ?1-3galactose (Antigal) Antibody with the CA 15-3 Tumor Marker in Breast Cancer Patients. D. Hernandez-Morales, L. Fernandez, M. Guzman, M. Sanchez, J. Avila . 2000 ASCO Annual Meeting ,2615. |
| 123 | Bone-Specific Alkaline Phosphatase in Plasma as Tumour Marker for Osteosarcoma in Childhood. I. Ilhan, S. Berberoglu, I. Unsal . 2000 ASCO Annual Meeting ,2344. |
| 124 | Scatter Factor/Hepatocyte Growth Factor as a Serum Marker for Prostate Cancer. Michael Naughton, Joel Picus, Xiaopei Zhu, William Catalona, Robin Vollmer, Peter Humphrey . 2000 ASCO Annual Meeting ,1331. |
| 125 | DNA of Human Papilloma Virus in Plasma of Patients with Cervical Cancer. A Potential Marker of Minimal Residual Disease. Marcela Lizano, Adela Carrillo, Ewa W贸jcik, Alfonso Duenas, Jaime De la Garza . 2000 ASCO Annual Meeting ,1526. |
| 126 | Plasma Carboxyterminal Telopeptide of Collagen as a Potential Marker of Bone Resorption in Breast Cancer. Pascal Houz茅, Paul Cottu, F Bouro, Michel Marty, Bernard Gourmel, B Bousquet . 2000 ASCO Annual Meeting ,387. |
| 127 | Comparison of the Effects of Clodronate and Pamidronate on Bone Resorption Markers and Symptoms in Metastatic Bone Disease. Om Purohit, Satinder Jagdev, Sarah Heatley, Christian Herling, Robert Coleman . 2000 ASCO Annual Meeting ,2425. |
| 128 | The Prognostic Value of Traditional Clinical Pathologic Factors (TF) and Molecular Markers (MM) in Early Breast Cancer (EBC). Shailendra Verma, Simone Dahrouge, Vivien Frenkel, Susan Robertson, Louise Pigeon, Christine Pratt . 2000 ASCO Annual Meeting ,2586. |
| 129 | PCR-Based Analysis of Patients with Malignant Melanoma (MM): Assessment of Genetic Alterations and Detection of Occult Metastases as Tumor Progression Markers. Giuseppe Palmieri, Paolo Ascierto, Sabrina Satriano, Maria Motti, Gerardo Botti, Antonio Cossu, Francesco Tanda, Nicola Mozzillo, Giuseppe Castello . 2000 ASCO Annual Meeting ,2233. |
| 130 | Evaluation of the Clinical Impact of CEA, CA 19-9 and CA 72-4 Serum Tumor Markers in Colorectal Cancer-A Prospective Longitudinal Study. Fiorella Guadagni, Mario Roselli, Sabrina Mariotti, Roberta D'Alessandro, Antonella Spila, Simona Aloe, Maria Carone, Maria Abbolito, Manfredo Tedesco, Franco Graziano, Oreste Buonomo, Maurizio Cosimelli . 2000 ASCO Annual Meeting ,940. |
| 131 | The Role of Bcl-2, Bax, Bcl-2/Bax Ratio, and P53 as Prognostic Markers in Patients (Pts) with Muscle-Invasive, Node-Negative Transitional Cell Carcinoma (Tcc) of the Bladder Treated with Neoadjuvant Methotrexate, Vinblastine, Adriamycin, and Cisplatin (M- Dean Bajorin, Fernando Maluf, Carlos Cordon-Cardo, Jaya Satagopan, David Verbel, Steven Novick, Geralyn Higgins, Mary Boyle, Harry Herr, And . 2000 ASCO Annual Meeting ,1295. |
| 132 | A Randomised Multicentre Study of Vorozole Compared to Tamoxifen as Primary Therapy in post-Menopausal Breast Cancer: Relationship of Biological Markers with Clinical Response. Catherine Harper-Wynne, Karyn Shenton, Roger A'Hern, Fiona MacNeil, Paul Sauven, Ian Laidlaw, Zen Rayter, Stephanie Miall, Nigel Sacks, Mitchell Dowsett . 2000 ASCO Annual Meeting ,358. |
| 133 | The Impact of Paclitaxel-Based Chemotherapy on the Reverse Transcriptase Polymerase Chain Reaction (RT-PCR) Detection Rate of Prostatic Specific Markers in Androgen-Independent Prostate Cancer (AIPC) Patients. Iman Osman, R Ghossein, H Scher, D Verbel, R Petrisko, S Bhattacharya, W Kelly . 2000 ASCO Annual Meeting ,1371. |
| 134 | Prediction of Risk of Relapse in Patients with Germ Cell Tumours Undergoing Retroperitoneal Lymph Node Dissection Using Immuno-Histochemical Markers in Patients with Evidence of Malignant Elements in the Resection Specimens. Jonathan Shamash, Dan Berney, Ajay Arora, Rtd Oliver . 2000 ASCO Annual Meeting ,1375. |
| 135 | Pathological Response to Paclitaxel/Radiation in Locally Advanced Breast Cancer: Association with Molecular Markers. Silvia Formenti, Kristin Skinner, Kathy Danenberg, Darcy Spicer, Susan Groshen, Peter Danenberg . 2000 ASCO Annual Meeting ,356. |
| 136 | YKL-40, as a Prognostic Tumor Marker in Recurrent Ovarian Cancer. H. Dehn, E. V. S. H酶gdall, J. S. Johansen, J. A. Price, M. J酶rgensen, S. A. A. Engelholm, C. K. H酶gdall, Rigshospitalet, University of Copenhagen, Copenhagen, Denmark; Institute of Cancer Epidemiology, The Danish Cancer Society, Copenhagen, Denmark; Hvidovre Hospital, Hvidovre, Denmark; Department of Biology, University of California, San Diego, La Jolla, CA. . 2001 ASCO Annual Meeting ,2508. |
| 137 | Gleason Score as a Prognostic Marker of Survival in Men with Advanced Prostate Cancer. Scott M. Dresden, Karin B. Olson, Kenneth J. Pienta, University of Michigan, Ann Arbor, MI. . 2001 ASCO Annual Meeting ,2422. |
| 138 | A New Marker for Cervical Cancer Screening. Svetomir Markovic, Olivera Markovic, James Sundeen, Shelly Parr, Mario Belledonne, Nenad Markovic, Mayo Clinic, Rochester, MN; BioSciCon, Rockville, MD; Diagnostic Pathology Services Inc., Rockville, MD; University of Maryland, College Park, MD; Biolab Research, Rockville, MD. . 2001 ASCO Annual Meeting ,855. |
| 139 | Use of Multiple Markers to Detect Stage I Epithelial Ovarian Cancers: Neural Network Analysis Improves Performance. Z Zhang, F Xu, Y Yu, A Berchuck, LJ Havrilesky, HWA de Bruijn, AGJ van der Zee, RP Woolas, I Jacobs, RC Bast, Jr., Medical University of South Carolina, Charleston, SC; University of Texas, MD Anderson Cancer Center, Houston, TX; Duke University Medical Center, Durham, NC; Groningen University Hospital, Groningen, AA, Netherlands; Royal London Hospital, London, UK; St. Bartholomew's Hospital, London, UK. . 2001 ASCO Annual Meeting ,873. |
| 140 | Lysophosphatidic Acid is not a Useful Marker for the Detection of Ovarian Cancer. Patton Morrison, Daniel L. Baker, Dominic M. Desiderio, Brigitte E. Miller, Gabor J. Tigyil, University of Tennessee, Memphis, Memphis, TN; Wake Forest University School of Medicine, Winston-Salem, NC. . 2001 ASCO Annual Meeting ,841. |
| 141 | Assay of Sentinel Nodes for Breast Cancer Markers Using Q-PCR. Joel Turner, Steve Shivers, Katherine Gres, Pratima Chaurasia, Wenbin Li, Marcus Stafford, Karen Fields, Charles Cox, Douglas Reintgen, H. Lee Moffitt Cancer Center, Tampa, FL. . 2001 ASCO Annual Meeting ,159. |
| 142 | Identification of New Marker Genes for Breast Cancer. Laura van 't Veer, A.J. Bosma, C.P. Schr枚der, S. Rodenhuis, The Netherlands Cancer Institute, Amsterdam, Netherlands. . 2001 ASCO Annual Meeting ,196. |
| 143 | KL-6, a New Tumor Marker for Breast Cancer. Yoshinari Ogawa, Osaka City University Medical School, Osaka, Japan. . 2001 ASCO Annual Meeting ,1704. |
| 144 | Sequential Evaluation of Multiple Potential Bio-Markers in Patients with Metastatic Prostate Cancer (PCA) Following Androgen Deprivation. Mario A. Eisenberger, Menachem Laufer, Lori Sokoll, Victoria Sinibaldi, Marianna Zahurak, David Crawford, Johns Hopkins University, Baltimore, MD; University of Colorado, Denver, CO. . 2001 ASCO Annual Meeting ,758. |
| 145 | Plasma Big ET-1 没a Tumour Marker and Potential Surrogate Marker for Angiogenesis in Lung Cancer. CHOKKALINGAM ARUN, K E Porter, G McMohan, N J M London, K J O'Byrne, D M Hemingway, University of Leicester, Leicester, UK. . 2001 ASCO Annual Meeting ,3101. |
| 146 | Carbonic Anhydrase XII: a Marker of Good Prognosis in Invasive Breast Cancer. Stephen K.L. Chia, Peter H. Watson, Charles C. Wykoff, Cheng Han, Russell D. Leek, William S. Sly, Kevin C. Gatter, Peter Ratcliffe, Adrian L. Harris, British Columbia Cancer Agency, Vancouver, BC, Canada; University of Manitoba, Winnipeg, MB, Canada; ICRF Molecular Oncology Laboratory, University of Oxford, Oxford, UK; St. Louis University School of Medicine, St. Louis, MO; University of Oxford, Oxford, UK; Welcome Trust, Oxford, UK. . 2001 ASCO Annual Meeting ,1658. |
| 147 | Development of Surrogate Markers of Response for Antiangiogenic Therapies Utilizing Non-Invasive and Invasive Assessments of Tumor Angiogenesis. Robert L. Capizzi, Eduardo D. De Moraes, Derrick Grant, Miriam Wahl, Dennis Leeper, Steven McKenzie, Flemming Forsberg, Barry Goldberg, Donald Mitchell, Jefferson Medical College, Philadelphia, PA. . 2001 ASCO Annual Meeting ,2090. |
| 148 | Replication Error Positive Phenotype: a Potential Prognostic Marker in Breast Cancer. Anees Chagpar, Annette Kerviche, Leonard Tan, Anthony Magliocco, Barb Walley, John DeCoteau, University of Saskatchewan, Saskatoon, SK, Canada; University of Calgary, Calgary, AB, Canada. . 2001 ASCO Annual Meeting ,1702. |
| 149 | Selection of markers for Immunocytochemical detection of circulating melanoma cells Manuel Valladares-Ayerbes, Pilar Iglesias, Mar Haz, Silvia Antol铆n, Rosario Campelo, Guillermo Alonso, Lourdes Calvo, Luis Ant贸n-Aparicio, Juan Canalejo Hospital, La Coru帽a Spain . 2001 ASCO Annual Meeting ,3133. |
| 150 | Characterization of the Variability and Prognostic Value of Repeated Measurements of the CA125 Tumor Marker in a Large Ovarian Cancer Clinical Trial. Howard T Thaler, Larry J Copeland, Maury Markman, Memorial Sloan-Kettering Cancer Center, New York, NY; James Cancer Hospital, Ohio State U, Columbus, OH; Cleveland Clinic Foundation, Cleveland, OH. . 2001 ASCO Annual Meeting ,806. |
| 151 | Mammographic Density Correlates with Gail Model: Implications for a New Marker of Breast Cancer Risk. Melanie R. Palomares, Kelley D. Pratt, Constance D. Lehman, Anne McTiernan, University of Washington, Seattle, WA; Fred Hutchinson Cancer Research Center, Seattle, WA. . 2001 ASCO Annual Meeting ,1689. |
| 152 | The Identification of Molecular Markers in Breast Cancer Metastasis to Bone. Susan M. Poelman, Alexander H.G. Paterson, Douglas J. Demetrick, Anthony M. Magliocco, University of Calgary, Calgary, AB, Canada. . 2001 ASCO Annual Meeting ,1693. |
| 153 | Cerebrospinal Fluid Levels of Temozolomide as a Surrogate Marker for Brain Penetration. Roger Stupp, Sandrine Ostermann, Serge Leyvraz, Chantal Csajka, Thierry Buclin, Laurent A Decosterd, University Hospital CHUV, Lausanne, Switzerland. . 2001 ASCO Annual Meeting ,232. |
| 154 | Cross-Linked Carboxyterminal Telopeptide of Type I Collagen as Useful Marker for Bone Metastasis in Patients with Primary Lung Cancer. Hirofumi Oda, Tatsuhiko Kashii, Toshiro Miwa, Nobuki Arai, Shoko Matsui, Muneharu Maruyama, Naohiro Yamashita, Masashi Kobayashi, Toshiki Tatsumura, Toyama Medical and Pharmaceutical University, Toyama, Japan. . 2001 ASCO Annual Meeting ,3076. |
| 155 | Combination of Unexpressed p21 and High p53 Phenotype: a Newly Prognostic/Predictive Molecular Marker (MM) in Metastatic Breast Cancer (MBC) Patients (Pts) Treated with Two Different Regimens Containing Cisplatin (P) or Epirubicin (E). Franco Nole, Elisabetta Munzone, Giuseppe Renne, Patrick Maisonneuve, Chiara Catania, Elena Verri, Ida Minchella, Giuseppe Viale, Aron Goldhirsch, European Institute of oncology, Milan, Italy. . 2001 ASCO Annual Meeting ,3124. |
| 156 | Clinical Evaluation of Tumor Angiogenesis Markers in Metastatic Cancer. Walter Michael Stadler, Ruth Heimann, Gregory Karczmar, Thomas Gajewski, Hedy Kindler, Peter MacEneany, Marta Zamora, Milica Medved, Everett E Vokes, University of Chicago, Chicago, IL. . 2001 ASCO Annual Meeting ,382. |
| 157 | Thymidylate Synthase Expression in Colorectal Cancer . 2001 ASCO Annual Meeting ,492. |
| 158 | A Pilot Study of Troponin T as a Prognostic Marker in Patients Treated with 5-Flurouracil. Shona Milon Nag, Karen Briscoe, Paul Desouza, M.N.B cancer institute, Pune, India; Cancer Care centre, St. George Hospital, Sydney, Australia. . 2001 ASCO Annual Meeting ,3126. |
| 159 | Serum Markers Add to Traditional Prognostic Factors in Metastatic Breast Cancer. Kim E Leitzel, Suhail M Ali, Vernon Chinchilli, Linda Engle, Laurence Demers, Harold H Harvey, Carolyn Brady, Walter Carney, Gabriella B Cook, Debra Cambetas, Jeffrey Allard, Allan Lipton, Penn State/Hershey Medical Center, Hershey, PA; Novartis Corp., East Hanover, NJ; Bayer Corp., Cambridge, MA. . 2001 ASCO Annual Meeting ,1700. |
| 160 | Molecular Markers Affect Survival Following Adjuvant Treatment for Resected Hepatic Metastases from Colorectal Cancer (CRC). Amanda Hummer, Nancy Kemeny, Alice Zervoudakis, Carlos Cordon-Cardo, David Klimstra, Deidre Sullivan, Yuman Fong, Mithat Gonen, Memorial Sloan-Kettering Cancer Center, New York, NY. . 2001 ASCO Annual Meeting ,516. |
| 161 | Survival of Metastatic Breast Cancer Patients: Importance of Initial Prognostic Markers in 654 Patients. Jenny Chang, Gary M Clark, D Craig Allred, C Kent Osborne, Richard M Elledge, Breast Center at Baylor College of Medicine, Houston, TX. . 2001 ASCO Annual Meeting ,168. |
| 162 | Isotype Switching Events and the Presence of Pre-Switch Clonotypic Cells Within the Myeloma Clone Correlate with Clinical Markers of Disease and Survival. Tony Reiman, Brian J Taylor, Karen Seeberger, John Hanson, Robert W Coupland, Andrew R Belch, Linda M Pilarski, Cross Cancer Institute, University of Alberta, Edmonton, AB, Canada. . 2001 ASCO Annual Meeting ,1192. |
| 163 | Results of Post Chemotherapy Retroperitoneal Lymph Node Dissection for Patients with Persistantly Elevated Tumor Markers. Richard Foster, Stephen Beck, Richard Bihrle, Lawrence Einhorn, John Donohue, Indiana University School of Medicine, Indianapolis, IN. . 2001 ASCO Annual Meeting ,713. |
| 164 | Expression of Bcl-2, CD95, VEGF, Ki-67 and COX-2 in Barrett's Esophagus and Esophageal Carcinoma: Markers for Esophageal Cancer Chemoprevention Trials. Koyamangalath Krishnan, Dawn Arnett, George Youngberg, East Tennessee State University and James H. Quillen VA Medical Center, Johnson City, TN; East Tennessee State University, Johnson City, TN. . 2001 ASCO Annual Meeting ,2276. |
| 165 | Sputum cytology as a marker of risk for lung cancer- preliminary results from the University of Colorado high risk cohort study Fred R Hirsch, Sheila A Prindiville, Tim Byers, York E Miller, Wilbur A Franklin, Robert Keith, Holly J Wolf, Paul A Bunn Jr., Timothy C Kennedy, Univ of Colorado Cancer Center, Denver, CO. . 2002 ASCO Annual Meeting ,1201. |
| 166 | CA15-3 level as a prognostic marker in multiple myeloma (MM) patients receiving thalidomide (T) Linda R Mileshkin, H M Prince, James J Biagi, Paul Mitchell, David Westerman, Craig Underhill, Andrew Grigg, Richard Bell, Joe McKendrick, Peter Briggs, John F Seymour, Peter MacCallum Cancer Institute, Melbourne, VI, Australia; Austin and Repatriation Medical Centre, Melbourne, Australia; Border Medical Oncology, Albury, VI, Australia; Royal Melbourne Hospital, Melbourne, Australia; The Geelong Hospital, Geelong, VI, Australia; Box Hill Hospital, Box Hill, VI, Australia; Monash Medical Centre, Clayton, Australia. . 2002 ASCO Annual Meeting ,1105. |
| 167 | TA-90 immune complex: a new tumor marker for early detection of subclinical metastatic melanoma Rishab K Gupta, Eddy C Hsueh, Mark C Kelley, Donald L Morton, John Wayne Cancer Inst at Saint Johns Health Center, Santa Monica, CA. . 2002 ASCO Annual Meeting ,1352. |
| 168 | Breast density is a marker for family history of breast cancer Elad Ziv, John Shepherd, Rebecca Smith Bindman, Karla Kerlikowske, Univ of California San Francisco, San Francisco, CA. . 2002 ASCO Annual Meeting ,1732. |
| 169 | The bone resorption marker Ntx is strongly correlated with skeletal events in metastatic bone disease and is influenced by dose escalation of clodronate Janet E Brown, Susan Ellis, Sandra Gutcher, Catherine Thompson, Pra-Kash Purohit, Robert E Coleman, Cancer Research Centre, Weston Park Hospital, Sheffield, UK. . 2002 ASCO Annual Meeting ,1537. |
| 170 | CD44 exon v7-8 is a prognostic marker in cervical cancer Angelos Koutras, Shashikant O Lele, Marilyn Intengan, Farzana Habib, Deborah Driscoll, Kunle Odunsi, General Prefectural Hospital, Cyros, Greece; Roswell Park Cancer Inst, Buffalo, NY. . 2002 ASCO Annual Meeting ,868. |
| 171 | Use of the tumour marker CA19-9 for monitoring response to immunotherapy for advanced pancreatic cancer Andrew Gilliam, Susan A Watson, Mark Henwood, Brian Rowlands, Paul Broome, Dov Michaeli, Michael Meyers, Ian Beckingham, University of Nottingham, Nottingham, UK; Aphton Corp, Chorleywood, UK; Aphton Corp, Woodland, CA; Aventis Pharmaceuticals, Bridgewater, NJ. . 2002 ASCO Annual Meeting ,2981. |
| 172 | Phase II translational marker study of p53 sequence variations in ovarian cancer John A Green, Jo Thompson-Hehir, Maria van der Burg, Els M Berns, Gemma Kenter, Paul van Diest, Marc van de Vijver, Rene Verheijen, Ivana Teodorovic, Ignace Vergote, for the Translational Research Group of the EORTC Gynaecological Cancer Cooperative Group, Brussels, Belgium. . 2002 ASCO Annual Meeting ,841. |
| 173 | Markers of tumor-related hypoxia (TRH) in a phase I study of tirapazamine (TPZ) plus taxol &amp; carboplatin (carbo) Cynthia L Martel, Primo N Lara Jr., Paul H Gumerlock, Philip C Mack, Irina V Galvin, Derick H Lau, Corinne Turrell, Ann F Chambers, Sylvia M Wilson, Quynh Le, Heins-Josef Lenz, James H Doroshow, David R Gandara, UC Davis Medical Center, Sacramento, CA; London Regional Cancer Center, London, ON, Canada; Stanford University, Palo Alto, CA; University of Southern California, Los Angeles, CA; City of Hope Medcl Ctr, Duarte, CA. . 2002 ASCO Annual Meeting ,68. |
| 174 | Osteopontin - a new serum prognostic marker for head and neck squamous cell carcinomas (HNSCC). Sheue Ching T Yu, Patrick D Sutphin, Albert Koong, Amato J Giaccia, Quynh-Thu Le, Stanford University, Stanford, CA. . 2002 ASCO Annual Meeting ,929. |
| 175 | The relationship between surrogate markers and survival in women receiving first-line chemotherapy for advanced breast cancer Allan Hackshaw, Peter J Barrett-Lee, Robert Leonard, Alastair Knight, Wolfson Institute of Environmental &amp; Preventive Medicine, London, UK; Velindre Hospital, Cardiff, UK; Singleton Hospital, Swansea, UK; Evicom, London, UK. . 2002 ASCO Annual Meeting ,224. |
| 176 | Prognostic markers in malignant gliomas Mirta S Varela, Stella Ranuncolo, Ana M Morandi, Jos茅 M Lastiri, Elisa Bal de Kier Joff茅, Maria G Pallotta, Lydia Puricelli, Hospital Italiano, Buenos Aires, Argentina; Research Area of the Institute of Oncology Angel H. Roffo, Buenos Aires, Argentina. . 2002 ASCO Annual Meeting ,1795. |
| 177 | Genetic markers of individual chemotherapy response in non-small cell lung cancer (NSCLC): a pharmacogenomic interpretation of an Italian Lung Cancer Project randomized trial Rafael Rosell-Costa, Mariano Monzo, Silvia Novello, Miquel Taron, Jose Javier Sanchez, Lucio Crino, Corrado Boni, Filippo De Marinis, Maurizio Tonato, Maurizio Marangolo, Felice Gozzelino, Francesco Di Costanzo, Massimo Rinaldi, Jose M Sanchez, Giorgio Scagliotti, Hospital Germans Trias i Pujol, Badalona, Spain; University of Turin, Turin, Italy; Free University of Madrid, Madrid, Spain; Bellaria Hospital, Bologna, Italy; Santa Maria Nuova Hospital, Reggio Emilia, Italy; Forlanini Hospital, Rome, Italy; Policlinico Hospital, Perugia, Italy; Santa Maria delle Croci Hospital, Ravenna, Italy; Infermi Hospital, Biella, Italy; S. Maria Hospital, Terni, Italy; Regina Elena Institute, Rome, Italy. . 2002 ASCO Annual Meeting ,1199. |
| 178 | Prospective controlled trial of circulating angiogenic markers in stable and hormone-escaping advanced prostate cancer Manish Kohli Jr., Varsha Kaushal, Horace J Spencer, Paulette Mehta, Central Arkansas Veteran Healthcare System &amp; University of Arkansas for Medical Sciences, Little Rock, AR. . 2002 ASCO Annual Meeting ,788. |
| 179 | 5-HIAA versus chromogranin A as biochemical markers in the malignant carcinoid syndrome: results from a randomized multi-institutional clinical trial Lowell B Anthony, David Vance, Joseph Rubin, Jaffer A Ajani, Mats Stridsberg, LSU New Orleans, New Orleans, LA; Mayo Clinic, Rochester, MN; M.D. Anderson Cancer Center, Houston, TX; University Hospital, Uppsala, Sweden. . 2002 ASCO Annual Meeting ,664. |
| 180 | Serum genetic markers as surrogates of response to biochemotherapy in patients with metastatic melanoma Bret Taback, Steven O'Day, Patricia Fournier, Dave S Hoon, John Wayne Cancer Inst, Santa Monica, CA. . 2002 ASCO Annual Meeting ,1353. |
| 181 | Estrogen receptor (ER) status supercedes both p27 loss and Her2/neu overexpression as an age-associated prognostic marker in breast cancer (BrCa) Tracy S d'Entremont, Rosemarie Mick, Ken Simbiri, Rebecca Telep, Lisa Wray, Geza Acs, Anne Blackwood, Angela DeMichele, University of Pennsylvania, Philadelphia, PA. . 2002 ASCO Annual Meeting ,1776. |
| 182 | Analysis of molecular markers and targets in trials of depsipeptide, FR901228, a histone deacetylase inhibitor with clinical activity in T-cell lymphoma Richard Piekarz, Rob Robey, Tito Fojo, Susan Bakke, Wyndham Wilson, Elaine Jaffe, Maria Turner, Richard Cannon, Susan E Bates, National Cancer Institute, NIH, Bethesda, MD. . 2002 ASCO Annual Meeting ,88. |
| 183 | Biological markers (BM) with predictive value in metastatic breast cancer (MBC) patients (pts) randomly treated with doxorubicin (A) or docetaxel (D) Angelo Di Leo, Stephen Chan, Kay Friedrichs, Tamas Pinter, Simon Van Belle, Elizabeth Murray, Istvan Bodrogi, Euan Walpole, Bernard Lesperance, Stefan Korec, John P Crown, Peter Simmonds, Stella Dolci, Denis Larsimont, Nacer Azli, Isabelle Treilleux, Jorma Isola, Marianne Paesmans, Martine J Piccart, Jules Bordet Institute, Bruxelles, Belgium; City Hospital, Nottingham, UK; University Hospital, Hamburg, Germany; Aladar Hospital, Gyor, Hungary; UZ Hospital, Gent, Belgium; Groote Schuur Hospital, Cape Town, South Africa; National Institute of Oncology, Budapest, Hungary; Princess Alexandra Hospital, Queensland, Australia; Sacre-Coeur Hospital, Montreal, Canada; National Cancer Institute, Bratislava, Slovak Republic; St Vincents Hospital, Dublin, Ireland; General Hospital, Southampton, UK; Aventis, Paris, France; Lyon Berard Center, Lyon, France; University of Tampere, Tampere, Finland. . 2002 ASCO Annual Meeting ,213. |
| 184 | Surrogate markers for the assessment of biological activity of the VEGF-receptor inhibitor PTK787/ZK 222584 (PTK/ZK) in two clinical phase I trials Joachim Drevs, Carmen I Schmidt-Gersbach, Klaus Mross, Michael Medinger, Marianne Mueller, William Steward, Dirk Laurent, Lucy Lee, Margaret Dugan, Andrew Henry, Dieter Marme, Clemens Unger, Tumor Biology Center, Freiburg, Germany; Royal Hospital, Leicester, UK; Schering AG, Berlin, Germany; Novartis, E. Hanover, NJ. . 2002 ASCO Annual Meeting ,337. |
| 185 | The early decline of tumor markers predicts outcome in poor-prognosis non-seminomatous germ-cell tumors (NSGCT): results of a multi-institution study. Karim Fizazi, Andrew Kramar, Stephane Culine, Isan Chen, Jean-Pierre Droz, Christopher J Logothetis, Institute Gustave Roussy, Villejuif, France; Genito-Urinary Group of the French Federation of Cancer Centers (GETUG), Paris, France; UT M.D. Anderson Cancer Center, Houston, TX. . 2002 ASCO Annual Meeting ,739. |
| 186 | Real-time tumor-tracking radiation therapy (RTRT) for lung cancer by the aid of insertion of a gold marker using bronchofiberscopy Toshiyuki Harada, Hiroki Shirato, Shigeaki Ogura, Koichi Yamazaki, Rikiya Onimaru, Kei Kitamura, Kazuo Miyasaka, Masaharu Nishimura, Hirotoshi Dosaka-Akita, Hokkaido University School of Medicine, Sapporo, Japan. . 2002 ASCO Annual Meeting ,2676. |
| 187 | Tartrate-resistant acid phosphatase 5b (TRAP 5b) as a serum marker of bone metastasis in breast cancer patients Tsu-Yi Chao, Su-Huei Lee, Mary M Chen, Anthony Janckila, Lung Tam Yam, Tri-Service General Hospital, Taipei, Taiwan; Veterans Administrative Medical Center, Louisville, KY. . 2002 ASCO Annual Meeting ,3013. |
| 188 | A multivariate analysis of genetic markers for clinical response to 5-FU/oxaliplatin chemotherapy in advanced colorectal cancer. Heinz-Josef Lenz, David J Park, Wu Zhang, Denice Tsao-Wei, Susan Groshen, Sepideh Zahedy, Nalin Mallik, Ji Gil, Jan Stoehlmacher, USC Norris Cancer Ctr, Los Angeles, CA; LAC/USC Medical Center, Dept of Medicine, Los Angeles, CA. . 2002 ASCO Annual Meeting ,513. |
| 189 | Does second-line chemotherapy, performed in response to marker recurrence, improve the outcome of ovarian cancer? Kan Komai, Kurume University School of Medicine, Kurume, Japan. . 2002 ASCO Annual Meeting ,879. |
| 190 | The estrogen receptor coactivator AIB1 (SRC3) in combination with HER-2 is a prognostic and predictive marker in patients with breast cancer C. K Osborne, Valerie-Jeanne Bardou, Susan G Hilsenbeck, Suzanne A Fuqua, Torsten Hopp, Gary Chamness, Jiemin Wong, Rachel Schiff, Craig Allred, Gary Clark, Breast Center, Baylor College of Medicine, Houston, TX. . 2002 ASCO Annual Meeting ,129. |
| 191 | Is HER-2 a true or a surrogate predictive marker (PM) of response to anthracycline-based chemotherapy (CT) in metastatic breast cancer (MBC) patients? Fatima Cardoso, Chantal Bernard-Marty, Ghislaine Rouas, David Gancberg, Jean-Yves Leroy, Virginie Durbecq, Denis Larsimont, Marianne Paesmans, Martine J Piccart, Angelo Di Leo, Institut Jules Bordet, Brussels, Belgium. . 2002 ASCO Annual Meeting ,3027. |
| 192 | RhoC expression in benign and malignant breast disease: a promising marker of metastatic potential in small tumors Celina G Kleer, Kenneth L van Golen, Yanhong Zhang, Zhi-Fen Wu, Mark A Rubin, Sofia D Merajver, University of Michigan, Ann Arbor, MI. . 2002 ASCO Annual Meeting ,1695. |
| 193 | Identification of a putative intestinal stem cell and neoplastic marker Gerard Brady, Christopher S Potten, Catherine Booth, Gregory L Tudor, Dawn Booth, Patricia T Hurley, Shin-ichi Sakakibara, Hideyuki Okano, EpiStem Ltd, Manchester, UK; Department of Neuroanatomy, Biomedical Research Center, Osaka University, Osaka, Japan; Department of Physiology, Keio University School of Medicine, Tokyo, Japan. . 2002 ASCO Annual Meeting ,3028. |
| 194 | von Willebrand factor antigen may be a surrogate marker for biological effect on endothelial cells in patients with solid tumors treated with Angiozyme Paul A Masci, Radka Susan, Chris Pasco, Roger D Aitchison, Vann Parker, Nassim Usman, Ernest Borden, David Weng, Cleveland Clinic Fndtn, Cleveland, OH; Ribozyme Pharmaceuticals Inc, Boulder, CO. . 2002 ASCO Annual Meeting ,1915. |
| 195 | The role of maspin and maminin-5 as novel prognostic markers in axillary node-negative (ANN) breast cancer Massimo Cristofanilli, Aysegul Sahin, Mien C Hung, Terry L Smith, L. Diaz, D. Yu, M. Z Gilcrease, Shu Wan Kau, Gabriel N Hortobagyi, UT MD Anderson Cancer Ctr, Houston, TX. . 2002 ASCO Annual Meeting ,248. |
| 196 | Comparative value of SBR grade, hormonal receptors, KI 67, HER-2 and topo-isomerase II alpha (topo II alpha) status as predictive markers in breast cancer patients treated with neoadjuvant anthracycline-based chemotherapy Marc Wilt, R茅gine Millon, Michel Velten, Jean-Francois Rodier, Pierre Haegele, Michel Eber, Jean-Pierre Ghnassia, Simon Schraub, Thierry Petit, Paul Strauss Cancer Center, Strasbourg, France. . 2002 ASCO Annual Meeting ,123. |
| 197 | Neoadjuvant paclitaxel/radiation for stage II/III breast cancer with correlative molecular markers. Anuradha Chakravarthy, M Kelley, D H Johnson, Z Stewart, D Beauchamp, S Pearson, N Merchant, B Nicholson, R Stein, R Young, J Simpson, Y Shyr, J A Pietenpol, Vanderbilt Univ, Nashville, TN. . 2002 ASCO Annual Meeting ,271. |
| 198 | Prognostic value of defective DNA mismatch repair, p53 overexpression, proliferation and apoptosis markers in patients (pts) with resected Dukes B2 or C colon cancer: a North Central Cancer Treatment Group (NCCTG) study. Megan Garrity, Lawrence Burgart, Michelle Mahoney, Alfred F Furth, Linda Murphy, Eunice Hill, Darren Riehle, Martin Wiesenfeld, Harold Windschitl, James Krook, Maria Tria Tirona, John Michalak, Daniel Sargent, Richard M Goldberg, Michael O'Connell, Cecelia Meyers, Thomas Witzig, Mayo Clinic, Rochester, MN; North Central Cancer Treatment Group, Rochester, MN. . 2002 ASCO Annual Meeting ,510. |
| 199 | Clinical evaluation of the novel vascular-targeting agent, ZD6126: assessment of toxicity and surrogate markers of vascular damage Sandra A Radema, Laurens V Beerepoot, Petronella O Witteveen, Martijn F Gebbink, Catherine Wheeler, Emile E Voest, University Medical Center, Utrecht, Netherlands; AstraZeneca, Boston, MA. . 2002 ASCO Annual Meeting ,439. |
| 200 | p53, bcl-2 and bax immunohistochemistry in colorectal carcinogenesis: immunohistochemical markers for colorectal cancer chemoprevention trials Rebecca Cody, Sarah Whaley, George Youngberg, Koyamangalath Krishnan, James H. Quillen VA Medical Center and East Tennessee State University, Johnson City, TN. . 2002 ASCO Annual Meeting ,2335. |
| 201 | Molecular markers predictive of disease outcome in medulloblastoma. A. J. Gajjar, R. Hernan, C. Fuller, Y. Lee, M. Kocak, C. Lau, M. Chintagumpala, D. Ashley, S. Kellie, R. Gilbertson; St Jude Children's Rsrch Hosp, Memphis, TN; Texas Children's Hospital, Houston, TX; Royal Children's Hospital, Melbourne, Australia; Children's Hospital at Westmead, Sydney, Australia . 2003 ASCO Annual Meeting ,420. |
| 202 | Circulating endothelial cells as a surrogate marker of antiangiogenic activity in patients treated with endostatin. J. Heymach, M. H. Kulke, C. S. Fuchs, J. Force, S. Connors, C. Sidor, D. George, J. P. Eder, J. Folkman, S. Soker; Dana-Farber Cancer Institute, Boston, MA; Children's Hospital, Boston, MA; EntreMed, Inc., Rockville, MD . 2003 ASCO Annual Meeting ,979. |
| 203 | Evaluation of mRNA markers for lymph node (LN) micrometastases detection by real time RT-PCR in non small cell lung carcinoma (NSCLC) P. Saintigny, S. Ricci, M. Kambouchner, J.-L. Breau, J.-F. Bernaudin; APHP Avicenne, Bobigny Cedex, France; APHP Tenon, Paris, France; APHP Avicenne, Paris, France; APHP Avicenne, Avicenne, France . 2003 ASCO Annual Meeting ,3405. |
| 204 | Marker expression of monocyte-derived dendritic cells in healthy individuals and cancer patients; correlation to clinical response. C. Matthes, D. Marx, N. Cillien, R. Gorter, D. R. Lorenzen, J. H. Peters, T. Nesselhut; University Goettingen, Goettingen, Germany; Institut for Tumor Therapy, Duderstadt, Germany; University of California, Medical School, San Francisco, CA . 2003 ASCO Annual Meeting ,723. |
| 205 | COX-2 and MMP-9 as prognostic markers for lung adenocarcinoma. N. H. Yamaguchi, R. T. Vollmer, D. N. Rangel-Pestana, L. M. Demarchi, V. A. Alves, C. Michelin, R. Beyruti, F. Vargas-Suso, J. R. Milanesi, V. L. Capelozzi; Lung and Thoracic Division, Clinical Hospital, University of Sao Paulo, Sao Paulo, Brazil; Duke University and VA Medical Centers, Durham, NC; Lung and Thoracic Division, Clinical Hospital University of Sao Paulo, Sao Paulo, Brazil . 2003 ASCO Annual Meeting ,2844. |
| 206 | Drug resistance and sensitivity markers of Chinese prostate cancer patients J. M. Liu, J. S. Wang, C. C. Pan, C. Lan, C. L. Lee, Y. H. Chang, C. T. Lin; DCR, NHRI, Taipei, Taiwan; Taipei VGH, Taipei, Taiwan . 2003 ASCO Annual Meeting ,1789. |
| 207 | Troponin I as an early marker of late systolic myocardial dysfunction in breast cancer patients submitted to adjuvant chemotherapy with FEC regimen. F. Moura Silva, C. Tosello, L. W. F. Ramos, M. T. Laloni, F. M. Correa, M. T. Pardo, F. Medina, J. C. G. S. Goes; Brazilian Institute for Cancer Control, Sao Paulo - SP, Brazil . 2003 ASCO Annual Meeting ,122. |
| 208 | Celecoxib modulates cyclooxygenase-2 (COX-2)and markers of proliferation and neoangiogenesis in cervical cancer G. Ferrandina, F. O. Ranelletti, F. Legge, V. Salutari, M. Gessi, A. C. Testa, U. Werner, C. Pozzo, L. Lauriola, G. Scambia; Catholic University of Rome, Rome, Italy; Friedrich Alexander University Erlangen-Nuremberg, Erlangen, Germany . 2003 ASCO Annual Meeting ,1840. |
| 209 | Cyclin D1 as a predictive marker in patients with esophageal squamous cell carcinoma and neoadjuvant radiochemotherapy B. L. Brucher, K. Specht, H. J. Stein, J. R. Siewert, H. H枚fler; Depts of Pathology and Surgery, Klinikum Rechts Der Isar, Munich, Germany; GSF National Research Centre for Environment and Health, Munich, Germany . 2003 ASCO Annual Meeting ,1162. |
| 210 | Molecular markers in metastatic gastrointestinal neuroendocrine tumors. T. J. Hobday, J. Rubin, R. Goldberg, C. Erlichman, R. Lloyd; Mayo Clinic, Rochester, MN . 2003 ASCO Annual Meeting ,1078. |
| 211 | The role of bone turnover markers in predicting clinical events in metastatic bone disease J. Brown, R. Cook, R. E. Coleman, P. P. Major, A. Lipton, M. Zheng, Y. Hei, J. Seaman; Cancer Research Centre, University of Sheffield, Sheffield, UK; University of Waterloo, Waterloo, Ontario, Canada; Academic Unit of Oncology, Weston Park Hospital, Sheffield, UK; McMaster University and Hamilton Regional Cancer Centre, Hamilton, Ontario, Canada; Milton S. Hershey Medical Center, Hershey, PA; Novartis Pharmaceutical Corporation, East Hanover, NJ . 2003 ASCO Annual Meeting ,2969. |
| 212 | TIMP-2 and E-cadherin as prognostic markers in colon cancer M. G. Pallotta, F. Roca, J. M. Lastiri, L. Puriccelli, M. S. Varela, E. Bal de Kier Joffe; Hospital Italiano de Buenos Aires, Buenos Aires, Argentina; Instituto Angel Roffo, Buenos Aires, Argentina . 2003 ASCO Annual Meeting ,1393. |
| 213 | The use of predicting factors and surrogate markers in patients' cancer biopsies treated with targeted antibodies to erbB receptors and erbB tyrosine kinase inhibitors. S. S. Bacus, P. J. Beresford, Y. Yarden, N. Spector, B. Smith; Quantitative Diagnostics Lab, Westmont, IL; Quantitative Diagnostics Laboratory, Inc., Westmont, IL; The Weizmann Institute of Science, Rehovot, Israel; GlaxoSmithKline (GSK), Research Triangle Park, NC; Cell Signaling Technology, Beverly, MA . 2003 ASCO Annual Meeting ,3408. |
| 214 | Microvessels density (MVD) as a marker of neoangiogenesis: Prognostic significance in correlation to grading and stage in adult soft tissue sarcomas (STS) of the extremities. A perspective study. A. Comandone, A. Boglione, E. Berardengo, A. Bernardi, G. Gino, E. Brach Del Prever, P. Bergnolo, O. Dal Canton, S. Chiado' Cutin, C. Oliva, for the Italian Sarcoma Group; Ospedale Gradenigo, Torino, Italy; Ospedale San Giovanni Antica Sede, Torino, Italy; Ospedale CTO, Torino, Italy . 2003 ASCO Annual Meeting ,3303. |
| 215 | Pretreatment serum markers and survival in metastatic breast cancer patients treated with letrozole vs. tamoxifen. S. M. Ali, K. Leitzel, L. Demers, H. A. Harvey, H. A. Chaudri-Ross, R. Lang, W. Hackl, W. Carney, P. Hamer, A. Lipton; Penn State Hershey Medical Center/Lebanon VA Medical Center, Hershey/Lebanon, PA; Penn State Hershey Medical Center, Hershey, PA; Novartis Pharma AG, Basel, Switzerland; Oncogene Science/Bayer Diagnostics, Cambridge, MA . 2003 ASCO Annual Meeting ,3413. |
| 216 | Cross-sectional analysis of markers of osteoblast and osteoclast activity in men with advanced prostate cancer D. Michaelson, R. R. Marujo, M. R. Smith; Massachusetts General Hospital, Boston, MA . 2003 ASCO Annual Meeting ,1521. |
| 217 | Role of molecular markers in the clinical outcome and prediction of response to treatment in squamous anal cancer. S. Mawdsley, H. M. Meadows, P. Royston; Mt Vernon Hosp, Middlesex, UK; Cancer Research UK &amp; UCL Cancer Trials Centre, London, UK; MRC Clinical Trials Unit, London, UK . 2003 ASCO Annual Meeting ,3566. |
| 218 | Biological markers and pathological features in early breast cancer (EBC): A comparison between very young women (&lt;35 years-old) and older patients (&gt; 50 years-old). N. Nascimento, F. F. Ferreira Filho, O. Obando, S. Lago, M. Motta, F. Furtado, L. Hermann, R. Rosa, Z. Zetler; Hospital Santa Rita - Santa Casa de Misericordia de Porto Alegre, Porto Alegre, Brazil; Oncology Department, Porto Alegre, Brazil; Pathology Department, Porto Alegre, Brazil . 2003 ASCO Annual Meeting ,3608. |
| 219 | Cytokines as local lung cancer related biopathological markers in bronchoalveolar lavage fluid M. Crohns, S. Saarelainen, M. Erhola, H. Alho, P. Kellokumpu-Lehtinen; Tampere University Hospital, Tampere, Finland; Lammi-Tuulos Health Care Center, Lammi, Finland; National Public Health Institute, Helsinki, Finland . 2003 ASCO Annual Meeting ,3613. |
| 220 | Postoperative follow-up of tumor markers once a month is beneficial to the colorectal cancer patients with pharmacokinetic modulating chemotherapy treatment M. Gega; Hyogo College of Medicine, Nishinomiya, Japan . 2003 ASCO Annual Meeting ,1323. |
| 221 | Stage-specific expression of lymphangiogenic markers in prostate cancer V. Kaushal, P. Mukunyadzi, P. Mehta, M. Kohli; UAMS, Little Rock, AR; UAMS and CAVHS, Little Rock, AR . 2003 ASCO Annual Meeting ,3496. |
| 222 | An attempt to identify biological markers predicting response to primary chemotherapy with epirubicin/ cyclophosphamid in locally advanced breast cancer U. Euler, A. H. Tulusan; Womens Hospital Bayreuth, Bayreuth, Germany . 2003 ASCO Annual Meeting ,3569. |
| 223 | DNA methylation profiling of patients (pts) with myelodysplastic syndromes (MDS) identified methylation of RIL as an independent marker of poor prognosis G. Garcia Manero, Y. Boumber, R. Mannari, J. Vilaythong, E. Youssef, H. Yang, E. Estey, S. Strom, H. Kantarjian, J.-P. Issa; University of Texas M.D. Anderson Cancer Center, Houston, TX . 2003 ASCO Annual Meeting ,2323. |
| 224 | MDM-2 gene amplification in operable non-small cell lung cancer (NSCLC): A strong and independent marker of adverse prognosis J. Jassem, D. Dworakowska, E. Jassem, B. Peters, R. Dziadziuszko, J. Jakobkiewicz, G. Kobierska, W. Rzyman, A. Roessner, R. Schneider-Stock; Medical University of Gdansk, Poland, Gdansk, Poland; Otto von Guericke University, Magdeburg, Germany; Medical University of Gdansk, Gdansk, Poland; University of Gdansk, Gdansk, Poland; Otto von Guericke University, Magfeburg, Germany . 2003 ASCO Annual Meeting ,2639. |
| 225 | Genomic amplification of 10p by CGH analysis: A unique marker for thymic tumors. G. H. Vance, D. Heiber, J. Henley, P. J. Loehrer; Indiana Univ School of Medcn, Indianapolis, IN . 2003 ASCO Annual Meeting ,3467. |
| 226 | Virulizin, a novel immunotherapeutic agent stimulates natural killer (NK) cell function, potential biologic marker of clinical response A. Young, J. De La Garza, C. Du, N. P. Feng, V. Lee, H. Jin, M. Wang, Y. Lee, J. Wright; Lorus Therapeutics Inc., Toronto, Canada; National Cancer Institute, Mexico, Mexico . 2003 ASCO Annual Meeting ,751. |
| 227 | p53, EGFR, and PTEN as markers of diagnosis and prognosis in patients with anaplastic glioma enrolled in NCCTG clinical trials J. C. Buckner, B. W. Scheithauer, J. R. O'Fallon, P. Schaefer, C. Giannini, K. Ballman, R. Jenkins; Mayo Clinic, Rochester, MN; Toledo Community CCOP, Toledo, OH . 2003 ASCO Annual Meeting ,393. |
| 228 | Virtual in-situ hybridization (VISH) for HER-2 as a surrogate marker for predicting response to trastuzumab in patients with breast carcinoma. G. Francis, S. R. Stein; QML Pathology, Southport, Australia; Prognostic Pathology Research Facility, Brisbane, Australia . 2003 ASCO Annual Meeting ,3521. |
| 229 | Immunohistochemical evaluation of anti-cytokeratin and MUC-1 antibodies as potential tumor associated markers for circulating epithelial cells. T. G. Fraizer, I. M. Peterson, D. Chianese, M. C. Miller, G. V. Doyle, L. W. Terstappen, D. F. Hayes; Bryn Mawr Hospital, Bryn Mawr, PA; Immunicon Corporation, Huntingdon Valley, PA; University of Michigan Comprehensive Cancer Center, Ann Arbor, MI . 2003 ASCO Annual Meeting ,3421. |
| 230 | Thymidilate synthase (TS) and dihydropyrimidine dehydrogenase (DPD) expression in inoperable gastric cancer: A predictive marker of benefit to decide the regimen S. Kawabe, H. Takiuchi, M. Gotoh, S. Ohta, K.-I. Katsu; Osaka Medcl College, Takatsuki, Japan . 2003 ASCO Annual Meeting ,1436. |
| 231 | Analysis of the strength of association of multiple post-treatment time-dependent serum markers with survival in patients with castrate metastatic prostate cancer M. D. Galsky, H. I. Scher, A. Hummer, G. Heller, K. Regan, W. K. Kelly; Memorial Sloan-Kettering Cancer Center, New York, NY . 2003 ASCO Annual Meeting ,1589. |
| 232 | Patterns of expression of the ErbB receptors family and their downstream effectors, as markers predicting the efficacy of single-agent trastuzumab (H) in HER-2 amplified metastatic breast cancer (MBC). R. Giuliani, V. Durbecq, D. Larsimont, M. Borms, A. Vindevoghel, G. Jerusalem, V. D'Hondt, M. Pestrin, M. J. Piccart, A. A. Di Leo; Jules Bordet Institute, Brussels, Belgium; AZ Groeninge, Kortrijk, Belgium; Ste Elisabeth Hospital, Namur, Belgium; CHU Sart Tilman, Liege, Belgium; Cliniques Universitaires Saint Luc, Universite Catholique de Louvain, Brussels, Belgium . 2003 ASCO Annual Meeting ,145. |
| 233 | A phase II study of neoadjuvant doxorubicin and docetaxel with molecular markers in women with operable and locally advanced breast cancer. A. Kallab, R. Dalton, R. Vartanian, K. Yeh, T. Wang, A. May, A. Jillella; Medcl College of Georgia, Augusta, GA . 2003 ASCO Annual Meeting ,246. |
| 234 | A dose finding study of an oral antiangiogenesis inhibitor, NM-3 - safety profile and effects on surrogate markers with once-daily dosing. P. Soulie, E. Gamelin, J. P. Eder, J. Bonneterre, D. P. Ryan, J. W. Clark, L. J. Appleman, P. Herait, S. Marsh, A. Jekunen; Centre Paul Papin, Angers, France; Div. of Med. Oncology, Dana-Farber Cancer Inst. and Dept. of Medicine, Brigham and Womens Hospital, Dana-Farber/Harvard Cancer Center, Harvard Medical School, Boston, MA; Centre Oscar Lambert, Lille, France; Div. of Hematology-Oncology, Dept. of Medicine, Massachusetts General Hospital, Dana-Farber/Harvard Cancer Center, Harvard Medical School, Boston, MA; Dana-Farber Cancer Inst, Boston, MA; Cvitkovic &amp; Associes, Bicentre, France; ILEX Oncology Inc., San Antonio, TX . 2003 ASCO Annual Meeting ,777. |
| 235 | A phase II trial of STI571 in patients with metastatic breast cancer (MBC). S. Modi, A. Seidman, M. Dickler, M. Moasser, G. D'Andrea, M. E. Moynahan, K. S. Panageas, L. Tan, L. Norton, C. Hudis; Memorial Sloan-Kettering Cancer Center, New York, NY . 2003 ASCO Annual Meeting ,68. |
| 236 | An adjuvant phase II pilot trial of mutant ras peptide vaccine in stage II and III pancreatic and Dukes C and D colorectal cancer J. Hamilton, R. J. Behrens, M. Achtar, V. Herrin, S. Bernstein, T. Brent-Steele, M. Yancey, C. Maruffi, S. Khleif; GlaxoSmithKline, Philadelphia, PA; National Cancer Institute, Bethesda, MD . 2003 ASCO Annual Meeting ,745. |
| 237 | Genomic profile of vascular permeable regions of patients with glioblastoma multiforme for identification of blood markers for disease S. Guccione, Y. Yang, R. Homer, S. Chang, G. Harsh, S. Atlas, M. Bednarski; Stanford University, Stanford, CA . 2003 ASCO Annual Meeting ,3453. |
| 238 | Telomerase RNA expression as a prognostic marker in patients with favorable histology Wilms tumor. . 2004 ASCO Annual Meeting ,8519. |
| 239 | Preliminary evidence of relationship between genetic markers and oncology patient quality of life (QOL). . 2004 ASCO Annual Meeting ,5. |
| 240 | Thymidylate synthase activity as a significant prognostic marker in patients with renal cell carcinoma. . 2004 ASCO Annual Meeting ,4630. |
| 241 | Cyclin D1 repression as a marker of erlotinib response in aerodigestive tract cancers. . 2004 ASCO Annual Meeting ,3093. |
| 242 | BRCA1 status, molecular markers, clinical variables in breast cancer patients with high probability of having an inherited genetic mutation. . 2004 ASCO Annual Meeting ,9648. |
| 243 | Osteopontin (OPN): A novel tumor marker of potential utility in metastatic breast cancer. . 2004 ASCO Annual Meeting ,575. |
| 244 | Hypoxia-related markers in the plasma of patients with advanced non-small cell lung cancer (NSCLC) and survival from chemotherapy: Southwest Oncology Group (SWOG) S0003. . 2004 ASCO Annual Meeting ,7146. |
| 245 | Urine PGE-M as a marker of intratumoral cyclooxygenase-2 activity in non-small cell lung cancer (NSCLC). . 2004 ASCO Annual Meeting ,9527. |
| 246 | Procalcitonin (PCT) as a diagnostic and prognostic marker in patients with solid tumors and febrile neutropenia. . 2004 ASCO Annual Meeting ,8037. |
| 247 | Tumor budding as a useful prognostic marker in esophageal squamous cell carcinoma. . 2004 ASCO Annual Meeting ,4199. |
| 248 | Validation of markers for the intraoperative detection of metastasis in breast sentinel lymph nodes. . 2004 ASCO Annual Meeting ,9547. |
| 249 | First experience with a new implantable gold marker for extracranial high precision radiotherapy. . 2004 ASCO Annual Meeting ,5144. |
| 250 | Chfr methylation as a marker of taxane sensitivity in breast cancer. . 2004 ASCO Annual Meeting ,9583. |
| 251 | Nuclear beta-catenin as a marker of outcome in localized prostate cancer. . 2004 ASCO Annual Meeting ,9570. |
| 252 | FASs/FASLs ratio: A marker of chemoresistance in advanced colorectal cancer (CRC). . 2004 ASCO Annual Meeting ,3583. |
| 253 | Biological markers associated with response to gefitinib (ZD1839) in patients with breast cancer. . 2004 ASCO Annual Meeting ,648. |
| 254 | Cln101 and Ovr110 as novel serum markers for detection of ovarian cancer. . 2004 ASCO Annual Meeting ,5033. |
| 255 | MCM2: Test of utility as a molecular marker in advanced-stage oropharyngeal carcinoma. . 2004 ASCO Annual Meeting ,5590. |
| 256 | Molecular prognostic markers in oropharyngeal squamous cell carcinoma; the role of C-met. . 2004 ASCO Annual Meeting ,5517. |
| 257 | A novel DNA marker associated with breast metastasis. . 2004 ASCO Annual Meeting ,681. |
| 258 | Neuroendocrine markers as predictor of octreotide acetate therapy in patients with hormone-refractory prostate cancer. . 2004 ASCO Annual Meeting ,4750. |
| 259 | Immunohistochemical tumor markers do not predict response to neoadjuvant doxorubicin and docetaxel in breast cancer. . 2004 ASCO Annual Meeting ,794. |
| 260 | Assessment of multiple markers for association with response rate (RR) and failure-free survival (FFS) in patients with advanced colorectal cancer (CRC) treated with chemotherapy in the MRC CR08 (FOCUS) randomized trial. . 2004 ASCO Annual Meeting ,9506. |
| 261 | Molecular markers of the mTOR pathway activation in human tumors: A baseline analysis. . 2004 ASCO Annual Meeting ,9524. |
| 262 | Serum markers of angiogenesis in patients with von Hippel-Lindau Disease. . 2004 ASCO Annual Meeting ,1033. |
| 263 | Markers of recurrence and predictors of clinical behavior of granulosa cell ovarian tumors. . 2004 ASCO Annual Meeting ,5114. |
| 264 | ERCC1, RRM1 and XPD mRNA expression as a predictive marker of outcome in non-small-cell lung cancer (NSCLC) patients after platinum/gemcitabine induction chemotherapy followed by surgery. . 2004 ASCO Annual Meeting ,7142. |
| 265 | NK-92 cells transduced with retroviral vectors encoding marker genes and class I MHC suppression genes for improved use in adoptive cellular immunotherapy. . 2004 ASCO Annual Meeting ,2592. |
| 266 | Correlation of selected molecular markers to in vitro chemo sensitivity of node-negative primary human breast cancer. . 2004 ASCO Annual Meeting ,9667. |
| 267 | High incidence of new translocations in B-CLL detected by CD40L-enhanced cytogenetics (CEC): A new prognostic marker for infavorable survival outcome in patients with B-CLL. . 2004 ASCO Annual Meeting ,6561. |
| 268 | Intensive post-operative follow-up of breast cancer patients with tumour markers: Accuracy of serum MCA-CA15.3 and CEA-TPA-CA15.3 tumour marker panels for early detection of relapse. . 2004 ASCO Annual Meeting ,753. |
| 269 | Evaluation of epidermal growth factor receptor (EGFR) as a predictive marker in patients with non-small-cell lung cancer (NSCLC) receiving first-line gefitinib combined with platinum-based chemotherapy. . 2004 ASCO Annual Meeting ,7013. |
| 270 | The prognostic importance of the CA19-9 tumour marker and its use as a biochemical indicator of treatment response during (chemo-) radiotherapy of locally advanced pancreatic cancer. . 2004 ASCO Annual Meeting ,4177. |
| 271 | Evaluation of chromosomal aneusomy in bronchial epithelium of smokers as a marker for lung cancer risk. . 2004 ASCO Annual Meeting ,7184. |
| 272 | Prospective Trial for clinical evaluation of TRACP 5b as serum marker of bone resorption and for monitoring an oral clodronat-medication in osteolytic metastases of breast carcinoma. . 2004 ASCO Annual Meeting ,862. |
| 273 | Male breast cancer in Parma Province: Descriptive epidemiology, molecular markers and clinical variables. . 2004 ASCO Annual Meeting ,9655. |
| 274 | Mammaglobin expression in leukapheresis products is a predictive marker of poor prognosis in women with high-risk breast cancer. . 2004 ASCO Annual Meeting ,9596. |
| 275 | A double blind, parallel, placebo controlled, dose ranging study on efficacy and safety of oral clodronate in cancer induced osteolysis measured by biochemical markers of bone resorption. . 2004 ASCO Annual Meeting ,8056. |
| 276 | Severe neutropenia in cycle one of CHOP is a marker of early death among intermediate and high grade lymphoma (IHL) patients. . 2004 ASCO Annual Meeting ,6717. |
| 277 | Cyclin E1 (CCNE1) and E2 (CCNE2) as prognostic and predictive markers for endocrine therapy (ET) in early breast cancer. . 2004 ASCO Annual Meeting ,9504. |
| 278 | A randomised trial of carboplatin versus carboplatin and thalidomide in ovarian cancer, with evaluation of potential surrogate markers of angiogenesis. . 2004 ASCO Annual Meeting ,5024. |
| 279 | Tissue microarrays (TMAS) in colorectal cancer: Study of clinical and molecular markers. . 2004 ASCO Annual Meeting ,9665. |
| 280 | Neoadjuvant paclitaxel/radiation for Stage II/III breast cancer with correlative molecular markers. . 2004 ASCO Annual Meeting ,9622. |
| 281 | Dynamic contrast-enhanced MRI for assessing the disease activity of multiple myeloma: A comparative study with histology, proangiogenic cytokines and clinical markers. . 2004 ASCO Annual Meeting ,6719. |
| 282 | Male breast cancer: Clinico-pathological study with evaluation of biologic markers. . 2004 ASCO Annual Meeting ,777. |
| 283 | Intensive post-operative follow-up of breast cancer patients with tumour markers: Accuracy of serum MCA-CA15.3 and CEA-TPA-CA15.3 tumour marker panels for early detection of relapse. . 2004 ASCO Annual Meeting ,753. |
| 284 | Gene expressions of VEGF and Survivin as molecular markers of lymph node involvement in patients with locally advanced rectal cancer. . 2004 ASCO Annual Meeting ,9526. |
| 285 | Evaluation of biological activity of PCK3145 in metastatic hormone resistant prostatic cancer (HRPC) using serum markers. . 2004 ASCO Annual Meeting ,4692. |
| 286 | Effects of steroidal and nonsteroidal aromatase inhibitors (AIs) on markers of bone turnover and lipid metabolism in healthy volunteers. . 2004 ASCO Annual Meeting ,8038. |
| 287 | Comparison of p53 antibody status and carcinoembryonic antigen (CEA) levels and their clinical impact as diagnostic markers in colorectal cancer patients. . 2004 ASCO Annual Meeting ,9725. |
| 288 | A phase II randomized trial of cisplatin/paclitaxel plus lonidamine versus cisplatin/ifosfamide plus lonidamine in the treatment of ovarian cancer. . 2004 ASCO Annual Meeting ,9678. |
| 289 | NT-pro-BNP and CA 125 as potential markers of mortality during long-term immunetherapy with trastuzumab in HER-2-positive metastatic breast cancer. . 2004 ASCO Annual Meeting ,804. |
| 290 | Effects of atrasentan on disease progression and biological markers in men with metastatic hormone-refractory prostate cancer: Phase 3 study. . 2004 ASCO Annual Meeting ,4508. |
| 291 | The role of biological molecular markers in predicting both response to treatment and clinical outcome in squamous cell carcinoma of the anus. S. Mawdsley, Anal Cancer Trial Management Group, H. Meadows, R. James . 2004 Gastrointestinal Cancers Symposium ,183. |
| 292 | Mononucleotide microsatellite markers are the better markers for microsatellite instability detection. S. Huang, B. Anderson, S. Chen, W. Sun, C. Strom, R. Bender . 2004 Gastrointestinal Cancers Symposium ,278. |
| 293 | A novel method of pancreatic cancer detection by simultaneous analysis of multiple serum markers. S. Winikoff, A. Lokshin, D. Landsittel, K. L. Lee, A. J. Moser, W. H. Schraut, M. T. Lotze, D. L. Bartlett, H. J. Zeh . 2004 Gastrointestinal Cancers Symposium ,166. |
| 294 | Correlation of molecular markers including mutations with clinical outcomes in advanced non small cell lung cancer (NSCLC) patients (pts) treated with gefitinib, chemotherapy or chemotherapy and gefitinib in IDEAL and INTACT clinical trials T. J. Lynch, D. Bell, D. Haber, D. Johnson, G. Giaccone, M. Fukuoka, M. Kris, R. Herbst, A. Krebs, J. Ochs . 2005 ASCO Annual Meeting ,7006. |
| 295 | Circulating tumor cells - not serum tumor markers - predict survival in metastatic breast cancer A. Stopeck, M. Cristofanilli, G. T. Budd, M. J. Ellis, J. Matera, M. C. Miller, G. V. Doyle, W. J. Allard, L. W. Terstappen, D. F. Hayes . 2005 ASCO Annual Meeting ,9516. |
| 296 | Relevant prognostic markers involved in apoptosis in colorectal cancer M. Gasser, M. Bueter, M. Grimm, S. Hom, M. Koenigshausen, A. Thalheimer, S. Mueller, A. Thiede, H. Gassel, D. Meyer, A. Waaga-Gasser . 2005 ASCO Annual Meeting ,9662. |
| 297 | Correlation of pre-treatment apoptotic marker expression with response to neo-adjuvant chemotherapy in locally advanced breast cancer V. Garimella, M. Watson, D. Manton, A. Hubbard, A. Chaturvedi, A. Maraveyas, J. Greenman, P. J. Drew, L. W. Turnbull, M. J. Lind, L. Cawkwell . 2005 ASCO Annual Meeting ,701. |
| 298 | HER2 gene amplification is a marker for global genomic instability R. E. Ellsworth, D. L. Ellsworth, B. Deyarmin, L. R. Hoffman, J. A. Hooke, C. D. Shriver . 2005 ASCO Annual Meeting ,9571. |
| 299 | Prognostic value of clinical and molecular markers in advanced ovarian cancer (AOC): importance of residual (Rs) disease. Translational study using tumor specimens from EORTC 55931/NCIC OV10 Phase III Randomized Clinical Trial (RCT). J. A. Green, F. Duffaud, C. Coens, S. Laframboise, I. Vergote, E. Eisenhauer, M. Bacon, W. Chapman, M. E. van der Burg, A. M. Oza . 2005 ASCO Annual Meeting ,5005. |
| 300 | Efficacy and correlation with predictive markers of gefitinib in pretreated Chinese patients with advanced NSCLC X. T. Zhang, L. Y. Li, X. Chang, X. Mu, S. Wang, M. Wang, W. Zhong, L. Zhang . 2005 ASCO Annual Meeting ,7240. |
| 301 | Recursive partitioning analysis of selected molecular markers to predict mediastinal lymph node involvement in operable non-small cell lung cancer (NSCLC) H. S. Bozcuk, A. Gumus, G. Ozbilim, A. Sarper, M. Ozdogan, M. Artac, M. Samur, B. Savas . 2005 ASCO Annual Meeting ,7320. |
| 302 | Tumor marker expression is predictive of survival in patient with esophageal squamous cell cancer treated with definitive chemoradiotherapy T. Kii, H. Takiuchi, S. Kawabe, M. Gotoh, S. Ohta, K. Katsu . 2005 ASCO Annual Meeting ,4080. |
| 303 | SERUM PROTEINS MARKER FOR EARLY DETECTION OF OVARIAN CANCER G. Mor, I. Visintin, H. Zhao, P. Schwartz, T. Rutherford, L. Yui, P. Bray-Ward, D. C. Ward . 2005 ASCO Annual Meeting ,9508. |
| 304 | Circadian rhythm of bone turnover markers in breast cancer patients with bone metastases and in control subjects. D. G. Generali, S. Tedoldi, M. Tampellini, A. Berruti, M. Torta, S. Bonardi, A. Bottini, M. Tucci, M. Milani, A. Angeli, L. Dogliotti . 2005 ASCO Annual Meeting ,737. |
| 305 | Monitoring tumor markers in serial sera predicts disease failure in lung cancer patients following surgery D. Harpole, M. M. Joshi, R. P. Petersen, C. C. Miller, D. Conlon, K. R. Brooks, T. A. D'Amico . 2005 ASCO Annual Meeting ,7040. |
| 306 | Modeling oncology surrogate markers K. Johnson, B. Stokes, D. Anthony, R. Ward, N. Freemantle, S. Hill . 2005 ASCO Annual Meeting ,9639. |
| 307 | Surrogate markers of activity of AG-013736, a multi-target tyrosine kinase receptor inhibitor, in metastatic renal cell cancer (RCC). O. Rixe, J. Meric, J. Bloch, A. Gentile, R. Mouawad, V. Adam, D. Buthiau, D. Khayat . 2005 ASCO Annual Meeting ,3003. |
| 308 | Responses and molecular markers in patients with peripheral T-cell lymphoma treated on a phase II trial of depsipeptide, FK228. R. L. Piekarz, R. Frye, M. Turner, J. Wright, J. Leonard, S. Allen, S. Smith, M. Kischbaum, J. Zain, S. E. Bates, for all collaborators . 2005 ASCO Annual Meeting ,3061. |
| 309 | Identification of a predictive gene expression marker for recurrence in childhood ependymoma using microarray analysis. N. K. Foreman, J. Straessle, M. Handler, A. Donson . 2005 ASCO Annual Meeting ,1531. |
| 310 | Expression and prognostic significance of tissue hypoxia markers in head and neck squamous cell carcinomas (HNSCC) A. Koong, C. Kong, S. Zhao, D. Richardson, K. O'Byrne, H. Pinto, N. C. Denko, A. J. Giaccia, Q. Le . 2005 ASCO Annual Meeting ,5559. |
| 311 | The tumor metabolic marker Tumor M2-PK in stool: a new biomarker for colorectal cancer H. U. Kloer, P. D. Hardt, P. Schlierbach, M. Toepler . 2005 ASCO Annual Meeting ,3598. |
| 312 | Multiple marker analysis in breast tumors with long term follow-up using Tissue Microarray: Immunohistochemical evaluation of ER, PgR, MIB1, p27, p63, HMWCK, FITH, Her2/neu and p53. D. Di Vizio, A. Sboner, F. de Michelis, R. Micciolo, A. Lucenti, A. Ferro, R. Triolo, E. Galligioni, P. Dalla Palma, M. Barbareschi . 2005 ASCO Annual Meeting ,686. |
| 313 | Identification of Pharmacogenomic Markers for Predicting Sensitivity to BMS-354825, a SRC/ABL Kinase Inhibitor E. Clark, K. A. Reeves, X. Han, P. M. Shaw, C. Fairchild, Q. Wu, S. Platero, T. W. Wong, F. Lee, F. Huang . 2005 ASCO Annual Meeting ,3010. |
| 314 | Overexpression and Prevalence of Molecular Markers in Patients with Cancer of Unknown Primary (CUP) A. Rashid, K. R. Hess, R. Lenzi, M. N. Raber, J. L. Abbruzzese, G. R. Varadhachary . 2005 ASCO Annual Meeting ,9683. |
| 315 | Molecular prognostic markers in oropharyngeal squamous cell carcinoma: the role of phospho-Akt Z. Yu, P. M. Weinberger, C. T. Sasaki, B. Haffty, R. Camp, D. Rimm, B. Burtness, A. Psyrri . 2005 ASCO Annual Meeting ,5517. |
| 316 | Markers of bone metabolism predict survival in hormone refractory prostate cancer (HRPC): results from a randomized California Cancer Consortium &amp; Univ of Chicago trial P. N. Lara, J. Longmate, W. Stadler, M. van Loan, J. Wexler, D. I. Quinn, P. Twardowski, E. E. Vokes, D. R. Gandara . 2005 ASCO Annual Meeting ,4569. |
| 317 | Molecular markers in extragonadal germ cell tumors (EGCTs): a matched-case control study. M. Nikolaou, C. Valavanis, I. Lekka, G. Aravantinos, G. Fountzilas, D. Skarlos, H. Kalofonos, V. Kostopoulou, P. Arapantoni Dadioti, D. Pectasides . 2005 ASCO Annual Meeting ,9700. |
| 318 | Immunological Markers of Outcome of Non-small Cell Lung Cancer D. A. Potter, C. Amin, A. Mohiuddin, S. Badve, D. Davidson, K. Kesler, N. Hanna, H. Edenberg, L. Einhorn, R. Mitra . 2005 ASCO Annual Meeting ,7232. |
| 319 | Candidate molecular markers for oral precancer and cancer R. Ralhan, N. Chakravarti, J. Kaur, S. Soni, A. Kumar, S. D. Gupta, S. Bahadur, N. K. Shukla, S. V. Deo . 2005 ASCO Annual Meeting ,9598. |
| 320 | Prognostic value of tumor markers CA 15-3 and CEA during fulvestrant treatment R. Bartsch, G. Steger, C. Wenzel, U. Pluschnig, D. Hussian, U. Sevelda, R. Mader, C. C. Zielinski . 2005 ASCO Annual Meeting ,730. |
| 321 | Suppression of bone turnover markers by zoledronic acid and correlation with clinical outcome A. Lipton, Y. Hei, R. Coleman, P. Major, R. Cook . 2005 ASCO Annual Meeting ,532. |
| 322 | Do Molecular markers predict when to implement aromatase inhibitor therapy in invasive breast cancer. S. Tovey, B. Dunne, C. J. Witton, A. Forsyth, T. G. Cooke, J. M. Bartlett . 2005 ASCO Annual Meeting ,9559. |
| 323 | Phase II Study 2000mg of intravesical Gemcitabine in marker lesions F. M. Calais Da Silva, F. E. Calais . 2005 ASCO Annual Meeting ,4593. |
| 324 | Urine di-acetyl sperimine (DiAcSpm) as a new cancer screening marker for colorectal cancer K. Takahashi, K. Hiramatsu, T. Yamaguchi, H. Matsumoto, H. Miyamoto, S. Saji, T. Mori, M. Kawakita, Tokyo Metropolitan Institute of Medical Science, Kogakuin University . 2005 ASCO Annual Meeting ,3731. |
| 325 | Baseline Proliferation Markers and Cytopathologic Findings in Breast Epithelium of Women at Increased Risk for Breast Cancer N. Sneige, Y. Gong, J. Lammey, V. Valero, G. Babiera, H. Kuerer, G. Hortobagyi, B. Arun . 2005 ASCO Annual Meeting ,9694. |
| 326 | Immunohistochemical markers as prognostic indicators for efficacy of imatinib therapy in GIST E. Stepanova, A. Meshcheryakov, O. Anurova, D. Shubina, M. Lichinitser . 2005 ASCO Annual Meeting ,9703. |
| 327 | Evaluation of serum YKL-40 as a prognostic marker for overall survival in patients with metastatic malignant melanoma H. Schmidt, J. S. Johansen, J. Gehl, P. Geertsen, K. Fode, H. von der Maase . 2005 ASCO Annual Meeting ,7520. |
| 328 | Basal sFAS/sFASL ratio and FAS polymorphisms, as a prognostic marker, in advanced colorectal carcinoma patients (ACRC), treated with oxaliplatin-based chemotherapy. P. Gascon . 2005 ASCO Annual Meeting ,3631. |
| 329 | Chemokine receptor CXCR4 mRNA expression may be a marker of the presence of compromised lymph nodes in oral cavity squamous cell carcinoma C. M. Campofiorito, F. R. Mangone, F. S. Pasini, S. Maistro, I. M. Snitcovsky, F. Walder, C. Lehn, M. B. Carvalho, M. M. Brentani, M. H. Federico . 2005 ASCO Annual Meeting ,9622. |
| 330 | Biochemical markers of bone turnover in the diagnosis of myeloma bone disease O. Dizdar, I. Barista, U. Kalyoncu, O. Karadag, I. Celik, A. Kars, G. Tekuzman . 2005 ASCO Annual Meeting ,6600. |
| 331 | A prospective blinded study of microsatellite instability (MSI) as a marker of overall survival (OS) in the adjuvant treatment of colorectal cancer (CRC) patients S. Popat, H. Pan, Y. Shao, D. Zhao, Z. Chen, R. S. Houlston . 2005 ASCO Annual Meeting ,9544. |
| 332 | Early hypertension (HTN) as a potential pharmacodynamic (PD) marker for survival in pancreatic cancer (PC) patients (pts) treated with bevacizumab (B) and gemcitabine (G) G. Friberg, K. Kasza, E. E. Vokes, H. L. Kindler . 2005 ASCO Annual Meeting ,3020. |
| 333 | Specific up-regulation of FAK expression in primary breast cancer as a marker for malignant transformation E. Stickeler, D. O. Watermann, B. Gabriel, M. J盲ger, G. Gitsch . 2005 ASCO Annual Meeting ,9647. |
| 334 | A Novel Mode of Antitumor Activity For Imatinib Mesylate: Consequences for the design of surrogate markers of efficacy and combination therapies. L. Zitvogel, F. Ghiringhelli, M. Terme, C. Borg, N. Chaput, C. M茅nard, A. Lecesne, J. Fletcher, M. C. Heinrich, T. Tursz, J. Taieb . 2005 ASCO Annual Meeting ,2516. |
| 335 | Plasma levels of 5-hydroxyindole-3-acetic acid (5HIAA) as a pharmacodynamic marker of blood flow changes induced by the vascular targeting agent (VTA) 5,6 dimethyl xanthenone acetic acid, DMXAA L. R. Kelland, B. C. Baguley, L. Zhao, Q. Ding, P. Kestell, M. Ravic, M. B. Jameson, M. J. McKeage . 2005 ASCO Annual Meeting ,3123. |
| 336 | Mutational analysis of K-RAS and EGFR implicates K-RAS as a resistance marker in the Southwest Oncology Group (SWOG) trial S0126 of bronchioalveolar carcinoma (BAC) patients (pts) treated with gefitinib. P. H. Gumerlock, W. S. Holland, H. Chen, W. A. Franklin, F. R. Hirsch, P. C. Mack, A. M. Davies, J. McCoy, H. J. West, D. R. Gandara . 2005 ASCO Annual Meeting ,7008. |
| 337 | Exploratory analyses EGFR, kRAS mutations and other molecular markers in tumors of NSCLC patients (pts) treated with chemotherapy +/- erlotinib (TALENT) U. Gatzemeier, A. Heller, D. Foernzler, J. Moecks, C. Ward, F. de Rosa, G. Sauter, U. Brennscheidt . 2005 ASCO Annual Meeting ,7028. |
| 338 | Efficacy and influence of neoadjuvant Epiriubicin/Vinorelbine chemotherapy (EN) on molecular markers in inflammatory breast cancer (IBC) and preliminary results of a randomized study testing as adjuvant chemotherapy the same EN-combination versus docetaxel (TXT). M. Brandi, A. Cataldi, G. Di Corato, R. Altieri, M. Digennaro, S. Longo, G. Ranieri, F. Schittulli, S. Petroni . 2005 ASCO Annual Meeting ,886. |
| 339 | A retrospective analysis of clinical outcomes in patients treated for malignant mixed mesodermal tumors of the ovary and uterus with an evaluation of molecular marker expression D. R. Crotzer, J. K. Wolf, R. R. Broaddus, L. M. Ramondetta . 2005 ASCO Annual Meeting ,5107. |
| 340 | Serum vascular endothelial growth factor(VEGF) and VEGF-C levels as tumor markers in patients with cervical cancer A. Mitsuhashi, K. Suzuka, K. Yamazawa, H. Matsui, K. Seki, S. Sekiya . 2005 ASCO Annual Meeting ,5075. |
| 341 | Molecular Markers of Metastases in Advanced Stage Adenocarcinoma of the Prostate. A. Potti, G. Chen, N. Shukeir, S. A. Rabbani, I. Sehgal . 2005 ASCO Annual Meeting ,9672. |
| 342 | Recurrences of colorectal cancer: time distribution and diagnostic sensitivity of serum CEA, TPA, CA19.9, CA72.4 tumour markers. A. Nicolini, P. Ferrari, L. Anselmi, M. Metelli, A. Carpi, R. Spisni, M. Conte, P. Berti, P. Miccoli . 2005 ASCO Annual Meeting ,3723. |
| 343 | Effect of oral ibandronate versus intravenous (i.v.) zoledronic acid on markers of bone resorption in patients with breast cancer and bone metastases: Results from a comparative phase III trial J. Body, M. Lichinitser, S. Tjulandin, M. Budde, B. Bergstr枚m . 2005 ASCO Annual Meeting ,534. |
| 344 | The use of ErbB/HER activation status as prognostic markers in breast cancer patients treated with trastuzumas A. Mukherjee . 2005 ASCO Annual Meeting ,553. |
| 345 | Activation State (Phosphorylated) EGFR and STAT3 as Prognostic Markers in Resected Non-Small Cell Lung Cancer (NSCLC) T. Cortas, R. Eisenberg, P. Fu, J. Robke, J. A. Kern, A. Dowlati . 2005 ASCO Annual Meeting ,7090. |
| 346 | Levels of circulating endothelial cells (CECs) and monocytes as pharmacodynamic markers of SU11248 activity in patients (pts) with metastatic imatinib-resistant GIST A. Norden-Zfoni, J. Manola, J. Desai, J. Morgan, C. L. Bello, S. E. Deprimo, D. R. Shalinsky, C. Baum, G. D. Demetri, J. Heymach . 2005 ASCO Annual Meeting ,9036. |
| 347 | High Dose Interferon-a2b (HDI): Toxicity, Response, and Predictive Markers in a Neoadjuvant trial for Regional Lymph Node Metastatic Melanoma S. J. Moschos, H. D. Edington, U. N. Rao, D. Jukic, J. Shipe-Spotloe, S. R. Land, S. Agarwala, J. M. Kirkwood . 2005 ASCO Annual Meeting ,7517. |
| 348 | The clinical significance of the follow up of tumor markers in breast carcinoma J. Finek, L. Holubec, O. Topolcan, V. Treska, E. Helmichova . 2005 ASCO Annual Meeting ,865. |
| 349 | Role of CEA, PLUNC and CK19 mRNA expression in lymph nodes from resected stage I non-small cell lung cancer (NSCLC) patients (p) as markers of occult micrometastasis. A pilot study S. Benlloch, J. Galbis, F. M. Peiro, C. Alenda, J. M. Rodriguez-Paniagua, J. Sanchez-Paya, S. Romero, J. L. Marti-Ciriquian, B. Massuti . 2005 ASCO Annual Meeting ,9654. |
| 350 | Multiplex genotyping and gene expression assays for colorectal cancer treatment selection markers S. Y. Kwok, W. Kim, S. Tom, C. Christopherson, D. Wolfson, T. Toombs, S. Broder, J. Sninsky . 2005 ASCO Annual Meeting ,3651. |
| 351 | Gene expression profiles of breast cancer obtained from core cut biopsies before neoadjuvant docetaxel, doxorubicin and cyclophoshamide chemotherapy correlate with routine prognostic markers and could be used to identify predictive signatures. A. Rody, T. Karn, M. Munnes, G. von Minckwitz, S. Loibl, C. Solbach, U. Holtrich, M. Kaufmann . 2005 ASCO Annual Meeting ,646. |
| 352 | Ixabepilone (BMS-247550) plus trastuzumab combination chemotherapy induces synergistic antitumor efficacy in HER2 dependent breast cancers and is accompanied by modulation of molecular response markers F. Y. Lee, S. Castaneda, I. Inigo, D. Kan, B. Paul, M. Wen, C. Fairchild, E. Clark, H. Lee . 2005 ASCO Annual Meeting ,561. |
| 353 | Evaluating angiogenic cytokines VEGF, basic FGF and TGF- 脽1 as tumor markers in ascites I. L. Nascimento, H. S. Barbosa, R. E. Schaer, D. C. Lemaire, S. M. Freire, V. Vale, M. L. Salomao, G. S. Lessa, R. J. Meyer . 2005 ASCO Annual Meeting ,9627. |
| 354 | The Effect of Doxycycline on Bone Turnover and Tumor Markers in Breast Cancer (BC) Patients with Skeletal Metastases S. K. Dhesy-Thind, J. Julian, R. Tozer, P. Ellis, A. Arnold, G. Singh, M. Levine . 2005 ASCO Annual Meeting ,3198. |
| 355 | Molecular discrimination of benign and malignant esophageal tissues using multi-marker real-time RT-PCR M. Mitas, K. Mikhitarian, J. S. Almeida, W. E. Gillanders, D. N. Lewin, L. Hoover, A. Graham, R. Hawes, C. E. Reed, B. J. Hoffman . 2005 Gastrointestinal Cancers Symposium ,15. |
| 356 | Quantitative multiplex TaqMan assays for colorectal cancer treatment selection markers S. Kwok, S. Tom, W. Kim, T. Toombs, C. Christopherson . 2005 Gastrointestinal Cancers Symposium ,179. |
| 357 | MASP-2 and TIMP-1 protein levels as selection markers for adjuvant therapy in colon cancer. H. J. Nielsen, H. Ytting, I. J. Christensen, J. C. Jensenius, S. Thiel, N. Br眉nner . 2005 Gastrointestinal Cancers Symposium ,286. |
| 358 | Elevated soluble p55TNF-Receptor in colon cancer patients as markers of impending relapse and aggressive tumor behavior: paradoxically the p75TNF-R heralds cure. D. Aderka, T. Eshed, M. Inbar . 2005 Gastrointestinal Cancers Symposium ,287. |
| 359 | Lymphovascular invasion (LVI) is a marker for reduced efficacy of post-operative radiotherapy (P-XRT) after radical prostatectomy (RP) J. P. Brooks, P. S. Albert, J. J. O'Connell, T. F. Donahue, D. G. McLeod, M. M. Poggi . 2005 Prostate Cancer Symposium ,176. |
| 360 | Use of gold markers for setup in fractionated HDR brachytherapy and organ tracking during IMRT in combined radiotherapy of prostate cancer D. M. Aebersold, D. Vetterli, S. Thalmann, C. Kolotas, I. Bernhard, L. Kemmerling, F. Behrensmeier, R. Mini, G. Thalmann, R. Greiner . 2005 Prostate Cancer Symposium ,138. |
| 361 | Intensity modulated radiation therapy (IMRT) using implanted fiducial markers with daily portal imaging: assessment of prostate organ motion R. J. Lee, C. Chu, W. T. Sause . 2005 Prostate Cancer Symposium ,102. |
| 362 | Targeting the Prostate with External Beam Radiotherapy: Prostate Volume Changes and their Relationship to Implantable Markers C. C. Pan, H. M. Sandler, L. L. Levine, M. E. Ray, J. T. Wei, J. M. Balter . 2005 Prostate Cancer Symposium ,92. |
| 363 | Transcription factor NFkB a potential molecular marker for predicting and improving treatment efficacy in esophageal cancer. J. G. Izzo, T. Wu, U. Malhotra, J. Ensor, R. Luthra, C. K. Chao, S. G. Swisher, Z. Liao, B. B. Aggarwal, W. N. Hittelman, J. A. Ajani . 2006 ASCO Annual Meeting ,10065. |
| 364 | Principal factor analysis of predictive markers of response and resistance to primary chemo-endocrine treatment in elderly breast cancer (BC) patients. D. G. Generali, S. Fox, F. M. Buffa, A. Berruti, M. Brizzi, G. Allevi, S. Bonardi, L. Dogliotti, A. Bottini, A. Harris . 2006 ASCO Annual Meeting ,618. |
| 365 | Identification of integrin as potential molecular markers in human glioblastoma multiforme: Microarray analysis guided by contrast-enhanced MRI. Y. Yang, Y. Chia, D. Rubin, G. Harsh, S. Atlas, M. Bednarski, S. Guccione . 2006 ASCO Annual Meeting ,10007. |
| 366 | Phenotypic expression of Bax is a predictive marker of 5-fluorouracil treatment in colorectal cancer. U. Manne, C. Suarez-Cuervo, N. C. Jhala, J. Posey, C. B. Herring, S. Meleth, W. E. Grizzle . 2006 ASCO Annual Meeting ,3605. |
| 367 | Identification of predictive markers of clinical activity from a phase II trial of single agent pertuzumab (rhuMab 2C4), a HER dimerization inhibitor, in advanced ovarian cancer (OC). L. Amler, M. S. Gordon, A. Strauss, N. Rabbee, M. K. Derynck, K. Krueger, D. A. Eberhard, D. Matei, B. Y. Karlan . 2006 ASCO Annual Meeting ,3001. |
| 368 | A. Knoop, H. Knudsen, E. Balslev, B. B. Rasmussen, J. Overgaard, M. During, K. V. Nielsen, J. T. J酶rgensen, H. Mouridsen, B. Ejlertsen . 2006 ASCO Annual Meeting ,532. |
| 369 | Association of molecular markers with toxicity outcomes in a randomized trial of chemotherapy for advanced colorectal cancer (FOCUS). M. T. Seymour, M. S. Braun, S. D. Richman, C. Daly, L. C. Thompson, A. Meade, M. Parmar, J. M. Allan, P. Selby, P. Quirke, and the FOCUS Trial Investigators . 2006 ASCO Annual Meeting ,2022. |
| 370 | Serum carboxyterminal telopeptide (ICTP) as a prognostic marker in bone metastases (BM) treated with bisphosphonates. L. Costa, R. Carvalho, A. G. Oliveira, M. Bicho, A. Fernandes, T. Rodrigues, S. Ali, K. Leitzel, L. Demers, A. Lipton . 2006 ASCO Annual Meeting ,10107. |
| 371 | D. G. Mollevi, T. Serrano, M. M. Ginesta, J. Valls, J. Torras, E. Ramos, J. Germ脿, V. Moreno, G. Capella, J. Figueras, A. Villanueva . 2006 ASCO Annual Meeting ,10092. |
| 372 | Clinical pathologic status and expression of tumor markers in 5,000 Chinese breast cancers. J. Zhang, R. Hui, P. Liu, Y. Yu, Y. Liu, X. Hao . 2006 ASCO Annual Meeting ,10644. |
| 373 | A phase II report of longitudinal bone marker time trends in racially diverse prostate cancer patients receiving zoledronic acid with androgen deprivation. V. R. Phooshkooru, H. Spencer, M. Kohli . 2006 ASCO Annual Meeting ,14568. |
| 374 | NCOA3: A molecular prognostic marker for melanoma. J. Rangel, S. Torabian, L. Shaikh, M. Nosrati, J. R. Miller, R. W. Sagebiel, M. Kashani-Sabet . 2006 ASCO Annual Meeting ,8007. |
| 375 | Predictive markers for the treatment of colorectal cancer with cetuximab. E. Razis, E. Briasoulis, I. Kostopoulos, M. Bobos, C. Christodoulou, D. Papamichael, S. K. Rigatos, P. Papakostas, P. Kosmidis, G. Fountzilas . 2006 ASCO Annual Meeting ,13500. |
| 376 | Impact of prognostic markers on outcomes in patients with advanced chronic lymphocytic leukemia treated with the regimen of fludarabine/rituximab plus oblimersen (Bcl-2 Antisense). B. Mavromatis, K. Rai, P. K. Wallace, C. Soho, B. Landrigan, P. Meyn, T. Wei, K. K. Chan, A. Chanan-Khan . 2006 ASCO Annual Meeting ,6609. |
| 377 | Circulating chromogranin-a (CgA) as a useful marker in the diagnosis and follow up of neuroendocrine tumors (nets): An Italian multicenter observational study (cromAnet). L. Dogliotti*, F. De Braud掳, G. Delle Fave搂, E. Degli Uberti#, P. Tomassetti掳掳, M. Gion**, A. Leon**, M. Torta*, L. Borgna$, (CromAnet italian study group) . 2006 ASCO Annual Meeting ,14091. |
| 378 | Circulating molecular markers in predicting radiation-induced lung damage. L. Zhao, F. Kong, J. Hayman, G. Kalemkarian, D. Arenberg, S. Lysons, J. Curtis, K. Cease, D. Brenner, T. Lawrence . 2006 ASCO Annual Meeting ,7144. |
| 379 | Bone turnover markers in postmenopausal breast cancer patients treated with fulvestrant. A. Agrawal, R. A. Hannon, K. L. Cheung, R. Eastell, J. F. Robertson . 2006 ASCO Annual Meeting ,680. |
| 380 | Targeting tumour markers: What makes therapy discriminating? K. Johnson, R. Ward . 2006 ASCO Annual Meeting ,13129. |
| 381 | Utility of clinical markers to identify patients with high risk cervical dysplasia and cancer. S. Ueda, M. Carney, J. Killeen, M. Wakabayashi . 2006 ASCO Annual Meeting ,10055. |
| 382 | MBD4 and SEI1 as new prognostic markers in colorectal cancer patients receiving adjuvant 5-Fluorouracil. J. Ju, A. Formentini, G. Nakajima, M. Kornmann, Y. Xi . 2006 ASCO Annual Meeting ,20085. |
| 383 | Endothelial progenitor marker CD133 mRNA expression in peripheral blood mononuclear cells predicts outcome of cancer patients. N. Mehra, M. Penning, J. Maas, N. Van Daal, E. Voest . 2006 ASCO Annual Meeting ,10087. |
| 384 | Circulating tumour markers and time to progression in the chemotherapy treatment of metastatic breast cancer. M. Ruiz-Lopez-tejada, L. Tejedor-Cabrera, C. Iradi-Martinez . 2006 ASCO Annual Meeting ,10674. |
| 385 | HER2 comparison with novel 3-gene marker sets for risk prediction of distant recurrence of breast carcinoma. J. Alsobrook, P. Hraber, L. Davis, C. Harris, P. Doherty, B. Hall, T. Williams, J. Hozier . 2006 ASCO Annual Meeting ,20009. |
| 386 | Glycodelin: A possible new biological marker in colorectal cancer. R. Govindarajan, S. Parthasarathy . 2006 ASCO Annual Meeting ,20081. |
| 387 | Molecular markers for lymph node involvement in locally advanced cervical carcinomas. G. B. Kristensen, H. Lyng, D. H. Svendsrud, R. Holm, K. Knutstad, R. Br酶vig, K. Sundfoer, H. Oksefjell, T. Stokke . 2006 ASCO Annual Meeting ,5049. |
| 388 | Evaluation of mRNA markers for micrometastases detection by RT-PCR in lymph nodes (LN), peripheral blood (PB) and bone marrow (BM) of breast cancer patients (pts). A. M. Morelle, A. Frasson, F. Zerwes, S. Alves, G. Devenz, C. M. Silveira, A. V. Nectoux, M. Wagner, B. Garicochea . 2006 ASCO Annual Meeting ,20068. |
| 389 | The value of multi-marker reverse transcriptase-polymerase chain reaction (MM RT-PCR) assay in lymphatic drainage (LY) and peripheral blood (BL) for molecular staging of melanoma patients (pts) after lymph node dissection (LND). W. Ruka, Z. I. Nowecki, P. Rutkowski, J. Kulik, E. Lorenc, A. Nasierowska-Guttmejer, Z. Zurawski, M. Zdzienicki, J. A. Siedlecki . 2006 ASCO Annual Meeting ,8053. |
| 390 | Gene and surface-antigen expression profilings concordantly identify alpha4-integrin/CD49d as a marker for unmutated (UM) bad prognosis B-cell chronic lymphocytic leukemia (B-CLL). V. Gattei, D. Benedetti, D. Marconi, M. Dal Bo, A. Zucchetto, G. Del Poeta, A. Steffan, R. Bomben, R. Campanini, M. Degan . 2006 ASCO Annual Meeting ,10076. |
| 391 | Changes in tumor biological markers during primary systemic chemotherapy (PST). E. Solomayer, H. Neubauer, V. Beck, J. Huober, U. Vogel, T. Fehm . 2006 ASCO Annual Meeting ,20035. |
| 392 | Quantitative real time-PCR of CD133 mRNA: A potential surrogate angiogenic marker of response for patients with metastatic sarcoma. E. H. Lin, Y. Li, J. Trent, S. Patel, M. A. Burgess, D. M. Steinert, R. S. Benjamin, D. Li, L. L. Chen . 2006 ASCO Annual Meeting ,20034. |
| 393 | Prognostic significance of immunohistochemical markers in non-small cell lung cancer. D. Renouf, R. Wood-Baker, D. Ionescu, S. Leung, H. Massoudi, B. Gilks, J. Laskin . 2006 ASCO Annual Meeting ,7211. |
| 394 | Elevated preoperative serum level of tumor markers CEA and CA 15-3 in breast cancer patients. B. Park, H. Kim, J. Oh, S. Kim, K. Kim, J. Sohn . 2006 ASCO Annual Meeting ,621. |
| 395 | Role of early serial change in serum carcinoembryonic antigen levels as a predictive marker for gefitinib sensitivity in Japanese patients with non-small cell lung cancer. K. Hotta, K. Kiura, M. Tabata, N. Takigawa, Y. Fujiwara, S. Umemura, S. Hosokawa, A. Hisamoto, H. Ueoka, M. Tanimoto . 2006 ASCO Annual Meeting ,17029. |
| 396 | An evaluation of changes in biological markers (ER, PgR, or Ki67) after neoadjuvant chemotherapy (ET: epirubicin and docetaxel) in operable breast cancer. R. Tashima, R. Nishimura . 2006 ASCO Annual Meeting ,10613. |
| 397 | Prognostic value of baseline inflammatory markers in inoperable non-small cell lung cancer (NSCLC). N. MacDonald, G. Kasymjanova, S. Dobson, H. Kreisman, V. Cohen, J. S. Agulnik, D. Small . 2006 ASCO Annual Meeting ,17035. |
| 398 | CD8 P. Sharma, E. Sato, D. Bajorin, Y. Shen, S. Wen, V. Reuter, A. Jungbluth, S. Gnjatic, L. Old . 2006 ASCO Annual Meeting ,4544. |
| 399 | Serum vascular endothelial growth factor as a significant marker of treatment response in Hodgkin disease. M. Weyl Ben Arush, A. Ben Barak, P. Shenzer, S. Maurice, E. Livne . 2006 ASCO Annual Meeting ,9033. |
| 400 | Identification of urinary markers using proteomics analysis of urine in patients with bladder cancer. G. Nabi, P. Cash, J. N'Dow . 2006 ASCO Annual Meeting ,14605. |
| 401 | High expression of Tenascin-C extra domains, markers of angiogenesis, in human lung cancer. S. Hillinger, M. Silacci, S. Arni, S. Brack, W. Weder, D. Neri . 2006 ASCO Annual Meeting ,20051. |
| 402 | Identification of serum markers of glioblastoma multiforme patients using image-guided genomic and proteomic analysis. S. Guccione, Y. Yang, Y. Chia, D. Rubin, Y. Wang, G. Harsh, S. Atlas, M. Bednarski . 2006 ASCO Annual Meeting ,20008. |
| 403 | Identification of ABCA5 as a tissue and urine diagnostic marker for prostatic intraepithelial epithelial or PIN. M. E. Stearns, Y. Hu, M. Wang, K. Veverka . 2006 ASCO Annual Meeting ,10089. |
| 404 | Serial RT-PCR detection of circulating tumour cells as a marker of disease progression in patients with malignant melanoma. P. A. Ascierto, M. Budroni, A. Cossu, S. Scala, E. Simeone, P. Giuliano, S. M. Satriano, C. Carac貌, M. Casula, G. Palmieri . 2006 ASCO Annual Meeting ,18009. |
| 405 | Tumor markers in metastatic breast cancer: High tumor specificity within the reference range. P. Stieber, D. Nagel, V. Heinemann . 2006 ASCO Annual Meeting ,10554. |
| 406 | Pharmacogenomic analysis for individual response to CPT-11 in colorectal cancer: Prediction formula of tumor response using novel marker genes and genotypes associated with the toxicity. H. Narahara, N. Sugimoto, ICSG (Individualized Chemotherapy Study Group), N. Tomita, K. Murata, M. Fukunaga, M. Ohue, T. Sakaeda, H. Ishida, K. Tanimoto, M. Nishiyama . 2006 ASCO Annual Meeting ,2060. |
| 407 | EGFR mutation and intron 1 CA repeat polymorphism as predictive markers of gefitinib responsiveness in non-small cell lung cancer (NSCLC). S. W. Han, T. Y. Kim, K. W. Lee, D. Y. Oh, S. H. Lee, D. W. Kim, D. H. Chung, S. A. Im, D. S. Heo, Y. J. Bang . 2006 ASCO Annual Meeting ,7173. |
| 408 | A prospective study of the effects of once weekly bortezomib on markers of bone metabolism in patients with multiple myeloma (MM). S. Peles, N. M. Fisher, F. Gao, M. H. Tomasson, J. F. Dipersio, R. Vij . 2006 ASCO Annual Meeting ,7548. |
| 409 | ECOG 3503: A pilot study to determine if downstream markers of EGFR linked signaling pathways predict response to erlotinib (OSI-774) in the first-line treatment of patients with advanced non-small cell lung cancer (NSCLC). J. Kolesar, J. Brahmer, S. Li, P. Guaglianone, J. Patel, M. Keppen, M. Hidalgo, D. Carbone, J. Siegfried, J. Schiller . 2006 ASCO Annual Meeting ,7162. |
| 410 | Efficacy of zoledronic acid versus placebo on biochemical markers of bone metabolism in patients with breast cancer metastatic to bone. N. Kohno, K. Aogi, H. Minami, S. Takashima . 2006 ASCO Annual Meeting ,10559. |
| 411 | Comparison and analysis among expression of CRP and tumor markers in advanced non-small cell lung cancer patients. W. Dong, X. Ren, P. Liu . 2006 ASCO Annual Meeting ,17126. |
| 412 | Levels of circulating endothelial cells (CECs) as early pharmacodynamic markers of activity of anti-angiogenic (AA) therapy in advanced pancreatic cancer (PC). R. V. Iyer, M. M. Javle, A. Pande, J. Yu, D. Wilkinson, Y. Rustum, P. Wallace . 2006 ASCO Annual Meeting ,14080. |
| 413 | Differential circulation kinetics during antiangiogenic therapy of four distinct blood cell populations expressing endothelial markers. D. G. Duda, K. S. Cohen, E. Di Tomaso, D. T. Scadden, C. G. Willett, R. K. Jain . 2006 ASCO Annual Meeting ,3038. |
| 414 | Nuclear magnetic resonance spectroscopy of expressed prostatic secretions: Metabolite citrate and derivatives are potential markers of prostate cancer. D. Crawford, N. Serkova, E. Gamito, R. Jones, C. O'Donnell, T. Hedlund . 2006 ASCO Annual Meeting ,4623. |
| 415 | Thymidylate synthase polymorphisms and its mRNA expression levels as independent chemo-predictive markers in esophageal adenocarcinoma patients receiving 5-fluorouracil chemotherapy. H. Kuramochi, K. Tanaka, B. J. Lehman, C. M. Dunst, D. S. Oh, S. R. Demeester, J. A. Hagen, K. D. Danenberg, T. R. Demeester, P. V. Danenberg . 2006 ASCO Annual Meeting ,4063. |
| 416 | Enhanced diagnosis in suggested malignant pleural effusion using combined modality of genetic and biochemical tumor markers. J. Chang . 2006 ASCO Annual Meeting ,17137. |
| 417 | Tissue inhibitor of metalloproteinases 1 (TIMP-1) and tumor type M2 pyruvate kinase (TuM2-PK) were compared with established markers in advanced colorectal cancer. S. Siepmann, D. Pollmann, R. Geppert, K. D. Wernecke, K. Possinger, D. Lueftner . 2006 ASCO Annual Meeting ,13537. |
| 418 | Markers of folate metabolism in pancreatic cancer: Thymidylate synthase, thymidine phosphorylase, and MTHFR: Results from the RTOG 9704 prospective randomized adjuvant treatment trial J. J. Farrell, K. Winter, M. Van Rijnsoever, W. Regine, R. Abrams, H. Safran, J. MacDonald, A. Bowen Benson, C. Willett, H. Elsaleh . 2006 Gastrointestinal Cancers Symposium ,144. |
| 419 | Analysis of multiple serum markers for the early detection of colon cancer: DcR3 (DD-C248), Spondin-2 (DD-P108), Reg IV (DD-C101), and TRAIL-R R. A. Fan, K. L. Krall, N. W. Kim, I. Simon, M. J. Sarno, L. A. Boardman, R. L. Wolfert . 2006 Gastrointestinal Cancers Symposium ,321. |
| 420 | Predictive factors and molecular markers of postoperative prognosis for patients with intrahepatic cholangiocarcinoma Y. Yamashita, A. Taketomi, H. Kayashima, D. Kitagawa, Y. Kuroda, N. Harimoto, E. Tsujita, S. Aishima, K. Shirabe, Y. Maehara . 2006 Gastrointestinal Cancers Symposium ,181. |
| 421 | Over-expression of RIa subunit (RIas) of protein kinase A may be a marker for favorable pancreatic cancer: An analysis of patients treated on Radiation Therapy Oncology Group (RTOG) 98-12 A. A. Konski, K. Winter, L. Khor, T. Al-Saleem, R. Abrams, W. Small, T. Rich, C. Willett . 2006 Gastrointestinal Cancers Symposium ,148. |
| 422 | Promoter methylation profiling identifies GATA-4 as a sensitive and specific marker for detection of colorectal cancer M. Lentjes, S. van den Bosch, Y. Akiyama, M. Weijenberg, S. Baylin, J. Herman, W. van Criekinge, A. de Bruine, M. van Engeland, t. dept. of Gastroenterology and Hepatology, Universi . 2006 Gastrointestinal Cancers Symposium ,283. |
| 423 | Pathologic fractures and bone markers are predictors of clinical outcome in patients with prostate cancer and bone metastases. F. Saad, R. E. Coleman, R. Cook, J. E. Brown, M. R. Smith, Y. Hei, Y. Chen . 2006 Prostate Cancer Symposium ,150. |
| 424 | Method comparison analysis of ultrasound- and fiducial marker-based prostate localization. C. D. Fuller, T. Scarbrough, A. Wong, J. Ting, N. Golden, P. Kupelian, C. R. Thomas . 2006 Prostate Cancer Symposium ,81. |
| 425 | Bone turnover markers for the early detection of bone metastases in patients with prostate cancer. I. J. de Jong, N. Koopmans, A. J. Breeuwsma, R. J. Nijman, E. van der Veer . 2006 Prostate Cancer Symposium ,145. |
| 426 | Intensity modulated radiotherapy using implanted fiducial markers with daily portal imaging: Assessment of prostate organ motion. J. Chen, R. J. Lee, D. Handrahan, W. T. Sause . 2006 Prostate Cancer Symposium ,94. |
| 427 | Beneficial effects on skeletal complications and bone markers from zoledronic acid compared with pamidronate in prostate cancer patients with bone metastases. F. Saad, E. J. Small, M. R. Smith . 2006 Prostate Cancer Symposium ,148. |
| 428 | Epidermal growth factor receptor familiy members (EGFR and HER2) are prognostic markers and potential therapeutic targets in prostate cancer. T. Schlomm, A. Erbersdobler, R. Simon, M. Graefen, G. Sauter, H. Huland . 2006 Prostate Cancer Symposium ,310. |
| 429 | Outcome of radical radiotherapy in clinically localised prostate cancer: A molecular approach to identify the role of markers of tumour hypoxia. R. VERGIS, S. JHAVAR, C. PARKER, C. COOPER . 2006 Prostate Cancer Symposium ,312. |
| 430 | Minimal benefit of an endorectal balloon for prostate immobilization as verified by daily localization of intraprostatic fiducial markers. C. A. Canning, M. Garzotto, A. Y. Hung . 2006 Prostate Cancer Symposium ,76. |
| 431 | Zoledronic acid (ZA) initiated during the first year of androgen deprivation therapy (ADT) increases bone mineral density (BMD) and suppresses bone turnover markers in prostate cancer patients. C. W. Ryan, D. Huo, L. M. Demers, T. M. Beer, L. V. Lacerna . 2006 Prostate Cancer Symposium ,192. |
| 432 | IFCT0401-bio trial: Predictive biological markers for disease control (DC) of patients with non-resectable, adenocarcinoma with bronchioloalveolar carcinoma features (ADC-BAC) treated with gefitinib. m. wislez, M. Antoine, V. Poulot, J. F. Morere, M. Perol, P. J. Souquet, M. P. Lebitasy, G. Zalcman, F. Coulet, J. Cadranel, Intergroupe Francophone de Cancerologie Thoracique(IFCT) . 2007 ASCO Annual Meeting ,7653. |
| 433 | Markers of angiogenesis in cervical cancer: A Gynecologic Oncology Group study. L. Randall-Whitis, B. J. Monk, E. S. Han, K. Darcy, R. A. Burger, S. Liao, W. A. Peters, R. J. Stock, J. P. Fruehauf . 2007 ASCO Annual Meeting ,5536. |
| 434 | Validation of genomic markers that predict distant recurrence risk via FISH assays in women with early stage breast carcinoma. T. M. Williams, G. T. Budd, M. Gunter, R. Hines, S. Masood, P. Pawloski, L. Schwartzberg, F. Wei, R. R. Tubbs . 2007 ASCO Annual Meeting ,10506. |
| 435 | Molecular markers expression in mediastinal nodes from resected stage I non-small cell lung cancer (NSCLC): Pronostic impact and potential role as markers of occult micrometastasis. B. Massuti, J. Galbis, C. Alenda, F. M. Peir贸, J. Mart铆, B. Baswitch, J. M. Rodriguez-Paniagua, R. Rosell, M. Taron, S. Benlloch . 2007 ASCO Annual Meeting ,21016. |
| 436 | Prognostic value of preoperative serum tumor markers, including CEA, Cyfra21-1, SCC, CA19-9, SCC, CA125, TPA, NSE, and SLX, in patients with completely resected pathological stage I non-small cell lung cancer. H. Matsuguma, H. Suzuki, Y. Ishikawa, T. Kondo, R. Nakahara, Y. Kamiyama, K. Mori, T. Kodama, S. Honjo . 2007 ASCO Annual Meeting ,7681. |
| 437 | Normalization of bone markers and improved survival during zoledronic acid therapy. A. Lipton, R. Cook, R. E. Coleman, P. Major, E. Terpos, J. Body, M. R. Smith, J. Brown, P. Garnero . 2007 ASCO Annual Meeting ,9013. |
| 438 | Stem cell markers in gastrointestinal cancer. M. Valladares-ayerbes, S. D铆az Prado, V. Medina, P. Iglesias, B. Rodr铆guez, M. Haz, M. Reboredo, M. Quind贸s, G. Alonso-Jaudenes, L. Ant贸n Aparicio . 2007 ASCO Annual Meeting ,21095. |
| 439 | Identification of predictive and surrogate molecular markers for dasatinib in prostate cancer: Rationale for patient selection and efficacy monitoring. X. Wang, K. Reeves, R. Luo, S. Wu, L. Xu, M. Ayers, F. Lee, E. Clark, F. Huang . 2007 ASCO Annual Meeting ,3579. |
| 440 | Clinical and pathological prognostic markers for survival in adult patients with post-transplant lymphoproliferative disorders: BCL2 as a prognostic marker. A. B. Oton, W. Hong, M. Melhem, D. George, K. Foon, I. M. Ghobrial . 2007 ASCO Annual Meeting ,8073. |
| 441 | Prognostic impact of angiogenic markers in tumor and stromal cells in non-small cell lung cancer (NSCLC). T. Donnem, S. Al-Saad, K. Al-Shibli, M. P. Delghandi, M. Persson, M. Nilsen, L. T. Busund, R. M. Bremnes . 2007 ASCO Annual Meeting ,10596. |
| 442 | Prognostic and biologic impact of molecular markers in papillary renal cell carcinoma. S. B. Riggs, T. Klatte, D. B. Seligson, A. J. Pantuck, J. T. Leppert, J. S. Lam, N. Zomorodian, F. F. Kabbinavar, A. S. Belldegrun . 2007 ASCO Annual Meeting ,15525. |
| 443 | Heparanase and Survivin--potential markers of aggressiveness of colon cancers. A. C. Hoffmann, C. Hoffmann, K. D. Danenberg, H. J. Lenz, P. V. Danenberg . 2007 ASCO Annual Meeting ,14524. |
| 444 | Association between different potential predictive markers from TRUST, a trial of erlotinib in non-small cell lung cancer (NSCLC). E. Laack, C. Schneider, T. Gutjahr, E. Heinm枚ller, V. Lutz, J. Moecks, K. Rohr, C. Schmidtgen, O. Spleiss, M. Reck . 2007 ASCO Annual Meeting ,7651. |
| 445 | Prognostic serum markers in malignant pleural mesothelioma: Epidermal growth factor and platelet-derived growth factor. P. Betta, R. Libener, S. Orecchia, M. Salvio, F. Schillaci, R. Filiberti, R. Puntoni, M. Paganuzzi, P. Marroni . 2007 ASCO Annual Meeting ,18097. |
| 446 | Coagulation markers D-dimer and prothrombin fragment 1.2 compared to disease and treatment status in prostate cancer patients. B. Y. Wong, L. Fink, J. Symanowski, A. Dincer, D. Adcock, N. Vogelzang . 2007 ASCO Annual Meeting ,15587. |
| 447 | Usefulness of frailty markers in the assessment of the health and functional status in older cancer patient referred for chemotherapy. F. G. Retornaz, J. Monette, M. Monette, N. Sourial, D. Wan-Chow-Wah, G. Batist, H. Bergman . 2007 ASCO Annual Meeting ,19673. |
| 448 | Molecular markers associated with response and clinical outcome to cetuximab/bevacizumab/irinotecan (CBI) versus cetuximab/bevacizumab (CB) in irinotecan-refractory colorectal cancer (BOND2). M. Azuma, D. Yang, M. Carpanu, E. Hollywood, M. Lue-Yat, W. Zhang, K. D. Danenberg, P. V. Danenberg, L. Saltz, H. Lenz . 2007 ASCO Annual Meeting ,4113. |
| 449 | Identification of sensitivity markers for BMS-536924, an inhibitor for insulin-like growth factor-1 receptor. F. Huang, W. Hurlburt, R. Hafezi, X. Han, J. Chen, J. Carboni, R. M. Attar, L. Helman, E. Clark, A. Dongre . 2007 ASCO Annual Meeting ,3506. |
| 450 | Preliminary study of three tumor markers for hepatocellular carcinoma: AFP, AFP-L3, and Glypican-3. H. Li, K. Z. Qu, R. M. Ner, A. D. Sferruzza, R. A. Bender . 2007 ASCO Annual Meeting ,15014. |
| 451 | Identification of predictive markers to differentiate ixabepilone from paclitaxel activity in ER-negative breast cancer patients. S. Wu, S. Chasalow, H. Lee, L. Xu, B. Paul, O. Mokliatchouk, W. F. Symmans, K. E. Zerba, L. Pusztai, E. Clark . 2007 ASCO Annual Meeting ,2525. |
| 452 | Quantification of a panel of prognostic markers gives a novel method for predicting radiosensitivity in head and neck squamous cell carcinoma cell lines. K. B. Roberg, L. Farnebo, L. Norberg-Spaak . 2007 ASCO Annual Meeting ,6082. |
| 453 | Molecular markers to differentiate between pancreatic cancer and normal pancreas/chronic pancreatitis: Gene expression profiling study. M. Jarzab, M. Olakowski, M. Oczko-Wojciechowska, M. Kowalska, K. Fujarewicz, E. Chmielik, D. Lange, A. 艢wierniak, B. Jarz膮b, P. Lampe . 2007 ASCO Annual Meeting ,21150. |
| 454 | Prognostic markers in stage II/III rectal cancer after neoadjuvant 5-fluorouracil-based chemoradiotherapy. C. Pinto, F. Di Fabio, C. Longobardi, C. Ceccarelli, D. Cuicchi, B. Iacopino, G. Ugolini, F. Minni, B. Cola, A. A. Martoni . 2007 ASCO Annual Meeting ,14514. |
| 455 | High BAALC expression associates with other molecular prognostic markers, poor outcome and a distinct gene expression signature in cytogenetically normal acute myeloid leukemia (CN AML): A Cancer and Leukemia Group B (CALGB) study. C. Langer, A. S. Ruppert, M. D. Radmacher, S. P. Whitman, P. Paschka, C. D. Baldus, K. Mr贸zek, J. E. Kolitz, G. Marcucci, C. D. Bloomfield . 2007 ASCO Annual Meeting ,7013. |
| 456 | Serum YKL-40 levels are usuful diagnostic tumor markers for adenocarcinoma of the uterine cervix. A. Mitsuhashi, H. Usui, H. Matsui, S. Tate, Y. Unno, K. Hirashiki, M. Shozu . 2007 ASCO Annual Meeting ,16008. |
| 457 | Prolonged survival in patients with persistently elevated tumor markers after chemotherapy for nonseminomatous germ cell cancer. Z. He, Z. Sun, G. Liu, J. Manola, P. Loehrer . 2007 ASCO Annual Meeting ,5051. |
| 458 | Procollagen-I-propeptide and 尾-crosslaps are prognostic markers for pretherapeutic estimation of treatment success of combined radio- and bisphosphonate therapy in patients with bone metastases--A phase-II study. W. Wagner, A. Radmard, M. Bach, C. Loitsch, M. G. Krukemeyer, G. J. Wiedemann . 2007 ASCO Annual Meeting ,9096. |
| 459 | Lymph node metastasis in breast cancer: The role of tumor markers. M. J. Lund, S. M. Graham, J. M. Liff, R. J. Coates, E. W. Flagg, X. Yuan, M. G. Lin, J. W. Eley, P. L. Porter . 2007 ASCO Annual Meeting ,21019. |
| 460 | Final results of ECOG 3503: A pilot study to determine if downstream markers of EGFR linked signaling pathways predict response to erlotinib (OSI-774) in the first-line treatment of patients with advanced non-small cell lung cancer (NSCLC). J. Kolesar, J. Brahmer, J. Lee, P. Guaglianone, J. Patel, M. Keppen, M. Hidalgo, D. Carbone, J. Schiller . 2007 ASCO Annual Meeting ,7588. |
| 461 | Development of prognostic models in non-small cell lung cancer based on 60 serum markers and clinical variables. S. Holdenrieder, D. Nagel, V. Heinemann, J. von Pawel, H. Raith, K. Feldmann, U. Stenman . 2007 ASCO Annual Meeting ,18079. |
| 462 | A multiplex assay for detection of common pediatric sarcoma tumor markers. R. A. Bender, J. Y. Liu, H. Li, K. Z. Qu, A. D. Sferruzza, H. R. Sanders . 2007 ASCO Annual Meeting ,10041. |
| 463 | The nuclear expressions of ERK1/2 and p38 can be useful markers for tumor invasiveness and lymph node metastasis in stomach cancer. H. Chang, K. Choi, S. Lee, K. Yoon . 2007 ASCO Annual Meeting ,15109. |
| 464 | Predictive value for survival of a risk model of two serological markers, beta-2-microglobulin (B2M) and lactic dehydrogenase (LDH), in diffuse large cell lymphoma (DLCL). M. A. Rodriguez, S. Temple, L. Fayad, F. Hagemeister, P. McLaughlin, J. Romaguera, F. Cabanillas . 2007 ASCO Annual Meeting ,10599. |
| 465 | Intratumoral lactate dehydrogenase 5 (LDH5) protein expression is associated with expression of angiogenesis markers and hypoxia in patients with colorectal cancer (CRC). M. I. Koukourakis, A. Giatromanolaki, E. Sivridis, K. C. Gatter, A. L. Harris, T. Trarbach, G. Folprecht, M. M. Shi, G. Meinhardt . 2007 ASCO Annual Meeting ,4107. |
| 466 | Serum REG4 protein in pancreatic cancer as a tumor marker: A prospective study. A. Sawaki, R. Takayama, N. Mizuno, M. Tajika, N. Hoki, Z. E. Sayed, K. Matsuo, H. Nakagawa, Y. Nakamura, K. Yamao . 2007 ASCO Annual Meeting ,15063. |
| 467 | ERCC1 Codon 118 polymorphism is a useful prognostic marker in patients with pancreatic cancer treated with platinum-based chemotherapy. H. Kamikozuru, H. Kuramochi, K. Hayashi, K. Uchida, M. Yamamoto . 2007 ASCO Annual Meeting ,15012. |
| 468 | Retrospective analysis of P-STAT6 expression as a predictive marker in primary nervous system lymphoma patients assigned to high-dose methotrexate. G. G. Altavilla, V. V. Pitini, C. C. Arrigo, G. G. Marabello, C. C. Naro, C. C. Tomasello, M. M. Righi, D. D. la Torre . 2007 ASCO Annual Meeting ,8059. |
| 469 | Clinical and pathological prognostic markers for survival in adult patients with post-transplant lymphoproliferative disorders: BCL2 as a prognostic marker. A. B. Oton, W. Hong, M. Melhem, D. George, K. Foon, I. M. Ghobrial . 2007 ASCO Annual Meeting ,8073. |
| 470 | Topoisomerase I expression in locally advanced rectal cancer as predictive marker for response to preoperative chemoradiation. K. E. Horisberger, R. D. Hofheinz, B. Muessle, P. Findeisen, A. Hochhaus, S. Post, F. Willeke . 2007 ASCO Annual Meeting ,14566. |
| 471 | Markers of bone turnover (ICTP, NTX) and serum matrix metalloproteinase 1 (MMP1) as prognostic markers in breast cancer patients (BC) with bone metastases (BM) treated with bisphosphonates (BP). L. A. Costa, I. Alho, S. Casimiro, A. G. Oliveira, I. Lu铆s, A. Fernandes, M. Bicho, S. M. Ali, K. Leitzel, L. Demers, A. Lipton . 2008 ASCO Annual Meeting ,1024. |
| 472 | Prognostic markers for stage 3 neuroblastoma (NB): A report from the International Neuroblastoma Risk Group (INRG) project. J. R. Park, W. B. London, J. M. Maris, H. Shimada, Y. Zhang, K. K. Matthay, T. Monclair, P. F. Ambros, S. L. Cohn, A. Pearson . 2008 ASCO Annual Meeting ,10009. |
| 473 | Alterations in estrogen-associated proliferation markers in histologically-normal endometrium of obese women. R. A. Lacour, S. N. Westin, L. A. Meyer, P. T. Soliman, E. R. Keeler, S. G. Boyd-Rogers, D. L. Urbauer, R. R. Broaddus, D. S. Loose, K. H. Lu . 2008 ASCO Annual Meeting ,16503. |
| 474 | Identification of predictive markers of response in colorectal cancer following treatment with dasatinib, an orally active tyrosine kinase inhibitor of Z. A. Wainberg, J. Dering, C. Ginther, A. Anghel, O. Kalous, A. Desai, J. R. Hecht, E. Clark, D. Slamon, R. S. Finn . 2008 ASCO Annual Meeting ,14688. |
| 475 | Biological markers of cisplatin resistance in advanced testicular germ cell tumors (GCT). A. Garcia-Velasco, I. Duran, J. Menendez, S. del Barco, J. Brunet, H. Cortes-Funes, L. G. Paz-Ares . 2008 ASCO Annual Meeting ,16063. |
| 476 | Molecular markers distinguish patients at differential risk of brain metastases in lung cancer by immunohistochemistry. D. N. Hayes, C. B. Lee, M. Hayward, M. A. Socinski, T. E. Stinchcombe, P. Roberts, L. Thorne, P. S. Bernard, X. Yin, A. Parsons, W. K. Funkhouser . 2008 ASCO Annual Meeting ,8083. |
| 477 | Molecular prognostic markers in squamous cell carcinoma of the head and neck: the role of E-cadherin. P. Kountourakis, E. Pectasides, B. A. Burtness, D. Pectasides, A. Psyrri, G. Fountzilas . 2008 ASCO Annual Meeting ,6040. |
| 478 | Serum Immunoglobulin free light chains as markers of disease burden and response to treatment in patients with Waldenstrom's macroglobulinemia. E. Hatjiharissi, B. T. Ciccarelli, L. Ioakimidis, R. Borok, J. D. Soumerai, R. J. Manning, Z. R. Hunter, L. Xu, C. J. Patterson, S. Alvin, S. P. Treon . 2008 ASCO Annual Meeting ,8617. |
| 479 | The predictive value of serum tumor markers for pathologic findings after chemotherapy for primary mediastinal nonseminomatous germ cell tumors. L. E. Kruter, K. A. Kesler, M. Yu, Z. T. Hammoud, K. M. Rieger, L. H. Einhorn . 2008 ASCO Annual Meeting ,5087. |
| 480 | Correlation of blood and physiologic markers with effect of bevacizumab (BV) with chemoradiation therapy in rectal cancer (RC). C. G. Willett, D. G. Duda, L. Xu, E. diTomaso, Y. Boucher, B. Czito, M. Ancukiewicz, J. Clark, M. Carroll, G. Lauwers, R. K. Jain . 2008 ASCO Annual Meeting ,4096. |
| 481 | Relationship between angiogenic markers and clinicopathologic factors/outcome in GOG-170D, a phase II trial of bevacizumab (Bev) in recurrent or persistent epithelial ovarian cancer (EOC) and primary peritoneal cancer (PPC). E. S. Han, R. Burger, K. M. Darcy, M. W. Sill, B. E. Greer, J. I. Sorosky, J. P. Fruehauf . 2008 ASCO Annual Meeting ,5577. |
| 482 | Influence of baseline inflammatory markers on the response to first line chemotherapy and overall survival in advanced NSCLC. G. Kasymjanova, N. MacDonald, J. S. Agulnik, C. Pepe, H. Kreisman, V. Cohen, R. Sharma, B. Gagnon, D. Small . 2008 ASCO Annual Meeting ,8102. |
| 483 | Utility of tumor markers for the early detection of metastatic breast cancer. I. Ertl, V. Heinemann, I. Bauerfeind, M. Untch, D. Laessig, D. Nagel, D. Seidel, P. Stieber . 2008 ASCO Annual Meeting ,1072. |
| 484 | Role of MTHFR polymorphisms as predictive markers of acute toxicity during 5-fluorouracil based chemotherapy for colorectal cancer: Preliminary data. V. Adamo, T. Franchina, B. Adamo, R. Briguglio, E. Restuccia, G. Chiofalo, G. Ferraro, D. Caccamo, R. Ientile . 2008 ASCO Annual Meeting ,15036. |
| 485 | The prognostic value of radiological bio-markers for detection of cerebral glioma grades and early evaluation of tumor response to radiation therapy using MRI perfusion and spectroscopy. M. M. Abdel Wahab, K. M. Maher, M. A. Osman . 2008 ASCO Annual Meeting ,13015. |
| 486 | Bone remodeling markers in breast and prostate cancer patients undergoing endocrine and bisphosponate therapy. F. K. Wenz, G. Welzel, A. Keller, P. Findeisen, M. Neumaier, B. Hermann . 2008 ASCO Annual Meeting ,9602. |
| 487 | Alterations in serum prothrombotic markers induced by treatment with bevacizumab-based chemotherapy regimens. C. Murphy, F. Crotty, E. C. Smyth, G. Healy, O. S. Breathnach, L. Grogan, F. Ni Ainle, P. Murphy . 2008 ASCO Annual Meeting ,15034. |
| 488 | Clinical utility of blood-borne markers of epithelial cell death in pancreatic cancer. S. St. George-Smith, R. A. Smith, W. Greenhalf, N. Smith, P. Ghaneh, J. P. Neoptolemos, C. Dive . 2008 ASCO Annual Meeting ,22056. |
| 489 | Predictive markers of response to neoadjuvant chemotherapy in locally advanced breast cancer (LAPC). A. Mukherjee, M. Shehata, R. Sharma, E. Rakha, I. Ellis, G. Ball, B. M. Ball, S. Chan . 2008 ASCO Annual Meeting ,581. |
| 490 | Markers of a chronic inflammatory state and trial completion: Data from cancer cachexia trial. N. MacDonald, G. Kasymjanova, B. Gagnon, R. Sharma, V. Baracos, W. Droege, H. Kreisman . 2008 ASCO Annual Meeting ,9594. |
| 491 | Utility of four tumor markers, alone and in combination, for detection of hepatocellular carcinoma: AFP, AFP-L3, DCP, and Cystatin C. H. Li, A. Sferruzza, K. Z. Qu, N. H. Afdhal, M. Lai, J. S. Radcliff, R. A. Bender . 2008 ASCO Annual Meeting ,4585. |
| 492 | Surrogate markers of antiangiogenic therapy in patients with locally advanced breast cancer with lymphangitic spread to the chest wall: Results from a phase II randomized study of bevacizumab with sequential versus concurrent oral vinorelbine plus capecitabine. M. Locatelli, G. Curigliano, P. Mancuso, F. Bertolini, G. Peruzzotti, M. Scicchitano, S. Dellapasqua, L. Lunghi, A. Goldhirsch . 2008 ASCO Annual Meeting ,14649. |
| 493 | Predictive utility of molecular markers for efficacy of preoperative gemcitabine and pemetrexed in lung cancer. K. E. Sommers, J. Zhou, A. Canter, X. Li, A. Sharma, L. Robinson, G. Bepler . 2008 ASCO Annual Meeting ,7544. |
| 494 | Clinical significance of biochemical markers in prostate cancer. M. M. Meshref, O. Youssef, D. Elgayar, M. Kamal . 2008 ASCO Annual Meeting ,16041. |
| 495 | Use of a panel of proteomic markers to improve the sensitivity of CA125 for detecting stage I epithelial ovarian cancer. C. H. Clarke, E. T. Fung, C. Yip, C. Joy, D. Badgwell, K. R. Coombes, Z. Zhang, K. H. Lu, R. C. Bast . 2008 ASCO Annual Meeting ,5542. |
| 496 | Common tumor markers predict extent of disease and success of cytoreductive surgery in patients with appendiceal malignancies. V. Gushchin, A. Ross, C. Nieroda, B. Kalesan, P. Kostuik, D. Holter, A. Sardi . 2008 ASCO Annual Meeting ,15527. |
| 497 | High expression of epithelial-mesenchymal transition (EMT) markers in malignant mesothelioma and possible therapeutic intervention using an N-cadherin antagonist. M. B. Suraokar, H. Lin, D. He, N. Llansa, G. Mendoza, D. Woods, L. Prudkin, J. J. Lee, I. I. Wistuba, A. S. Tsao . 2008 ASCO Annual Meeting ,8067. |
| 498 | Transcription factors Stat5a and Stat5b: Favorable prognostic markers in breast cancer. A. Witkiewicz, A. Ryder, L. M. Neilson, F. E. Utama, T. H. Tran, T. Hyslop, H. Rui . 2008 ASCO Annual Meeting ,22071. |
| 499 | Prognostic impact of bone turnover markers in multiple myeloma. O. Dizdar, I. Barista, U. Kalyoncu, O. Karadag, I. Celik, A. Kars, G. Hascelik, A. Cila, A. Pinar, G. Tekuzman . 2008 ASCO Annual Meeting ,8610. |
| 500 | Role of cytokeratin and vimentin as prognostic markers in oral cancers. V. Rao, D. Chaukar, M. Vaidya, s. Sawant, A. Dcruz, S. Kane . 2008 ASCO Annual Meeting ,17030. |
| 501 | Outcome following resection for patients with primary mediastinal nonseminomatous germ cell tumors and rising serum tumor markers post-chemotherapy. S. M. Radaideh, V. Cook, K. A. Kesler, L. H. Einhorn . 2008 ASCO Annual Meeting ,5038. |
| 502 | Genomic alterations identified by array comparative genomic hybridization as prognostic markers in breast cancer recurrence. K. Hwang, W. Han, J. Lee, J. Cho, E. Ko, E. K. Kim, S. Jung, E. Jeong, J. Bae, S. Kim, D. Noh . 2008 ASCO Annual Meeting ,11105. |
| 503 | Correlation of FGF2 tumor expression with tumor response, PFS, and changes in plasma pharmacodynamic (PD) markers following treatment with brivanib alaninate, an oral dual inhibitor of VEGFR and FGFR tyrosine kinases. S. Platero, O. Mokliatchouk, G. C. Jayson, D. J. Jonker, L. S. Rosen, S. Luroe, J. Kelsey, D. Feltquate, L. Velasquez, S. Galbraith . 2008 ASCO Annual Meeting ,3506. |
| 504 | Early epirubicin (EPI)-induced myocardial dysfunction revealed by serial tissue doppler imaging (TDI): Correlation with inflammatory and oxidative stress markers. G. Mantovani, G. Mercuro, M. Dessi', C. Madeddu, R. Serpe, E. Massa, C. Cadeddu, A. Piras . 2008 ASCO Annual Meeting ,20517. |
| 505 | A study to determine whether the subclassification of DLBCL (using immunohistochemical (IHC) markers with tissue microarray (TMA)) is better than international prognostic index (IPI) to predict prognosis and survival. M. F. Jawad, A. M. Noonan, E. Watson, G. Avalos, M. Keane, C. E. Connolly . 2008 ASCO Annual Meeting ,22153. |
| 506 | A longitudinal prospective study of YKL-40 and matrix metalloproteinase-9 (MMP-9) as serum tumor markers in gliomas. F. M. Iwamoto, A. F. Hottinger, S. Karimi, E. Riedel, K. Panageas, B. Gu, J. Rao, M. Fleisher, E. Holland, L. M. DeAngelis, A. Hormigo . 2008 ASCO Annual Meeting ,2038. |
| 507 | Value of CYFRA 21-1,carcinoembryonic antigen, and squamous cell carcinoma antigen as tumor markers in non-small cell lung cancer patients. J. L. Patel, J. A. Erickson, D. G. Grenache, W. L. Roberts . 2008 ASCO Annual Meeting ,19042. |
| 508 | Pemetrexed as second-line treatment for patients with advanced non-small cell lung cancer (NSCLC): Efficacy and correlation with molecular markers. A. Blasco, R. Sirera, J. Terrasa, M. Provencio, C. Guill茅n, I. Maestu, R. de las Pe帽as, I. Bover, M. Berdiel, I. de Aguirre . 2008 ASCO Annual Meeting ,8095. |
| 509 | Circulating endothelial cells and endothelial progenitors as predictive markers of clinical response to bevacizumab-based treatment in advanced colorectal cancer. M. Manzoni, K. Bencardino, M. Ronzoni, B. Rovati, F. Loupakis, S. Mariucci, S. Brugnatelli, E. Villa, A. Falcone, M. Danova . 2008 ASCO Annual Meeting ,14527. |
| 510 | 2008 Molecular Markers . ,. |
| 511 | Immunotherapeutic Markers . ,. |
| 512 | Immunohistochemical markers of cisplatin resistance and prognosis in advanced testicular germ cell tumors (GCT) A. Garcia-Velasco, I. Duran, J. Menendez, S. del Barco, J. Brunet, H. Cort茅s-Funes, L. Paz-Ares . 2008 Molecular Markers ,20. |
| 513 | Use of protein expression profiling to identify markers of radiation sensitivity and resistance in the NCI 60 cell lines J. S. Yordy, L. A. Byers, M. Davies, G. B. Mills, Z. Ju, U. Raju, K. Ang, J. V. Heymach . 2008 Molecular Markers ,70. |
| 514 | Serum antibodies as predictive markers of clinical response to anti-CTLA-4 (Ipimilumab) treatment in advanced melanoma patients S. Gnjatic, J. Yuan, E. Ritter, A. A. Jungbluth, H. Gallardo, S. Terzulli, G. Ritter, A. Houghton, L. J. Old, J. P. Allison, J. D. Wolchok . 2008 Molecular Markers ,21. |
| 515 | Assessment of markers for progressive liver alterations during hepatic tumorigenesis through a NMR-based approach G. Palmieri, D. Paris, D. Melck, G. Corso, R. Calemma, F. Tatangelo, F. Izzo, G. Castello, A. Motta . 2008 Molecular Markers ,121. |
| 516 | Role of bone turnover markers in the diagnosis of bone metastases from breast cancer T. Ibrahim, E. Sacanna, L. Mercatali, E. Flamini, R. Ricci, P. Serra, E. Scarpi, S. Biserni, M. Ricci, D. Amadori . 2008 Molecular Markers ,119. |
| 517 | Gene methylation markers in prostate cancer S. Anderson, L. Kam-Morgan, A. Chatterjee, M. Eisenberg . 2008 Molecular Markers ,115. |
| 518 | Serum prognostic markers in head and neck cancer F. Meyer, 脡. Samson, P. Douville, T. Duchesne, G. Liu, I. Bairati . 2008 Molecular Markers ,27. |
| 519 | Characterization of BRCA1 mutant cancer stem cells reveals heterogeneity of cell markers L. Varticovski, M. H. Wright, A. M. Calcagno, M. Hollingshead, S. V. Ambudkar . 2008 Molecular Markers ,9. |
| 520 | Potential diagnostic markers in bronchial fluid of small cell lung cancer (SCLC). . 2009 ASCO Annual Meeting ,e22221 . |
| 521 | A pilot study of biological markers (BM) as predictors for response and prognosis in patients (pts) with R0 resected gastric cancer (GC). . 2009 ASCO Annual Meeting ,e22076 . |
| 522 | Developing genetic markers for melanoma risk assessment. . 2009 ASCO Annual Meeting ,9046. |
| 523 | Stage-specific prognostic value of molecular markers in colon cancer: Results of the translational study on the PETACC 3-EORTC 40993-SAKK 60-00 trial. . 2009 ASCO Annual Meeting ,4002. |
| 524 | Prognostic significance of preoperative serum tumor markers in the patients with curatively resected advanced gastric cancers. . 2009 ASCO Annual Meeting ,e15515 . |
| 525 | Association between inflammatory markers, symptom burden, hypogonadism, and survival in cancer patients with cachexia. . 2009 ASCO Annual Meeting ,9594. |
| 526 | Identification of potential diagnostic markers in bronchial fluid of patients with non small cell lung cancer (NSCLC). . 2009 ASCO Annual Meeting ,e22216 . |
| 527 | DUSPs as markers of MEK/Erk activation in primary colorectal cancer. . 2009 ASCO Annual Meeting ,4064. |
| 528 | Bone markers in prostate cancer (PC) patients: Biologic criteria to identify patients at risk of developing distant metastases. . 2009 ASCO Annual Meeting ,e16069 . |
| 529 | The evaluation of peritoneal lavage tumor markers for gastric cancer. . 2009 ASCO Annual Meeting ,e15643 . |
| 530 | Identification of baseline predictive markers of sunitinib activity using a human cytokine antibody array in patients with metastatic renal cell carcinoma (MRCC). . 2009 ASCO Annual Meeting ,5113. |
| 531 | The influence of bisphosphonates on bone turnover markers in patients (pts) with breast cancer (BC). . 2009 ASCO Annual Meeting ,1058. |
| 532 | Expression and prognostic significance of differentiation markers in primary CNS lymphoma (PCNSL). . 2009 ASCO Annual Meeting ,e13028 . |
| 533 | Microvesicular-mediated gene transfer of prostate tumor markers. . 2009 ASCO Annual Meeting ,e16076 . |
| 534 | Prognostic significance of immunohistochemical markers in endometrial cancer treated with chemotherapy. . 2009 ASCO Annual Meeting ,e16551 . |
| 535 | Cost-effectiveness of predictive markers in breast cancer: An analysis from a Swiss perspective. . 2009 ASCO Annual Meeting ,6578. |
| 536 | Expression of stem cell markers in circulating melanoma cells. . 2009 ASCO Annual Meeting ,e22056 . |
| 537 | Use of peripheral blood genomic markers whose expression levels reflect that of breast tumor genomic markers to predict drug treatment and sensitivity. . 2009 ASCO Annual Meeting ,3588. |
| 538 | Important serum markers in malignant lymphoma. . 2009 ASCO Annual Meeting ,e22225 . |
| 539 | Relationship between lymphovascular invasion (LVI) and prognostic markers in different subtypes of breast cancer. . 2009 ASCO Annual Meeting ,e22109 . |
| 540 | SWOG S0342 and S0536: Expression of EGFR protein and markers of epithelial-mesenchymal transformation (EMT) in cetuximab/chemotherapy-treated non-small cell lung cancer (NSCLC). . 2009 ASCO Annual Meeting ,11076. |
| 541 | Ductal carcinoma in situ of the breast and gene expression markers for prediction of invasive recurrence. . 2009 ASCO Annual Meeting ,550. |
| 542 | Thymidilate synthase (TS), thymidine phosphorylase (TP), dihydropyrimidine dehydrogenase (DPD), and dihydrofolate reductase (DHFR) as predictive markers of capecitabine efficacy in breast cancer patients. . 2009 ASCO Annual Meeting ,11033. |
| 543 | Benefit of radiotherapy dose escalation in localized prostate cancer with respect to expression of intrinsic markers of hypoxia. . 2009 ASCO Annual Meeting ,e16068 . |
| 544 | Evaluation of thymidylate synthase and ERCC1 mRNA levels as predictive markers in colorectal cancer patients treated with S-1 and oxaliplatin. . 2009 ASCO Annual Meeting ,e15071 . |
| 545 | . 2009 ASCO Annual Meeting ,8021. |
| 546 | Evaluation of tumor-infiltrating lymphocytes (TIL) and tumor cell apoptosis as predictive markers for response to neoadjuvant chemotherapy in triple-negative breast cancer. . 2009 ASCO Annual Meeting ,559. |
| 547 | Integration of mRNA and microRNA profiles as prognostic and predictive markers in lung adenocarcinoma. . 2009 ASCO Annual Meeting ,7522. |
| 548 | The effects of sorafenib and sunitinib on bone turnover markers in patients with bone metastases from renal cell carcinoma. . 2009 ASCO Annual Meeting ,e16145 . |
| 549 | Evaluation of plasma total and cleaved cytokeratin 18 as predictive markers of chemotherapy in colorectal cancer. . 2009 ASCO Annual Meeting ,e15091 . |
| 550 | Gene expression profiling and copy number analysis to identify predictive molecular markers in breast cancer: Successful use of formalin fixed paraffin embedded tissue (FFPE). . 2009 ASCO Annual Meeting ,574. |
| 551 | The role of KRAS, BRAF, NRAS, and PIK3CA mutations as markers of resistance to cetuximab in chemorefractory metastatic colorectal cancer. . 2009 ASCO Annual Meeting ,4020. |
| 552 | Evaultion of CD133, VEGF, or EGFR as predictive markers of distant recurrence after preoperative chemoradiotherapy in rectal cancer. . 2009 ASCO Annual Meeting ,4050. |
| 553 | Association of baseline and on-study tumor biopsy markers with clinical activity in patients (pts) with advanced melanoma treated with ipilimumab. . 2009 ASCO Annual Meeting ,9008. |
| 554 | Efficacy and safety of gefitinib and potential prognostic value of soluble EGFR, EGFR mutations, and tumor markers in a Gynecologic Oncology Group phase II trial of persistent or recurrent endometrial cancer. . 2009 ASCO Annual Meeting ,e16542 . |
| 555 | Use of surface-enhanced laser desorption/ionisation time-of-flight mass spectrometry (SELDI-TOF-MS) to detect breast cancer markers in serum. . 2009 ASCO Annual Meeting ,e22133 . |
| 556 | Induction of primary systemic therapy by weekly paclitaxel: Predictive value of hormone receptors, HER-2, topoisomerase II- alpha, and other biological markers in relation to pathological complete response. . 2009 ASCO Annual Meeting ,e11525 . |
| 557 | Development of an assay for . 2009 ASCO Annual Meeting ,e15048 . |
| 558 | Involvement of ppGalNAc-T6, a new colon cancer marker, in the molecular basis of simple mucin-type . 2009 ASCO Annual Meeting ,e15060 . |
| 559 | Correlation between expression of MCM6, a new proliferative marker, and treatment outcome in patients with Hodgkin's disease. . 2009 ASCO Annual Meeting ,e19547 . |
| 560 | Significance of serum colony-stimulating factor-1 as a breast cancer marker. . 2009 ASCO Annual Meeting ,11071. |
| 561 | A six-panel PCR of both plasma and blood for EBV-DNA as tumor marker for nasopharyngeal carcinoma patients treated in the United States. . 2009 ASCO Annual Meeting ,e17014 . |
| 562 | Correlation of serum urokinase plasminogen activator (uPA) to progression of recurrent malignant glioma during bevacizumab treatment: A marker of invasive phenotype and a candidate to monitor therapy. . 2009 ASCO Annual Meeting ,2059. |
| 563 | Identification of candidate genetic markers predicting sensitivity to sorafenib and sunitinib. J. Li, J. Wu, G. Jiang, S. Yashinsky, L. Li; Karmanos Cancer Institute, Wayne State University, Detroit, MI; Indiana University, Indianapolis, IN . 2010 ASCO Annual Meeting ,e13610 . |
| 564 | An exploratory determination of new bone markers in natural history of prostate cancer (PC) patients. J. Ayllon, J. Medioni, R. Elaidi, F. Leviel, E. Barrascout, B. Beuselinck, F. Scotte, S. Oudard, G. Maruani, P. Houillier; Medical Oncology Department, Georges Pompidou European Hospital, Paris, France; Georges Pompidou European Hospital, Paris, France; Physiology, Functional Exploration and Radio-isotope Department, Georges Pompidou European Hospital, Paris, France . 2010 ASCO Annual Meeting ,e15121 . |
| 565 | Early magnesium modifications as a surrogate markers of efficacy of cetuximab-based anticancer treatment in KRAS wild-type colorectal cancer patients. B. Vincenzi, D. Santini, S. Galluzzo, F. Loupakis, P. Correale, R. Addeo, S. Del Prete, A. Falcone, G. Francini, G. Tonini; Universit脿 Campus Bio-Medico, Rome, Italy; U.O. Oncologia Medica 2 Universitaria, Azienda Ospedaliera-Universitaria Pisana, Pisa, Italy; Section of Medical Oncology Department "G.Segre" of Pharmacology, Siena University School of Medicine, Siena, Italy; Ospedale San Giovanni Di Dio, Napoli, Italy; San Giovanni Di Dio Hospital, Napoli, Italy; Division of Medical Oncology 2, Azienda Ospedaliero-Universitaria Pisana, Istituto Toscano Tumori, Pisa, Italy; Medical Oncology Section, Siena, Italy . 2010 ASCO Annual Meeting ,3564. |
| 566 | Molecular prognostic and predictive markers in gynecologic cancers: The translational 1 (T-1) study of the Multicentre Italian Trials in Ovarian Cancer and Gynecologic Malignancies (MITO) group. A. Mosconi, L. Crino, V. Ludovini, G. Scambia, G. Ferrandina, E. Breda, R. Sabbatini, C. Caserta, V. De Angelis, S. Pignata; Medical Oncology, Perugia, Italy; MITO, Roma, Italy; MITO, Campobasso, Italy; Medical Oncology, Ospedale Fatebenefratelli, Roma, Italy; Medical Oncology University, Modena, Italy; Medical Oncology, Terni, Italy; MITO, Napoli, Italy . 2010 ASCO Annual Meeting ,TPS340 . |
| 567 | PTEN/p-AKT expression as predictive markers for cetuximab in colorectal cancer. T. Yokota, N. Shibata, T. Ura, D. Takahari, K. Shitara, T. Shibata, K. Muro, Y. Yatabe; Aichi Cancer Center Hospital, Nagoya, Japan . 2010 ASCO Annual Meeting ,e14031 . |
| 568 | Potential markers predicting bevacizumab efficacy for metastatic colorectal cancer patients. M. Suenaga, S. Matsusaka, K. Takagi, Y. Kuboki, T. Watanabe, E. Shinozaki, K. Chin, N. Mizunuma, K. Hatake; Department of Medical Oncology, Cancer Institute Hospital, Tokyo, Japan; Cancer Institute Hospital, Tokyo, Japan . 2010 ASCO Annual Meeting ,e14107 . |
| 569 | Circulating tumor cells as prognostic and predictive markers in metastatic breast cancer patients receiving first-line therapy. M. Giuliano, A. Giordano, L. Hsu, B. C. Handy, N. T. Ueno, E. Andreopoulou, R. H. Alvarez, V. Valero, G. N. Hortobagyi, M. Cristofanilli; University of Naples Federico II, Napoli, Italy; University of Texas M. D. Anderson Cancer Center, Houston, TX . 2010 ASCO Annual Meeting ,1033. |
| 570 | Association of germ-line genetic markers in H. A. Ball, C. Xu, C. N. Sternberg, N. Bing, D. Rajagopalan, C. F. Spraggs, V. E. Mooser, R. G. Amado, L. R. Cardon, L. Pandite; GlaxoSmithKline, Collegeville, PA; GlaxoSmithKline, Harlow, United Kingdom; San Camillo-Forlanini Hospital, Rome, Italy; GlaxoSmithKline, King of Prussia, PA; GlaxoSmithKline, Research Triangle Park, NC . 2010 ASCO Annual Meeting ,4520. |
| 571 | Markers associated with circulating endothelial cells (CEC) in advanced NSCLC: Prognostic value. A. Blasco, S. Gallach, L. Garcia, E. Jantus, R. Sirera, E. Sanmartin, N. del Pozo, R. M. Bremnes, A. Berrocal, C. Camps; Consorcio Hospital General Universitario de Valencia, Valencia, Spain; Fundaci贸n para la Investigaci贸n del Hospital General Universitario de Valencia, Valencia, Spain; Universidad Polit茅cnica de Valencia, Valencia, Spain; Institute of Clinical Medicine, University of Troms酶, Troms酶, Norway . 2010 ASCO Annual Meeting ,e21003 . |
| 572 | Plasma angiogenic markers in patients with metastatic breast cancer treated with weekly docetaxel. G. Papaxoinis, D. G. Pectasides, I. Korantzis, A. Koutras, P. A. Kosmidis, H. Linardou, G. Aravantinos, I. Varthalitis, K. T. Kalogeras, G. Fountzilas; Hellenic Cooperative Oncology Group Data Office, Athens, Greece . 2010 ASCO Annual Meeting ,e21004 . |
| 573 | Molecular types and prognostic markers uPA/PAI-1 for 2,497 early breast cancer patients in the multicenter, randomized NNBC 3-Europe trial. E. J. Kantelhardt, C. Thomssen, M. Vetter, C. Meisner, M. Schmidt, P. Martin, F. Sweep, G. Von Minckwitz, M. Schmitt, N. Harbeck, On behalf of the NNBC 3-Europe study group.; Department of Gynecology, Martin Luther University, Halle, Germany; Institute for Medical Biometry, Eberhard-Karls-University, Tuebingen, Germany; Department of Obstetrics and Gynecology, Johannes Gutenberg University, Mainz, Germany; Laboratoire de Transfer en Oncologie Biologieque, lxAP-HM, Marseille, France; Chemical Endocrinology, Radboud University, Nijmegen, Netherlands; German Breast Group, Neu-Isenburg, Germany; Technical University Munich, Klinikum Rechts der Isar, Munich, Germany; Department of Obstetrics and Gynecology, Breast Center, University of Cologne, K枚ln, Germany . 2010 ASCO Annual Meeting ,10539. |
| 574 | Quantitative assessment of diagnostic markers and correlations with efficacy in two phase II studies of trastuzumab-DM1 (T-DM1) for patients (pts) with metastatic breast cancer (MBC) who had progressed on prior HER2-directed therapy. P. LoRusso, I. E. Krop, H. A. Burris III, S. J. Vukelja, K. Miller, M. Zheng, Y. Chu, M. Lu, L. C. Amler, H. S. Rugo; Karmanos Cancer Institute, Wayne State University, Detroit, MI; Dana-Farber Cancer Institute, Boston, MA; Sarah Cannon Research Institute/Tennessee Oncology, Nashville, TN; Texas Oncology, Tyler, TX; Indiana University Melvin and Bren Simon Cancer Center, Indianapolis, IN; Genentech, South San Francisco, CA; University of California, San Francisco Helen Diller Family Comprehensive Cancer Center, San Francisco, CA . 2010 ASCO Annual Meeting ,1016. |
| 575 | Markers of anaerobic glycolysis as predictive factor in neoadjuvant chemoradiotherapy of rectal cancer. D. Sun, B. Shim, J. Jung, K. Lee, H. Kim, S. Hong, S. Kim, H. Cho; St. Vincent's Hospital, Suwon, South Korea; Department of Surgery, College of Medicine, Catholic University of Korea, Seoul, South Korea; Seoul St. Mary's Hospital, Catholic University of Korea, Seoul, South Korea . 2010 ASCO Annual Meeting ,10573. |
| 576 | Correlation of pharmacogenetic markers with docetaxel-based chemotherapy resistance in patients with advanced/metastatic non-small cell lung cancer. C. Papadaki, E. Tsaroucha, L. Kaklamanis, D. Mavroudis, E. Lagoudaki, M. Trypaki, E. Tsakalaki, V. Georgoulias, I. Sougklakos; Laboratory of Tumor Cell Biology, School of Medicine, University of Crete, Heraklion, Greece; 8th Department of Pulmonary Diseases, Sotiria General Hospital, Athens, Greece; Department of Pathology, Onassis Center for Cardiovascular Diseases, Athens, Greece; Department of Medical Oncology, University General Hospital of Heraklion, Heraklion, Greece; Department of Pathology, University General Hospital of Heraklion, Heraklion, Greece; Hellenic Oncology Research Group, Athens, Greece . 2010 ASCO Annual Meeting ,7615. |
| 577 | Identification of prognostic genomic markers in patients with localized clear cell renal cell carcinoma (ccRCC). B. I. Rini, M. Zhou, H. Aydin, P. Elson, T. Maddala, D. Knezevic, L. Parodi, R. M. Bukowski, W. F. Novotny, J. W. Cowens; Cleveland Clinic, Cleveland, OH; Genomic Health, Redwood City, CA; Pfizer, Inc., New York, NY; Cleveland Clinic Taussig Cancer Institute, Cleveland, OH . 2010 ASCO Annual Meeting ,4501. |
| 578 | Beyond KRAS: The quest for novel genetic markers predictive for response to anti-epidermal growth factor receptor (EGFR) therapy in patients with metastatic colorectal cancer (mCRC). A. Sood, D. McClain, R. Seetharam, M. Al-rahamneh, A. Kaubisch, L. Rajdev, K. Tanaka, J. Mariadason, S. Goel; Montefiore Medical Center, Bronx, NY; Morristown Memorial Hospital, Morristown, NJ; Ludwig Institute for Cancer Research, Melbourne, Australia; Montefiore Cancer Center, Bronx, NY . 2010 ASCO Annual Meeting ,3567. |
| 579 | Molecular markers that predict for recurrence in men with margin-positive localized prostate cancer. P. Yip, J. Kench, K. Rasiah, R. Benito, C. Lee, S. Henshall, R. Sutherland, L. Horvath; Department of Medical Oncology, Sydney Cancer Centre, Royal Prince Alfred Hospital, Sydney, Australia; Department of Tissue Pathology and Diagnostic Oncology, Royal Prince Alfred Hospital, Sydney, Australia; Department of Urology, Royal North Shore Hospital, St. Lenonards, Sydney, Australia; Garvan Institute of Medical Research, Darlinghurst, Sydney, Australia . 2010 ASCO Annual Meeting ,4650. |
| 580 | Association of epithelial mesenchymal transition (EMT) markers and outcome measures in advanced non-small cell lung cancer (NSCLC) patients treated with erlotinib. N. B. Shah, M. J. Fidler, K. K. Walters, E. Braun, S. Basu, H. Harasty, J. Coon, M. Pool, T. A. Hensing, P. D. Bonomi; Rush University Medical Center, Chicago, IL; NorthShore University HealthSystem, Evanston, IL . 2010 ASCO Annual Meeting ,7550. |
| 581 | Genome-wide association study for germline prognostic markers in colorectal cancer. A. Walther, E. Domingo, D. Mesher, E. Johnstone, T. Orntoft, P. Sasieni, M. Dunlop, S. Tejpar, D. J. Kerr, I. Tomlinson, Victor Trial Steering Group and Petacc TR Steering Group; Wellcome Trust Centre for Human Genetics, Oxford, United Kingdom; Wolfson Institute of Preventive Medicine, London, United Kingdom; University of Oxford, Oxford, United Kingdom; Aarhus University Hospital, Skejby, Denmark; University of Edinburgh, Edinburgh, United Kingdom; Human Genetics Unit, Medical Research Council, Edinburgh, United Kingdom; Digestive Oncology Unit and Center for Human Genetics, University Hospital Gasthuisberg, Leuven, Belgium; Sidra Medical and Research Center, Doha, Qatar . 2010 ASCO Annual Meeting ,3514. |
| 582 | Serum markers to monitor response to zoledronic acid in patients with bone metastases from breast cancer. L. Mercatali, T. Ibrahim, E. Sacanna, R. Ricci, E. Scarpi, F. Fabbri, P. Serra, C. Tison, D. Amadori; Osteo-Oncology Center, Meldola, Italy; I.R.S.T., Meldola, Italy . 2010 ASCO Annual Meeting ,1105. |
| 583 | Proliferation and lymphangiogenesis markers, and risk of lymph-nodal metastasis in early cervical cancer. S. Bogliolo, A. Buenerd, P. Mathevet, F. Lecuru; University Claude Bernard, Lyon, France; Women and Children Department, San Paolo Hospital, Savona, Italy; Centre de Pathologie Est-HFME Hospital, University Claude Bernard Lyon, Bron, France; University Claude Bernard Lyon 1, HFME Hospital Lyon, Bron, France; European Georges Pompidou Hospital, Paris, France . 2010 ASCO Annual Meeting ,5099. |
| 584 | Molecular markers for epithelial-mesenchymal transition (EMT) and tumor aggressiveness in breast carcinoma. S. Sethi, F. H. Sarkar, Q. Ahmed, S. Bandyopadhyay, Z. A. Nahleh, W. Sakr, A. Munkarah, R. Ali-Fehmi; Wayne State University School of Medicine, Detroit, MI; Karmanos Cancer Institute, Wayne State University, Detroit, MI; Henry Ford Health System, Detroit, MI . 2010 ASCO Annual Meeting ,10604. |
| 585 | Early therapeutic intervention based on blood tumor markers (TMs) in patients with primary breast cancer (PBC). J. F. Robertson, J. Mathew, P. Prinsloo, A. Agrawal, E. Gutteridge, E. Merenah, K. Cheung; Division of Breast Surgery, University of Nottingham, Nottingham, United Kingdom; Department of Clinical Pathology, Nottingham University Hospitals, Nottingham, United Kingdom . 2010 ASCO Annual Meeting ,639. |
| 586 | Melanoma cell expression of macrophage markers in AJCC stage I/II melanoma. T. O. Jensen, H. Schmidt, T. Steiniche, M. Hoyer, H. J. Moller; Aarhus University Hospital, Aarhus, Denmark; Department of Oncology, Aarhus University Hospital, Aarhus, Denmark . 2010 ASCO Annual Meeting ,e19034 . |
| 587 | Circulating endothelial cells, microparticles, and markers of inflammation and coagulation in glioblastoma patients before and after protracted temozolomide and radiotherapy. G. Reynes, V. Martinez-Sales, V. Vila, T. Fleitas, M. Martin, E. Reganon; Hospital Universitario La Fe, Valencia, Spain . 2010 ASCO Annual Meeting ,2086. |
| 588 | Prognostic markers for response and overall survival in patients with esophageal adenocarcinoma treated with neoadjuvant therapy. J. M. Leers, G. Lurje, D. Yang, A. Oezcelik, J. A. Hagen, S. R. DeMeester, T. R. DeMeester, H. Lenz; Department of Surgery, University of Cologne, Cologne, Germany; Department for Visceral and Transplantation Surgery, University Hospital, Zurich, Switzerland; University of Southern California Norris Comprehensive Cancer Center, Los Angeles, CA; Department of Surgery, University of Southern California, Los Angeles, CA; University of Southern California, Los Angeles, CA . 2010 ASCO Annual Meeting ,4123. |
| 589 | Use of methylation profiling to identify predictive markers in gastric cancer for respond to 5-fluorouracil (5FU)-based chemotherapy. N. Liem, P. Lim, M. Loh, A. Vaithilingham, R. C. Soong, W. Yong; National University Hospital Singapore, Singapore, Singapore; National University of Singapore, Singapore, Singapore; National University Health System, Singapore, Singapore . 2010 ASCO Annual Meeting ,e14546 . |
| 590 | Prognostic and predictive markers in recurrent high-grade glioma (HGG): Results from the BR12 randomized trial. M. Brada, V. P. Collins, K. Ichimura, L. C. Thompson, R. Gabe, S. P. Stenning, BR12 Collaborators; The Institute of Cancer Research and The Royal Marsden NHS Foundation Trust, Sutton, United Kingdom; Department of Pathology and Molecular Histopathology, Addenbrooke's Hospital, Cambridge, United Kingdom; Department of Pathology, University of Cambridge, Addenbrooke's Hospital, Cambridge, United Kingdom; Medical Research Council Clinical Trials Unit, London, United Kingdom . 2010 ASCO Annual Meeting ,2035. |
| 591 | Breast cancer classification according to three immunohistochemical markers: Clinicopathologic features and prognosis in a single institution from Peru. C. R. Desposorio, S. G. Falcon, F. Hurtado de Mendoza, L. A. Riva, D. Morales, A. Yabar; Hospital Nacional Edgardo Rebagliati Martins, Lima, Peru . 2010 ASCO Annual Meeting ,e12047 . |
| 592 | Relationship among antigenic markers, disease progression, and survival in colorectal cancer (CCR) patients. A. Zwenger, M. V. Croce, A. Segal-Eiras, M. Rabassa, J. Iturbe, C. T. Vallejo, B. A. Leone, G. Grosman; Hospital Regional Neuquen, Neuquen, Argentina; Facultad de Medicina de la Universidad Nacional de la Plata, CINIBA, La Plata, Argentina; Grupo Oncologico Cooperativo del Sur, Neuquen, Argentina . 2010 ASCO Annual Meeting ,e14060 . |
| 593 | Prospective evaluation of predictive and prognostic molecular markers in colorectal carcinomas. R. S. Croner, R. M. Wirtz, B. Lausen, H. Prokosch, C. Roedel, F. Rodel, E. Naschberger, A. Hartmann, W. Hohenberger, M. St眉rzl; Department of Surgery, University of Erlangen-Nuremberg, Erlangen, Germany; Siemens Healthcare Diagnostics, Cologne, Germany; Department of Mathematical Sciences, University of Essex, Colchester, United Kingdom; Chair of Medical Informatics, University of Erlangen-Nuremberg, Erlangen, Germany; University of Frankfurt, Frankfurt, Germany; Department of Radiotherapy and Oncology, University of Frankfurt, Frankfurt, Germany; Division of Molecular and Experimental Surgery, University of Erlangen-Nuremberg, Erlangen, Germany; University of Erlangen-Nuremberg, Erlangen, Germany . 2010 ASCO Annual Meeting ,e14114 . |
| 594 | The changes of molecular markers with neoadjuvant dose-dense doxorubicin hydrochloride, cyclophosphamide, and paclitaxel chemotherapy regimen. D. Sener Dede, B. Gumuskaya, G. Guler, D. A. Onat, K. Altundag, Y. Y. Ozisik; Department of Medical Oncology, Hacettepe University Institute of Oncology, Ankara, Turkey; Diskapi Yildirim Beyazit Education and Research Hospital, Ankara, Turkey; Department of Pathology, Hacettepe University Faculty of Medicine, Ankara, Turkey; Department of Surgery, Hacettepe University Faculty of Medicine, Ankara, Turkey . 2010 ASCO Annual Meeting ,e21028 . |
| 595 | Prognostic significance of proliferation markers and telomerase activity in gastrointestinal stromal tumors. A. Augustinakova, H. Brizova, I. Hilska, M. Kalinova, R. Kodet; 2nd Medical School, Charles University in Prague and Faculty Hospital in Motol, Prague 5, Czech Republic . 2010 ASCO Annual Meeting ,10078. |
| 596 | Comparison of FDG-PET and tumor markers for the diagnosis of lung carcinoma. H. Terai, K. Soejima, K. Naoki, H. Yasuda, S. Yoda, R. Satomi, S. Nakayama, S. Ikemura, T. Satou, A. Ishizaka; School of Medicine, Keio University, Tokyo, Japan . 2010 ASCO Annual Meeting ,e21067 . |
| 597 | Expression of multiple cancer stem cell markers by triple-negative breast carcinomas. A. Behdad, S. Bose; Cedars-Sinai Medical Center, Los Angeles, CA . 2010 ASCO Annual Meeting ,e21108 . |
| 598 | NT-pro-BNP and cTnT as markers for subclinical early-onset anthracycline-induced cardiotoxicity in children: A prospective study. A. M. Mavinkurve-Groothuis, J. Groot-Loonen, T. Feuth, P. Hoogerbrugge, L. Kapusta; Radboud University Medical Centre, Nijmegen, Netherlands . 2010 ASCO Annual Meeting ,e19503 . |
| 599 | Polymorphisms in VEGF, eNOS, COX-2, and IL-8 as predictive markers of response to bevacizumab. L. lo Giudice, M. Di Salvatore, A. Astone, M. Rodriquenz, G. Nazzicone, A. Cassano, C. Bagal脿, C. Santonocito, E. D. Capoluongo, C. Barone; Department of Medical Oncology, Catholic University of the Sacral Heart, Rome, Italy; Medical Oncology, Catholic University of Sacred Heart, Rome, Italy; Department of Biochemistry, Catholic University of the Sacral Heart, Rome, Italy; Catholic University of Sacred Heart, Rome, Italy . 2010 ASCO Annual Meeting ,e13502 . |
| 600 | Use of day 4 CEP and baseline CXCR4 plus CEC as predictive markers for bevacizumab in mCRC. S. Matsusaka, Y. Mishima, M. Suenaga, K. Takagi, E. Shinozaki, Y. Terui, N. Mizunuma, K. Hatake; Department of Medical Oncology, Cancer Institute Hospital, Tokyo, Japan; Cancer Institute Hospital, Tokyo, Japan . 2010 ASCO Annual Meeting ,3599. |
| 601 | Effect of zoledronic acid on tartrate-resistant acid phosphatase isoform 5b (TRAP-5b) and other bone markers in lung cancer patients with bone metastasis. W. Zhang, G. Rabinowits, A. J. Janckila, L. Yam, F. J. Hendler, D. A. Laber, C. Li, G. H. Kloecker; James Graham Brown Cancer Center, University of Louisville, Louisville, KY; Division of Hematology, Veterans Administrative Medical Center, Louisville, KY; Department of Bioinformatics and Biostatistics, School of Public Health and Information Science, University of Louisville, Louisville, KY . 2010 ASCO Annual Meeting ,e18118 . |
| 602 | Phase I/II trial of association of sorafenib in combination with temozolomide in patients with metastatic melanoma: Looking for predictive markers of efficacy. C. Robert, N. Chaput, N. Lassau, A. Auperin, S. Koscielny, E. Hollville, V. Lazar, L. Lacroix, J. Soria, C. Mateus; Institut Gustave Roussy, Villejuif, France; Department of Biopathology, Institut Gustave Roussy, Villejuif, France . 2010 ASCO Annual Meeting ,8552. |
| 603 | The effect of sunitinib on immune parameters and haemopoetic stem cell markers in patients with untreated clear cell renal cancer. S. Chowdhury, T. S. O'Brien, N. Sarwar, J. Shamash, S. McGrath, S. Agrawal, L. Lim, S. M. Rudman, P. G. Harper, T. Powles; Guy's and St. Thomas' Hospital, London, United Kingdom; Guy's Hospital, London, United Kingdom; St. Bartholomew's Hospital, London, United Kingdom; Division of Cancer Studies, King's College London, Guy's Hospital, London, United Kingdom; Barts and The London School of Medicine and Dentistry, London, United Kingdom . 2010 ASCO Annual Meeting ,4620. |
| 604 | Use of a four gene panel of epigenetic markers to classify serrated colonic adenomas with CpG island methylator phenotype. J. Jenab-Wolcott, Y. Yao, W. Yan, S. Schulte, S. Harada, C. Brensinger, B. J. Giantonio, A. Rustgi, A. Sepulveda; University of Pennsylvania, Philadelphia, PA; Johns Hopkins University School of Medicine, Baltimore, MD; Abramson Cancer Center, University of Pennsylvania, Philadelphia, PA . 2010 ASCO Annual Meeting ,3600. |
| 605 | Clinical significance of selected angiogenesis and lymphangiogenesis modulators and markers in ovarian cancer patients. D. Klasa-Mazurkiewicz, T. Milczek, M. Jarzab, J. Narkiewicz, B. Lipi帽ska, D. Wydra; Department of Gynecology-Oncology, Medical University of Gda帽sk, ul Kliniczna 1a, Gda帽sk, Poland; Department of Gynecology Oncology, Medical University of Gda帽sk, Kliniczna 1a, Gda帽sk, Poland; Department of Biochemistry, University of Gda帽sk, Gda帽sk, Poland; Gda帽sk, Poland . 2010 ASCO Annual Meeting ,5119. |
| 606 | An academic prospective single-arm phase II clinical trial for evaluation of advanced functional neuroimaging and molecular markers during bevacizumab/irinotecan therapy for recurrent malignant glioma. M. Hutterer, M. Nowosielski, T. Gotwald, D. Putzer, D. Waitz, H. Maier, A. Muigg, H. Kostron, G. Stockhammer; Department for Neurology, Medical University Innsbruck, Innsbruck, Austria; Department for Radiology, Medical University Innsbruck, Innsbruck, Austria; Department for Nuclear Medicine, Medical University Innsbruck, Innsbruck, Austria; Institute for Pathology, Medical University Innsbruck, Innsbruck, Austria; Department for Neurology, Medical University Innsbruck, Innbruck, Austria; Department for Neurosurgery, Medical University Innsbruck, Innsbruck, Austria . 2010 ASCO Annual Meeting ,TPS153 . |
| 607 | ERCC1 protein, mRNA expression, and T19007C polymorphism as prognostic markers in head and neck squamous cell carcinoma (HNSCC) patients treated with surgery and adjuvant cisplatin-based chemoradiation (CRT). G. Castro, F. S. Pasini, S. A. Siqueira, A. R. Ferraz, R. C. Villar, I. M. Snitcovsky, M. H. Federico; Oncologia Clinica, Instituto do Cancer do Estado de S茫o Paulo, S茫o Paulo, Brazil; Faculdade de Medicina da Universidade de Sao Paulo, Sao Paulo, Brazil; Divis茫o de Anatomia Patol贸gica, Hospital das Clinicas da Faculdade de Medicina da Universidade de Sao Paulo, Sao Paulo, Brazil; Disciplina de Cirurgia de Cabe莽a e Pescoco, Faculdade de Medicina da Universidade de Sao Paulo, Sao Paulo, Brazil; Servi莽o de Radioterapia-InRad, Hospital das Clinicas da Faculdade de Medicina da Universidade de Sao Paulo, Sao Paulo, Brazil; Disciplina de Oncologia, Faculdade de Medicina da Universidade de Sao Paulo, Sao Paulo, Brazil . 2010 ASCO Annual Meeting ,5540. |
| 608 | A. Wozniak, E. Van Mieghem, H. Ardon, S. De Vleeschouwer, J. Menten, R. Sciot, F. Van Calenbergh, S. Van Gool, M. Debiec- Rychter, P. M. Clement; Laboratory of Experimental Oncology, Catholic University of Leuven, Leuven, Belgium; Department of General Medical Oncology, University Hospitals Leuven, Leuven, Belgium; Department of Neurosurgery, University Hospitals Leuven, Leuven, Belgium; Department of Radiotherapy, University Hospitals Leuven and Catholic University of Leuven, Leuven, Belgium; Department of Pathology, University Hospitals Leuven and Catholic University of Leuven, Leuven, Belgium; Department of Pediatric Oncology, University Hospitals Leuven and Catholic University of Leuven, Leuven, Belgium; Department of Human Genetics, University Hospitals Leuven and Catholic University of Leuven, Leuven, Belgium; Department of General Medical Oncology, University Hospitals Leuven and Catholic University of Leuven, Leuven, Belgium . 2010 ASCO Annual Meeting ,2053. |
| 609 | EGFR and G. Liu, D. Cheng, A. Le Maitre, N. Liu, Z. Chen, L. Seymour, K. Ding, F. A. Shepherd, M. S. Tsao; Princess Margaret Hospital, University of Toronto, Toronto, ON, Canada; Queen's University, Kingston, ON, Canada; NCIC Clinical Trials Group, Kingston, ON, Canada; University Health Network, Princess Margaret Hospital, Toronto, ON, Canada . 2010 ASCO Annual Meeting ,7538. |
| 610 | Are MGMT promoter methylation and EGFR mutations early markers of tumor progression in colorectal cancer? T. Wenner, B. Metzger, L. Chambeau, G. Mahon, M. Pauly, J. Kaiser, G. J. Berchem, M. Dicato; RCMS, Luxembourg, Luxembourg; Zitha Klinik, Luxembourg, Luxembourg; Centre Hospier du Luxembourg, Luxembourg, Luxembourg; Centre Hospitalier Luxembourg, Luxembourg, Luxembourg . 2010 ASCO Annual Meeting ,3584. |
| 611 | KRAS and BRAF mutational status and PTEN, cMET, and IGF1R expression as predictive markers of response to cetuximab plus chemotherapy in metastatic colorectal cancer (mCRC). M. Di Salvatore, A. Inno, A. Orlandi, M. Martini, G. Nazzicone, D. Ferraro, A. Astone, A. Cassano, L. Larocca, C. Barone; Medical Oncology, Catholic University of Sacred Heart, Rome, Italy; Institute of Pathological Anatomy, Catholic University of the Sacred Heart, Rome, Italy; Catholic University of Sacred Heart, Rome, Italy . 2010 ASCO Annual Meeting ,e14065 . |
| 612 | 2010 Molecular Markers . ,. |
| 613 | Immune System and Immunotherapeutic Markers . ,. |
| 614 | Circulating levels of EGFR ligands as surrogate markers of EGFR inhibition with cetuximab in metastatic colorectal cancer (mCRC) patients (pts). C. Cremolini, F. Loupakis, A. Fioravanti, M. Schirripa, P. Orlandi, L. Salvatore, G. Fontanini, R. Danesi, G. Bocci, A. Falcone; U. O. Oncologia Medica 2 Universitaria, Azienda Ospedaliera-Universitaria Pisana, Pisa, Italy; Divisione di Farmacologia e Chemioterapia, Universit脿 di Pisa, Pisa, Italy; Divisione di Anatomia Patologica, Dipartimento di Chirurgia, Universit脿 di Pisa, Pisa, Italy; Division of Chemotherapy and Pharmacology, Department of Internal Medicine, University of Pisa, Pisa, Italy; Division of Medical Oncology 2, Azienda Ospedaliero-Universitaria Pisana, Istituto Toscano Tumori, Universit脿 di Pisa, Pisa, Italy . 2010 Molecular Markers ,99. |
| 615 | Large-scale profiling of serum markers, single-cell polyfunctionality, and antigen diversity of T-cell response against melanoma. C. Ma, A. Chueng, B. Conmin-Anduix, T. Condon, A. Ribas, J. Heath; California Institute of Technology, Pasadena, CA . 2010 Molecular Markers ,148. |
| 616 | Molecular markers of high-grade bone sarcomas revealed by proteomics. A. Kawai, K. Kikuta, D. Kubota, T. Fujiwara, Y. Suehara, E. Kobayashi, A. Hosono, T. Kondo; National Cancer Center Hospital, Tokyo, Japan; National Cancer Center Research Institute, Tokyo, Japan . 2010 Molecular Markers ,5. |
| 617 | Molecular markers meet anatomic staging: The AJCC prepares for personalized medicine. C. C. Compton; National Cancer Institute/National Institutes of Health, Bethesda, MD . 2010 Molecular Markers ,63. |
| 618 | Volatile compounds as molecular markers of melanoma. T. Abaffy; University of Miami, Miami, FL . 2010 Molecular Markers ,15. |
| 619 | Correlation of leukemic cell birth rates, quantified by heavy water labeling, with prognostic markers in early-stage chronic lymphocytic leukemia. E. J. Murphy, C. Emson, L. Rassenti, G. Hayes, S. Turner, N. Chiorazzi, J. R. Brown, D. S. Neuberg, T. J. Kipps, K. Rai; University of California, San Francisco, San Francisco, CA; KineMed, Emeryville, CA; Chronic Lymphocytic Leukemia Research Consortium, University of California, San Diego Moores Cancer Center, La Jolla, CA; Long Island Jewish Medical Center, New York, NY; Dana-Farber Cancer Institute, Boston, MA; Department of Biostatistics and Computational Biology, Dana-Farber Cancer Institute, Boston, MA; University of California, San Diego Moores Cancer Center, La Jolla, CA . 2010 Molecular Markers ,53. |
| 620 | Clinical significance of tumor markers as prognostic factors in patients with metastatic gastric cancer receiving first-line chemotherapy. J. Jo, M. Ryu, D. Koo, B. Ryoo, H. J. Kim, J. Lee, H. Chang, T. W. Kim, K. D. Choi, G. H. Lee, H. Jung, K. C. Kim, J. H. Yook, S. T. Oh, B. S. Kim, J. Kim, Y. Kang; Department of Oncology, University of Ulsan College of Medicine, Asan Medical Center, Seoul, South Korea; Department of Oncology, Asan Medical Center, University of Ulsan College of Medicine, Seoul, South Korea; Clinical Epidemiology and Biostatistics, University of Ulsan College of Medicine, Asan Medical Center, Seoul, South Korea; Department of Gastroenterology, University of Ulsan College of Medicine, Asan Medical Center, Seoul, South Korea; Department of Gastroenterology, Asan Medical Center, University of Ulsan College of Medicine, Seoul, South Korea; Department of Surgery, Asan Medical Center, University of Ulsan College of Medicine, Seoul, South Korea; Department of Surgery, University of Ulsan College of Medicine, Asan Medical Center, Seoul, South Korea; Asan Medical Center, Seoul, South Korea . 2011 ASCO Annual Meeting ,4035. |
| 621 | Polymorphisms as markers of sunitinib efficacy and toxicity in first-line treatment of renal clear cell carcinoma: Final results of a multicentric prospective study by the Spanish Oncology Genitourinary Group. J. Garcia-Donas, E. Esteban, L. J. Leandro-Garc铆a, D. E. Castellano, A. Gonzalez del Alba, M. A. Climent, J. A. Arranz, E. Gallardo, J. Puente, J. Bellmunt, B. Mellado, E. Mart铆nez, F. Moreno, A. Font, M. Robledo, C. Rodriguez de Antona, Spanish Oncology GenitoUrinary Group SOGUG; Hospital Universitario Fundaci贸n Alcorc贸n, Alcorcon, Spain; Hospital General de Asturias, Oviedo, Spain; Spanish National Cancer Research Center, Madrid, Spain; Hospital Universitario 12 de Octubre, Madrid, Spain; Hospital Son Dureta, Palma de Mallorca, Spain; Fundacion Instituto Valenciano de Oncolog铆a, Valencia, Spain; Hospital General Universitario Gregorio Mara帽贸n, Madrid, Spain; Corporacio Parc Tauli, Sabadell, Spain; Hospital Clinico Universitario San Carlos, Madrid, Spain; University Hospital del Mar-IMIM, Barcelona, Spain; Hospital Clinic University of Barcelona, Barcelona, Spain; Hospital de Jaen, Jaen, Spain; Fundacion Hospital Fuenlabrada, Fuenlabrada, Spain; Catalan Institute of Oncology, Hospital Germans Trias i Pujol, Barcelona, Spain . 2011 ASCO Annual Meeting ,4559. |
| 622 | Association between bone turnover markers and skeletal-related events in patients with breast cancer and bone metastases on treatment with bisphosphonates: ZOMAR study results at nine months of follow-up. A. Barnadas, C. De la Piedra, C. Crespo, P. Gomez Pardo, L. Calvo, E. G. Calvo, M. Ruiz-Borrego, J. Rifa, L. Manso, A. Anton, M. Codes, M. Margeli, A. Murias, J. Salvador, M. A. Segu铆-Palmer, A. De Juan, J. Gavila, D. Perez, M. Luque, I. Tusquets; Medical Oncology Department, Hospital de la Santa Creu i Sant Pau, Barcelona, Spain; Fundacion Jimenez Diaz, Madrid, Spain; Hospital Ramon y Cajal, Madrid, Spain; Breast Cancer Center, Vall d'Hebron University Hospital, Barcelona, Spain; CHU Juan Canalejo, A Coruna, Spain; Hospital Civil de Basurto, Bilbao, Spain; Hospital Universitario Virgen del Roc铆o, Seville, Spain; Hospital Son Espases, Palma de Mallorca, Spain; Hospital Universitario 12 de Octubre, Madrid, Spain; Hospital Miguel Servet, Zaragoza, Spain; Hospital Virgen de la Macarena, Sevilla, Spain; Hospital Germans Trias i Pujol, Badalona, Spain; Hospital Universitario Insular Gran Canaria, Gran Canaria, Spain; Hospital Virgen de Valme, Seville, Spain; Corporaci贸 Sanit脿ria Parc Taul铆, Sabadell, Spain; Hospital Marques de Valdecilla, Santander, Spain; Instituto Valenciano de Oncologia, Valencia, Spain; Hospital Costa del Sol, Marbella, Spain; Asturias Central University Hospital, Oviedo, Asturias, Spain; Hospital del Mar, Barcelona, Spain . 2011 ASCO Annual Meeting ,594. |
| 623 | The usefulness of serum tumor markers in monitoring of response to systemic chemotherapy in patients with gastric cancer: Ca 72-4 and CEA. B. Han, D. Y. Zang, H. J. Kim, H. Y. Kim; Department of Internal Medicine, Hallym University Medical Center, Hallym University College of Medicine, Anyang, South Korea . 2011 ASCO Annual Meeting ,e14580 . |
| 624 | Molecular prognostic markers in advanced gastric cancer: Correlative study in the Japan Clinical Oncology Group trial JCOG9912. Y. Yamada, J. Mizusawa, A. Takashima, K. Nakamura, Y. Tsuji, Y. Suzuki, K. Amagai, K. Yamaguchi, K. Konishi, T. Yoshino, T. Denda, W. Koizumi, S. Ohkawa, H. Kawai, H. Kojima, H. Nishizaki, T. Nishina, A. Tsuji, H. Fukuda, N. Boku; National Cancer Center Hospital, Tokyo, Japan; JCOG Data Center, National Cancer Center, Tokyo, Japan; JCOG Operations Office, National Cancer Center, Tokyo, Japan; KKR Sapporo Medical Center Tonan Hospital, Hokkaido, Japan; Yamagata Prefectural Central Hospital, Yamagata, Japan; Ibaraki Prefectural Central Hospital, Kasama, Japan; Saitama Cancer Center, Saitama, Japan; Showa University, Tokyo, Japan; National Cancer Center Hospital East, Kashiwa, Japan; Chiba Cancer Center, Chiba, Japan; Kitasato University, Sagamihara, Japan; Division of Gastroenterology, Kanagawa Cancer Center Hospital, Yokohama, Japan; Aichi Cancer Center Hospital, Nagoya, Japan; Aichi Cancer Center Aichi Hospital, Okazaki, Japan; Hyogo Cancer Center, Akashi, Japan; National Hospital Organization Shikoku Cancer Center, Ehime, Japan; Kochi Health Sciences Center, Kochi, Japan; Saint Marianna University School of Medicine, Kanagawa, Japan . 2011 ASCO Annual Meeting ,4021. |
| 625 | Pharmacodynamic and predictive markers of ipilimumab on melanoma patients鈥?T-cells. J. S. Weber, B. Yu, M. Hall, D. Morelli, D. Yu, Y. Zhang, X. Zhao, A. Sarnaik, W. Wang; Moffitt Cancer Center, Tampa, FL; H. Lee Moffitt Cancer Center &amp; Research Institute, Tampa, FL . 2011 ASCO Annual Meeting ,2503. |
| 626 | MicroRNAs as prognostic markers for survival in renal cell carcinoma conventional type T 2-4. Z. A. Dotan, E. Fridman, Y. Spector, I. Barshack, A. Chajut, S. Rosenwald, S. Gilad, R. Catane, E. Meiri, R. Berger, R. T. Aharonov, E. Ezra, J. Ramon; Chaim Sheba Medical Center, Tel-Hashomer, Israel; Sheba Medical Center, Tel HaShomer, Israel; Rosetta Genomics, Rehovot, Israel; Department of Pathology, Sheba Medical Center, Tel Hashomer, Israel; Oncology Institue, Chaim Sheba Medical Center, Ramat-Gan, Israel; Uro-oncologist, Tel-Hashomer, Tel-Hashomer, Israel; Tel-Hashomer, Tel-Hashomer, Israel; Urology Department, Tel-Hashomer, Israel . 2011 ASCO Annual Meeting ,e21115 . |
| 627 | Biological markers to predict response to neoadjuvant chemotherapy (NCT) in patients with locally advanced breast cancer (LABC): Ready for prime time? M. S. Pino, A. Fabi, M. Tedeschi, M. Mottolese, P. Papaldo, P. Vici, G. Ferretti, C. Nistic貌, M. Russillo, F. Cuppone, A. Di Benedetto, C. Botti, D. Giannarelli, F. Cognetti; Regina Elena National Cancer Institute, Rome, Italy; Medical Oncology, Regina Elena National Cancer Institute, Rome, Italy . 2011 ASCO Annual Meeting ,e11073 . |
| 628 | Molecular markers of the EGFR pathway in erlotinib-treated patients with advanced pancreatic cancer (APC): Translational analyses of a randomized, cross-over AIO phase III trial. S. H. Boeck, A. Jung, R. P. Laubender, J. Neumann, R. Egg, C. Goritschan, U. Vehling-Kaiser, C. Winkelmann, L. Fischer von Weikersthal, M. R. Clemens, T. C. Gauler, A. Marten, S. Klein, G. Kojouharoff, M. Barner, M. Geissler, T. F. Greten, U. Mansmann, T. Kirchner, V. Heinemann; Department of Internal Medicine III, Klinikum Grosshadern, University of Munich, Munich, Germany; Department of Pathology, University of Munich, Munich, Germany; Institute for Medical Informatics, Biometry and Epidemiology, University of Munich, Munich, Germany; Practice for Medical Oncology, Landshut, Germany; Department of Internal Medicine, Krankenhaus Lutherstadt-Wittenberg, Lutherstadt-Wittenberg, Germany; Department of Oncology, Gesundheitszentrum St. Marien GmbH, Amberg, Germany; Klinikum Mutterhaus, Trier, Germany; West German Tumor Center, University Hospital Essen, Essen, Germany; Department of Surgery, University of Heidelberg, Heidelberg, Germany; Department of Internal Medicine IV, Klinikum Bayreuth, Bayreuth, Germany; Practice for Medical Oncology, Darmstadt, Germany; Department of Internal Medicine, Evangelisches Krankenhaus Koeln-Weyertal, Koeln, Germany; Department of Gastroenterology and Oncology, Klinikum Esslingen, Esslingen, Germany; Department of Gastroenterology, Hepatology and Endocrinology, Medical School Hannover, Hannover, Germany . 2011 ASCO Annual Meeting ,4047. |
| 629 | Molecular markers and outcomes in penile squamous cancer (PSC). T. B. Dorff, A. Schuckman, R. Schwartz, K. Danenberg, Y. Ma, J. Cai, S. Rashad, E. C. Skinner, D. I. Quinn, J. K. Pinski; University of Southern California Norris Comprehensive Cancer Center, Los Angeles, CA; University of Southern California Institute of Urology, Los Angeles, CA; Keck School of Medicine of the University of Southern California, Los Angeles, CA; Response Genetics, Inc., Los Angeles, CA . 2011 ASCO Annual Meeting ,e15016 . |
| 630 | Identification of pre- and post-treatment markers of efficacy in patients with renal cancer treated with MVA-5T4 in a phase III study. R. Harrop, W. H. Shingler, M. McDonald, P. Treasure, R. J. Amato, R. E. Hawkins, H. Kaufman, J. de Belin, M. Goonewardena, S. Naylor; Oxford BioMedica, Oxford, United Kingdom; Peter Treasure Statistical Services Ltd, King's Lynn, United Kingdom; University of Texas Health Science Center at Houston, Houston, TX; Christie Cancer Research UK, Manchester, United Kingdom; Rush University Medical Center, Chicago, IL . 2011 ASCO Annual Meeting ,2542. |
| 631 | Identification of prognostic tumor markers in HIV+ diffuse large B-cell lymphoma (DLBCL). C. Chao, M. Silverberg, D. I. Abrams, R. Haque, H. D. Zha, O. Martinez-Maza, M. McGuire, M. D. Chi, B. Castor, L. Xu, J. Said; Kaiser Permanente Southern California, Pasadena, CA; Kaiser Permanente Northern California, Oakland, CA; San Francisco General Hospital, San Francisco, CA; Southern California Permanente Medical Group, Pasadena, CA; University of California, Los Angeles, Los Angeles, CA; University of California, Los Angeles School of Medicine, Los Angeles, CA . 2011 ASCO Annual Meeting ,8055. |
| 632 | Relation of inflammatory markers, disease stage and control, and chemotherapy in patients with breast cancer. O. Dizdar, C. Arslan, S. Aksoy, O. Atmaca, Y. Tekinel, E. Sener, I. H. Gullu, Y. Y. Ozisik, K. Altundag; Ankara Ataturk Education and Research Hospital, Ankara, Turkey; Department of Medical Oncology, Hacettepe University Institute of Oncology, Ankara, Turkey; Ankara Numune Training and Research of Medical Oncology, Ankara, Turkey; Hacettepe University, Ankara, Turkey; Hacettepe University Institute of Oncology, Ankara, Turkey . 2011 ASCO Annual Meeting ,e21159 . |
| 633 | Prognostic implications of lymphangiogenic markers in early-stage NSCLC. E. Sanmartin, S. Gallach, R. Sirera, C. Hernando, A. Honguero, R. Guijarro, M. Martorell, C. Camps, E. Jantus-Lewintre; Fundaci贸n para la Investigaci贸n del Hospital General Universitario de Valencia, Valencia, Spain; Consorcio Hospital General Universitario de Valencia, Valencia, Spain; Complejo Hospitalario Universitario de Albacete, Albacete, Spain . 2011 ASCO Annual Meeting ,e21108 . |
| 634 | Predictive markers of utilization of chemotherapy in patients with advanced colorectal cancer (CRC): A population-based study. S. Ahmed, T. Zhu, R. Lee-Ying, C. Ubhi, V. Kundapur, R. Alvi, K. Haider; Saskatoon Cancer Centre, Saskatoon, SK, Canada; Department of Medicine, University of Saskatchewan, Saskatoon, SK, Canada; Department of Medicine, Saskatoon, SK, Canada; Saskatoon Cancer Center, Saskatoon, SK, Canada; Saskatoon Cancer Centre, University of Saskatchewan, Saskatoon, SK, Canada . 2011 ASCO Annual Meeting ,6053. |
| 635 | Prognostic importance of inflammation, apoptosis, and angiogenesis markers in high-grade glial tumors. U. Demirci, U. E. Bagriacik, S. Buyukberber, U. Coskun, M. Yaman, A. Ozet, K. Uslu, M. Benekli; Department of Medical Oncology, Gazi University Faculty of Medicine, Ankara, Turkey; Department of Immunology, Gazi University Faculty of Medicine, Ankara, Turkey; Gazi University Hospital, Ankara, Turkey; Refik Saydam National Public Health Agency, Ankara, Turkey; Faculty of Medicine, Department of Medical Oncology, Gazi University, Ankara, Turkey . 2011 ASCO Annual Meeting ,e12522 . |
| 636 | Prognositic value of suppressed markers of bone turnover (BTO) after 6 months of androgen deprivation therapy (ADT) in prostate cancer. J. Sharma, C. T. Yiannoutsos, N. M. Hahn, C. Sweeney; Dana-Farber Cancer Institute, Boston, MA; Hoosier Oncology Group, Indiana University, Indianapolis, IN; Indiana University Melvin and Bren Simon Cancer Center, Indianapolis, IN . 2011 ASCO Annual Meeting ,4594. |
| 637 | Use of PSA percent tumor dimensional markers to predict biochemical recurrence after radical prostatectomy in clinically localized prostate cancer. R. Mizuno, J. Nakashima, K. Shinoda, H. Kono, E. Kikuchi, H. Nagata, H. Asanuma, A. Miyajima, K. Nakagawa, M. Oya; Department of Urology, Keio University School of Medicine, Tokyo, Japan; Keio University, Tokyo, Japan . 2011 ASCO Annual Meeting ,e15134 . |
| 638 | Nanoparticle-based ultra-sensitive assay for discovery and evaluation of cancer markers. Y. P. Bao, R. Beal, R. Khan, W. Gibbons; Nanosphere Inc., Northbrook, IL . 2011 ASCO Annual Meeting ,e21169 . |
| 639 | A pilot study of tumor-derived exosomes as diagnostic and prognostic markers in breast cancer patients receiving neoadjuvant chemotherapy. R. E. Raab, T. L. Pawlowski, D. Spetzler, M. R. Klass, C. D. Kuslich, L. S. Bellin, J. H. Wong, A. S. Asch; East Carolina University Brody School of Medicine, Greenville, NC; Caris Life Sciences, Phoenix, AZ; CarisDx, Phoenix, AZ; Caris, Phoenix, AZ; East Carolina University, Greenville, NC; Leo Jenkins Cancer Center, Brody School of Medicine at East Carolina University, Greenville, NC . 2011 ASCO Annual Meeting ,TPS248 . |
| 640 | Utility of novel dynamic clinical indices in patients (pts) enrolled in a phase I (Ph I) oncology trial as markers of prognosis and treatment benefit. J. E. Ang, D. Olmos, V. Moreno Garcia, A. Brunetto, D. Papadatos-Pastos, H. Arkenau, M. Blanco, R. D. Baird, P. A. Cassier, D. S. W. Tan, R. S. Kristeleit, S. Dolly, K. J. Shah, B. Amin, B. G. Anghan, L. R. Molife, U. Banerji, J. S. De Bono, S. B. Kaye; The Royal Marsden Hospital, Sutton, United Kingdom; The Institute for Cancer Research and Royal Marsden Hospital, Sutton, United Kingdom; Sarah Cannon Research Institute, London, United Kingdom; Royal Marsden Hospital, Sutton, United Kingdom; Royal Marsden Hospital &amp; Institute of Cancer Research, Sutton, United Kingdom; Royal Marsden Hospital NHS, Foundation Trust/Institute of Cancer Research, Sutton, United Kingdom; Section of Medicine, The Institute of Cancer Research, Sutton, UK and Drug Development Unit, The Royal Marsden Hospital, Sutton, United Kingdom; The Royal Marsden Hospital, Surrey, United Kingdom . 2011 ASCO Annual Meeting ,2555. |
| 641 | Array-based comparative genomic hybridization analysis to identify prognostic markers for resected pancreatic cancer. J. Lee, I. Petrini, J. Hwang, E. Giovannetti, J. Voortman, Y. Wang, S. M. Steinberg, N. Funel, P. S. Meltzer, Y. Wang, G. Giaccone; Medical Oncology Branch, National Cancer Institute, Bethesda, MD; Department of Internal Medicine, Seoul National University Bundang Hospital, Seongnam-si, Gyeonggi-do, South Korea; VU University Medical Center, Amsterdam, Netherlands; Genetic Branch, National Cancer Institute, Bethesda, MD; Biostatistics and Data Management Section, National Cancer Institute, National Institutes of Health, Bethesda, MD; U.O. Chirurgia Generale e dei Trapianti nell'Uremico e nel Diabetico, Azienda Ospedaliero-Universitaria Pisana, Pisa, Italy; National Cancer Institute, Bethesda, MD . 2011 ASCO Annual Meeting ,4097. |
| 642 | Diagnosis of breast cancer metastases with PET/TC in patients with elevation of tumor markers: First data update. A. Mafodda, A. Prestifilippo, D. Aric貌, A. Vadal脿, M. Mare, C. Fornito, D. Giuffrida; Mediterranean Institute of Oncology, Viagrande, Catania, Italy; Istituto Oncologico del Mediterraneo, Viagrande, Catania, Italy; ARNAS Garibaldi Nesima, Catania, Italy . 2011 ASCO Annual Meeting ,1541. |
| 643 | Circulating baseline plasma cytokines and angiogenic factors (CAF) as markers of tumor burden and therapeutic response in a phase III study of pazopanib for metastatic renal cell carcinoma (mRCC). Y. Liu, H. T. Tran, Y. Lin, A. Martin, A. J. Zurita, C. N. Sternberg, R. G. Amado, L. N. Pandite, J. Heymach, VEG105192 Team; Oncology Research and Development, GlaxoSmithKline, Collegeville, PA; University of Texas M. D. Anderson Cancer Center, Houston, TX; GlaxoSmithKline, Collegeville, PA; San Camillo-Forlanini Hospital, Rome, Italy; GlaxoSmithKline, Research Triangle Park, NC . 2011 ASCO Annual Meeting ,4553. |
| 644 | Imaging with [ C. A. Martin, R. F. Perini, A. N. Avadhani, M. Redlinger, K. Harlacker, K. T. Flaherty, M. A. Rosen, C. R. Divgi, P. J. O'Dwyer; Hospital of the University of Pennsylvania, Philadelphia, PA; Thomas Jefferson University Hospital, Philadelphia, PA; Abramson Cancer Center, University of Pennsylvania, Philadelphia, PA; Massachusetts General Hospital, Boston, MA; University of Pennsylvania, Philadelphia, PA . 2011 ASCO Annual Meeting ,3048. |
| 645 | Design of an open-label randomized phase II trial examining the effect of sequencing of sipuleucel-T and androgen deprivation therapy (ADT) on immune markers in prostate cancer patients with a rising prostate specific antigen (PSA) after primary therapy. E. S. Antonarakis, A. S. Kibel, D. W. Lin, R. C. Tyler, M. Tabesh, C. G. Drake; The Sidney Kimmel Comprehensive Cancer Center at Johns Hopkins University, Baltimore, MD; Washington University School of Medicine, St. Louis, MO; University of Washington, Seattle, WA; Dendreon Medcl Aff, Seattle, WA; Dendreon Corporation, Seattle, WA; Sidney Kimmel Comprehensive Cancer Center at Johns Hopkins University, Baltimore, MD . 2011 ASCO Annual Meeting ,TPS189 . |
| 646 | Preventing cancer in the colon: Effect of ginger root extract on markers of inflammation in colon mucosa in people at high risk for colorectal cancer. S. M. Zick, D. K. Turgeon, S. Vareed, M. T. Ruffin, Z. Djuric, A. J. Litzinger, B. Wright, D. P. Normolle, D. E. Brenner; University of Michigan, Ann Arbor, MI; Medical College of Georgia, Augusta, GA; University of Toledo, Toledo, OH; University of Pittsburgh, Pittsburgh, PA; University of Michigan Medical Center, Ann Arbor, MI . 2011 ASCO Annual Meeting ,1570. |
| 647 | SOX2-related micrornas, miR-145 and miR-367, as prognostic markers of time to recurrence (TTR) in surgically resected non-small cell lung cancer (NSCLC) patients (p). A. Navarro, M. Campayo, N. Vinolas, V. Ciria, T. Diaz, R. M. Marrades, L. Molins, J. Ramirez, M. Monzo; School of Medicine, University of Barcelona, IDIBAPS, Barcelona, Spain; Department of Medical Oncology, Hospital Clinic de Barcelona, Barcelona, Spain; Hospital Clinic de Barcelona, Barcelona, Spain; Department of Pneumology. ICT. IDIBAPS. Hospital Clinic de Barcelona, Barcelona, Spain; Thoracic Surgery, ICT, IDIBAPS, Hospital Clinic de Barcelona, Barcelona, Spain; Department of Pathology, CDB, IDIBPAS, Hospital Clinic de Barcelona, Barcelona, Spain . 2011 ASCO Annual Meeting ,10583. |
| 648 | Identification of pathogenic macrophages in breast cancer as markers of tumor aggressiveness. R. Mukhtar, A. P. Moore, V. Tandon, O. Nseyo, A. Au, F. L. Baehner, C. A. Adisa, N. Eleweke, O. I. Olopade, D. H. Moore, M. Campbell, L. Esserman; University of California, San Francisco, San Francisco, CA; Abia State University Teaching Hospital, Aba, Nigeria; The University of Chicago Medical Center, Chicago, IL; University of California, San Francisco Helen Diller Family Comprehensive Cancer Center, San Francisco, CA . 2011 ASCO Annual Meeting ,1043. |
| 649 | Soluble mesothelin, megakaryocyte potentiating factor, and osteopontin as markers of patient response and outcome in malignant pleural mesothelioma. K. Hollevoet, K. Nackaerts, R. Gosselin, W. De Wever, L. Bosqu茅e, P. De Vuyst, P. R. Germonpre, E. Kellen, C. Legrand, Y. Kishi, J. R. Delanghe, J. P. Van Meerbeeck; Department of Respiratory Medicine, Ghent University Hospital, Ghent, Belgium; Gasthuisberg University Hospital, Leuven, Belgium; Department of Radiology, Ghent University Hospital, Ghent, Belgium; Department of Radiology, University Hospital Gasthuisberg, Leuven, Belgium; Department of Respiratory Medicine, CHU Sart-Tilman, Li猫ge, Belgium; Department of Respiratory Medicine, Erasme Hospital ULB, Brussels, Belgium; Department of Respiratory Medicine, Antwerp University Hospital, Antwerp, Belgium; Department of Respiratory Medicine, Gasthuisberg University Hospital, Leuven, Belgium; Institut de Statistique, Biostatistique et Sciences Actuarielles, Universit茅 Catholique de Louvain, Louvain-la-Neuve, Belgium; Ina Institute, Medical and Biological Laboratories, Nagano, Japan; Department of Clinical Chemistry, Ghent University Hospital, Ghent, Belgium; Gent University Hospital, Gent, Belgium . 2011 ASCO Annual Meeting ,7086. |
| 650 | Growth factor receptors and cell cycle proteins as new molecular prognosis markers in high-grade undifferentiated pleomorphic sarcoma (HGUPS). C. Serrano, S. Simonetti, C. M. Valverde, R. Morales, C. Suarez, T. Moline, J. Carles, S. Ramon y Cajal, C. Romagosa; Oncology Department, Vall d'Hebron University Hospital, Barcelona, Spain; Pathology Department, Vall d'Hebron University Hospital, Barcelona, Spain; Vall d'Hebron University Hospital, Barcelona, Spain . 2011 ASCO Annual Meeting ,10077. |
| 651 | CpG island methylator phenotype and KRAS mutation status as prognostic markers in patients with resected colorectal cancer. D. H. Koo, Y. S. Hong, K. Kim, J. Lee, H. Chang, Y. Kang, C. S. Yu, J. C. Kim, M. Kim, S. J. Jang, T. W. Kim; Department of Oncology, Asan Medical Center, University of Ulsan College of Medicine, Seoul, South Korea; Asan Medical Center, Seoul, South Korea; Department of Colorectal Surgery, Asan Medical Center, University of Ulsan College of Medicine, Seoul, South Korea; Department of Pathology, Asan Medical Center, University of Ulsan College of Medicine, Seoul, South Korea . 2011 ASCO Annual Meeting ,3595. |
| 652 | Fulvestrant administered in two different schedules: Pharmacokinetics, biological markers, and activity. A. Felici, G. Naso, S. Di Segni, P. Vici, A. Antenucci, F. Angelini, L. Pizzuti, C. Mandoj, G. D'Auria, A. Fabi, A. Tata, F. Cognetti, P. Papaldo; Medical Oncology, Regina Elena National Cancer Institute, Rome, Italy; Division of Medical Oncology, Policlinico Umberto I, Roma, Italy; Laboratory of Pharmacokinetics, Regina Elena Cancer Institute, Rome, Italy; Clinical Pathology, Regina Elena Cancer Institute, Rome, Italy; O.U. of Oncology, Ospedale Regina Apostolorum, Albano Laziale, Italy; Medical Oncology, Belcolle Hospital, VIterbo, Italy; Regina Elena National Cancer Institute, Rome, Italy . 2011 ASCO Annual Meeting ,e11026 . |
| 653 | Chemotherapeutic drugs induced apoptosis-related mRNAs in whole blood ex vivo as a new diagnostic markers for the prediction of clinical outcome of AML. N. Usui, K. Mitani, T. Maeda, T. Sakura, N. Dobashi, F. Yagasaki, K. Obata, M. Mitsubashi, S. Miyawaki; Jikei University School of Medicine, Tokyo, Japan; Dokkyo Medical University, Tochigi, Japan; Saitama Medical University, Saitama, Japan; Saiseikai Maebasshi Hospital, Gunmma, Japan; Hitachi Chemical Co., Ltd., Ibaraki, Japan; Hitachi Chemical Research Center, Irvine, CA; Metropolitan Ohtsuka Hospital, Tokyo, Japan . 2011 ASCO Annual Meeting ,6627. |
| 654 | Long-term prognostic factors of node-negative invasive breast cancer of luminal subtype: A comparison between histologic grades and molecular markers including Ki-67 and HER2. M. Ono, H. Tsuda, C. Shimizu, K. Yonemori, M. Ando, K. Tamura, N. Katsumata, T. Kinoshita, Y. Fujiwara; National Cancer Center Hospital, Tokyo, Japan . 2011 ASCO Annual Meeting ,e11050 . |
| 655 | The biology of early-onset colorectal cancer: An examination of tumor markers, pathology, and survival in a large cohort of patients. S. A. Khan, M. Morris, K. Idrees, M. Gimbel, S. Rosenberg, Z. Zeng, J. Shia, M. P. La Quaglia, P. Paty; The University of Chicago, Chicago, IL; University of Western Australia, Crawley, Australia; Memorial Sloan-Kettering Cancer Center, New York, NY . 2011 ASCO Annual Meeting ,3537. |
| 656 | The role of KRAS, BRAF, and PI3K mutations as markers of resistance to cetuximab in metastatic colorectal cancer. A. Passardi, P. Ulivi, M. Valgiusti, E. Scarpi, R. Moscati, E. Chiadini, P. Rosetti, L. Saragoni, L. Capelli, A. Casadei Gardini, A. Ragazzini, M. Monti, S. Calpona, W. Zoli, C. Milandri, L. Frassineti; IRST, Meldola, Italy; Unit of Biostatistics and Clinical Trials, Istituto Scientifico Romagnolo per lo Studio e la Cura dei Tumori, Meldola, Italy; Istituto Scientifico Romagnolo, Meldola, Italy; Biosciences Laboratory, IRST, Meldola, Italy; Department of Medical Oncology, I.R.S.T., Meldola, Italy . 2011 ASCO Annual Meeting ,3603. |
| 657 | Circulating endothelial cells, endothelial progenitor cells, microparticles, and markers of inflammation and coagulation in advanced non-small cell lung cancer. T. Fleitas, G. Reynes, V. Martinez-Sales, V. Vila, E. Reganon, D. Mesado, M. Martin, J. G贸mez-Codina, J. Montalar; Hospital Universitario La Fe, Valencia, Spain; Hospital La Fe, Valencia, Spain . 2011 ASCO Annual Meeting ,7583. |
| 658 | A study of L. Horvath, K. L. Mahon, W. Qu, J. Devaney, M. D. Chatfield, C. Paul, R. Wykes, M. J. Boyer, M. R. Stockler, G. M. Marx, R. L. Sutherland, S. J. Clark; Sydney Cancer Centre, Sydney, Australia; Garvan Institute of Medical Research, Sydney, Australia; National Centre for Biomedical Engineering Science, Galway, Ireland; NHMRC Clinical Trials Centre, University of Sydney, Sydney, Australia; Royal Prince Alfred Hospital, Sydney, Australia; Sydney Haematology Oncology Clinics, Sydney, Australia; Australian New Zealand Breast Cancer Trials Group, University of Newcastle, Newcastle, Australia . 2011 ASCO Annual Meeting ,4603. |
